# Sources and sinks: Geoclimatic dynamics shaped the assembly of endemic fauna in the world’s Third Pole

**DOI:** 10.1073/pnas.2602323123

**Published:** 2026-10-26

**Authors:** Dechun Jiang, Mao-Liang Li, Jin-Long Ren, Rui-Han Li, Zhi-Tong Lyu, Sebastian Klaus, Ru-Wan Jia, Tao Xue, Jia-Tang Li

**Affiliations:** ^a^ Mountain Ecological Restoration and Biodiversity Conservation Key Laboratory of Sichuan Province and Key Laboratory of National Forestry and Grassland Administration on Biodiversity Conservation on the Qinghai-Xizang Plateau, Chengdu Institute of Biology, Chinese Academy of Sciences, Chengdu 610213, China; ^b^ China-Croatia Belt and Road Joint Laboratory on Biodiversity and Ecosystem Services and National Engineering Research Center for Natural Medicines, Chengdu Institute of Biology, Chinese Academy of Sciences, Chengdu 610213, China; ^c^ University of Chinese Academy of Sciences, Beijing 100049, China; ^d^Southeast Asia Biodiversity Research Institute, Chinese Academy of Sciences, Nay Pyi Taw 05282, Myanmar; ^e^ Department of Ecology and Evolution, J. W. Goethe University, Frankfurt am Main 60438, Germany

**Keywords:** biogeography, the Qinghai–Tibet Plateau, mountain biology, phylogenetics, multiple taxa

## Abstract

The Qinghai–Tibet Plateau (QTP), a biodiversity hotspot shaped by tectonic and climatic forces, has seen significant progress in understanding its floristic origins. However, a comprehensive, multitaxon perspective on how its terrestrial fauna assembled remains lacking. Here we integrate time-calibrated phylogenies of over 20,000 species from five major groups (invertebrates, amphibians, reptiles, birds, and mammals), to reconstruct the evolutionary history of 527 terrestrial animal species endemic to the QTP. Our finding reveals that the QTP has functioned both as a center of in situ diversification and as a “biogeographic sink” that receives species from surrounding regions, however, with pronounced subregional differentiation: the Hengduan Mountains acted primarily as a diversification source, while the Himalaya and the Plateau Platform acted as sinks, accumulating lineages from adjacent regions. The Eocene–Oligocene climatic transition, rather than late Neogene uplift, initiated sustained faunal diversification and biotic exchange. Across subregions, distinct uplift histories and monsoon regimes drove asynchronous evolutionary trajectories, showing that even geographically adjacent regions can exhibit distinct accumulation patterns of lineages. Notably, integrating our faunal evidence with recent floristic assembly data reveals a consistent “plants first, animals later” pattern across the QTP and its subregions, suggesting that early-arriving plant lineages created ecological niches that facilitated subsequent faunal colonization and diversification. This framework advances our understanding of how mountain biotas assemble over time, with implications extending far beyond the QTP.

## Significance

The Qinghai–Tibet Plateau (QTP), known as the “Third Pole” due to its unique geographic and climatic conditions, is one of the most biodiverse regions on Earth, harboring many species found nowhere else. Our study reveals that its rich animal communities began assembling rapidly after a major global climate shift around 34 million years ago, much earlier than the QTP’s late-stage uplift, suggesting that climate-driven ecological changes were the primary trigger. Fauna and flora of different subregions of the QTP took very different evolutionary routes caused by different uplift histories and monsoon patterns. The entire process followed a “plants first, animals later” pattern, thereby revealing a predictable, cross-kingdom sequence in the formation of this biodiversity hotspot.

**Fig. 1.**
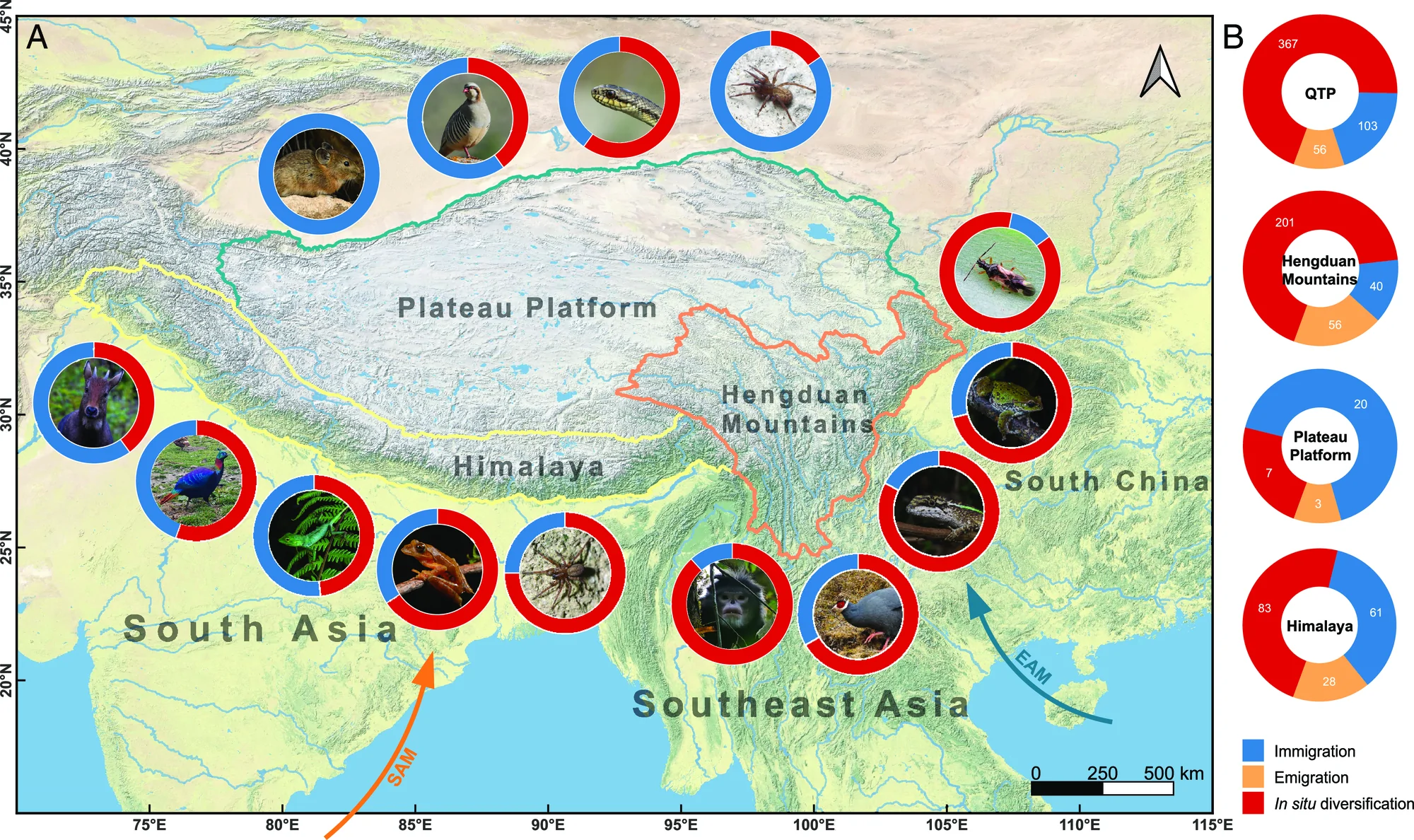
The Qinghai-Tibet Plateau (QTP) region, adjacent regions, and associated biogeographical events. (*A*) Topographic map of the Qinghai–Tibet Plateau (QTP) and surrounding regions with boundaries referenced from Liu et al. (1). Circular photographs show representative endemic lineages for each region, presented in geographic order. *Top* (Plateau Platform, from *Right* to *Left*): invertebrates (*Draconarius yigongensis*, photographed by Qianle Lu), reptiles (*Thermophis baileyi*, photographed by Junjie Huang), birds (*Alectoris magna*, photographed by Jun Tang), and mammals (*Ochotona ladacensis*, photographed by Junjie Huang). *Right* (Hengduan Mountains, from *Right* to *Left*): invertebrates (*Anthocoris* sp. photographed by Fan Gao), amphibians (*Zhangixalus dugritei*, photographed by Maoliang Li), reptiles (*Diploderma swild*, photographed by Maoliang Li), birds (*Crossoptilon auritum*, photographed by Ziyang Huang), and mammals (*Rhinopithecus strykeri*, photographed by Lei Dong). *Bottom* (Himalaya, from *Right* to *Left*): invertebrates (Coelotinae sp. photographed by Xinyu Luo), amphibians (*Rhacophorus translineatus*, photographed by Junjie Huang), reptiles (*Calotes medogensis*, Photographed by Maoliang Li), birds (*Lophophorus impejanus*, photographed by Shuangqi Liu), and mammals (*Naemorhedus goral*, photographed by Zongzhuang Liu). Colored outer rings around each photograph indicate the proportional contributions of immigration (blue) and in situ diversification (red) to the endemic lineages of that region. SAM: South Asian Monsoon; EAM: East Asian Monsoon. (*B*) Summary of dispersal and in situ diversification events of QTP and its three subregions.

The region of the Qinghai-Tibet Plateau (QTP) sensu lato, known as the “Third Pole”, encompasses a vast area of 3.07 × 10^6^ km^2^, slightly smaller than India (3.29 × 10^6^ km^2^) but larger than Argentina (2.78 × 10^6^ km^2^), ranging in elevation from 88 to 8,848 m (1). This region includes three major subregions: the Plateau Platform, the Himalaya, and the Hengduan Mountains (1, 2) (Fig. 1). The complex tectonic history of the QTP region, initiated by the India–Eurasia collision (ca. 65 to 61 Ma) and followed by phases of asynchronous rapid uplift and erosion across the different subregions (3), raises a key question: how local biotas responded to this Cenozoic orogeny and climatic history, and how these interacting processes affected regional biodiversity.

Biologically, this geologically dynamic region sustains exceptional biodiversity, with two of the three subregions long recognized as “biodiversity hotspots” (4). The Hengduan Mountains form one such center, hosting ~12,000 vascular plant species (including 24 genera endemic to the Hengduan Mountains), >4,800 insect species, more than half of China’s avifauna, and over one-third of its herpetofauna (4–9). The Himalaya constitutes another biodiversity hotspot, harboring ~10,000 plant species (including 3,500 species endemic to the Himalaya), 124 amphibian, 194 reptile, 987 bird, and 240 mammal species (10, 11). In contrast, the Plateau Platform, while less biodiverse, is renowned for its unique endemism. It harbors a range of highly adapted species such as *Thermophis* spp. (hot-spring snakes), *Phrynocephalus* spp. (Toad-headed agamas), *Pantholops hodgsonii* (Tibetan antelope), and *Ochotona* spp. (Pikas) (12).

The origins of this spectacular diversity and unique degree of endemism have thus attracted considerable scientific interest. In the botanical realm, causal relationships between floral diversification and abiotic factors such as monsoon intensification, mountain uplift, and glacial cycles (9, 13) have been proposed based on phylogenetic data. In detail, the Sino-Himalayan flora experienced major shifts in composition, from a temperate regime predating 50 Ma, to a tropical one during 30 to 50 Ma, and back to a temperate dominance thereafter (14). Concerning the fauna, however, a corresponding synthesis has been hindered because relevant studies are typically restricted to specific taxa (15–17) or specific subregions (18, 19).

Over recent decades, numerous hypotheses have been proposed regarding the timing and mode of uplift of the QTP (3), ranging from ancient uplift as early as the Paleocene (20) to later phases during the Pliocene (21, 22). Recent geological syntheses increasingly support a scenario in which the Plateau Platform and the Hengduan Mountains underwent rapid uplift during the mid–late Eocene, reaching near-modern elevations (3), whereas the major uplift phase of the Himalaya occurred during the late Oligocene to early Miocene (23). Due to these long-standing geological controversies, many phylogenetic studies on QTP biota have linked recent speciation events to late-stage orogenic uplift, an assumption that may not align with updated geological evidence. Renner (24) reviewed 95 such studies attributing Miocene–Pleistocene diversification to the merely hypothetical “recent uplift” of the QTP. The paradigm shift concerning the age of the QTP suggests that other abiotic drivers such as climatic oscillations, river incision, and glacial-interglacial dynamics, may still be relevant factors that influenced diversification after the major orogeny.

Importantly, different taxa can respond idiosyncratically to the same geological processes due to their specific biological and ecological traits, leading to incongruent biogeographical patterns across taxa (24–26). Accordingly, previous biogeographic studies on the QTP fauna have proposed contrasting scenarios based on the area identified as major faunal donor. This includes the “out of Himalaya” hypothesis (27), the “out of Tibet” hypothesis (17, 28–30), the “into and out of the Qinghai-Tibet Plateau and the Himalayas” hypothesis (31), the “out of the Himalaya–Hengduan Mountains” hypothesis (32), and the “out of the Hengduan Mountains” hypothesis (33). However, these hypotheses have largely been based on single-clade phylogenies or isolated fossil discoveries, and have not been rigorously tested using multitaxon analyses to generalize patterns found. In addition, inconsistencies in the geographical definitions of the Plateau Platform, the Himalaya, and the Hengduan Mountains have further complicated comparisons among these scenarios. Therefore, comparative and integrative studies that incorporate multiple taxonomic groups, along with both updated geological reconstructions and paleoclimatic simulations, are needed to provide a more robust framework for understanding how the coupled orogenic and climatic history has shaped the biodiversity of the QTP over time.

In this study, we reconstructed the assembly history of the terrestrial fauna endemic to the QTP region by analyzing a comprehensive set of phylogenies encompassing more than 20,000 vertebrate and invertebrate species. We reconstructed the evolutionary trajectories of 527 species within the dataset that are endemic to the QTP region, quantifying biotic exchange both between the QTP region and adjacent regions, and between different subregions. Specifically, we tested two hypotheses: i) mountain uplift generally triggers faunal speciation. If true, we predict that earlier-uplifted subregions should harbor faunal assemblages with deeper divergence times and higher diversification than later-uplifted ones, with key node ages specifically coinciding with mid–late Eocene uplift in the former but showing younger peaks in the latter; and ii) periods of major floral compositional shifts should generally be paralleled by faunal assembly, because both experienced the same abiotic environmental changes. If true, we predict that we can detect similar patterns of faunal diversification that are tied to the known transitions between tropical and temperate climates. Finally, we contextualized these general evolutionary patterns within an updated framework of geological and climatic events. Thus, our integrative studies allow for a unified understanding of biotic assembly and its tectonic and climatic drivers across the entire QTP region including its subregions.

## Results

### The Hengduan Mts. are the Speciation-Hotspot Within the QTP Area.

The three subregions analyzed in our study—the Hengduan Mountains (HDM), the Himalaya (HIM), and the Plateau Platform (TP)—were delimited based on distinct tectonic histories, topographic configurations, and monsoon regimes (1). Within our phylogenetic dataset we identified 527 species endemic to the QTP, comprising 145 invertebrate, 112 amphibian, 108 reptile, 69 bird, and 93 mammal species (Datasets S1 and S2). At the regional level, we detected 367 in situ speciation events, 103 immigration events, and 56 emigration events (Fig. 1*B* and *SI Appendix*, Tables S16 and S17). The distribution of these events, however, differed markedly among the subregions (Fig. 1*B*). The Hengduan Mountains recorded the highest number of in situ speciation events (201), followed by the Himalaya (34). In both regions, in situ speciation was the dominant biogeographical process. A key difference emerged in their patterns of biotic exchange: the Hengduan Mountains showed greater emigration than immigration events (56 vs. 40), supporting a net source role. The Himalaya displayed the opposite pattern, with immigration greatly exceeding emigration (61 vs. 28), identifying it as a sink. In contrast, the Plateau Platform showed minimal in situ speciation (7 events), with its fauna being largely shaped by immigration (20 events).

### Southeast Asia Is a Major Source of Diversity for the QTP.

To identify the geographic origins and destinations of biotic exchange with the QTP, we quantified immigration and emigration events between the QTP and six adjacent biogeographic regions. Our results delineate clear pathways of faunal exchange between the QTP and its surroundings. Southeast Asia served as the principal biogeographic corridor, contributing 35 immigration events to the QTP and receiving 18 emigration events from it—the highest in both categories (*SI Appendix*, Fig. S2 and Table S17). South China was the second most significant region, facilitating 25 immigrations and 12 emigrations (*SI Appendix*, Fig. S2 and Table S17).

At the subregional scale, patterns of biotic exchange show clear spatial heterogeneity. The Hengduan Mountains exhibit most frequent exchange with southern China (18 immigration events vs. 9 emigration events), functioning as both a recipient of lineages and a hub for diversity export. The Himalaya show a strong immigration bias from Southeast Asia (23 immigration events vs. 9 emigration events), suggesting they act primarily as a frontier for south-to-north input, with topographic and climatic gradients facilitating high-altitude adaptation. In contrast, the Plateau Platform has the most limited exchange with surrounding regions; its biota derives mainly from immigration from the Hengduan Mountains (9 events), with only rare outward dispersal (2 events to the Himalaya), supporting its role as a species accumulation zone rather than a dispersal source (*SI Appendix*, Fig. S2 and Table S17).

### Regional-Level Temporal Dynamics of Faunal Assembly.

**Fig. 2.**
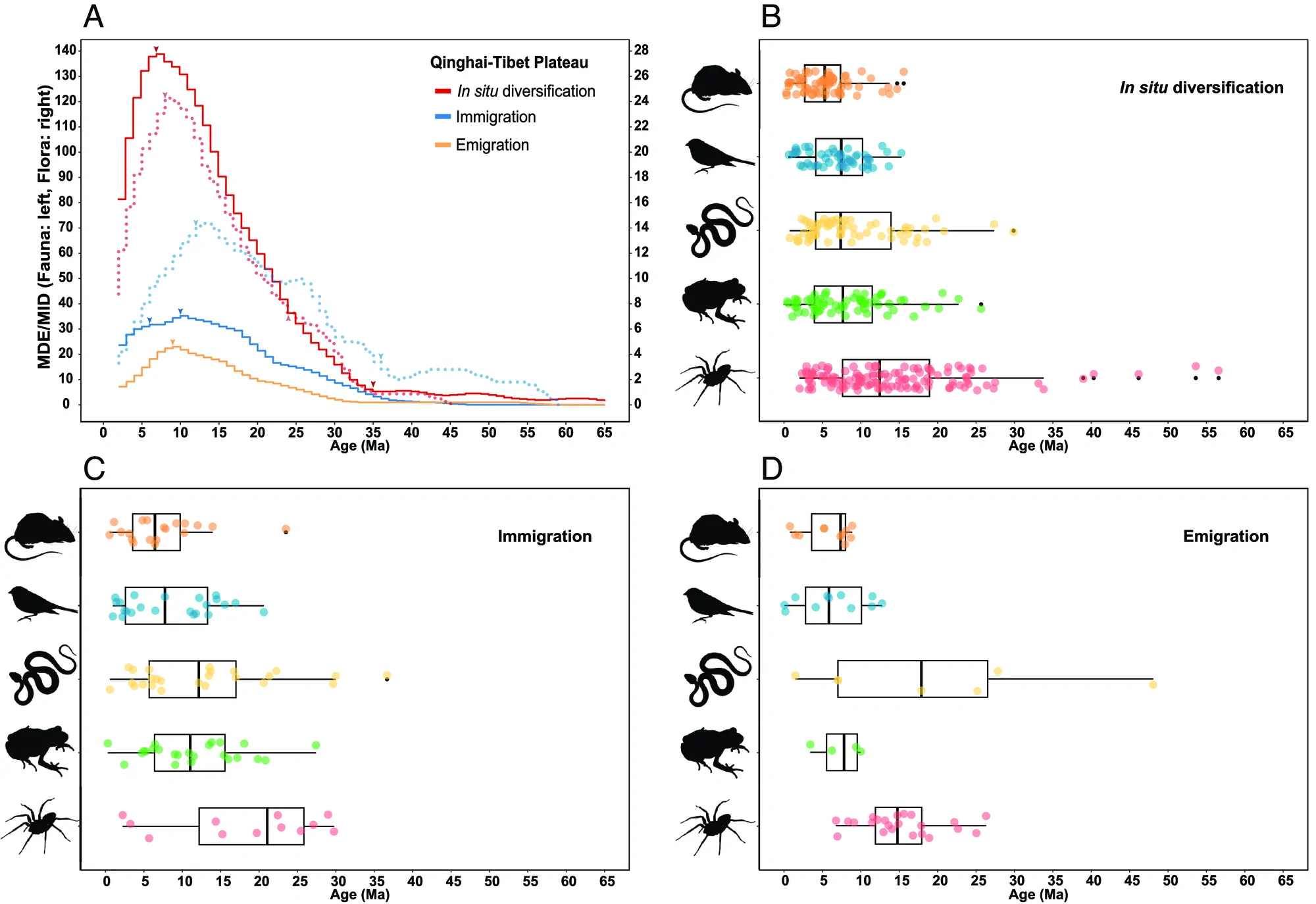
Temporal dynamics of in situ diversification and faunal exchange in the Qinghai-Tibet Plateau. (*A*) MID (the maximum number of in situ diversification events per million years) and MDE (the maximum number of dispersal events per million years) curves of in situ diversification (red), immigration (blue), and emigration (orange) events since the 65 Ma, dotted lines indicate temporal trajectories of origin events of QTP’s endemic flora modified from Cao et al. (9), arrows indicate change points of trend. (*B*–*D*) Boxplots showing the temporal distributions of in situ diversification (*B*), immigration (*C*), and emigration (*D*) events across major taxonomic groups (from *Top* to *Bottom*: mammals, birds, reptiles, amphibians, and invertebrates).

To directly test hypothesis (ii)—that periods of major floral compositional shifts should generally be paralleled by faunal assembly due to shared abiotic drivers—we examined the timing of faunal assembly across the entire QTP by constructing the accumulation of in situ diversification and dispersal over the past 65 million years. The reconstruction of in situ diversification (the maximum number of in situ diversification events per million years, MID) and dispersal (the maximum number of dispersal events per million years, MDE) over the past 65 million years reveals a dynamic and nonlinear history of faunal assembly in the QTP (Fig. 2*A*). The period from 65 Ma until the late Eocene was characterized by a low and slowly increasing background level of in situ diversification (MID at 1.6 events/Ma by 65 Ma with a slow rise to 5.4 events/Ma by 35 Ma) (*SI Appendix*, Table S18), while dispersal events were nearly absent (MDE generally ≤1) (*SI Appendix*, Table S18). A major transition began in the middle Eocene, with immigration events starting a continuous rise around 46 Ma. This was followed by a more persistent increase in emigration from approximately 32 Ma onward and an acceleration in in situ diversification around 35 Ma. (Fig. 2*A*). The most dramatic increases occurred during the Miocene: the MID accumulation rate rose substantially between 24 to 16 Ma (averaging 5.18 events/Ma) and then sharply between 15 to 13 Ma (averaging 6.73 events/Ma). (Fig. 2*A* and *SI Appendix*, Table S18). After 13 Ma, the growth of MID slowed, peaking with a value of 138.8 at 7 Ma before declining rapidly after 5 Ma (Fig. 2*A*).

### Taxonomic-Level Heterogeneity in Diversification and Dispersal.

We next asked whether different faunal groups exhibit distinct temporal patterns of biogeographic events. The following results are based on median ages of in situ diversification, immigration, and emigration events for each taxonomic group. A clear temporal hierarchy in biogeographic events emerged across taxonomic groups (Fig. 2 *B*–*D*). Invertebrates consistently underwent diversification, immigration, and emigration earliest, with median ages of 12.5 Ma, 21.1 Ma, and 14.8 Ma, respectively (Fig. 2 *B*–*D*). Reptiles and amphibians generally followed in the mid-Miocene, while birds and mammals, particularly the latter (median age of in situ diversification: 5.4 Ma; immigration: 6.5 Ma; emigration: 7.4 Ma), were the last to diversity and disperse (Fig. 2 *B*–*D*). Notably, the CI for invertebrates and reptiles were substantially wider, indicating a more protracted history of faunal exchange compared to the more recent and narrowly-timed events like mammals and amphibians. (Fig. 2 *B*–*D*).

MID and MDE analyses of the QTP further showed that all groups exhibited a unimodal pattern of in situ diversification and emigration, except reptiles, with invertebrates displaying the earliest peak at approximately 13 Ma (*SI Appendix*, Fig. S5). In contrast, immigration events revealed a multipulse pattern for most groups except amphibians. Invertebrates and birds showed their first major peaks at ~25 Ma and ~15 Ma, respectively, followed by a shared decrease around 6 Ma and a subsequent continuous increase in birds and invertebrates toward the present (*SI Appendix*, Fig. S5).

### Subregional-Level Evolutionary Trajectories.

**Fig. 3.**
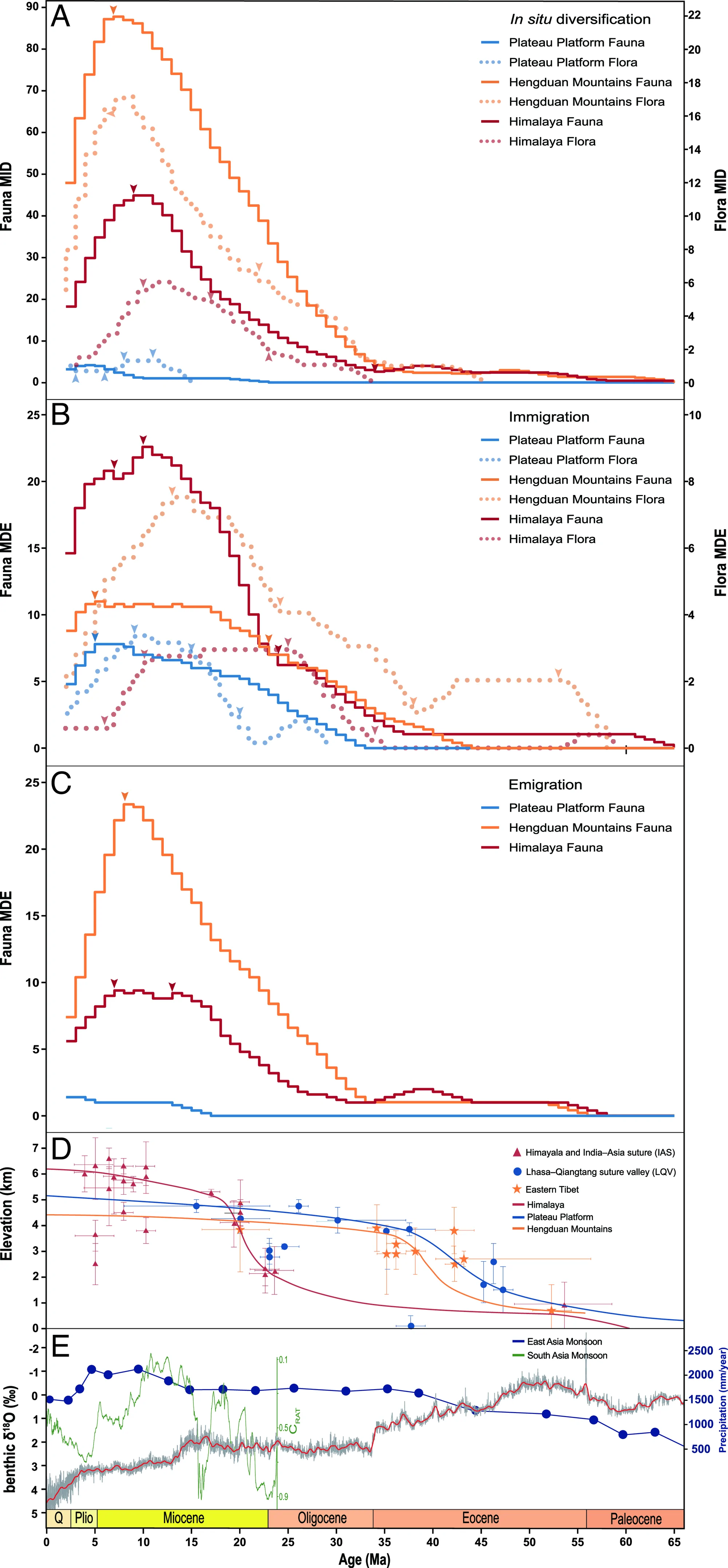
Temporal dynamics of in situ diversification, biogeographic exchange, and environmental change in three subregions of QTP region. (*A*–*C*) MID (the maximum number of in situ diversification events per million years) and MDE (the maximum number of dispersal events per million years) curves of in situ diversification (*A*), immigration (*B*), and emigration (*C*) across the three subregions of QTP, including the Plateau Platform (blue), Hengduan Mountains (orange), and Himalaya (red), dotted lines indicate temporal trajectories of origin events of three subregions endemic flora modified from Cao et al. (9), arrows indicate change points of trend. (*D*) Paleoelevation reconstructions for the three subregions of QTP modified from (23). (*E*) Long-term climate records showing benthic δ^1^^8^O (gray curve with smoothed red line) as a proxy for global temperature and ice volume (35), together with reconstructions of South Asian Monsoon (SAM) intensity since 23.8 Ma (green line) based on the chemical weathering index (CRAT) (36), and East Asian Monsoon (EAM) intensity since 66 Ma (blue line) inferred from modeled mean annual precipitation (mm) (37).

To test hypothesis (i)—that earlier-uplifted subregions should harbor deeper divergence times and higher diversification than later-uplifted ones, with key node ages tied to mid–late Eocene uplift in the former but younger peaks in the latter—we compared the MID and MDE curves over time across the Hengduan Mountains (HDM), Himalaya (HIM), and Plateau Platform (TP) to assess whether the three subregions follow similar or divergent evolutionary trajectories (Fig. 3). Our results show that three QTP subregions exhibited starkly distinct evolutionary trajectories, as reflected in their MID and MDE curves (Fig. 3). In situ diversification (MID) initiated synchronously around 35 Ma in both the Hengduan Mountains (HDM) and the Himalaya (HIM). However, their subsequent paths diverged: MID of the Hengduan Mountains increased steadily to a peak at 7 Ma before a rapid decline, whereas MID of the Himalaya rose gradually before a sharp acceleration that culminated in a peak around 10 Ma, followed by a steady decrease. MID of the Plateau Platform showed no pronounced fluctuations, with only minor fluctuations at ~22 Ma and ~9 Ma (Fig. 3*A*).

Immigration (MDE) began earliest in the Himalaya (~64 Ma), remaining low until 36 Ma, then rising through a series of phases to peak at 11 to 10 Ma before declining after 3 Ma (Fig. 3*B*). The Hengduan Mountains exhibited steadier and later immigration peaks (~16 to 5 Ma) (Fig. 3*B*). The Plateau Platform showed a delayed and more gradual increase, peaking last at ~7 to 5 Ma (Fig. 3*B*).

Emigration began early in both the Hengduan Mountains and Himalaya (Fig. 3*C*). In the Hengduan Mountains, emigration rose sharply after ca. 32 Ma, peaked at 9 to 8 Ma, and then declined markedly (Fig. 3*C*). The Himalaya showed a more complex history with an early pulse (43 to 34 Ma) and twice Miocene peaks (Fig. 3*C*). In contrast, The Plateau Platform showed negligible emigration, with only minimal activities after 16 Ma (Fig. 3*C*).

### Robustness of Inferred Diversification and Dispersal Dynamics.

Bootstrap analyses recovered temporal patterns highly consistent with the original MID and MDE curves, supporting the robustness of the inferred diversification and dispersal dynamics (*SI Appendix*, Fig. S4). In contrast, randomized MID and MDE curves differed substantially from the empirical trajectories (*SI Appendix*, Fig. S7). The absence of the major empirical peaks in the randomized datasets indicates that the observed temporal dynamics are not artifacts of phylogenetic structure or lineage accumulation, but are primarily driven by nonrandom biogeographic events.

### Predictors of Dispersal Events.

In the main (subregional) analysis, the retained predictors were largely independent of one another, except that area and geographic isolation remained strongly correlated after perimeter had been removed (r = 0.91); total branch length was only weakly correlated with the other predictors (r ≤ 0.39; *SI Appendix*, Tables S23 and S24).

**Fig. 4.**
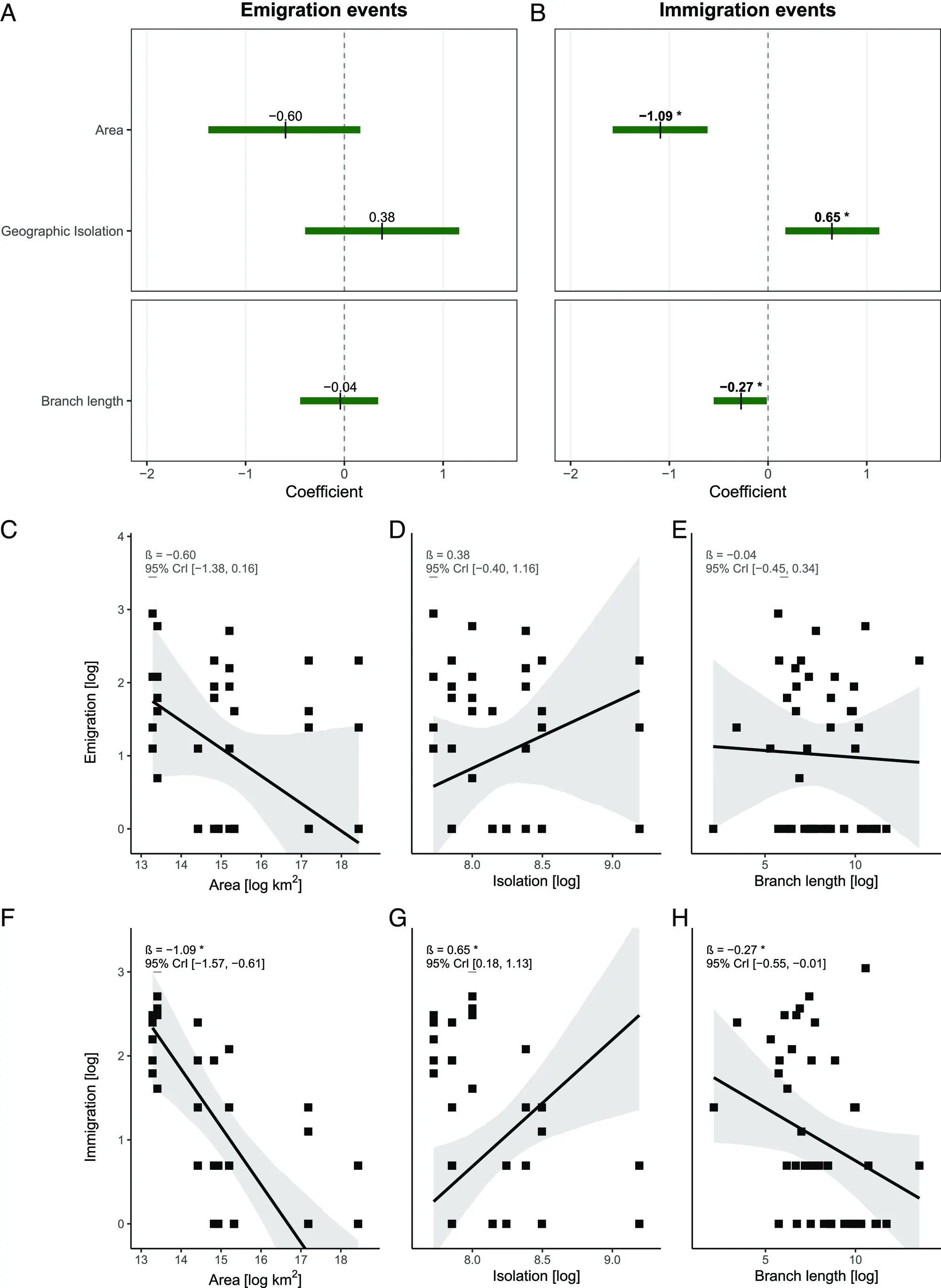
Relationship between the number of dispersal events out of (emigration) and into (immigration) a region and predictor variables across three independent subregions of the QTP (Scheme 2). (*A* and *B*) Regression coefficients of a Bayesian multiple linear mixed-effects model calculated based on all subregions and clades for emigration and immigration events, respectively. Green horizontal bars indicate the posterior mean and 95% credible intervals (CrI) of each regression coefficient; asterisks (*) denote predictors whose 95% CrI does not include zero. (*C*–*E*) Partial fit of emigration events against area, geographic isolation, and branch length, respectively; (*F*–*H*) Partial fit of immigration events against the same three predictor variables. Fitted lines show the posterior mean predicted values from the same model, assuming average values for all other predictors, whereas the points show the raw data. Shaded areas show 95% credible intervals. All variables are log-transformed.

No predictor had an important effect on emigration: the 95% credible intervals for area (β = − 0.60, 95% CrI [−1.38, 0.16]), geographic isolation (β = 0.38, [−0.40, 1.16]), and total branch length (β = −0.04, [−0.45, 0.34]) all included zero (Fig. 4 *A* and *C–E*). For immigration, all three predictors had important effects (Fig. 4 *B* and *F–H*): immigration decreased with area (β = −1.09, [−1.57, −0.61]), increased with geographic isolation (β = 0.65, [0.18, 1.13]), and decreased with total branch length (β = −0.27, [−0.55, −0.01]). In the subregional framework, then, regions that were smaller, more isolated, and had accumulated less lineage time received proportionally more immigration events.

In the sensitivity analysis treating the QTP as a single region, no predictor had an important effect on either emigration (area β = 0.52, [−0.35, 1.36]; geographic isolation β = −0.73, [−1.59 0.16]; total branch length β = 0.13, [−0.29, 0.57]) or immigration (area β = 0.06, [−0.76, 0.88]; geographic isolation β = −0.41, [−1.25, 0.43]; total branch length β = −0.10, [−0.51, 0.31]); all 95% credible intervals included zero (*SI Appendix*, Fig. S8). Full coefficient estimates and model residuals are given in *SI Appendix*, Table S22.

## Discussion

### The Dual Role of the QTP as a Diversification Center and Biogeographic Sink.

Our results reveal that most of the endemic faunal diversity in QTP originated through in situ diversification, with the Hengduan Mountains and the Himalaya acting as major centers of speciation (Fig. 1*B*). In terms of biogeographic exchange, immigration into QTP occurred at nearly twice the number of emigration, providing strong support for the “Into QTP” scenario (Fig. 1*B*). This pattern was particularly evident in the Himalaya and the Plateau Platform, where immigration significantly exceeded emigration (Fig. 1*B*). In contrast, the Hengduan Mountains not only hosted the highest frequency of in situ diversification but also acted as a regional source, with more emigration than immigration (Fig. 1*B*). Geologically, the Hengduan Mountains and the Plateau Platform uplifted earlier than the Himalaya (3, 23, 38), marking the initial stage in the formation of the QTP’s endemic fauna. However, their differential geological and geomorphological development also led to contrasting faunal evolutionary patterns. The Hengduan Mountains underwent a series of geological processes (river incision, river capture, glacial erosion, etc.) that generated one of the world’s most topographically diverse regions (3, 23, 39–45), supposed to greatly promote biological diversification (3, 13, 23). In contrast, the Plateau Platform was transformed from a humid basin into an alpine plateau with extreme environmental conditions, which drastically reshaped the preexisting biota (23, 46, 47). The vast, relatively uniform plateau landscape likely further limited in situ speciation due to low geographic isolation and niche heterogeneity. The Himalaya, which rose much later (3, 38), received numerous immigrant lineages from Southeast Asia and the Hengduan Mountains already harboring a diversified fauna (*SI Appendix*, Fig. S2). Its dramatic relief and thus wide range of habitats further facilitated local diversification, making the Himalaya both “sink” and “source” of biodiversity within the QTP. In general, our findings suggest that QTP functions both as a diversification center and a biogeographic sink for Eurasian biodiversity, while its different subregions play distinct roles: the Hengduan Mountains as a speciation hotspot and source area, and the Himalaya and Plateau Platform as important sinks. This highlights the multifaceted role of QTP in shaping overall Asian biodiversity.

Our study revealed a significant contribution of Southeast Asia to the biodiversity of the QTP region (*SI Appendix*, Fig. S2). Since the Eocene, Southeast Asia was dominated by a warm and humid climate and likely already supported broad-leaved evergreen forests (*SI Appendix*, Fig. S6 and Table S19) (48–50). In contrast, the QTP region was initially hot and dry but became increasingly humid by the late Eocene as the Asian monsoon expanded northward to 26–28°N, reaching the southern Hengduan Mountains and the Himalaya (50). Our MDE analysis indicates that dispersal from Southeast Asia began around 40 Ma into the Hengduan Mountains and around 35 Ma immigration preceded emigration (*SI Appendix*, Fig. S3). This corresponds to the onset of monsoon-driven humidification and supports a south-to-north evolution of the Asian monsoon system (50). A similar temporal pattern is observed for South China, the second major contributor to QTP’s endemic fauna. Fossil assemblages indicate that broad-leaved evergreen forests were first established in tropical China during the middle Eocene and subsequently expanded by the late Eocene to early Oligocene (*SI Appendix*, Fig. S6 and Table S19) (51–53). Consistent with this, our MDE results show faunal immigration from South China into the Hengduan Mountains beginning around 35 Ma (*SI Appendix*, Fig. S3), suggesting that this expansion of evergreen forests facilitated biogeographic exchange. In contrast, South Asia contributed only six immigration events and received a single emigration event (*SI Appendix*, Fig. S2). The Indian Plate’s separation from Asia by the Tethys Ocean and subsequently by the Gangdese–Himalayan orogeny (23), together with its extensive lowland plains, greatly limited direct faunal interchange with the QTP and possibly also hampered adaptation to the high-elevation Himalayan environment on a larger scale.

### The Eocene–Oligocene Transition: A Pivotal Trigger for Faunal Assembly.

The Eocene–Oligocene climate transition (EOT), which occurred around 34 Ma, was characterized by rapid global cooling which reduced sea levels (35, 54), widespread extinctions (55), and profound biotic turnover that laid the foundation for modern ecosystems (55). Our analyses reveal a pronounced acceleration in the accumulation of speciation events in the QTP region following the EOT (Figs. 2*A* and 3 *A*–*C*). This pattern is not unique to QTP; similar bursts of diversification have been documented globally across both fauna and flora lineages based on fossil evidence (51, 56), and molecular phylogenetic studies (57–60). For instance, *Hemidactylus* geckos diversified in Peninsular India during EOT cooling (58), and turtles underwent a burst of climate-associated diversification on continental margins worldwide (60). Taken together, these findings strongly support the interpretation that the EOT represented a pivotal trigger in the assembly of modern biotas, with its climatic upheavals fostering ecological opportunities that promoted rapid diversification.

### Asynchronous Evolution Driven by Subregional Geoclimatic Histories.

Recent studies on the geological history of QTP region have revealed distinct processes that were active in the three QTP subregions (Fig. 3*D*). The diversification patterns described here do reflect these geological processes, likely pointing to a causal relationship.

The central part of the Plateau Platform is first a basin situated between the Gangdese Mountains and the Watershed Mountains that was no higher than 2,000 m till middle Eocene (45 to 40 Ma) (23), and may have achieved an elevation over 4,000 m by late Eocene to late Oligocene (23, 61, 62). During the formation of the Plateau Platform, the environment underwent a dramatic transformation, shifting from lowland forests along westward-flowing rivers that served as migration corridors in the early to middle Eocene, to a cold and arid plateau by the Miocene (3). Following the major uplift, most of the dominant lowland and subtropical taxa became extinct, opening new ecological niches for preadapted clades to colonize. The plateau pika (*O. ladacensis*), for example, colonized the region via dispersal from lower elevations and subsequently adapted to the harsh environment through enhanced thermogenesis and specialized burrowing behavior (63–65). In contrast, the hot-spring snake (*T. baileyi*) exhibits a mixed history, having initially immigrated into the plateau and thereafter undergoing in situ diversification, reflected in its unique molecular adaptations to both low temperature and high ammonia levels from geothermal springs (12). Our analyses indicate that the colonization of the modern Plateau Platform fauna began around 32 Ma (Fig. 3*B*), closely corresponding to the timing for flora colonization of the plateau (~29 Ma). This process was characterized by the high contribution of invertebrates and a marked absence of amphibians, likely reflecting the latter’s limited ability to tolerate the high and arid plateau environments (*SI Appendix*, Fig. S5) (9). Emigration out of the Plateau Platform and in situ diversification within the plateau occurred at very low levels and were only observed after the middle and late Miocene, respectively (Fig. 3 *A* and *C*). This pattern may reflect the relatively low geological heterogeneity and species richness of the plateau, which likely constrained both speciation and emigration. Alternatively, it may result from the harsh environmental conditions that only allow specialists to survive, adapt, or diversify.

The Hengduan Mountains underwent a profound environmental transformation during their Eocene uplift, shifting from a hot desert lowland to a moist montane forest comparable to the modern landscape by ~34 Ma, concurrent with the emergence of a distinctive bimodal monsoon system (Fig. 3*E*) (37, 66). Our analyses indicate that in situ diversification accelerated rapidly following the establishment of this modern-like environment and continued to rise until ~7 Ma (Fig. 3*A*). *Diploderma* lizards exhibit exceptional species diversity in the Hengduan Mountains, with phylogenetic and biogeographical evidence indicating that their diversification is closely associated with the formation of river valleys and related geological events (67). Invertebrates appear to be the most sensitive to these environmental changes, showing intensified diversification immediately after ~34 Ma, whereas other groups did not begin to diversify substantially until the Miocene (*SI Appendix*, Fig. S5). During this interval, river incision initiated in the early Eocene and progressively intensified, alongside monsoon strengthening and the development of the modern East Asian Monsoon, which became fully established between 13 to 9 Ma (Fig. 3*E*) (3, 36, 68). The initial Eocene uplift that created leveled uplands, followed by recurrent geological processes such as incision, exhumation, and rapid cooling, greatly enhanced the region’s geological heterogeneity, generating numerous isolated habitats that facilitated speciation and ultimately fueled the burst of in situ diversification (39, 40, 69–71). Notably, immigration and emigration also peaked during the interval of East Asian Monsoon intensification, when increased moisture likely facilitated dispersal by driving favorable shifts in vegetation and habitat connectivity that opened migration corridors to adjacent regions (Fig. 3 *B* and *C*). Strikingly, the peak of faunal immigration (16 to 5 Ma) and in situ diversification (7 Ma) coincides with the peak of floral immigration (15 to 13 Ma) and in situ diversification (7 Ma) (Fig. 3 *A* and *B*) (9), pointing to a shared biogeographic history in the assembly of both fauna and flora.

The present data support that the interplay between rapid geological uplift and monsoon intensification was likely a major driver of biotic assembly and diversification in the Himalaya. The Himalaya, the youngest of the three QTP subregions and the one primarily influenced by the South Asian Monsoon (Figs. 1*A* and 3*E*), began rising gradually in the middle Paleocene, reaching ~900 m by the early Eocene (at ca. 56 Ma) and ~2,100 m by the late Oligocene to early Miocene (24 to 21 Ma), before undergoing rapid uplift during the Miocene to surpass 5,000 m and approach elevations comparable to the present by the late Miocene to Pliocene (Fig. 3*E*) (3, 38, 72). Our study revealed a pronounced pulse in the accumulation of biogeographic events during this rapid uplift period (Fig. 3 *A*–*C*), suggesting that the emergence of numerous new ecological niches within a relatively short time likely promoted local speciation and colonization. Coelotine spiders, for example, underwent rapid radiation in the Himalaya driven by Eocene–Oligocene orogenesis, with localized vicariant events fostering speciation—a pattern reflecting both immigration (early dispersal blocked by uplift) and subsequent in situ diversification (16). Moreover, this rapid uplift coincided with a strong intensification of the South Asian Monsoon (73), with peaks of dispersal and in situ diversification nested within the interval of strongest monsoon influence (Fig. 3*A*). Among all fauna groups, birds show the most pronounced diversification patterns in the Himalaya, with peaks of in situ diversification and dispersal exceeding the other groups investigated. This result supports previous studies on specific bird clades that highlighted the Himalaya as a center of origin for many clades (*SI Appendix*, Fig. S5) (74–76). These results indicate that the interplay between rapid geological uplift and monsoon intensification was a major driver of biotic assembly and diversification in the Himalaya.

The distinct biogeographic roles of the three subregions can be further understood by considering their topographic configurations. The south-north oriented valleys of the Hengduan Mountains likely enhance north–south dispersal, contributing to the specific immigration/emigration patterns we observed (77). However, the Himalaya and the Plateau Platform also harbored ancient river systems that predate Himalayan uplift, allowing some degree of north–south biotic exchange (78, 79). This is supported by pronounced diversification patterns in Himalayan birds, which have served as a center of origin for many clades despite the east-west orientation of the range (74–76). Thus, while the physiography of the Hengduan Mountains may have quantitatively increased dispersal probabilities, the qualitative potential for north–south dispersal was not entirely absent in the other two regions.

The temporal patterns of in situ diversification and dispersal that shaped the faunal composition of the three QTP’s subregions, point to three major factors that make a mountain region a biodiversification center (i.e., a region that acts as a major source of lineages; not to be confounded with “biodiversity hotspot” that may be congruent, but is defined by high endemism, high threat, and also in high species richness (80): i) pronounced geomorphological heterogeneity driven by processes such as spatially differential uplift and erosion that creates a highly fragmented landscape and thus promoting population isolation and subsequent allopatric speciation; ii) the emergence of novel ecological niches and gradients following rapid topographic or climatic transitions provides ecological opportunities that facilitate adaptive radiation and ecological speciation; iii) the establishment of a humid and seasonally dynamic climate system—such as the monsoon strengthening—ensured tropical to subtropical conditions across the region, in contrast to the more arid environments prevailing in areas like the Hindu Kush, where such climatic forcing is absent. This sustained high speciation rates through enhanced habitat diversity, productivity, and ecological complexity over evolutionary timescales. On the contrary, a mountain region acting as a biodiversity sink (the Himalaya and Plateau Platform) could be characterized by i) environmental transitions that generate available or vacant niches that can attract colonization from surrounding regions; ii) the establishment of dispersal corridors from areas with high biodiversity. e.g., humid phases of monsoon intensification periodically transformed river valleys and forests into continuous dispersal corridors, enabling repeated immigration from adjacent regions.

In light of the evidence from the three QTP subregions, the first hypothesis introduced in this study is partially supported. In the Hengduan Mountains, earlier mid–late Eocene uplift coincides with deep phylogenetic node ages (~34 Ma) and sustained high in situ diversification, fully corroborating the prediction. In contrast, the Plateau Platform, despite equally early uplift and deep colonization ages (~32 Ma), exhibited low diversification and emigration, reflecting harsh post-uplift arid conditions that constrained biotic accumulation rather than promoting it. The later-uplifted Himalaya, however, showed young diversification and dispersal peaks during Miocene rapid uplift, consistent with the hypothesis. Thus, while earlier uplift generally corresponds to deeper divergence times, higher diversification is not an automatic consequence—it requires additional facilitating factors such as geological heterogeneity (the Hengduan Mountains) or monsoon-driven ecological opportunity (the Himalaya), whereas their absence (the Plateau Platform) suppresses diversification despite ancient uplift.

### Biogeographic Event Patterns in Light of Zoogeographical Divisions.

Our quantitative reconstruction of immigration and emigration events (*SI Appendix*, Fig. S2 and Table S17) allows us to examine whether the observed dispersal patterns align with the traditional zoogeographical division of the study region into the Palearctic and Oriental realms (81). The Himalaya exhibits a strong immigration bias from Southeast Asia (23 immigration events vs. 9 emigration events). This asymmetrical pattern is consistent with the Himalaya’s geographic position adjacent to the Oriental realm, which is dominated by tropical and subtropical fauna. The 23 immigration events from Southeast Asia suggest that the southern flank of the Himalaya has served as a major entry point for Oriental lineages colonizing the Tibetan Plateau region, while the relatively low outbound dispersal (9 events) indicates that the Himalaya function primarily as a “sink” for southern biota rather than a source for southward export. The Hengduan Mountains received the highest number of immigration events from south China (18 immigration events vs. 9 emigration events). Despite this asymmetry, the high frequency of these dispersal events still demonstrates the Hengduan Mountains’ unique position at the transition between Palearctic and Oriental realms. The Hengduan region appears to function as a biogeographical corridor that facilitates faunal exchange in both directions. This intense dispersal activity is likely enhanced by the region’s distinctive south-north oriented valleys, which provide dispersal pathways along the north–south axis as discussed above. The Plateau Platform shows the most limited exchange with adjacent regions. Its biota derives primarily from immigration from the Hengduan Mountains (9 events), with only rare outward dispersal (2 events to the Himalaya). This pattern suggests that the Plateau Platform, situated entirely within the Palearctic realm, has functioned as an “accumulation zone” rather than a dispersal hub, receiving lineages primarily from its southern neighbor (the Hengduan Mountains) rather than directly from the Oriental realm.

It should be noted, however, that these zoogeographical divisions are approximations rather than sharp boundaries. The transition between the Palearctic and Oriental realms in the Himalaya–Hengduan region is gradual, with extensive faunal mixing and overlap (81). Nevertheless, the observed patterns—strong southern immigration into the Himalaya, balanced exchange in the Hengduan corridor, and limited exchange on the Plateau Platform—are broadly consistent with expectations from zoogeographical frameworks.

### Predictors of Faunal Dispersal.

Contrary to the classic MacArthur–Wilson island biogeography theory (82, 83), our results show that smaller, more geographically isolated subregions within the Qinghai-Tibet Plateau (QTP) received proportionally more immigration events (Fig. 4). In the Hengduan Mountains and the Himalaya, such small and isolated areas often correspond to high-altitude peaks or relict habitats. Rather than acting as dispersal barriers, these areas function as climate refugia or marginal habitats that attract and/or sustain species from surrounding regions (34, 84). During glacial cycles, small isolated peaks retained stable microclimates, becoming regional refugia where species accumulated. Additionally, marginal habitats experience lower competition, facilitating successful colonization (34). Furthermore, total branch length (lineage accumulation time) is negatively correlated with immigration rate—geologically younger regions, notably the Himalaya, received more immigration. This pattern supports the unsaturated niche hypothesis: the Himalaya uplifted recently (Pliocene–Pleistocene) and their biota remains in an assembly phase (85, 86), whereas the Hengduan Mountains, with longer lineage accumulation, acts as a paleo-endemism center where saturated niches limit new immigration.

### Synchronous Evolution Between Fauna and Flora Evolution.

Returning to the second hypothesis proposed in the Introduction—that across the QTP, periods of major compositional shifts in flora should find parallel counterparts in faunal assembly, yet the timing and magnitude of these shifts may differ depending on key biological traits—the evidence presented here provides strong support with a specific refinement. Consistent with the hypothesis, the temporal dynamics of faunal evolution on the QTP closely parallel the patterns reported for its flora by Cao et al. (9), both at the scale of the entire plateau and within its three major subregions (Figs. 2*A* and 3 *A* and *B*). The peak timings of in situ diversification across the Plateau Platform, the Hengduan Mountains, and the Himalaya are nearly identical between animals and plants (Figs. 2*A* and 3*A*), suggesting that both groups responded in similar ways to major geoclimatic changes.

However, the hypothesis also suggested that differences in biological traits would modulate the timing and magnitude of shifts. This is corroborated by the observed disparity in immigration dynamics: peak immigration times of flora consistently precede those of fauna across all subregions (Figs. 2*A* and 3*B*). Therefore, we suggest that earlier plant establishment likely played an enabling role in subsequent faunal immigration. Plants, possessing higher dispersal potential, shorter generation times, and broader physiological tolerances, are likely to colonize newly formed habitats more rapidly, thereby acting as ecological pioneers that stabilized soils, structured vegetation, and generated primary productivity (87, 88). Consequently, the predicted “parallel counterparts” in assembly exist for in situ diversification, but a trait-dependent difference is evident in the consistent time lag between floral and faunal immigration, with plants preceding animals. Thus, the hypothesis is supported, with the qualification that the strongest signal of trait-mediated divergence lies in immigration timing rather than in the overall diversification peaks.

It is also noteworthy that individual animal groups exhibit differences in the timing and magnitude of diversification and dispersal events (*SI Appendix*, Fig. S5). Invertebrates, amphibians, reptiles, mammals, and birds each display distinct evolutionary trajectories, likely reflecting differences in dispersal ability, ecological requirements, and life-history traits. Among these, invertebrates stand out as an exception: the in situ diversification and immigration peaks tend to occur earlier than or partially overlapping with the onset of floral assembly, suggesting that certain invertebrate lineages may have colonized the QTP ahead of or concurrently with the establishment of vegetation. In contrast, birds and mammals conform more closely to the general pattern observed across most animal groups: their diversification and immigration events consistently postdate those of the flora, exhibiting a clear temporal lag relative to plant assembly. Notably, despite clade-specific differences in the timing of diversification and dispersal peaks, the onset of immigration in most animal groups—including amphibians, reptiles, birds, and mammals—consistently postdates that of the flora (Figs. 2*A* and 3 *A* and *B* and *SI Appendix*, Fig. S5). This concordant lag across diverse faunal lineages further supports the importance of early plant assembly, suggesting that the establishment of vegetation and associated habitats provided the ecological foundation that facilitated subsequent animal colonization and diversification across the QTP. The deviant pattern of invertebrates, however, raises intriguing questions about potential alternative drivers—such as pre-existing microhabitats or differing trophic roles—that may have enabled their earlier arrival.

## Conclusion

Integrating “mega-phylogenies” of vertebrates and invertebrates with updated paleoclimatic and tectonic evidence, we demonstrate that the QTP has functioned as both a major source of in situ diversification—particularly within the Hengduan Mountains and the Himalaya—and a key biogeographic sink, receiving substantial immigration from adjacent regions such as Southeast Asia and South China (Fig. 1 and *SI Appendix*, Fig. S2). Our results demonstrate that even across three closely situated subregions, their distinct uplift histories and monsoon intensification have driven asynchronous, yet interconnected, faunal evolutionary trajectories. Importantly, while earlier uplift generally corresponds to deeper divergence times, it does not translate into higher diversification rates, a finding that provides only partial support for the first hypothesis (Figs. 2 and 3). Notably, by comparing our faunal evolutionary dynamics with the flora pattern (9), we reveal an interesting and consistent “plants first, animals later” pattern across the QTP and its subregions. This cross-kingdom temporal time lag provides an independent line of evidence for a sequential assembly process, in which early-arriving plant lineages likely created the ecological conditions that facilitated subsequent faunal colonization, revealing an ordered and interdependent construction of this biodiversity hotspot. Together, our findings collectively highlight that bridging phylogenetic data across taxa with detailed geological history is essential to reconstruct how Earth’s dynamics built this iconic biodiversity hotspot.

## Materials and Methods

### Taxon Sampling.

We utilized five globally sampled, time-calibrated “mega-phylogenies” spanning amphibians, reptiles, birds, and mammals (Dataset S1). The amphibian dataset comprised a 765 species Caudata tree and a 5,242 species Anura tree both obtained directly from the original studies (89, 90). The reptile phylogeny included a 5,415 species Squamata tree, derived by modifying 10,000 raw trees of 9,574 species, with taxa lacking molecular data excluded (91). Although two recently published avian phylogenies provide broader taxon sampling (92, 93), we retained an older fully Bayesian time-calibrated phylogeny because our analyses require node-specific age uncertainty for downstream biogeographic inference, which is not consistently available in these large-scale trees. We therefore used the avian dataset consisting of a 6,670-species phylogeny reconstructed from 10,000 genetic-only trees and anchored to the Hackett backbone (94). The mammalian dataset included a 4,098 species tree obtained directly from the original study (95). To ensure sufficient representation of the QTP region’s species and to include key invertebrate groups lacking comprehensive phylogenies, we reconstructed additional time-calibrated molecular trees for four invertebrate (370 species), six reptile (380 species), and five mammalian (285 species) clades using multigene sequences from GenBank (*SI* *Appendix*, Tables S1–S15).

### Phylogenetic Analysis and Divergence Time Estimation.

For each newly reconstructed clade, we assembled multigene datasets from GenBank based on previous phylogenetic studies, incorporating mitochondrial and nuclear markers where available (*SI Appendix*, Tables S1–S15). Depending on taxonomic group and data availability, datasets included 2 to 21 mitochondrial and nuclear markers (*SI Appendix*, Tables S1–S15). We aligned sequences and assembled concatenated dataset in Geneious Prime 2025.0.2 (https://www.geneious.com). We performed phylogenetic reconstruction and divergence time estimation in BEAST v2.6.6 under an uncorrelated relaxed molecular clock model with a Yule tree prior (96). We selected the best-fitting nucleotide substitution model for each clade using ModelFinder2 under the Bayesian Information Criterion, as implemented in PhyloSuite (97, 98). Divergence times were calibrated using multiple fossil constraints and/or well-established secondary calibration points derived from previous phylogenetic studies of the focal groups (Dataset S1). We ran BEAST analyses for 50 million generations, with parameters and trees sampled every 5,000 generations. We assessed convergence and sampling adequacy by examining effective sample sizes (ESS) in Tracer v1.6, all of which exceeded 200, indicating proper mixing of the Markov chains (99). We generated maximum clade credibility trees using TreeAnnotator v2.6.6, discarding the first 10% of sampled trees as burn-in (Dataset S2).

### Identification of QTP Endemic Species and Ancestral Distribution Reconstruction.

We delineated eight biogeographic regions within and adjacent to the QTP region by integrating QTP region boundaries (1) with established terrestrial ecoregions (100) including: the Plateau Platform (TP), Hengduan Mountains (HDM), Himalaya (HIM), Central and North Asia (CNA), East Asia (EA), South China (SC), Southeast Asia (SEA), and South Asia (SA) (*SI* *Appendix*, Fig. S1). All other terrestrial areas were classified as a single “Remaining World” (RW) unit. We assigned each species in the phylogenies to one or more of these regions based on its natural distribution. We define a species as “QTP-endemic” if its entire known global distribution falls within the QTP boundary. We obtained distribution data from the following databases: the IUCN Red List (https://www.iucnredlist.org/), the AmphibiaChina database (https://amphibiachina.org/) for amphibians, the IUCN Red List and the Reptile Database (http://www.reptile-database.org/) for reptiles, the IUCN Red List for mammals, and the BirdLife Data Zone (https://datazone.birdlife.org/) for birds (using breeding ranges only). For invertebrates, biogeographic assignments relied on the sources referenced in their original phylogenetic reconstructions (Dataset S1). For taxa absent from these databases, we determined distributions based on original species descriptions and type localities.

Ancestral range estimations were performed in BioGeoBEARS using both the dispersal–extinction–cladogenesis (DEC) model and its founder-event speciation extension (DEC + J) (101, 102). All analyses were based on the time-calibrated phylogenetic trees generated before. The maximum range size of terminal taxa was set to the largest number of regions occupied by any single species in the dataset. Collectively, the outputs from all previous analyses, including the dated phylogenies and the reconstructed biogeographic events, formed the basis for our subsequent biogeographical meta-analysis.

### Biogeographical Meta-Analysis.

We performed a meta-analysis of dated divergence events to examine the temporal dynamics of in situ diversification and dispersal events. These dynamics were quantified using two metrics: the maximum number of in situ diversification events per million years (MID) and the maximum number of dispersal events per million years (MDE) (103, 104). This approach rests on the premise that biogeographic events are driven by common abiotic factors and thus may occur synchronously across lineages, consistent with the concept of geodispersal (105).

For each clade, we extract the 95% CI for in situ diversification and dispersal events from the time-calibrated phylogenies. We calculated MID and MDE within 1-million-year (1 Ma) time bins and applied a sliding-window smoothing procedure with a 5 Ma window to reduce stochastic variation. The meta-analysis was conducted on multiple levels: 1) a regional level, considering the QTP as a whole; 2) a subregional level, analyzing the Tibetan Plateau, the Hengduan Mountains, and the Himalaya separately; 3) a taxonomic level, analyzing each of the five sampled groups independently.

To assess the robustness of the observed MDE and MID patterns, we performed a bootstrap resampling test. For regional and subregional level, 100 bootstrap replicates were generated by resampling (without replacement) the 95% credibility intervals of dated biogeographic events (dispersal or in situ diversification). The temporal distribution of events in each replicate was then used to generate bootstrap MDE and MID curves (*SI* *Appendix*, Fig. S4). Finally, the peaks and temporal patterns of the bootstrap distributions were compared with the original MDE and MID estimates to evaluate the consistency and support for the inferred biogeographical signals.

To test if observed diversification and dispersal dynamics arose from lineage accumulation or temporal structure of the phylogenies, we performed a randomization test (104). For each clade with QTP-associated events, we randomly sampled internal nodes matching the number of observed biogeographic events (dispersal or in situ diversification), treated them as pseudoevents and recalculated MID and MDE curves. This was repeated 100 times per clade to generate null distributions for the QTP and its three subregions. The resulting randomized curves were compared with the empirical MID and MDE curves. If the empirical temporal patterns differ substantially from those generated under the randomization procedure, this indicates that the observed diversification and dispersal dynamics are unlikely to be explained solely by phylogenetic structure or lineage accumulation, and instead reflect nonrandom biogeographic processes.

### Predictors of Dispersal Events.

Following Antonelli et al. (106), we tested whether simple geographic and phylogenetic descriptors predict dispersal events (immigration/emigration) across region. Response variables were inferred immigration/emigration counts (*SI* *Appendix*, Tables S20 and S21) per clade and region. Predictors included a region’s present-day area, perimeter (*SI Appendix*, Fig. S8), geographic isolation (median distance from centroid to others) (106), and total branch length [time lineages occupied a region, weighted by marginal probabilities from DEC/DEC + J reconstructions (101, 102)].

We log-transformed responses and predictors (replacing the few zero counts with one to allow transformation), standardized predictors, screened the predictors for collinearity using variance inflation factors [R package “car”; (107)] and pairwise Pearson correlations (*SI Appendix*, Tables S23 and S24). Perimeter was removed due to strong correlation with area and geographic isolation. Separately for emigration and immigration, we fitted Bayesian linear mixed-effects model with three predictors as fixed effects and taxon as a random intercept, to accommodate nonindependence among the five animal groups, using the integrated nested Laplace approximation implemented in R-INLA (108). We assigned log-gamma priors (shape 1, scale 0.5) to the precision of the random intercept and of the Gaussian likelihood. A predictor was treated as having an important effect when its 95% credible interval (CrI) excluded zero. Partial-fit plots (*SI Appendix*, Fig. S8 *C*–*H*) show posterior mean predictions.

We repeated the analysis under two regionalizations: main scheme splitting the QTP into Plateau Platform, the Himalaya, and the Hengduan Mountains (Fig. 4); and sensitivity scheme treating the QTP as one unit (*SI Appendix*, Fig. S8).

## Supplementary Material

Appendix 01 (PDF)

Dataset S01 (PDF)

Dataset S02 (PDF)

## Data Availability

Study data are included in the article and/or supporting information.
