# Supplementary material for: Sources and sinks: Geoclimatic dynamics shaped the assembly of endemic fauna in the world’s Third Pole
Source: Proc Natl Acad Sci U S A. 2026 Oct 26;123(44):e2602323123. doi: 10.1073/pnas.2602323123 (PMC13732247; doi:10.1073/pnas.2602323123)
Supplement: Supplementary file 1 [file pnas.2602323123.sapp.pdf]

PNAS

Supporting Information for

Sources and Sinks: Geo-climatic Dynamics Shaped the Assembly of Endemic Fauna in the world's Third Pole.

Dechun Jiang[#], Mao-Liang Li[#], Jin-Long Ren, Rui-Han Li, Zhi-Tong Lyu, Sebastian Klaus, Ru-Wan Jia, Tao Xue, Jia-Tang Li[*]

* Corresponding Author: Jia-Tang Li
Email: lijt@cib.ac.cn

[#]D.J. and M.-L.L. contributed equally to this work.

This PDF file includes:

Supporting text
Figures S1 to S8
Tables S1 to S24
Legends for Datasets S1 to S2
SI References

Other supporting materials for this manuscript include the following:

Datasets S1 to S2

Supporting Information Text

Calibration points used in dated phylogeny reconstruction

Invertebrates.

***Anthocoris* (1 genus, 11 species)**
One fossil calibration point was used: the age of a fossil species *Temnostethus blandus* that assigned to 23–35 Ma was used as the time of the splitting of *Temnostethus* and *Anthocoris*, set as lognormal distribution with offset = 23, mean = 1, and standard deviation = 0.90 (1).

Coelotinae (22 genera, 286 species)
Ten recalibration points were used: (a) the crown age of Agelenidae set as normal distribution with mean = 78.0 and standard deviation = 7.0; (b) the crown age of Coelotinae set as normal distribution with mean = 54.0 and standard deviation = 6.0; (c) the crown age of *Tegenaria* set as normal distribution with mean = 44.0 and standard deviation = 5.0; (d) the crown age of *Agelena* set as normal distribution with mean = 18.0 and standard deviation = 3.5; (e) the crown age of *Pireneitega* set as normal distribution with mean = 25.5 and standard deviation = 4.0; (f) the split of *Tegecoelotes* and *Coelotes* set as normal distribution with mean = 22.5 and standard deviation = 4.0; (g) the split of *Draconarius* and *Himalcoelotes* set as normal distribution with mean = 27.5 and standard deviation = 4.5; (h) the crown age of *Lineacoelotes* set as normal distribution with mean = 16.5 and standard deviation = 2.5; (i) the crown age of *Platocoelotes* set as normal distribution with mean = 16.0 and standard deviation = 2.5; (j) the crown age of Coras set as normal distribution with mean = 7.5 and standard deviation = 2.0 (2).

***Cryptocercus* (1 genus, 31 species)**
Two secondary calibration points were used: (a) the inferred divergence between *Cryptocercus* and termites (145.2 Ma, 95% CI 124.7–172.6 Ma), set as normal distribution with mean = 145.2 and standard deviation = 16; (b) the inferred crown age of *Cryptocercus* (74.8 Ma, 95% CI: 51.2–103.8 Ma), set as normal distribution with mean = 74.8 and standard deviation = 15 (3).

***Gnaptorina* sensu lato and *Agnaptoria* (2 genera, 42 species)**
Two secondary calibration points were used: (a) the crown age of *Gnaptorina* (55.9 Ma), set as normal distribution with mean = 55.9 and standard deviation = 5.5; (b) the crown age of *Hesperoptorina* (48.7 Ma), set as normal distribution with mean = 48.7 and standard deviation = 5 (4).

Reptiles

***Lycodon* (1 genus, 48 species)**
Two secondary calibration points was used: (a) the crown age of *Lycodon* (18.11 Ma, 95% CI: 14.06–25.14 Ma), set as normal distribution with mean = 18.11 and standard deviation = 3[5]; (b) the split between *Bioga* and *Lycodon* (22.21 Ma, 95% CI: 17.35–29.7 Ma), set as normal distribution with mean = 22.21 and standard deviation = 3.5 (5).

Natricidae (36 genera, 205 species)
One secondary calibration points was used: the crown age of Natricidae (39.02 Ma), set as normal distribution with mean = 18.49 and standard deviation = 2 (6).

Pareidae (4 genera, 39 species)
Four secondary calibration points were used: (a) split between *Xylophis* and Pareinae (44.9 Ma, 95% CI: 38.9–51.2 Ma), set as normal distribution with mean = 44.9 and standard deviation = 3.8; (b) split between *Asthenodipsas* and (*Aplopeltura* + *Pareas*) (38.0 Ma, 95% CI: 31.1 – 44.6 Ma), set as normal distribution with mean = 38 and standard deviation = 4; (c) split between *Aplopeltura* and *Pareas* (28.4 Ma, 95% CI: 21.7 – 35.7 Ma), set as normal distribution with mean = 28.4 and standard deviation = 4.5; (d) Split between *Xylophis captaini* and *X. perroteti* (32.5 Ma,

95% CI: 24.4 – 40.8 Ma), set as normal distribution with mean = 32.5 and standard deviation = 5 (7).

***Gloydius* (1 genus, 26 species)**
Two secondary calibration points were used: (a) the stem age of *Gloydius* (23.76 Ma), set as normal distribution with mean = 23.76 and standard deviation = 3; (b) the split between *Trimeresurus gracilis* and *Ovophis okinavensis* (11.06 Ma), set as normal distribution with mean = 11.06 and standard deviation = 3 (8).

***Diploderma* (1 genus, 39 species)**
One secondary calibration points was used: the split between *Pseudocalotes* and *Diploderma* (32.64 Ma, 95% CI: 23.7–41.01 Ma), set as normal distribution with mean = 32.64 and standard deviation = 5.5 (5).

***Scincella* (1 genus, 23 species)**
Two secondary calibration points were used: (a) the crown age of *Scincella* (37.56 Ma, 95% CI: 29.48–45.77 Ma), set as normal distribution with mean = 37.56 and standard deviation = 5; (b) the most common ancestor between *Scincella* and *Sphenomorphus* (40.54 Ma, 95% CI: 33.48–50.27 Ma), set as normal distribution with mean = 40.54 and standard deviation = 5.5 (5).

Mammals

***Ochotona* (1 genus, 34 species)**
Two secondary calibration points were used: (a) the most common ancestor between *Ochotona* and *Oryctolagus* (51.86 Ma, 95% CI: 47.37–56.84 Ma), set as normal distribution with mean = 51.86 and standard deviation = 3; (b) the crown age of Ochotona (13.75 Ma, 95% CI: 10.97–20.59 Ma), set as normal distribution with mean = 13.75 and standard deviation = 2.5 (9).

Spalacidae (7 genera, 23 species)
One secondary calibration points was used: the crown age of Spalacidae (25.11 Ma, 95% CI: 21.19–28.81 Ma), set as normal distribution with mean = 25.11 and standard deviation = 2.2 (10).

***Niviventer* (1 genus, 22 species)**
Two secondary calibration points were used: (a) the crown age of *Niviventer* (8.23 Ma, 95% CI: 6.95–9.69 Ma) set as normal distribution with mean = 8.23 and standard deviation = 1 (b) the split between *Niviventer* and *Chiromyscus* (9.47 Ma, 95% CI: 8.08–11 Ma), set as normal distribution with mean = 9.47 and standard deviation = 1 (11).

Talpidae (18 genera, 54 species)
Two secondary calibration points were used: (a) the crown age of Talpidae (39.81 Ma, 95% CI: 33.40–46.94 Ma), set as normal distribution with mean = 39.81 and standard deviation = 4; (b) the stem age of *Scalopus* + *Parascalops* + *Alpiscaptulus* + *Scapanulus* (29.64 Ma, 95% CI: 24.82–35.26 Ma), set as normal distribution with mean = 29.64 and standard deviation = 3.4 (11).

Arvicolinae (30 genera, 152 species)
Two secondary calibration points were used: (a) the crown age of Arvicolinae (14.43 Ma, 95% CI: 12.62–16.44 Ma), set as normal distribution with mean = 14.43 and standard deviation = 1.2; (b) the crown age of *Microtus* (8.11 Ma, 95% CI: 7.06–9.27 Ma), set as normal distribution with mean = 8.11 and standard deviation = 0.6 (11).

Figures

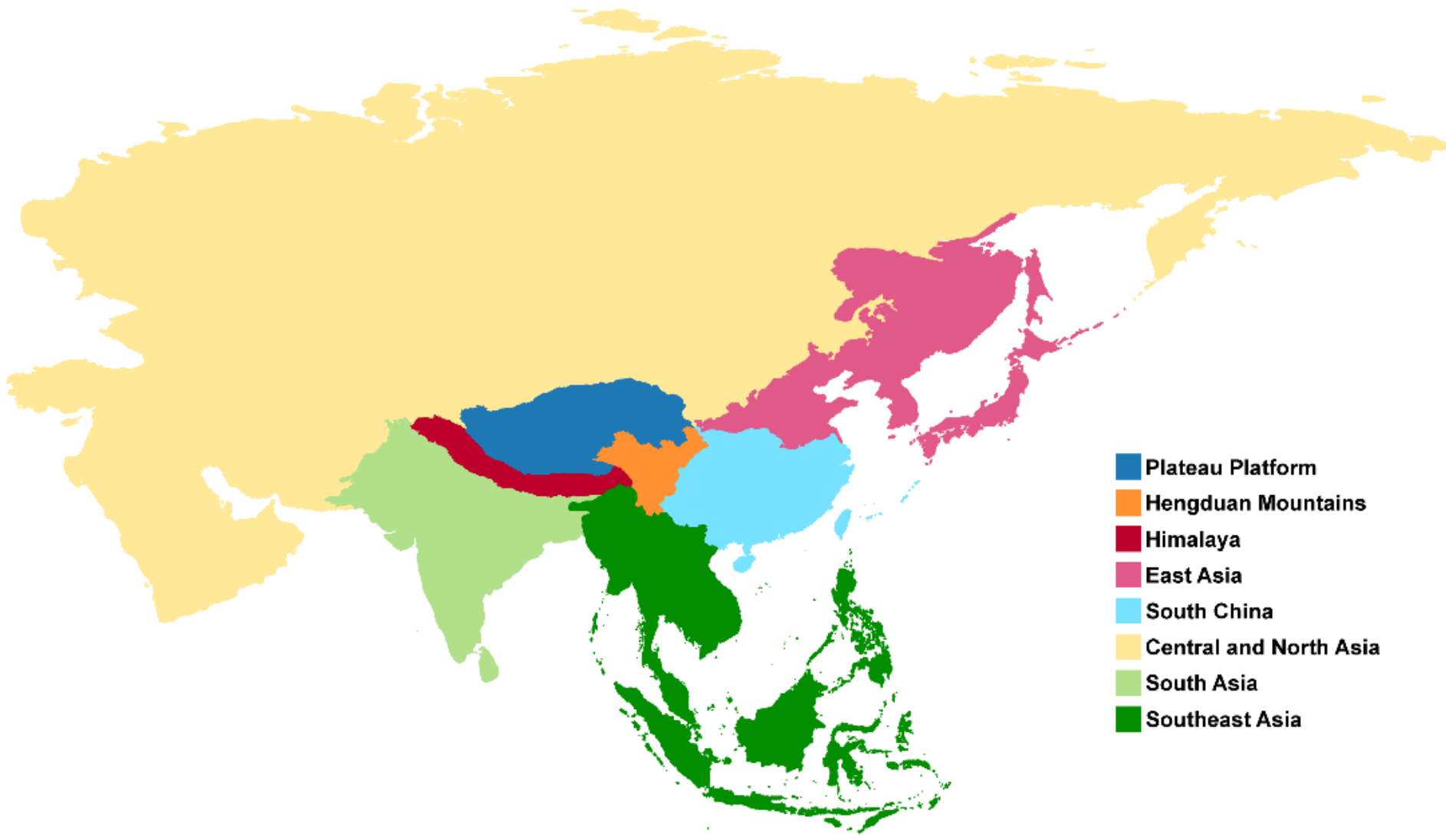

Fig. S1. Biogeographic delineations of Qinghai-Tibet Plateau and adjacent regions.

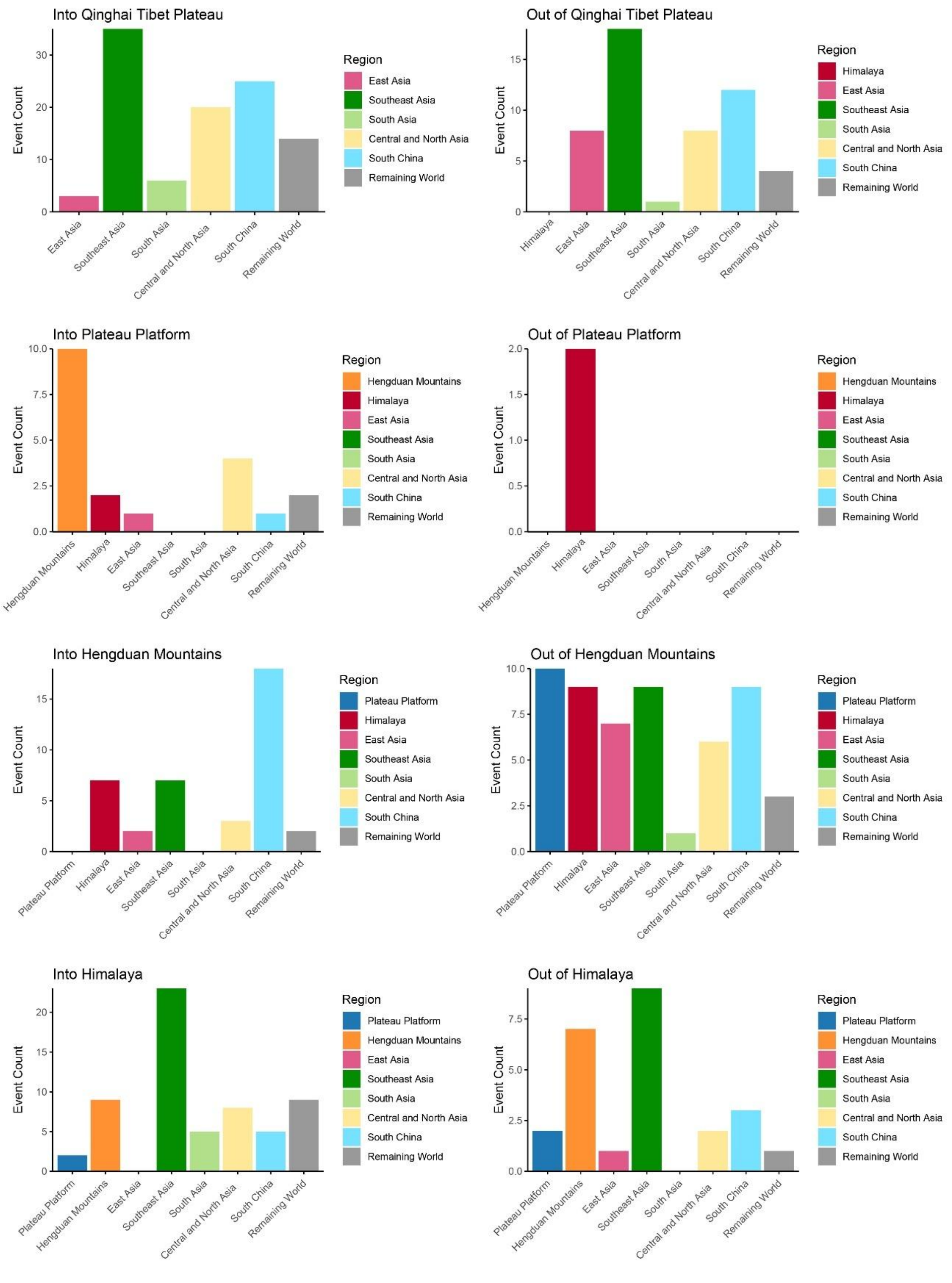

Fig. S2. Composition of immigration and emigration events for the Qinghai-Tibet Plateau and its three subregions.

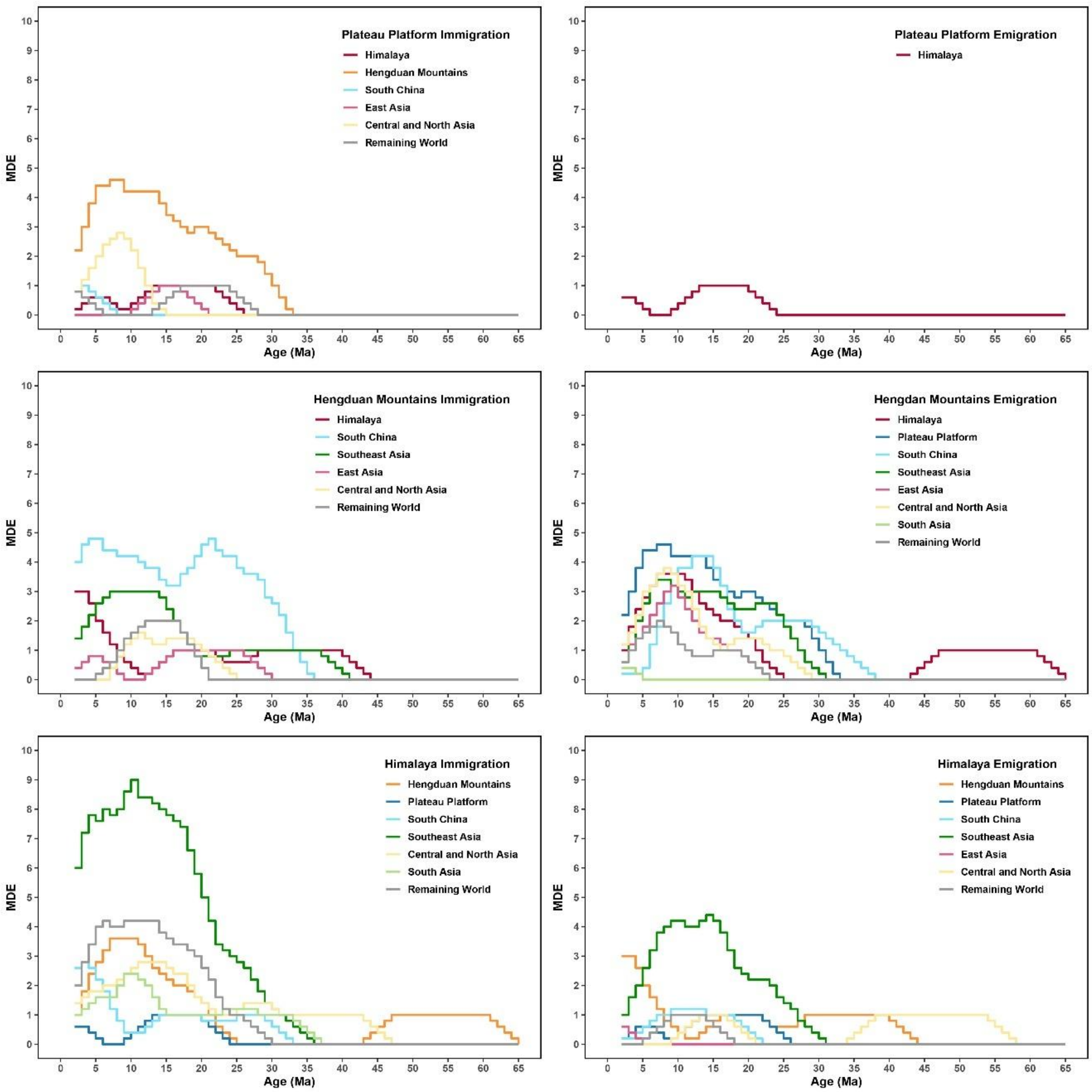

Fig. S3. MDE curves of immigration and emigration of Qinghai-Tibet Plateau's three subregions since 65 Ma with each direction represented by a single curve.

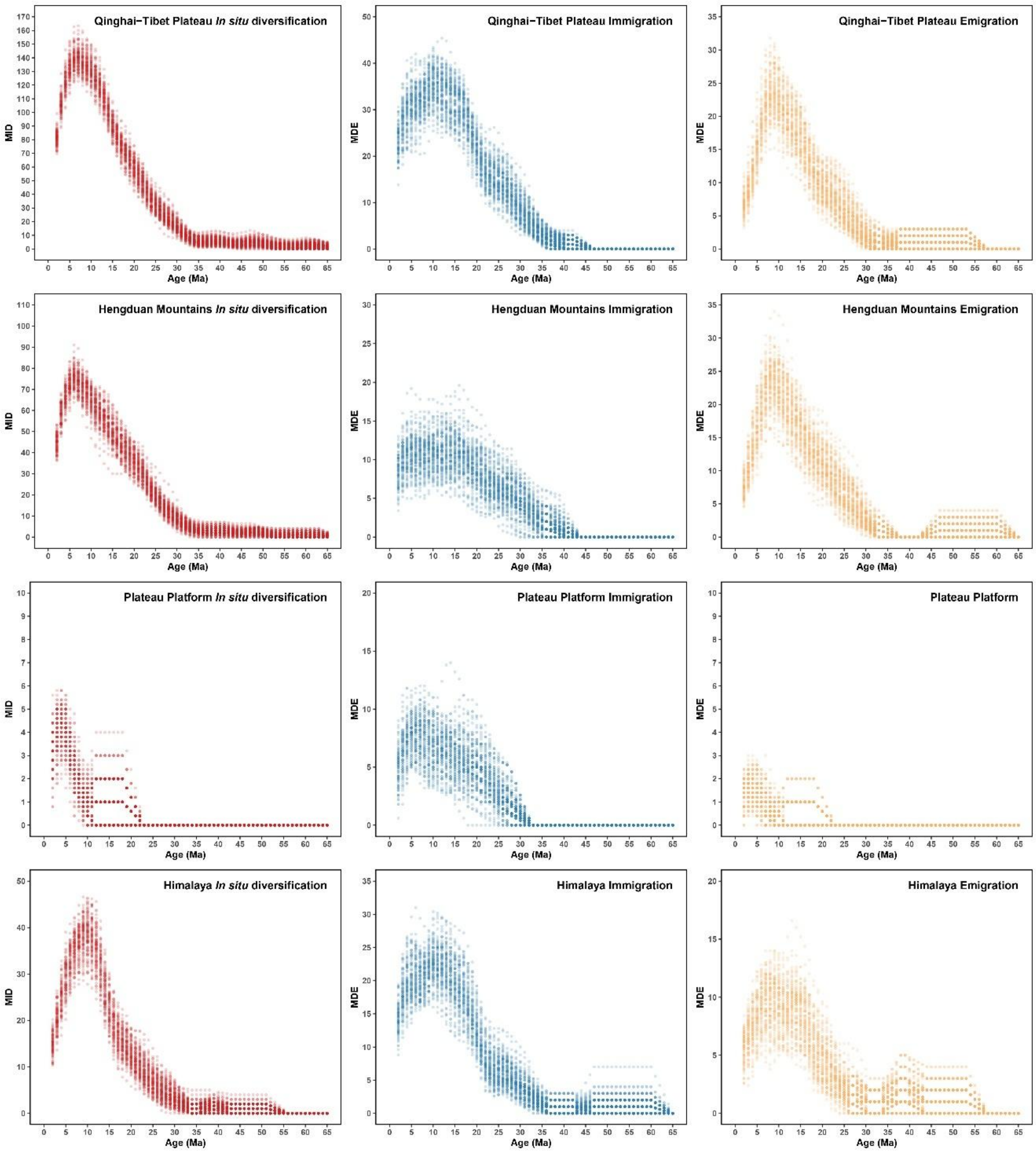

Fig. S4. Bootstrap analyses of biogeographic dynamics of Qinghai-Tibet Plateau and its three subregions since 65 Ma based on 100 replicates.

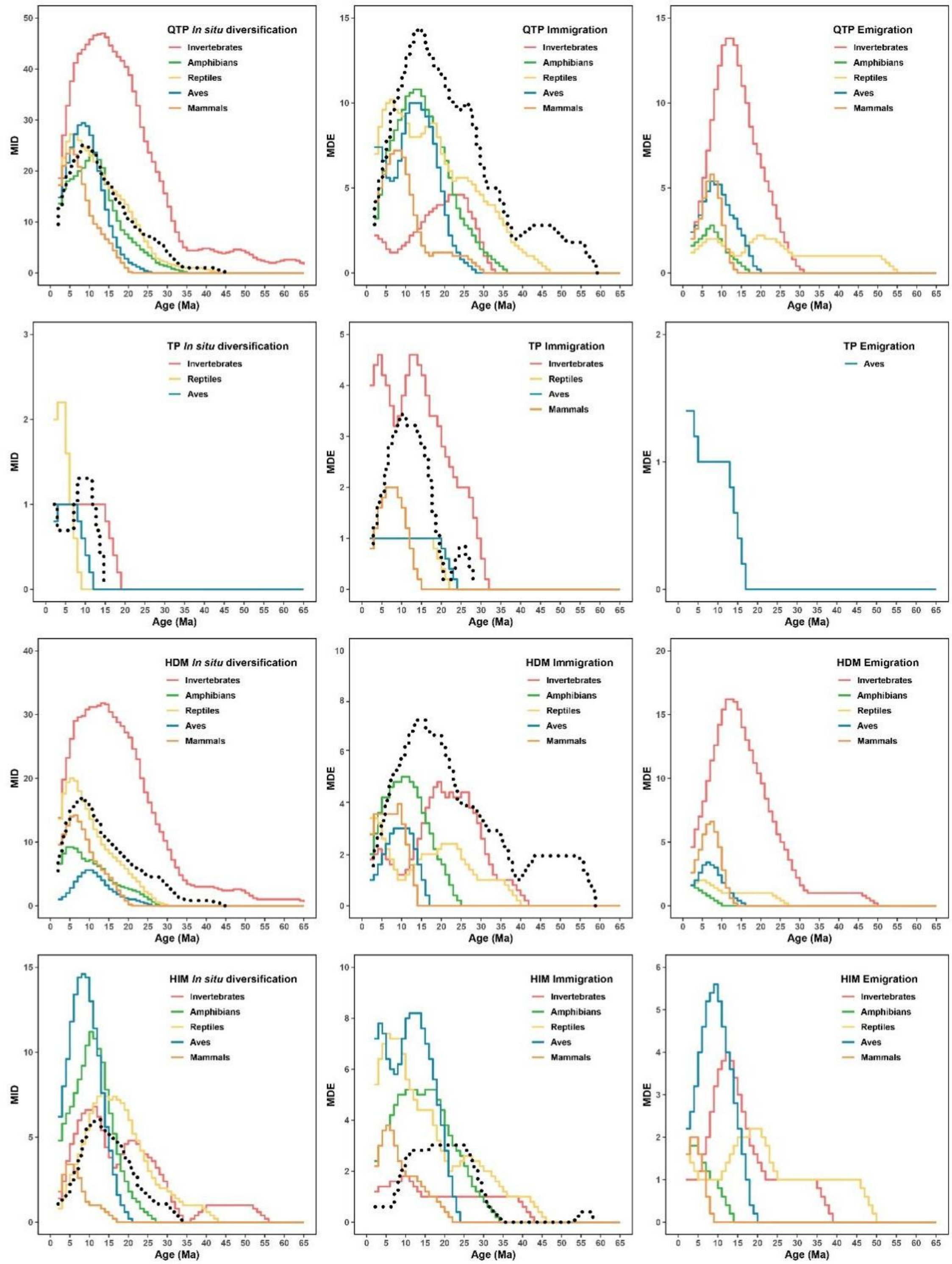

Fig. S5. MID (the maximum number of in situ diversification events per million years) and MDE (the maximum number of dispersal events per million years) dynamics for five fauna groups in the Qinghai-Tibet Plateau (QTP) and its three subregions since 65 Ma. MID and MDE curves of the endemic flora for the QTP and its subregions (modified from Cao et al., 9) are shown as black dotted lines.

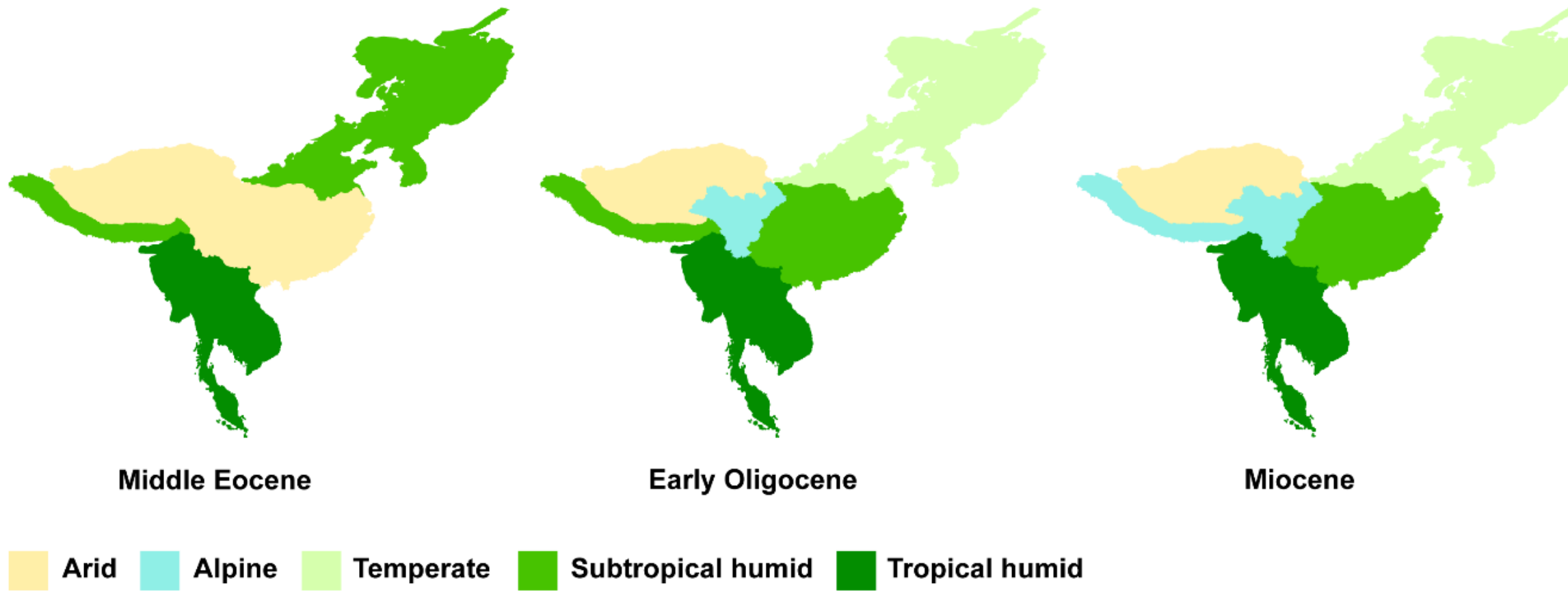

Fig. S6. Main climatic features of the Qinghai-Tibet Plateau and Adjacent Regions based on the summary of Table S19.

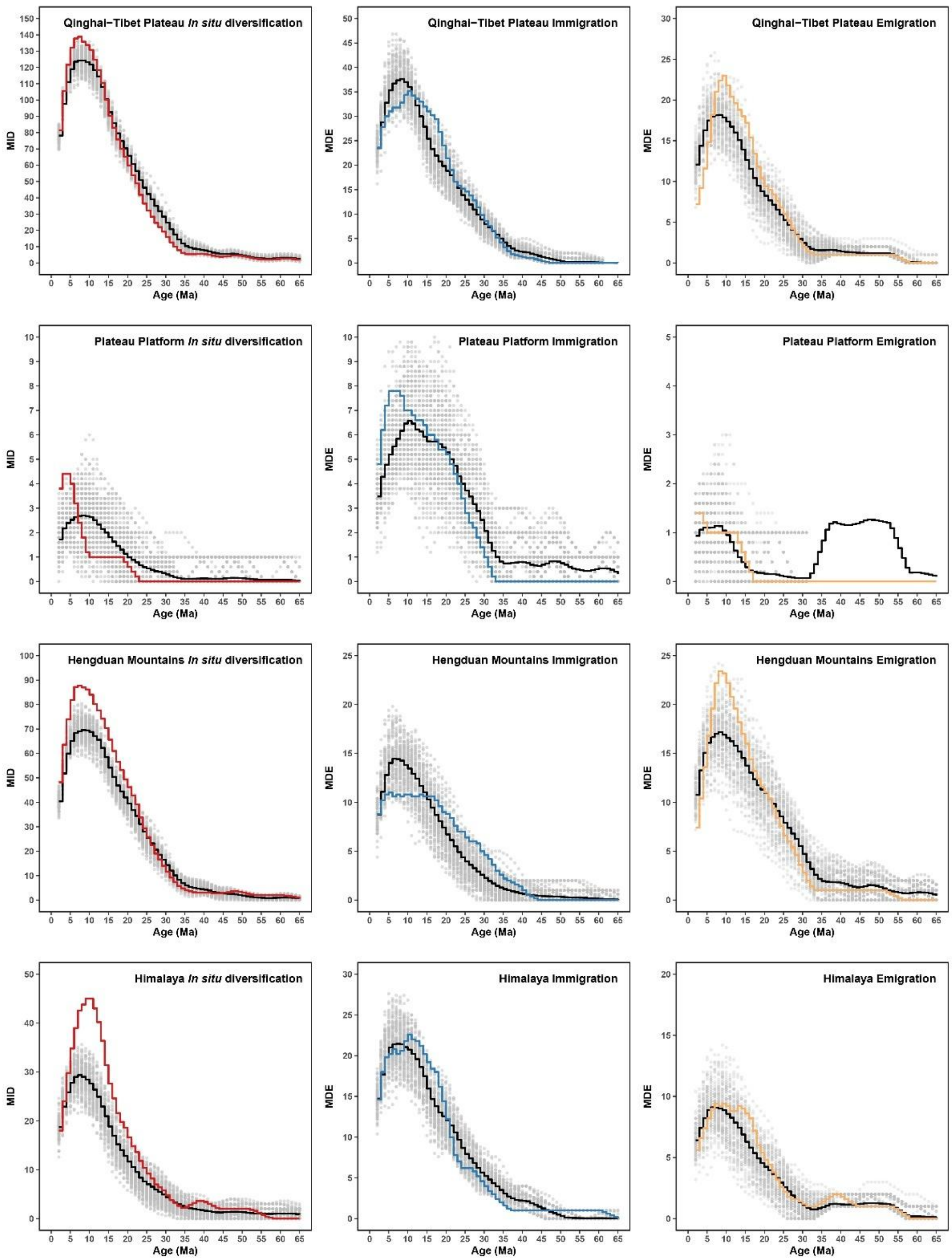

Fig. S7. Random MID and MDE dynamics of Qinghai-Tibet Plateau and its three subregions since 65 Ma. Black lines representing the mean and gray dots indicating individual values from 100 replications shown in comparison with MID and MDE dynamics based on inferred biogeographic events (colored lines).

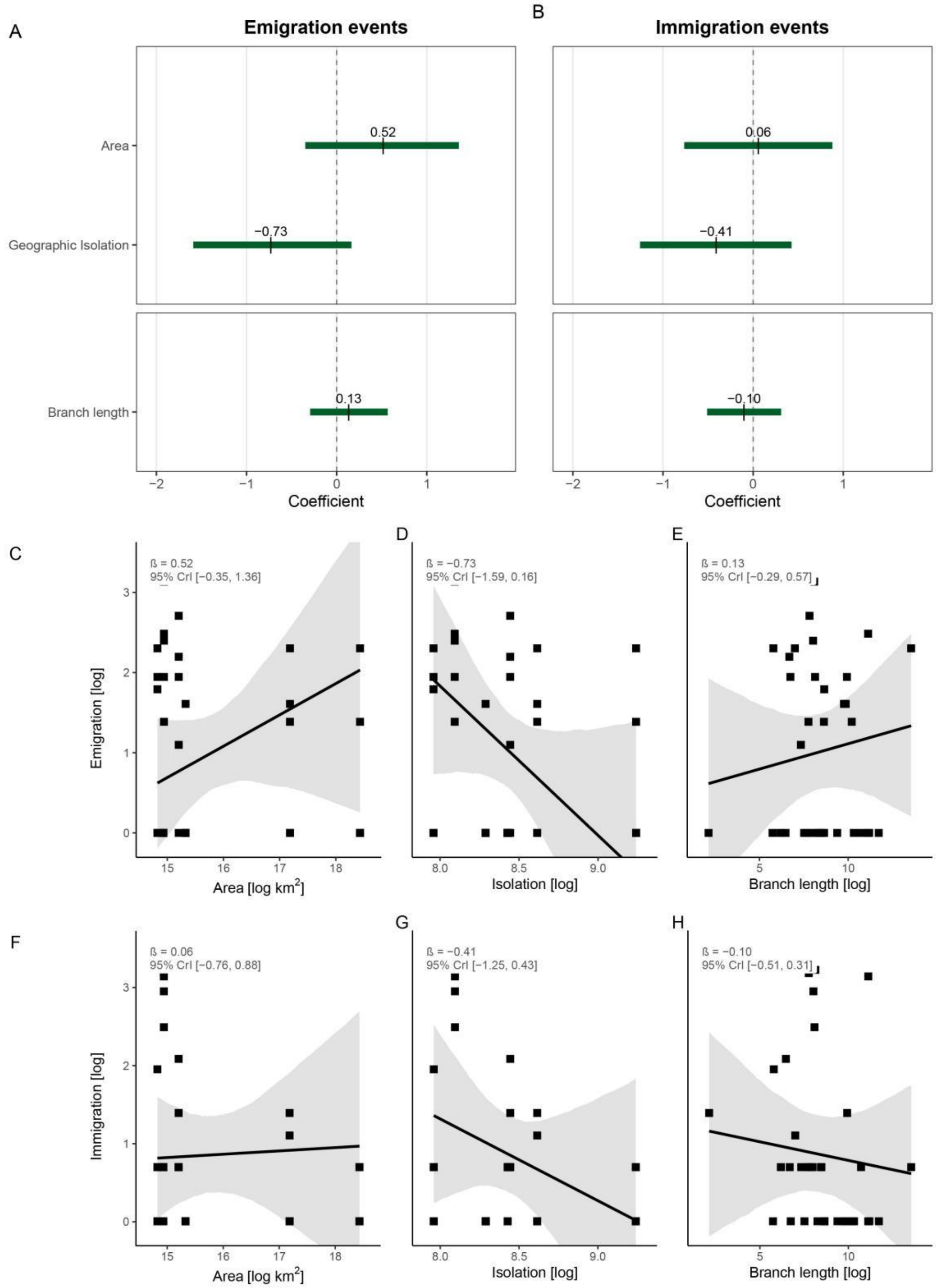

Fig. S8. Relationship between the number of dispersal events out of (emigration) and into (immigration) a region and predictor variables across the QTP (Scheme 1). (A and B) Regression coefficients of a Bayesian multiple linear mixed-effects model calculated based on all regions and clades for emigration and immigration events, respectively. (C–E) Partial fit of emigration events against area, geographic isolation, and branch length, respectively; (F–H) Partial fit of immigration events against the same three predictor variables. Fitted lines show the posterior mean predicted values from the same model, assuming average values for all other predictors, whereas the

points show the raw data. Shaded areas show 95% credible intervals. All variables are log-transformed.

Tables

Table S1. Species and GenBank accession numbers used for phylogeny reconstruction of *Anthocoris* (Insecta, Hemiptera, Anthocoridae).

Species	16S rRNA	COI
Anthocoris montanus	FJ914507	FJ917680
Anthocoris zoui	FJ914533	FJ917706
Anthocoris pericarti	GQ415595	GQ415603
Anthocoris expansus	GQ415591	GQ415599
Anthocoris kerzhneri	GQ415592	GQ415600
Anthocoris antevolens	GQ415590	GQ415598
Anthocoris nemorum	GQ415594	GQ415602
Anthocoris alpinus	GQ415589	GQ415597
Anthocoris qinlingensis	GQ415596	GQ415604
Anthocoris limbatus	GQ415593	GQ415601
Anthocoris miyamotoi	GU296664	GU296668
Orius sauteri	GU296665	GU296669
Temnostethus reduvinus	GU296666	GU296667

Table S2. Species and GenBank accession numbers used for phylogeny reconstruction of Coelotinae (Arachnida, Araneae, Agelenidae).

Species	12S	16S	18S	28S	COI	H3	ND1	wingless
Agelena_koreana		JN816567	JN816783	JN816993	JN817201			
Agelena_labyrinthica		AY633873	AY633862	FR694016	FN554797		FN678908	
Agelena_orientalis				FR694024			FN678909	
Agelena_silvatica		JN816564		JN816990				
Agelenopsis_aperta		FJ607444	FJ607478	FJ607517	FJ607552	FJ607591		FJ607629
Agelenopsis_pennsylvanica		AY770768		DQ628593	AY770799			
Aterigena_aculeata				FR694018	FN554790		FN678912	
Aterigena_ligurica				FR694031	FN554789		FN678913	
Barronopsis_barrowsi		DQ628581	DQ628737, DQ628700	DQ628664	DQ628609	DQ628636		
Barronopsis_sp		AY770748			AY770810			
Bifidocoelotes_sp	KY791815	KY791516	KY791215	KY790917	KY778803	KY779060	KY790630	KY779357
Calilena_californica		DQ628570		DQ628590	DQ628548			
Calilena_restricta		DQ628567		DQ628587	DQ628545			
Coelotes_acicularis1	KY792076	KY791777	KY791476	KY791174	KX555516	KY779321	KY790878	KY779582
Coelotes_acicularis2	KY792077	KY791778	KY791477	KY791175	KY779025	KY779322	KY790879	KY779583
Coelotes_antri	KY792054	KY791755	KY791454	KY791152	KY779012	KY779299	KY790858	KY779567
Coelotes_exitialis	KY792055	KY791756	KY791455	KY791153	KY779013	KY779300	KY790859	KY779568
Coelotes_forficatus	KY792078	KY791779	KY791478	KY791176	KX555512	KY779323	KY790880	KY779584

Coelotes_guangxian	KY792079	KY791780	KY791479	KY791177	KX555515	KY779324	KY790881	KY779585
Coelotes_icohamatus	KY791896	KY791597	KY791296	KY790994	KY778884	KY779141	KY790704	KY779422
Coelotes_kitazawai	KY792056	KY791757	KY791456	KY791154	KY779014	KY779301	KY790860	
Coelotes_lamellatus	KY792058	KY791759	KY791458	KY791156	KY779016	KY779303		
Coelotes_microps	KY791997	KY791698	KY791397	KY791095	KY778965	KY779242		KY779512
Coelotes_microps_XN	KY792045	KY791746	KY791445	KY791143	KY779003	KY779290	KY790849	KY779559
Coelotes_musashiensis	KY792057	KY791758	KY791457	KY791155	KY779015	KY779302	KY790861	
Coelotes_pickardi_tirolensis	KY792041	KY791742	KY791441	KY791139	KY778999	KY779286	KY790846	
Coelotes_plancyi	KY791839	KY791540	KY791239	KY790940	KY778827	KY779084	KY790650	KY779375
Coelotes_pseudoterrestris_DL	KY792065	KY791766	KY791465	KY791163	KX555513	KY779310	KY790868	KY779572
Coelotes_pseudoterrestris_KM1	KY792066	KY791767	KY791466	KY791164	KY779023	KY779311	KY790869	KY779573
Coelotes_pseudoterrestris_KM2	KY792067	KY791768	KY791467	KY791165	KX555518	KY779312	KY790870	KY779574
Coelotes_pseudoterrestris_TC	KY792068	KY791769	KY791468	KY791166	KX555511	KY779313		KY779575
Coelotes_pseudoyunnanensis_DL	KY792069	KY791770	KY791469	KY791167	KY779024	KY779314	KY790871	KY779576
Coelotes_pseudoyunnanensis_YK	KY792070	KY791771	KY791470	KY791168	KX555519	KY779315	KY790872	KY779577
Coelotes_satoi	KY792059	KY791760	KY791459	KY791157	KY779017	KY779304	KY790862	
Coelotes_solitarius_RO	KY792040	KY791741	KY791440	KY791138	KY778998	KY779285	KY790845	KY779555
Coelotes_sp1	KY792063	KY791764	KY791463	KY791161	KY779021	KY779308	KY790866	

Coelotes_sp10	KY791860	KY791781	KY791480	KY791178	KX555517	KY779325	KY790882	KY779586
Coelotes_sp11	KY791866	KY791782	KY791481	KY791179	KY779026	KY779326	KY790883	KY779587
Coelotes_sp12	KY792031	KY791783	KY791482	KY791180	KX555520	KY779327	KY790884	KY779588
Coelotes_sp13	KY792034	KY791784	KY791483	KY791181	KX555506	KY779328	KY790885	KY779589
Coelotes_sp2	KY792064	KY791765	KY791464	KY791162	KY779022	KY779309	KY790867	
Coelotes_sp3	KY792073	KY791774	KY791473	KY791171	KX555508	KY779318	KY790875	KY779580
Coelotes_sp4	KY792074	KY791775	KY791474	KY791172	KX555509	KY779319	KY790876	KY779581
Coelotes_sp5	KY792075	KY791776	KY791475	KY791173	KX555510	KY779320	KY790877	
Coelotes_sp6	KY792046	KY791747	KY791446	KY791144	KY779004	KY779291	KY790850	KY779560
Coelotes_sp7		KY791786	KY791485	KY791183	KY779028	KY779330	KY790887	KY779591
Coelotes_sp8		KY791787	KY791486	KY791184	KY779029	KY779331	KY790888	KY779592
Coelotes_sp9	KY792071	KY791772	KY791471	KY791169	KX555514	KY779316	KY790873	KY779578
Coelotes_suruga	KY792060	KY791761	KY791460	KY791158	KY779018	KY779305	KY790863	KY779569
Coelotes_terrestris	KY792039	KY791740	KY791439	KY791137	KY778997	KY779284	KY790844	KY779554
Coelotes_thailandensis	KY792072	KY791773	KY791472	KY791170	KX555507	KY779317	KY790874	KY779579
Coelotes_xinjiangensis		KY791785	KY791484	KY791182	KY779027	KY779329	KY790886	KY779590
Coras_juvenilis	KY791846	KY791547	KY791246	KY790947	KY778834	KY779091	KY790657	
Coras_lamellosus	KY791847	KY791548	KY791247	KY790948	KY778835		KY790658	

Coras_montanus	KY791848	KY791549	KY791248	KY790949	KY778836		KY790659	KY779378
Coras_perplexus	KY791849	KY791550			KY778837	KY779094	KY790660	
Draconarius_altissimus	KY791856	KY791557	KY791256	KY790956	KY778844	KY779101	KY790666	KY779383
Draconarius_anthonyi	KY791868	KY791569	KY791268	KY790968	KY778856	KY779113	KY790677	KY779395
Draconarius_bannaensis	KY791912	KY791613	KY791312	KY791010	KY778900	KY779157	KY790720	
Draconarius_brachialis	KY791890	KY791591	KY791290	KY790988	KY778878	KY779135	KY790698	KY779416
Draconarius_capitulatus	KY791918	KY791619	KY791318	KY791016	KY778906	KY779163	KY790726	KY779441
Draconarius_carinatus	KY791898	KY791599	KY791298	KY790996	KY778886	KY779143	KY790706	KY779424
Draconarius_curvabilis	KY791857	KY791558	KY791257	KY790957	KY778845	KY779102	KY790667	KY779384
Draconarius_exilis	KY791928	KY791629	KY791328	KY791026	KY778916	KY779173	KY790736	KY779450
Draconarius_falcatus	KY791919	KY791620	KY791319	KY791017	KY778907	KY779164	KY790727	
Draconarius_hanoiensis	KY791914	KY791615	KY791314	KY791012	KY778902	KY779159	KY790722	KY779437
Draconarius_himlayaensis	KY791920	KY791621	KY791320	KY791018	KY778908	KY779165	KY790728	KY779442
Draconarius_lutulentus	KY791851	KY791552	KY791251	KY790951	KY778839	KY779096	KY790662	KY779380
Draconarius_papai	KY791899	KY791600	KY791299	KY790997	KY778887	KY779144	KY790707	
Draconarius_paralateralis	KY791913	KY791614	KY791313	KY791011	KY778901	KY779158	KY790721	KY779436
Draconarius_paraspiralis	KY791921	KY791622	KY791321	KY791019	KY778909	KY779166	KY790729	KY779443
Draconarius_paratrifasciatus	KY791902	KY791603	KY791302	KY791000	KY778890	KY779147	KY790710	KY779427

Draconarius_patellabifidus	KY791922	KY791623	KY791322	KY791020	KY778910	KY779167	KY790730	KY779444
Draconarius_picta	KY791858	KY791559	KY791258	KY790958	KY778846	KY779103		KY779385
Draconarius_pseudobrunneus	KY791870	KY791571	KY791270	KY790970	KY778858	KY779115	KY790679	KY779397
Draconarius_pseudocapitulatus	KY791923	KY791624	KY791323	KY791021	KY778911	KY779168	KY790731	KY779445
Draconarius_pseudocoreanus	KY791903	KY791604	KY791303	KY791001	KY778891	KY779148	KY790711	KY779428
Draconarius_pseudospiralis	KY791924	KY791625	KY791324	KY791022	KY778912	KY779169	KY790732	KY779446
Draconarius_simplicidens	KY791805	KY791506	KY791205	KY790907	KY778795	KY779050	KY790625	KY779347
Draconarius_sonqi	KY791901	KY791602	KY791301	KY790999	KY778889	KY779146	KY790709	KY779426
Draconarius_sp1	KY791894	KY791595	KY791294	KY790992	KY778882	KY779139	KY790702	KY779420
Draconarius_sp10	KY791915	KY791616	KY791315	KY791013	KY778903	KY779160	KY790723	KY779438
Draconarius_sp11	KY791925	KY791626	KY791325	KY791023	KY778913	KY779170	KY790733	KY779447
Draconarius_sp12	KY791926	KY791627	KY791326	KY791024	KY778914	KY779171	KY790734	KY779448
Draconarius_sp13	KY791882	KY791583	KY791282	KY790980	KY778870	KY779127	KY790690	KY779408
Draconarius_sp14	KY791927	KY791628	KY791327	KY791025	KY778915	KY779172	KY790735	KY779449
Draconarius_sp15	KY791929	KY791630	KY791329	KY791027	KY778917	KY779174	KY790737	KY779451
Draconarius_sp16	KY791859	KY791560	KY791259	KY790959	KY778847	KY779104	KY790668	KY779386

Draconarius_sp17	KY791869	KY791570	KY791269	KY790969	KY778857	KY779114	KY790678	KY779396
Draconarius_sp18		KY791561	KY791260	KY790960	KY778848	KY779105	KY790669	KY779387
Draconarius_sp19	KY791861	KY791562	KY791261	KY790961	KY778849	KY779106	KY790670	KY779388
Draconarius_sp2	KY791885	KY791586	KY791285	KY790983	KY778873	KY779130	KY790693	KY779411
Draconarius_sp20	KY791930	KY791631	KY791330	KY791028	KY778918	KY779175	KY790738	KY779452
Draconarius_sp21	KY791888	KY791589	KY791288	KY790986	KY778876	KY779133	KY790696	KY779414
Draconarius_sp22	KY791889	KY791590	KY791289	KY790987	KY778877	KY779134	KY790697	KY779415
Draconarius_sp23	KY791877	KY791578	KY791277	KY790975	KY778865	KY779122	KY790686	KY779403
Draconarius_sp24	KY791872	KY791573	KY791272		KY778860	KY779117	KY790681	KY779399
Draconarius_sp25	KY791873	KY791574	KY791273		KY778861	KY779118	KY790682	KY779400
Draconarius_sp26	KY791891	KY791592	KY791291	KY790989	KY778879	KY779136	KY790699	KY779417
Draconarius_sp27	KY791853	KY791554	KY791253	KY790953	KY778841	KY779098	KY790664	KY779382
Draconarius_sp28	KY791862	KY791563	KY791262	KY790962	KY778850	KY779107	KY790671	KY779389
Draconarius_sp29	KY791892	KY791593	KY791292	KY790990	KY778880	KY779137	KY790700	KY779418
Draconarius_sp3	KY791886	KY791587	KY791286	KY790984	KY778874	KY779131	KY790694	KY779412
Draconarius_sp30	KY791819	KY791520	KY791219	KY790921	KY778807	KY779064		KY779360

Draconarius_sp31	KY791820	KY791521	KY791220	KY790922	KY778808	KY779065	KY790634	KY779361
Draconarius_sp32	KY791931	KY791632	KY791331	KY791029	KY778919	KY779176	KY790739	KY779453
Draconarius_sp33	KY791905	KY791606	KY791305	KY791003	KY778893	KY779150	KY790713	
Draconarius_sp34	KY791906	KY791607	KY791306	KY791004	KY778894	KY779151	KY790714	KY779430
Draconarius_sp35	KY791900	KY791601	KY791300	KY790998	KY778888	KY779145	KY790708	KY779425
Draconarius_sp36	KY791893	KY791594	KY791293	KY790991	KY778881	KY779138	KY790701	KY779419
Draconarius_sp37	KY791878	KY791579	KY791278	KY790976	KY778866	KY779123	KY790687	KY779404
Draconarius_sp38	KY791863	KY791564	KY791263	KY790963	KY778851	KY779108	KY790672	KY779390
Draconarius_sp39	KY791864	KY791565	KY791264	KY790964	KY778852	KY779109	KY790673	KY779391
Draconarius_sp4	KY791904	KY791605	KY791304	KY791002	KY778892	KY779149	KY790712	KY779429
Draconarius_sp40	KY791854	KY791555	KY791254	KY790954	KY778842	KY779099	KY790665	
Draconarius_sp41	KY791879	KY791580	KY791279	KY790977	KY778867	KY779124	KY790688	KY779405
Draconarius_sp42	KY791871	KY791572	KY791271	KY790971	KY778859	KY779116	KY790680	KY779398
Draconarius_sp43	KY791821	KY791522	KY791221	KY790923	KY778809	KY779066	KY790635	KY779362
Draconarius_sp44	KY791822	KY791523	KY791222	KY790924	KY778810	KY779067	KY790636	KY779363
Draconarius_sp45	KY791855	KY791556	KY791255	KY790955	KY778843	KY779100		
Draconarius_sp46	KY791932	KY791633	KY791332	KY791030	KY778920	KY779177	KY790740	KY779454

Draconarius_sp47	KY791907	KY791608	KY791307	KY791005	KY778895	KY779152	KY790715	KY779431
Draconarius_sp48	KY791806	KY791507	KY791206	KY790908	KY778796	KY779051	KY790626	KY779348
Draconarius_sp49	KY791874	KY791575	KY791274	KY790972	KY778862	KY779119	KY790683	
Draconarius_sp5	KY791887	KY791588	KY791287	KY790985	KY778875	KY779132	KY790695	KY779413
Draconarius_sp50	KY791933	KY791634	KY791333	KY791031	KY778921	KY779178	KY790741	KY779455
Draconarius_sp51	KY791880	KY791581	KY791280	KY790978	KY778868	KY779125	KY790689	KY779406
Draconarius_sp52	KY791908	KY791609	KY791308	KY791006	KY778896	KY779153	KY790716	KY779432
Draconarius_sp53	KY791909		KY791309	KY791007	KY778897	KY779154	KY790717	KY779433
Draconarius_sp54	KY791934	KY791635	KY791334	KY791032	KY778922	KY779179	KY790742	KY779456
Draconarius_sp55	KY791911	KY791612	KY791311	KY791009	KY778899	KY779156	KY790719	KY779435
Draconarius_sp56	KY791881	KY791582	KY791281	KY790979	KY778869	KY779126		KY779407
Draconarius_sp57	KY791935	KY791636	KY791335	KY791033	KY778923	KY779180	KY790743	KY779457
Draconarius_sp58	KY791876	KY791577	KY791276	KY790974	KY778864	KY779121	KY790685	KY779402
Draconarius_sp6	KY791818	KY791519	KY791218	KY790920	KY778806	KY779063	KY790633	KY779359
Draconarius_sp7	KY791816	KY791517	KY791216	KY790918	KY778804	KY779061	KY790631	
Draconarius_sp8	KY791852	KY791553	KY791252	KY790952	KY778840	KY779097	KY790663	KY779381
Draconarius_sp9	KY791817	KY791518	KY791217	KY790919	KY778805	KY779062	KY790632	KY779358

Draconarius_spirallus	KY791884	KY791585	KY791284	KY790982	KY778872	KY779129	KY790692	KY779410
Draconarius_streptus	KY791910	KY791611	KY791310	KY791008	KY778898	KY779155	KY790718	KY779434
Draconarius_sublutulentus	KY791850	KY791551	KY791250	KY790950	KY778838	KY779095	KY790661	KY779379
Draconarius_subtitanus	KY791865	KY791566	KY791265	KY790965	KY778853	KY779110	KY790674	KY779392
Draconarius_taihangensis		KY791524	KY791223	KY790925	KY778811	KY779068	KY790637	
Draconarius_tibetensis	KY791875	KY791576	KY791275	KY790973	KY778863	KY779120	KY790684	KY779401
Draconarius_tortus	KY791834	KY791535	KY791234	KY790936	KY778822	KY779079	KY790647	KY779371
Draconarius_transversus	KY791916	KY791617	KY791316	KY791014	KY778904	KY779161	KY790724	KY779439
Draconarius_triatus	KY791824	KY791525	KY791224	KY790926	KY778812	KY779069	KY790638	
Draconarius_trifasciatus	KY791897	KY791598	KY791297	KY790995	KY778885	KY779142	KY790705	KY779423
Draconarius_venustus		KY791567	KY791266	KY790966	KY778854	KY779111	KY790675	KY779393
Draconarius_yani	KY791867	KY791568	KY791267	KY790967	KY778855	KY779112	KY790676	KY779394
Eratigena_agrestis	KY791801	KY791502	KY791201	KY790903	KY778791	KY779046	KY790621	
Eratigena_atrica		AY138823		FR694030	FR714883		FN678928	
Eratigena_feminea				FR694020	FN554783		FN678932	
Eratigena_vomeroi				FR694045	FN554814		FN678945	
Flexicoelotes_huyunensis	KY791945	KY791646	KY791345	KY791043	KT727020	KY779190	KY790753	KY779466
Flexicoelotes_jiaohanyanensis	KY791949	KY791650	KY791349	KY791047	KT727021	KY779194	KY790757	KY779469

Flexicoelotes_jinlongensis	KY791947	KY791648	KY791347	KY791045	KT727018	KY779192	KY790755	KY779467
Flexicoelotes_pingzhaiensis	KY791946	KY791647	KY791346	KY791044	KT727019	KY779191	KY790754	
Flexicoelotes_xingwangensis	KY791948	KY791649	KY791348	KY791046	KT727017	KY779193	KY790756	KY779468
Himalcoelotes_xizangensis	KY791883	KY791584	KY791283	KY790981	KY778871	KY779128	KY790691	KY779409
Histopona_torpida1		DQ628580		FR694010	FN554793		FN678948	
Histopona_torpida2	KY791823	KY791610	KY791249	KY791185	KY779030	KY779092	KY790889	KY779593
Hololena_adnexa		DQ628576		DQ628596	DQ628554			
Hololena_curta		DQ628578		DQ628598	DQ628556			
Inermocoelotes_anoplus	KY792050	KY791751	KY791450	KY791148	KY779008	KY779295	KY790854	KY779564
Inermocoelotes_brevispinus	KY792043	KY791744	KY791443	KY791141	KY779001	KY779288	KY790848	KY779557
Inermocoelotes_falciger	KY792051	KY791752	KY791451	KY791149	KY779009	KY779296	KY790855	KY779565
Inermocoelotes_inermis_CZ	KY792044	KY791745	KY791444	KY791142	KY779002	KY779289		KY779558
Inermocoelotes_inermis_RO	KY792042	KY791743	KY791442	KY791140	KY779000	KY779287	KY790847	KY779556
Inermocoelotes_jurinitschi	KY792048	KY791749	KY791448	KY791146	KY779006	KY779293	KY790852	KY779562
Inermocoelotes_karlinskii	KY792049	KY791750	KY791449	KY791147	KY779007	KY779294	KY790853	KY779563
Inermocoelotes_kulczynskii	KY792052		KY791452	KY791150	KY779010	KY779297	KY790856	
Inermocoelotes_microlepidus	KY792047	KY791748	KY791447	KY791145	KY779005	KY779292	KY790851	KY779561

Inermocoelotes_paramicrolepidus	KY792053	KY791754	KY791453	KY791151	KY779011	KY779298	KY790857	KY779566
Inermocoelotes_sp	KY792038	KY791739	KY791438	KY791136	KY778996	KY779283	KY790843	KY779553
Iwogumoa_insidiosa	KY791840	KY791541	KY791240	KY790941	KY778828	KY779085	KY790651	KY779376
Iwogumoa_portus	KY791841	KY791542	KY791241	KY790942	KY778829	KY779086	KY790652	
Iwogumoa_songminjae		JN816577	JN816793	JN817001	JN817211			
Iwogumoa_sp	KY791842	KY791543	KY791242	KY790943	KY778830	KY779087	KY790653	
Iwogumoa_xinhuiensis1	KY791843	KY791544	KY791243	KY790944	KY778831	KY779088	KY790654	
Iwogumoa_xinhuiensis2	KY791844	KY791545	KY791244	KY790945	KY778832	KY779089	KY790655	
Iwogumoa_yushanensis	KY791845	KY791546	KY791245	KY790946	KY778833	KY779090	KY790656	KY779377
Lineacoelotes_bicultratus	KY791936	KY791637	KY791336	KY791034	KY778924	KY779181	KY790744	
Lineacoelotes_funiushanensis	KY791937	KY791638	KY791337	KY791035	KY778925	KY779182	KY790745	KY779458
Lineacoelotes_sp1	KY791938	KY791639	KY791338	KY791036	KY778926	KY779183	KY790746	KY779459
Lineacoelotes_sp2	KY791939	KY791640	KY791339	KY791037	KY778927	KY779184	KY790747	KY779460
Lineacoelotes_sp3	KY791940	KY791641	KY791340	KY791038	KY778928	KY779185	KY790748	KY779461
Lineacoelotes_sp4	KY791941	KY791642	KY791341	KY791039	KY778929	KY779186	KY790749	KY779462
Lineacoelotes_sp5	KY791942	KY791643	KY791342	KY791040	KY778930	KY779187	KY790750	KY779463
Longicoelotes_karschi	KY791943	KY791644	KY791343	KY791041	KY778931	KY779188	KY790751	KY779464
Longicoelotes_sp	KY791944	KY791645	KY791344	KY791042	KY778932	KY779189	KY790752	KY779465
Maimuna_cretica					FN554795		FN678953	

Malthonica_lusitanica				FR694058			FN678956	
Malthonica_oceanica				FR694059	FN554792		FN678957	
Notiocoelotes_lingulatus	KY791807	KY791508	KY791207	KY790909	KY778797	KY779052		KY779349
Notiocoelotes_maoganensis	KY791812	KY791513	KY791212	KY790914	KU886075	KY779057		KY779354
Notiocoelotes_orbiculatus	KY791808	KY791509	KY791208	KY790910	KY778798	KY779053		KY779350
Notiocoelotes_palinitropus	KY791809	KY791510	KY791209	KY790911	KY778799	KY779054	KY790627	KY779351
Notiocoelotes_pseudolingulatus	KY791810	KY791511	KY791210	KY790912	KY778800	KY779055	KY790628	KY779352
Notiocoelotes_qiongzhongensis	KY791813	KY791514	KY791213	KY790915	KU886074	KY779058		KY779355
Notiocoelotes_sp	KY791814	KY791515	KY791214	KY790916	KY778802	KY779059		KY779356
Notiocoelotes_sparus	KY791811	KY791512	KY791211	KY790913	KY778801	KY779056	KY790629	KY779353
Novalena_intermedia		DQ628568	DQ628752, DQ628715	DQ628679	DQ628618			
Orumcekia_sp	KY791917	KY791618	KY791317	KY791015	KY778905	KY779162	KY790725	KY779440
Papiliocoelotes_guanyinensis	KY791955	KY791656	KY791355	KY791053	KU991801	KY779200	KY790762	KY779473
Papiliocoelotes_guitangensis	KY791958	KY791659	KY791358	KY791056	KU991804	KY779203	KY790765	KY779476
Papiliocoelotes_jiepingensis	KY791957	KY791658	KY791357	KY791055	KU991803	KY779202	KY790764	KY779475
Papiliocoelotes_meiyuensis	KY791956	KY791657	KY791356	KY791054	KU991802	KY779201	KY790763	KY779474
Papiliocoelotes_yezhouensis	KY791954	KY791655	KY791354	KY791052	KU991800	KY779199	KY790761	KY779472

Pireneitega_burqinensis	KY792036	KY791737	KY791436	KY791134	KX011867	KY779281	KY790841	KY779551
Pireneitega_fuyunensis	KY792033	KY791734	KY791433	KY791131	KX011859	KY779278	KY790838	KY779548
Pireneitega_gongliuensis	KY792022	KY791723	KY791422	KY791120	KX011862	KY779267	KY790827	KY779537
Pireneitega_huochengensis	KY792020	KY791721	KY791420	KY791118	KX011861	KY779265	KY790825	KY779535
Pireneitega_involuta	KY792004	KY791705	KY791404	KY791102	KY778972	KY779249	KY790809	KY779519
Pireneitega_lini	KY792029	KY791730	KY791429	KY791127	KX011865	KY779274	KY790834	KY779544
Pireneitega_liui	KY792035	KY791736	KY791435	KY791133	KX011860	KY779280	KY790840	KY779550
Pireneitega_luctuosus1	KY792005	KY791706	KY791405	KY791103	KY778973	KY779250	KY790810	KY779520
Pireneitega_luctuosus2	KY792006	KY791707	KY791406	KY791104	KY778974	KY779251	KY790811	KY779521
Pireneitega_luniformis1	KY792007	KY791708	KY791407	KY791105	KY778975	KY779252	KY790812	KY779522
Pireneitega_luniformis2	KY792008	KY791709	KY791408	KY791106	KY778976	KY779253	KY790813	KY779523
Pireneitega_major		KY791735	KY791434	KY791132	KY778994	KY779279	KY790839	KY779549
Pireneitega_neglecta	KY792009	KY791710	KY791409	KY791107	KY778977	KY779254	KY790814	KY779524
Pireneitega_ovtchinnikovii	KY792010	KY791711	KY791410	KY791108	KY778978	KY779255	KY790815	KY779525
Pireneitega_sp1	KY792023	KY791724	KY791423	KY791121	KY778988	KY779268	KY790828	KY779538
Pireneitega_sp10	KY792019	KY791720	KY791419	KY791117	KY778986	KY779264	KY790824	KY779534

Pireneitega_sp11	KY792021	KY791722	KY791421	KY791119	KY778987	KY779266	KY790826	KY779536
Pireneitega_sp12	KY792024	KY791725	KY791424	KY791122	KY778989	KY779269	KY790829	KY779539
Pireneitega_sp13	KY792025	KY791726	KY791425	KY791123	KY778990	KY779270	KY790830	KY779540
Pireneitega_sp14	KY792026	KY791727	KY791426	KY791124	KY778991	KY779271	KY790831	KY779541
Pireneitega_sp15	KY792027	KY791728	KY791427	KY791125	KY778992	KY779272	KY790832	KY779542
Pireneitega_sp2	KY792012	KY791713	KY791412	KY791110	KY778980	KY779257	KY790817	KY779527
Pireneitega_sp3	KY792013	KY791714	KY791413	KY791111	KY778981	KY779258	KY790818	KY779528
Pireneitega_sp4	KY792014	KY791715	KY791414	KY791112	KY778982	KY779259	KY790819	KY779529
Pireneitega_sp5		KY791732	KY791431	KY791129	KY778993	KY779276	KY790836	KY779546
Pireneitega_sp6	KY792015	KY791716	KY791415	KY791113	KY593331	KY779260	KY790820	KY779530
Pireneitega_sp7	KY792016	KY791717	KY791416	KY791114	KY778983	KY779261	KY790821	KY779531
Pireneitega_sp8	KY792017	KY791718	KY791417	KY791115	KY778984	KY779262	KY790822	KY779532
Pireneitega_sp9	KY792018	KY791719	KY791418	KY791116	KY778985	KY779263	KY790823	KY779533
Pireneitega_spinivulva	KY792011	KY791712	KY791411	KY791109	KY778979	KY779256	KY790816	KY779526
Pireneitega_tianchiensis_TC	KY792037	KY791738	KY791437	KY791135	KY778995	KY779282	KY790842	KY779552
Pireneitega_wensuensis	KY792028	KY791729	KY791428	KY791126	KX011864	KY779273	KY790833	KY779543

Pireneitega_wui	KY792030	KY791731	KY791430	KY791128	KX011866	KY779275	KY790835	KY779545
Pireneitega_yaoi	KY792032	KY791733	KY791432	KY791130	KX011863	KY779277	KY790837	KY779547
Platocoelotes_ampulliformis	KY791970	KY791671	KY791370	KY791068	KY778946	KY779215	KY790777	KY779487
Platocoelotes_brevis	KY791959	KY791660	KY791359	KY791057	KY778935	KY779204	KY790766	KY779477
Platocoelotes_furcatus	KY791960	KY791661	KY791360	KY791058	KY778936	KY779205	KY790767	KY779478
Platocoelotes_globosus	KY791961	KY791662	KY791361	KY791059	KY778937	KY779206	KY790768	KY779479
Platocoelotes_icohamatoides	KY791962	KY791663	KY791362	KY791060	KY778938	KY779207	KY790769	KY779480
Platocoelotes_imperfectus	KY791963	KY791664	KY791363	KY791061	KY778939	KY779208	KY790770	KY779481
Platocoelotes_impletus	KY791964	KY791665	KY791364	KY791062	KY778940	KY779209	KY790771	KY779482
Platocoelotes_kailiensis	KY791971	KY791672	KY791371	KY791069	KY778947	KY779216	KY790778	KY779488
Platocoelotes_latus1	KY791984	KY791685	KY791384	KY791082	KY778956	KY779229	KY790791	KY779500
Platocoelotes_latus2	KY791965	KY791666	KY791365	KY791063	KY778941	KY779210	KY790772	KY779483
Platocoelotes_lichuanensis	KY791966	KY791667	KY791366	KY791064	KY778942	KY779211	KY790773	KY779484
Platocoelotes_luoi	KY791979	KY791680	KY791379	KY791077	KR065578	KY779224	KY790786	KY779495
Platocoelotes_paralatus	KY791967	KY791668	KY791367	KY791065	KY778943	KY779212	KY790774	KY779485
Platocoelotes_qinglinensis	KY791968	KY791669	KY791368	KY791066	KY778944	KY779213	KY790775	KY779486

Platocoelotes_shuiensis	KY791978	KY791679	KY791378	KY791076	KR065580	KY779223	KY790785	KY779494
Platocoelotes_sp1	KY791972	KY791673	KY791372	KY791070	KY778948	KY779217	KY790779	KY779489
Platocoelotes_sp2	KY791969	KY791670	KY791369	KY791067	KY778945	KY779214	KY790776	
Platocoelotes_sp3	KY791975	KY791676	KY791375	KY791073	KY778951	KY779220	KY790782	
Platocoelotes_sp4	KY791973	KY791674	KY791373	KY791071	KY778949	KY779218	KY790780	KY779490
Platocoelotes_sp5	KY791974	KY791675	KY791374	KY791072	KY778950	KY779219	KY790781	KY779491
Platocoelotes_sp6	KY791976	KY791677	KY791376	KY791074	KY778952	KY779221	KY790783	KY779492
Platocoelotes_sp7	KY791977	KY791678	KY791377	KY791075	KY778953	KY779222	KY790784	KY779493
Platocoelotes_sp8	KY791980	KY791681	KY791380	KY791078	KY778954	KY779225	KY790787	KY779496
Platocoelotes_strombuliformis	KY791983	KY791684	KY791383	KY791081	KY778955	KY779228	KY790790	KY779499
Platocoelotes_tianyangensis	KY791982	KY791683	KY791382	KY791080	KR065581	KY779227	KY790789	KY779498
Platocoelotes_xianwuensis	KY791981	KY791682	KY791381	KY791079	KR065577	KY779226	KY790788	KY779497
sp1	KY791998	KY791699	KY791398	KY791096	KY778966	KY779243	KY790804	KY779513
sp10	KY792001	KY791702	KY791401	KY791099	KY778969	KY779246	KY790806	KY779516
sp11	KY791830	KY791531	KY791230	KY790932	KY778818	KY779075	KY790643	KY779367
sp12	KY791831	KY791532	KY791231	KY790933	KY778819	KY779076	KY790644	KY779368
sp13	KY791835	KY791536	KY791235	KY790937	KY778823	KY779080	KY790648	KY779372

sp14	KY791825	KY791526	KY791225	KY790927	KY778813	KY779070	KY790639	KY779364
sp15	KY791832	KY791533	KY791232	KY790934	KY778820	KY779077	KY790645	KY779369
sp16	KY791833	KY791534	KY791233	KY790935	KY778821	KY779078	KY790646	KY779370
sp17	KY791953	KY791654	KY791353	KY791051	KY778934	KY779198	KY790760	KY779471
sp18	KY791836	KY791537	KY791236	KY790938	KY778824	KY779081		KY779373
sp19	KY791837	KY791538	KY791237	KY790939	KY778825	KY779082	KY790649	KY779374
sp2	KY791827	KY791528	KY791227	KY790929	KY778815	KY779072	KY790641	KY779366
sp20	KY792002	KY791703	KY791402	KY791100	KY778970	KY779247	KY790807	KY779517
sp21	KY792003	KY791704	KY791403	KY791101	KY778971	KY779248	KY790808	KY779518
sp3	KY791826	KY791527	KY791226	KY790928	KY778814	KY779071	KY790640	KY779365
sp4	KY791828	KY791529	KY791228	KY790930	KY778816	KY779073	KY790642	
sp5	KY792061	KY791762	KY791461	KY791159	KY779019	KY779306	KY790864	KY779570
sp6	KY791999	KY791700	KY791399	KY791097	KY778967	KY779244	KY790805	KY779514
sp7	KY791952	KY791653	KY791352	KY791050	KY778933	KY779197		
sp8	KY791829	KY791530	KY791229	KY790931	KY778817	KY779074		
sp9	KY792000	KY791701	KY791400	KY791098	KY778968	KY779245		KY779515
Spiricoelotes_anshiensis	KY791991	KY791692	KY791391	KY791089	KT896546	KY779236	KY790798	KY779506

Spiricoelotes_chufengensis	KY791987	KY791688	KY791387	KY791085	KT896541	KY779232	KY790794	KY779503
Spiricoelotes_nansheensis	KY791951	KY791652	KY791351	KY791049	KT896544	KY779196	KY790759	
Spiricoelotes_sp1	KY791985	KY791686	KY791385	KY791083	KY778957	KY779230	KY790792	KY779501
Spiricoelotes_sp2	KY791986	KY791687	KY791386	KY791084	KY778958	KY779231	KY790793	KY779502
Spiricoelotes_sp3	KY791989	KY791690	KY791389	KY791087	KY778959	KY779234	KY790796	
Spiricoelotes_sp4	KY791992	KY791693	KY791392	KY791090	KY778960	KY779237	KY790799	KY779507
Spiricoelotes_taipingensis	KY791988	KY791689	KY791388	KY791086	KT896542	KY779233	KY790795	KY779504
Spiricoelotes_xianheensis	KY791950	KY791651	KY791350	KY791048	KT896543	KY779195	KY790758	KY779470
Spiricoelotes_xiongxinensis	KY791990	KY791691	KY791390	KY791088	KT896545	KY779235	KY790797	KY779505
Tegecoelotes_corasides	KY792062	KY791763	KY791462	KY791160	KY779020	KY779307	KY790865	KY779571
Tegecoelotes_secundus		JN816578	JN816794	JN817002	JN817212			
Tegenaria_ariadnae	KY791795	KY791496	KY791195	KY790897	KY778785	KY779040	KY790615	KY779339
Tegenaria_circeoensis				FR694040			FN678961	
Tegenaria_dalmatica	KY791788	KY791489	KY791188	KY790890	KY778778	KY779033	KY790608	KY779332
Tegenaria_domestica1	KY791789	KY791490	KY791189	KY790891	KY778779	KY779034	KY790609	KY779333
Tegenaria_domestica2	KY791799	KY791500	KY791199	KY790901	KY778789	KY779044	KY790619	KY779343
Tegenaria_faniapollinis	KY791790	KY791491	KY791190	KY790892	KY778780	KY779035	KY790610	KY779334
Tegenaria_ferruginea	KY791800	KY791501	KY791200	KY790902	KY778790	KY779045	KY790620	KY779344

Tegenaria_hasperi	KY791791	KY791492	KY791191	KY790893	KY778781	KY779036	KY790611	KY779335
Tegenaria_henroti				FR694021	FN554771			
Tegenaria_lapicidinarum	KY791792	KY791493	KY791192	KY790894	KY778782	KY779037	KY790612	KY779336
Tegenaria_mirifica				FR694013	FN554775			
Tegenaria_pagana	KY791793	KY791494	KY791193	KY790895	KY778783	KY779038	KY790613	KY779337
Tegenaria_parietina	KY791794	KY791495	KY791194	KY790896	KY778784	KY779039	KY790614	KY779338
Tegenaria_parmenidis				FR694032	FN554773		FN678986	
Tegenaria_parvula				FR694036			FN678990	
Tegenaria_pieperi	KY791796	KY791497	KY791196	KY790898	KY778786	KY779041	KY790616	KY779340
Tegenaria_rilaensis				FR694023			FN678991	
Tegenaria_schmalfussi	KY791797	KY791498	KY791197	KY790899	KY778787	KY779042	KY790617	KY779341
Tegenaria_silvestris				FR694007	JQ412460		FN678992	
Tegenaria_sp	KY791798	KY791499	KY791198	KY790900	KY778788	KY779043	KY790618	KY779342
Tegenaria_tridentina				FR694014	FN554776		FN678996	
Tegenaria_vankeerorum					FN554779		FN678995	
Textrix_caudata				FR694047	FN554803		FN678997	
Textrix_denticulata		DQ628582	DQ628755, DQ628718	DQ628682	DQ628621	DQ628647	FN679000	
Tonsilla_defossa	KY791895	KY791596	KY791295	KY790993	KY778883	KY779140	KY790703	KY779421
Tonsilla_lyrata	KY791993		KY791393	KY791091	KY778961	KY779238	KY790800	KY779508
Tonsilla_sp1	KY791994	KY791695	KY791394	KY791092	KY778962	KY779239	KY790801	KY779509

Tonsilla_sp2	KY791995	KY791696	KY791395	KY791093	KY778963	KY779240	KY790802	KY779510
Tonsilla_truculenta	KY791996	KY791697	KY791396	KY791094	KY778964	KY779241	KY790803	KY779511
Wadotes_calcaratus					GU682855			
Wadotes_dixiensis			DQ628721, DQ628758	DQ628685	DQ628623			
Wadotes_hybridus	KY791838	KY791539	KY791238		KY778826	KY779083		

Table S3. Species and GenBank accession numbers used for phylogeny reconstruction of *Cryptocercus* (Insecta, Blattodea, Cryptocercidae).

Species	**12S**	**16S**	**COII**	**COI**	**18S**	**28S**	**H3**
Cryptocercus arcuatus	KR262967	KR262992	KR263017	KY940609	MK909308	KU312230	MK909474
Cryptocercus banshanmenensis	MK909175	MK909232	MK909398	KY940590	MK909293	KY944600	MK909459
Cryptocercus changbaiensis	MK909204	MK909261	MK909427	MK909527	MK909343	MK909370	MK909509
Cryptocercus chengkouensis	MK909186	MK909241	MK909409	KY940606	MK909306	KY944610	MK909472
Cryptocercus clevelandi	AF339638	AF339650	DQ007626				
Cryptocercus convexus	KR262966	KR262991	KR263016	KY940623	MK909320	KU312241	MK909486
Cryptocercus darwini	AY222737	AY222696		KY940656		KY944612	
Cryptocercus habaensis	KR262980	KR263005	KR263030	KY940578	MK909283	KU312222	MK909449
Cryptocercus hirtus	KR262970	KR262995	KR263020	KY940620	MK909318	MK909369	MK909484
Cryptocercus kyebangensis	AF310221	AF310220	KF855905	KP986401	FJ806324	KP986242	KF855884
Cryptocercus laojunensis	MK909165	MK909222	MK909388	KY940572	MK909279	KY944594	MK909445
Cryptocercus luanchuanensis	MK909183	MK909238	MK909406	MK913629	MK909301	MK909368	MK909467
Cryptocercus matilei	KR262961	KR262986	KR263011	KY940645	MK909334	KU312255	MK909500
Cryptocercus meridianus	KR262958	KR262983	KR263008	KY940614	MK909314	KU312236	MK909480
Cryptocercus neixiangensis	KR262976	KR263001	KR263026	KY940598	MK909302	KU312228	MK909468
Cryptocercus ningshanensis	KR262959	KR262984	KR263009	KY940629	MK909324	KU312245	MK909490
Cryptocercus pingwuensis	KR262960	KR262985	KR263010	KY940612	MK909312	KU312234	MK909478
Cryptocercus primarius	KR262968	KR262993	KR263018	KY940647	MK909336	KU312257	MK909502
Cryptocercus pudacuoensis	MK909167	MK909224	MK909390	KY940575	MK909281	KY944596	MK909447
Cryptocercus punctulatus	AY222732	AY222691	DQ007631		AY491148		AY491325
Cryptocercus relictus	MK909206	MK909263	MK909429	MK909531	MK909345	MK909372	MK909511
Cryptocercus shangmengensis	KR262981	KR263006	KR263031	KY940581	MK909285	KU312224	MK909451
Cryptocercus shennongjiaensis	KR262964	KR262989	KR263014	KY940627	MK909322	KU312243	MK909488
Cryptocercus sp1	MK909171	MK909228	MK909394	MK913622	MK909289	MK909361	MK909455
Cryptocercus sp2	MK909181	MK942642	MK909404	MK913626	MK909299	MK909366	MK909465

Cryptocercus sp3	MK909179	MK909236	MK909402	MK913624	MK909297	MK909364	MK909463
Cryptocercus tianbaensis	MK909173	MK909230	MK909396	KY940587	MK909291	KY944598	MK909457
Cryptocercus weixiensis	MK909199	MK909256	MK909422	KY940651	MK909338	KY944606	MK909504
Cryptocercus wolongensis	MK909177	MK909234	MK909400	KY940594	MK909295	KY944602	MK909461
Cryptocercus wuxiensis	KR262963	KR262988	KR263013	KY940617	MK909316	KU312238	MK909482
Cryptocercus zagunaoensis	KR262978	KR263003	KR263028	KY940584	MK909287	KU312226	MK909453
Amitermes dentatus	KY224513					DQ441853	
Bantia werneri	FJ806017	FJ806192	FJ806906	FJ802796	FJ806379	FJ806578	FJ806742
Blaptica dubia	DQ874030	JN615274	DQ874266		DQ874113	DQ874199	DQ873950
Coptotermes formosanus	AB626145						
Coptotermes lacteus	JX144934				FJ806332	FJ806530	
Deropeltis erythrocephala	DQ874035	KF855855	DQ874271		DQ874121	DQ874205	DQ873957
Dolichorhinotermes longilabius	KP026258						
Embiratermes brevinasus	KY224580					DQ441913	
Embiratermes neotenicus	KP026262						
Epilampra maya	MG882194						
Galiblatta cribrosa	MG882232				KY497640	KY497720	
Gyna capucina	MG882152				KF372499	KF372447	
Heterotermes sp	JX144936						
Mantis religiosa	U17792	EF383311	EF363226	KR146939	AY859586	FJ806632	AY491327
Mastotermes darwiniensis	JX144929				DQ882638	DQ441950	GU066915
Microcerotermes nervosus	KY224586						
Microcerotermes newmani	KY224394				DQ882639	DQ441956	
Microhodotermes viator	JX144931				DQ882640	DQ441959	
Neotermes insularis	JX144933						
Orthognathotermes aduncus	KP026289						
Panesthia sinuata	MF286797	MF286869	MF287043			MF286928	MF286972
Periplaneta australasiae	MG882181				DQ874169	DQ874242	DQ874000

Phoetalia pallida	DQ874086	JN615278	DQ874314	KF372532	DQ874174	DQ874245	DQ874004
Protagonista lugubris	MF286827	MF286891	MF287076	KU511286		MF286958	MF287018
Reticulitermes flavipes	EF206314				EF363230		
Rhabdoblatta marginata	MF286814	MF286885	MF287074	MK547381		MF286946	MF286989
Salganea raggei	MF286804	MF286876	MF287053		AB036201	MF286936	MF286980
Schedorhinotermes breinli	JX144935						
Tryonicus parvus	KF855799	KF855875	KF855920	KP986437	KF855844	KF855817	KF855896

Table S4. Species and GenBank accession numbers used for phylogeny reconstruction of *Gnaptorina* and *Agnaptoria* (Insecta, Coleoptera, Tenebrionidae).

Species	COI	CYTB	16S	28S
Gnaptorina tishkovi	MK415220		MK415106	MK415198
Gnaptorina compressa	MK415221	MK415066	MK415108	MK415171
Gnaptorina brucei	MK415223	MK415068	MK415110	MK415173
Gnaptorina kangmar	MK415226	MK415071	MK415111	MK415178
Gnaptorina himalaya	MK415227	MK415072	MK415112	MK415180
Gnaptorina cuonaensis	MK415224	MK415069	MK415113	MK415179
Gnaptorina nigera	MK415216	MK423893	MK415098	MK415181
Gnaptorina globithoracalis	MK415217	MK415064	MK415100	MK415182
Gnaptorina ampliptera	MK415163	MK415165	MK415139	MK415149
Gnaptorina pilifera	MK415215	MK423892	MK415102	MK415186
Gnaptorina longicornis	MK415204	MK415084	MK415096	MK415184
Gnaptorina polita	MK415154	MK415170	MK415138	
Gnaptorina crenata1	MK415157		MK415140	MK415150
Gnaptorina crenata2	MK415161	MK415167	MK415145	MK415152
Gnaptorina kashkarovi	MK415213	MK415074	MK415122	MK415188
Gnaptorina fairmairei	MK415212	MK415077	MK415120	MK415194
Gnaptorina rufipes	MK415229	MK415076	MK415121	
Gnaptorina lii	MK415211	MK415081	MK415118	MK415190
Gnaptorina minxiana	MK415231		MK415130	MK415191
Gnaptorina felicitana	MG993069	MK423896	MK415114	MK415183
Gnaptorina proxima	MK415232	MK415087	MK415115	
Gnaptorina media	MK415208	MK415079	MK415116	MK415193
Gnaptorina rugosipensis	MK423899	MK415088	MK415117	
Gnaptorina platytarsia	MK415209	MK415080	MK415125	MK415189
Gnaptorina potanini	MK423897		MK415119	
Gnaptorina miroshnikovi	MK423898		MK415126	
Gnaptorina dongdashanensis	MK415205	MK415082	MK415127	MK415196
Gnaptorina artipenis	MK415155	MK415169	MK415143	MK415153
Agnaptoria sp1	MK415238	MK415094	MK415137	MK415200
Agnaptoria sp2	MK415234	MK415091	MK415133	
Agnaptoria sp3	MK415237	MK415092	MK415136	
Agnaptoria sp4	MK415233	MK415093	MK415134	
Agnaptoria sp5	MK415235	MK415090	MK415131	MK415201
Agnaptoria sp6	MK415239	MK415095	MK415135	MK415202
Agnaptoria sp7	MK415236	MK415089	MK415132	

Table S5. Species and GenBank accession numbers used for phylogeny reconstruction of *Lycodon* (Reptilia, Squamata, Colubridae).

Species	*C-mos*	16S rRNA	*CYTB*	*COI*
Lycodon albofuscus	KX660373	KX660232	KX660501	
Lycodon alcalai	KC010304		KC010345	
Lycodon anakradaya			OM674284	
Lycodon aulicus	KC010307		KC010349	
Lycodon banksi			MH669272	
Lycodon bibonius	KC010309		KC010351	
Lycodon bicolor			OQ282988	
Lycodon butleri	KC010312		KC010354	
Lycodon capucinus	KC010313		KC010355	
Lycodon carinatus	KC347392	KC347352	KC347486	
Lycodon cathaya			MT602075	
Lycodon cavernicolus			KJ607890	
Lycodon chapaensis		MK193954	MK201305	MK064658
Lycodon chrysoprateros	KC010318		KC010360	
Lycodon deccanensis			MW006486	
Lycodon dumerilii	KC010320		KC010363	
Lycodon effraenis	KC010328		KC010376	
Lycodon fasciatus		MK194257	MK201557	OQ416414
Lycodon flavicollis			MW006488	
Lycodon flavozonatus		MK194068	MK201413	MK064753
Lycodon futsingensis	MW177828		MW199798	
Lycodon gongshan	KP901018		KP901026	OQ416324
Lycodon jara	KC010322		KC010367	
Lycodon laoensis	KC010323		KC010368	
Lycodon liuchengchaoi	KC733218		KC733201	OQ416325
Lycodon meridionalis		MK194280	MK201578	MK064927
Lycodon muelleri	KC010327		KC010375	
Lycodon multizonatus	KF732933		KF732926	
Lycodon nympha	KC347399	KC347360	KC347476	
Lycodon obvelatus			MW353745	
Lycodon paucifasciatus		EU999212		
Lycodon pictus			MT845095	
Lycodon rosozonatus		MK194228	MK201531	MK064882
Lycodon rufozonatus	KC733211		KC733194	
Lycodon ruhstrati	KC733224		KC733208	
Lycodon sealei	KC010335		KC010384	
Lycodon semicarinatus		AB008539	AB008539	AB008539
Lycodon septentrionalis	MW177831		MW199801	OQ416411
Lycodon serratus			MW353746	
Lycodon sidiki			KX822583	
Lycodon solivagus				MH274411
Lycodon stormi	KC010331		KC010380	
Lycodon striatus			MK089444	MK089438
Lycodon subcinctus	KC010333		KC010382	
Lycodon synaptor	KP901014		KP901020	
Lycodon truongi			OM674282	
Lycodon zawi	AF471111		AF471040	

Lycodon zayuensis	MW177822		MW199792	OQ416413
Boiga kraepelini		MK194112	MK201444	MK064789

Table S6. Species and GenBank accession numbers used for phylogeny reconstruction of Natricidae (Reptilia, Squamata).

Species	cytb	nd4	16s	cmos	nt3	bdnf	rag1
Adelophis foxi	AF420069	AF420072			KF234031	KF258601	
Afrotrix anoscopus	MW711484		MW699965	MW711535	MW711452	MW711558	MW711589
Afrotrix anoscopus	MW711486	MW711522	MW699967	MW711537		MW711560	MW711591
Amphiesma monticolum	OK315803					OK316030	
Amphiesma stolatum 1	MT587287	MT587313	MT586823	MT587301	MW711471	MW711579	MW711606
Amphiesma stolatum 2	OK315804	OK315863	OK326947	OK315914	OK315972	OK316031	OK316092
Amphiesma stolatum 3	OK315805	OK315864	OK326948	OK315915	OK315973	OK316032	OK316093
Amphiesmoides orticeps	OK315806		OK326949	OK315916		OK316033	
Aspidura ceylonensis	KC347477	KC347527	KC347361	KC347400			
Aspidura drummondhayi	KC347455	KC347519	KC347340	KC347379			
Aspidura guentheri	KC347472	KC347507	KC347341	KC347380			
Aspidura ravani	KC347456	KC347514	KC347342				
Aspidura trachyprocta	KC347473	KC347523	KC347343	KC347382			
Atretium schistosum	KC347487	KC347525		KC347383			
Atretium schistosum	OK315807		OK326950			OK316034	
Blythia hmuifangi	MN993853	MN993868	MT003010	MN993861			MN993876
Blythia reticulata			MT003015	MN993890	OK315974	OK316035	
Clonophis kirtlandii	KF258647	KF258630			KF234026	KF258596	
Fowlea asperrima	KC347480		KC347376	KC347413			
Fowlea flavipunctata	MT587286	MT587312	MT586822	MT587300	MW711470	MW711578	MW711605
Fowlea piscator 1		OK315866		OK315918	OK315976	OK316037	OK316095
Fowlea piscator 2	OK315810	OK315868	OK326953	OK315920	OK315978	OK316039	OK316097
Fowlea punctulata	AF471079	AY487074		AF471106			
Fowlea schnurrenbergeri	KY379922						
Fowlea sp	OK315811		OK326954	OK315922	OK315980	OK316040	OK316099
Fowlea tytleri	MG999955	KX017173					
Fowlea yunnensis	GQ281787	JQ687423		JQ687448	KJ685764		KJ685602
Haldea striatula	KF258657	KF258640			KF234036	KF258606	
Hebius andreae	MK253674			MK253675	MK253676		MK253677
Hebius anmensis	OK315812		OK326955			OK316041	OK316100

Hebius atemporalis 1	KJ685695			KJ685645	KJ685747		KJ685587
Hebius atemporalis 2	KJ685680			KJ685630	KJ685732		KJ685572
Hebius atemporalis 3	OK315813	OK315869	OK326956	OK315923	OK315981	OK316042	OK316101
Hebius bitaeniatus 1	OK315815	OK315870	OK326958	OK315925	OK315983	OK316044	OK316103
Hebius bitaeniatus 2	OK315816	OK315871	OK326959	OK315926	OK315984	OK316045	OK316104
Hebius bitaeniatus 3	KJ685688			KJ685648	KJ685740		KJ685580
Hebius boulengeri 1	KJ685684			KJ685634	KJ685736		KJ685576
Hebius boulengeri 2	OK315817	OK315872	OK326960	OK315927	OK315985	OK316046	OK316105
Hebius boulengeri 3	OK315818	OK315873	OK326961	OK315928	OK315986	OK316047	OK316106
Hebius chapaensis	OK315819	OK315874	OK326962	OK315929	OK315987	OK316048	OK316107
Hebius concelarus	AB989268	AB989270		AB989271	LC047778		LC047774
Hebius craspedogaster	KJ685682			KJ685632	KJ685734		KJ685574
Hebius deschauenseei 1	KJ685665			KJ685614	KJ685715		KJ685558
Hebius deschauenseei 2	OK315820	OK315875	OK326963	OK315930	OK315988	OK316049	OK316108
Hebius ishigakiensis	AB989284	AB989286		AB989287	LC047781		LC047772
Hebius johannis	KJ685678			KJ685628	KJ685731		KJ685571
Hebius khasiensis	KJ685668		OK326964	KJ685617	KJ685718		KJ685561
Hebius khasiensis	KJ685663			KJ685612	KJ685713		KJ685556
Hebius leucomystax	OK315822	OK315876	OK326965	OK315932	OK315989	OK316050	OK316109
Hebius maximus	KJ685706			KJ685656	KJ685765		KJ685603
Hebius metusia	KJ685707			KJ685657	KJ685766		
Hebius modestus	OK315823		OK326966	OK315933			
Hebius octolineatus 1	OK315824	OK315877	OK326967	OK315934	OK315990	OK316051	OK316110
Hebius octolineatus 2	KJ685673			KJ685623	KJ685724		
Hebius optatus	KJ685687			KJ685637	KJ685739		KJ685579
Hebius optatus	KJ685662			KJ685611	KJ685712		KJ685555
Hebius popei	KJ685697			KJ685647	KJ685749		KJ685588
Hebius pryeri 1	AB989102	AB989104		AB989105	LC047779		LC047776
Hebius pryeri 2	LC325312			LC325758			
Hebius sangzhiensis	MK340763						
Hebius sauteri	KJ685701			KJ685651	KJ685754		KJ685592
Hebius sp1	OK315825	OK315878	OK326968	OK315935	OK315991	OK316052	OK316111

Hebius sp2	OK315826	OK315879	OK326969	OK315936	OK315992	OK316053	OK316112
Hebius sp3	AB989298	AB989300		AB989301	LC047780		LC047773
Hebius sp4	OK315827	OK315880	OK326970	OK315937	OK315993	OK316054	OK316113
Hebius taronensis	OK315828		OK326971	OK315938		OK316055	OK316114
Hebius venningi 1	KJ685675			KJ685625	KJ685727		
Hebius venningi 2	KJ685670			KJ685619	KJ685720		KJ685563
Hebius venningi 3	OK315829	OK315881	OK326972	OK315939	OK315994	OK316056	OK316115
Hebius vibakari	KJ685677			KJ685627	KJ685729		KJ685568
Hebius yanbianensis	MH532291						
Helophis schoutedeni		MW711523	MW699968	MW711538	MW711454	MW711561	MW711592
Herpetoreas burbrinki	GQ281781	JQ687418		JQ687443	KJ685761		KJ685599
Herpetoreas pealii	MT571586		MT578066	MT571590		MT571588	
Herpetoreas platyceps	KJ685690			KJ685640	KJ685742		KJ685582
Herpetoreas sp	OK315830	OK315882	OK326973	OK315940	OK315996	OK316058	OK316116
Herpetoreas xenura	MN993891			MN993889	OK315995	OK316057	MN993881
Hydraethiops melanogaster		MW711525	MW699972	MW711542	MW711458	MW711565	MW711594
Limnophis bangweolicus	MT587279	MT587306	MT586815	MT587292	MW711461	MW711568	MW711597
Limnophis bicolor	MT587281	MT587307	MT586817	MT587294	MW711462	MW711569	MW711598
Limnophis branchi	MT587284	MT587309	MT586819	MT587297	MW711464	MW711571	MW711600
Liodytes alleni	KF258653	KF258636			KF234032	KF258602	
Liodytes pygae	KF258654	KF258637			KF234033	KF258603	
Liodytes rigida	KF258659	KF258642			KF234038	KF258608	
Lycogthophis seychellensis	MW711511	MW711534	MW700003	MW711554	MW711479	MW711585	MW711609
triciteres fuliginoides	MW711487	MW711528	MW699975	MW711545	MW711465	MW711572	MW711601
triciteres olivacea 1	MT587285	MT587311	MT586821	MT587299	MW711466	MW711573	
triciteres olivacea 2	MW711488	MW711529	MW699976	MW711546	MW711467	MW711574	MW711602
triciteres sylvatica	MW711491	MW711531	MW699979	MW711548	MW711469	MW711576	MW711604
triciteres sylvatica	MW711492	MW711532	MW699980	MW711549		MW711577	
trix astreptophora	AY866535	AY873714					
trix helvetica	AY866544	AY873710		AF471121			
trix maura	AF420077	AF420080					
trix trix	LL999946	LL999896					

trix tessellata	AY866531	AY873734					
Nerodia clarkii		KT884436			KT884208	KT884149	
Nerodia cyclopion	KF258649	KF258632			KF234028	KF258598	
Nerodia erythrogaster	AF402912	KT884443		JN090137	KT884211	KT884157	
Nerodia florida		KT884425			KT884197	KT884136	
Nerodia harteri	JQ743511						
Nerodia sipedon	KX694873		KX694654	KX694812	KX695033	KX694741	
Nerodia taxispilota	AF402914						
Opisthotropis andersonii	KY594735						
Opisthotropis daovantieni	OK315831		OK326974	OK315941	OK315997	OK316059	OK316117
Opisthotropis durandi	MK941137						
Opisthotropis guangxiensis	GQ281776	JQ687422		JQ687447			
Opisthotropis haihaensis	MN890017						
Opisthotropis hungtai	MN890018						
Opisthotropis jacobi	MG545602						
Opisthotropis kuatunensis	KY594745						
Opisthotropis lateralis	OK315832	OK315883	OK326975	OK315942	OK315998	OK316060	OK316118
Opisthotropis latouchii	GQ281783	JQ687421		JQ687446			
Opisthotropis laui	KY594738						
Opisthotropis maxwelli	KY594736						
Opisthotropis shenzhenensis	KY594729						
Opisthotropis typica	LC325343			LC325789			LC326028
Opisthotropis voquyi	OK315833	OK315884	OK326976	OK315943	OK315999	OK316061	OK316119
Pseudagkistrodon rudis	KX694875		KX694651		KX695027	KX694735	
Regi grahami	KF258650	KF258633			KF234029	KF258599	
Regi septemvittata	KF258646	KF258629			KF234025	KF258595	
Rhabdophis adleri	KF800931			KF800921			
Rhabdophis auriculatus		OK315885	OK326977	OK315944	OK316000	OK316062	OK316120
Rhabdophis callichromus	LC325325			LC325771			LC326020
Rhabdophis chrysargos 1	HG763866						
Rhabdophis chrysargos 2	OK315834	OK315886	OK326978	OK315945	OK316001	OK316063	OK316121
Rhabdophis chrysargos 3	OK315835	OK315887	OK326979		OK316002	OK316064	OK316122

Rhabdophis conspicillatus	LC325342			LC325788			LC326027
Rhabdophis flaviceps	LC325355			LC325801			
Rhabdophis formosanus	LC325304			LC325750			
Rhabdophis guangdongensis	KF800930			KF800920			
Rhabdophis himalayanus	OK315836	OK315888	OK326981	OK315947	OK316004	OK316066	OK316124
Rhabdophis leordi	OK315837	OK315889	OK326982	OK315948	OK316005	OK316067	OK316125
Rhabdophis murudensis	LC325341			LC325787			
Rhabdophis nigrocinctus	OK315838	OK315890	OK326983	OK315949	OK316006	OK316068	OK316126
Rhabdophis nuchalis 1	KF800935			KF800925			
Rhabdophis nuchalis 2	OK315839	OK315891	OK326984	OK315950	OK316007	OK316069	OK316127
Rhabdophis nuchalis 3			OK326985	OK315951	OK316008	OK316070	OK316128
Rhabdophis pentasupralabialis	OK315840	OK315892	OK326986	OK315952	OK316009	OK316071	OK316129
Rhabdophis plumbicolor	OK315841	OK315893	OK326987	OK315953	OK316010	OK316072	OK316130
Rhabdophis subminiatus 1	LC325328			LC325774			
Rhabdophis subminiatus 2	OK315842	OK315895	OK326989	OK315955	OK316012	OK316074	OK316132
Rhabdophis swinhonis	LC325334			LC325780			LC326023
Rhabdophis tigrinus 1	LC325305			LC325751			LC326013
Rhabdophis tigrinus 2	GQ281785	JQ687419		JQ687444			
Rhabdops aquaticus	MF352835	MF352840	MF352830	MF352833			
Rhabdops olivaceous	MF352838	MF352842	MF352831	MF352834	MW711472	MW711580	MW711607
Rhabophis ceylonensis	KC347474	KC347520	KC347344	KC347384			LC326026
Sahyadriophis beddomei	OK315814		OK326957	OK315924	OK315982	OK316043	OK316102
Sahyadriophis uttaraghati	OR393306			OR397259	OR397260		OR397261
Smithophis atemporalis	MK350262	MK350258	MK350255	MK350265	MW711473	MW711581	MK350257
Smithophis bicolor	MK350261	MK350259	MK350254	MK350264	MW711474	MW711582	MK350256
Smithophis linearis	MT185677						
Storeria dekayi	KX694874		KX694669	KX694821			
Storeria storerioides	KF258651	KF258634			KF234030	KF258600	
Thamnophis atratus		KT884437			KT884209	KT884150	
Thamnophis brachystoma	AF420089	AF420092			KT884191	KT884131	
Thamnophis butleri	KF258645	KF258628			KF234024	KF258594	
Thamnophis chrysocephalus		AF420098			KT884187	KT884127	

Thamnophis couchii	AF420103	AF420106					
Thamnophis cyrtopsis	AF420099	AF420102					
Thamnophis elegans		KT884424			KT884196	KT884135	
Thamnophis eques		KT884429			KT884202	KT884141	
Thamnophis exsul	OK315843	OK315896	OK326990	OK315956	OK316013	OK316075	OK316133
Thamnophis fulvus		KT884420			KT884190	KT884130	
Thamnophis gigas	AF420133	AF420134					
Thamnophis godmani	AF420135	AF420138		AF471165			
Thamnophis hammondii	AF420139	AF420142					
Thamnophis marcianus		KT884418			KT884188	KT884128	
Thamnophis melanogaster		KT884423			KT884195	KT884134	
Thamnophis nigronuchalis	AF420153	AF420156					
Thamnophis ordinoides	AF402927	AF420160					
Thamnophis proximus	KF258658	KF258641			KF234037	KF258607	
Thamnophis pulchrilatus		KT884421			KT884193	KT884132	
Thamnophis rufipunctatus	AF420173	AF420176					
Thamnophis saurita		KT884434			KT884206	KT884146	
Thamnophis scalaris	OK315844		OK326991	OK315957	OK316014	OK316076	OK316134
Thamnophis scaliger	OK315845	OK315897	OK326992	OK315958			
Thamnophis sirtalis			KX694670		KX695054	KX694767	
Thamnophis sumichrasti	AF420197	AF420200					
Thamnophis validus	EF417409	EF417361					
Trachischium apteii	MN656975		MN385432				MN393094
Trachischium fuscum			MT003016	MN993886	OK316015	OK316077	MN993883
Trachischium guentheri	MN993892		MT003017	MN993887	OK316016	OK316078	MN993884
Trachischium lavea			MT003018	MN993888			MN993885
Trachischium monticola	JQ687435	JQ687428		JQ687453			
Trachischium tenuiceps	MN656977		MN385435				
Trimerodytes aequifasciata	JQ687430	JQ687415		JQ687440			
Trimerodytes annularis	JQ687431	JQ687424		JQ687449			
Trimerodytes balteata	MN017774						
Trimerodytes percarita	OK315846	OK315898	OK326993	OK315959	OK316017	OK316079	OK316135

Trimerodytes praemaxillaris	OK315847	OK315899	OK326994	OK315960	OK316018	OK316080	OK316136
Trimerodytes yapingi	MK621916						
Tropidoclonion lineatum	OK315848	OK315900	OK326995	OK315961	KF234034	KF258604	
Tropidonophis aenigmaticus	OK315849	OK315901	OK326996	OK315962	OK316019	OK316081	OK316137
Tropidonophis dahlii	OK315850	OK315902	OK326997	OK315963	OK316020	OK316082	OK316138
Tropidonophis dolasii	OK315851	OK315903	OK326998	OK315964	OK316021	OK316083	OK316139
Tropidonophis doriae	OK315852	OK315904	OK326999	OK315965	OK316022	OK316084	OK316140
Tropidonophis mairii	OK315853	OK315905	OK327000	OK315966	OK316023	OK316085	
Tropidonophis multiscutellatus	OK315854	OK315906	OK327001				
Tropidonophis picturatus	OK315856	OK315908	OK327003	OK315967	OK316024	OK316086	OK316141
Tropidonophis spilogaster		OK315894	OK326988	OK315954	OK316011	OK316073	OK316131
Tropidonophis statisticus 1	OK315857	OK315909	OK327004	OK315968	OK316025	OK316087	OK316142
Tropidonophis statisticus 2	OK315858	OK315910	OK327005				
Virginia valeriae	KR814700	KR814721	KR814654	KR814673			
Xenochrophis bellula	OK315859	OK315911	OK327006	OK315969	OK316026	OK316088	OK316143
Xenochrophis cerasogaster	OK315860		OK327007		OK316027	OK316089	
Xenochrophis maculatus	OK315861	OK315912	OK327008	OK315970	OK316028	OK316090	OK316144
Xenochrophis trianguligerus 1	LC325344			LC325790			LC326029
Xenochrophis trianguligerus 2	OK315862	OK315913	OK327009	OK315971	OK316029	OK316091	OK316145
Xenochrophis vittatus	EF395895		EF395846	EF395920			LC326019
Sibynophis subpunctatus	KC347471	KC347516	KX694665	KC347411			
Grayia orta	AY612048	AF544663	AY611866	AF544684	KX695019	FJ434002	

Table S7. Species and GenBank accession numbers used for phylogeny reconstruction of Pareidae (Reptilia, Squamata).

Species	Cyt-*b*	ND4	c-mos	Rag1
Aplopeltura boa	JF827673	JF827650	JF827696	
Asthenodipsas borneensis	KX660469	KX660597	KX660336	
Asthenodipsas laevis	KX660468	KX660596	KX660335	
Asthenodipsas lasgalenensis	KC916755	MZ712267	MZ712299	MZ712342
Asthenodipsas tropidonota	AY425808			
Asthenodipsas vertebralis	KC916750	MZ712268	MZ712300	MZ712343
Pareas abros	MZ712235	MZ712262	MZ712293	MZ712337
Pareas andersonii	MT968772	MW287040	MW287022	MZ712303
Pareas atayal	KJ642124	MW287041		
Pareas berdmorei berdmorei	MZ712218	MZ712244	MZ712276	MZ712319
Pareas berdmorei truongsonicus	MZ712221	MZ712247	MZ712288	MZ712323
Pareas berdmorei unicolor	MZ712223	MZ712249	MZ712279	MZ712324
Pareas boulengeri	JF827678	JF827656	MK135141	MK805409
Pareas caritus caritus	KC916748	MZ712261	MZ712292	MZ712336
Pareas caritus tesserimicus	MZ712233	MZ712259	MZ712291	MZ712334
Pareas chinensis	JF827691	JF827669	MK135137	MK805405
Pareas formosensis 1	MW287060	MW287042	MW287023	MZ712314
Pareas gemitus 1	MW287074	MW287050	MW287031	MZ712310
Pareas gemitus 2	MW287073	MW287049	MW287030	MZ712311
Pareas hamptoni	MK135077	MK805342	MK135126	MK805394
Pareas iwasakii	MZ712216		MZ712270	
Pareas kaduri	MW026190			
Pareas komaii	KJ642185	MW287056	KJ642210	
Pareas kuznetsovorum	MZ712232	MZ712258	MZ712296	MZ712335
Pareas macularius	MT968770	MZ712241	MZ712271	MZ712306
Pareas margaritophorus	MZ712217	MZ712243	MZ712273	MZ712305
Pareas menglaensis	MK135113	MK805378	MK135162	MK805430
Pareas mengziensis	MK135079	MK805348	MK135132	MK805400
Pareas modestus	MT968773			
Pareas monticola	MK135109	MK805374	MK135158	MK805424
Pareas nigriceps	MK201455			
Pareas nuchalis		U49311		
Pareas stanleyi	JN230704	JN230705	MK135135	MK805403
Pareas temporalis	MZ712239	MZ712266	MZ712298	MZ712341
Pareas victorianus	MW438300	MW438302	MW438308	MZ712301
Pareas vindumi	MW287080	MW287059	MW287039	MZ712308
Pareas xuelinensis	MW436709			
Xylophis captaini	MK340914	MK340912	MK344195	
Xylophis perroteti	MN970042	MN970046	MN970049	

Table S8. Species and GenBank accession numbers used for phylogeny reconstruction of *Gloydius* (Reptilia, Squamata, Viperidae).

Species	cytb	nd4
Gloydius angusticeps	KY040631	KY040652
Gloydius blomhoffii	AY352751	AY352814
Gloydius brevicaudus	JQ687486	JQ687467
Gloydius caraganus	MF490455	MF490453
Gloydius caucasicus	MZ959166	MZ959159
Gloydius chambensis	OP480163	OP407969
Gloydius changdaoensis	KX063823	KX063796
Gloydius cognatus	KY040619	KY040640
Gloydius halys	KY040618	KY040639
Gloydius himalayanus	MZ959173	MZ959172
Gloydius huangi	MZ355578	MW732035
Gloydius intermedius	KY040617	KY040638
Gloydius lateralis	ON423417	ON423421
Gloydius lipipengi	KY040628	KY040649
Gloydius liupanensis	JQ687491	JQ687472
Gloydius monticola	KY040635	MG025935
Gloydius qinlingensis	JQ687490	JQ687471
Gloydius rickmersi		KM078592
Gloydius rubromaculatus	KY040632	KY040653
Gloydius shedaoensis	KX063819	KX063792
Gloydius stejnegeri	KX063818	KX063791
Gloydius strauchi	KY040629	KY040650
Gloydius swild	JQ687487	JQ687468
Gloydius tsushimaensis	JN870203	JN870211
Gloydius ussuriensis	JQ687503	JQ687484
Gloydius variegatus	OK239651	OK239646
Ovophis okinavensis	D84256	U41895
Trimeresurus gracilis	DQ305461	AY352823

Table S9. Species and GenBank accession numbers used for phylogeny reconstruction of *Diploderma* (Reptilia, Squamata, Agamidae).

Species	*C-mos*	*BDNF*	*R35*	*ND2*
Diploderma angustelinea	MT659049	MT578009	MT659093	MT577930
Diploderma aorun	MT659052	MT577964	MT659096	MT577938
Diploderma batangense		MT577991		MT577935
Diploderma bowoense				MW506020
Diploderma brevicauda				MW506023
Diploderma brevipes	MK001506	MK001540	MK001464	MK001429
Diploderma chapaense	MK001494	MK001526	MK001474	MG214263
Diploderma daochengense				OP595621
Diploderma drukdaypo		MT578013		MT577950
Diploderma dymondi	MK001513	MK001545	MK001477	MK001422
Diploderma fasciatum				OM055801
Diploderma flaviceps	MT659060	MT577969	MT659105	MT577894
Diploderma flavilabre	MT659037	MT578002	MT659079	MT577916
Diploderma formosgulae				MW506024
Diploderma iadinum	MT659034	MT577987	MT659076	MT577956
Diploderma kangdingense				OP595627
Diploderma laeviventre	MT659031	MT577965	MT659073	MT577892
Diploderma limingense				OP428781
Diploderma luei	MK001510	MK001532	MK001462	MK001433
Diploderma makii	MK001508	MK001536	MK001460	MK001431
Diploderma menghaiense				MT598658
Diploderma micangshanense		MT577984	MT659110	MK578665
Diploderma panchi	MT659056	MT577980	MT659100	MT577946
Diploderma panlong				MT577906
Diploderma polygonatum	MK001515	MK001538	MK001458	MK001427
Diploderma qilin		MT577996		MT577942
Diploderma shuoquense				OP428773
Diploderma slowinskii	MT659027	MT577958	MT659069	MT577910
Diploderma splendidum	MK001492	MK001522	MK001476	MK001418
Diploderma swild	MT659065	MT577973	MT659112	MN266298
Diploderma swinhonis	MK001495	MK001534	MK001456	MK001419
Diploderma varcoae	MT659068	MT577962	MT659116	MT577902
Diploderma vela	MK001497	MK001531	MK001449	MK001415
Diploderma xinlongense				OP595613
Diploderma yangi				OL449603
Diploderma yongshengense				OP428777
Diploderma yulongense	MT659043	MT577978	MT659085	MT577922
Diploderma yunnanense	MK001517	MK001527	MK001469	MK001408
Diploderma zhaoermii	MK001500	MK001547	MK001467	MK001425
Pseudocalotes kakhiensis	MK001489	MK001549	MK001447	MK001435
Pseudocalotes kingdonwardi	MK001503	MK001550	MK001453	MK001436

Table S10. Species and GenBank accession numbers used for phylogeny reconstruction of *Scincella* (Reptilia, Squamata, Scincidae).

Species	16S	12S	COI	Cyt *b*	RAG1
Scincella_chengduensis	PQ466921	PQ466924	PQ467109	PQ481143	PQ493651
Scincella_potanini	OP935937	OP942203	OP942210	OQ448540	PP849368
Sphenomorphus_cryptotis	OP942190	OP942206	OP942215	OQ448544	
Scincella_assata	KU 289795	JF498074	JF497946		
Scincella_badenensis			MK990602		
Scincella_baraensis			MT742256		
Scincella_boettgeri			LC630768	AB818747	
Scincella_cherriei	MW265932				
Scincella_doriae			MH119616		
Scincella_dunan			LC630778	AB818791	
Scincella_formosensis			LC630790	AB818818	
Scincella_gemmingeri	AY308294	AY308445			
Scincella_huanrenensis	NC030779	NC030780	NC030781	NC030782	
Scincella_lateralis	JF498077	JF497948			
Scincella_melanosticta			MH119622		
Scincella_nigrofasciata			MK990605		
Scincella_reevesii	NC054206	NC054207	NC054208		
Scincella_rufocaudata	HM773217				
Scincella_rupicola	AB057403	AB057388			
Scincella_vandenburghi	KU646826	KU646826	KU646826	KU646826	
Scincella_modesta		PP819197	PP819215	PP849366	PP849370
Scincella_liangshanensis	PP826315	PP826317	PP824806	PP849364	PP849372
Scincella_monticola	PP819195	PP819198	PP819217		
Scincella_wangyuezhaoi	OP941172	OP942191	OQ402205		

Table S11. Species and GenBank accession numbers used for phylogeny reconstruction of *Ochotona* (Mammalia, Lagomorpha, Ochotonidae).

Species	*RAG1*	*RAG2*	*OXA1L*	*TTN*	*IL1RAPL1*	*COI*	*Cytb*
Ochotona alpina	KY025147	KY025241	KY025072	KY025332	KY024980	KY024790	KY024886
Ochotona argentata						KT317752	
Ochotona cansus	KY025149	KY025243	KY025073	KY025334	KY024982	KY024792	KY024888
Ochotona collaris						AF348080	AF348080
Ochotona coreana						MT017929	MT017929
Ochotona curzoniae	KY025164	KY025258	KY025087	KY025349	KY024998	KY024809	KY024905
Ochotona dauurica						MK105852	MK105852
Ochotona erythrotis	KY025179	KY025273	KY025094	KY025364	KY025012	KY024824	KY024920
Ochotona flatcalvariam						MN547437	MN547437
Ochotona forresti	KY025172	KY025266	KY025092	KY025357	KY025006	KY024817	KY024913
Ochotona hoffmanni							JF911804
Ochotona huanglongensis							KU359705
Ochotona hyperborea	KY025181	KY025275	KY025096	KY025366	KY025014	KY024826	KY024922
Ochotona iliensis							AY191824
Ochotona koslowi						MG888668	MG888668
Ochotona ladacensis							JX682569
Ochotona macrotis						MN547451	MN547451
Ochotona mantchurica						DQ347451	EF567058
Ochotona nubrica						OP006094	OP006094
Ochotona opaca						KT317755	
Ochotona pallasii						MN547457	MN547457
Ochotona princeps	KY025188	KY025282	KY025103	KY025373	KY025021	KY024833	KY024929
Ochotona pusilla						MN547458	MN547458
Ochotona qionglaiensis							KU359535
Ochotona roylii	KY025192			KY025376	KY025023	KY024837	KY024932
Ochotona rufescens	KY025193	KY025284	KY025105	KY025377	KY025024	KY024838	KY024933
Ochotona rutila					MH361541	MH361537	JX682566
Ochotona sacraria						MN547465	MN547465
Ochotona sikimaria						MN547468	MN547468
Ochotona syrinx	KY025234	KY025325	KY025142	KY025418	KY025065	KY024879	KY024973
Ochotona thibetana	KY025239	KY025330	KY025145	KY025423	KY025070	KY024884	KY024978

Ochotona thomasi	MH361582	MH361505	
Ochotona turuchanensis			MT513191
Ochotona vizier			OP227091

Table S12. Species and GenBank accession numbers used for phylogeny reconstruction of Spalacidae (Mammalia, Rodentia).

**Species**	**12S rRNA**	**16S rRNA**	***COI***	***Cytb***	***ND4***
Eospalax baileyi	NC_018098	NC_018098	NC_018098	NC_018098	NC_018098
Eospalax cansus	NC_021129	NC_021129	NC_021129	NC_021129	NC_021129
Eospalax fontanierii	OM329962	OM329962	OM329962	OM329962	OM329962
Eospalax muliensis	OM329993	OM329993	OM329993	OM329993	OM329993
Eospalax rothschildi	NC_018535	NC_018535	NC_018535	NC_018535	NC_018535
Eospalax rufescens	OM329957	OM329957	OM329957	OM329957	OM329957
Eospalax smithii	OM329982	OM329982	OM329982	OM329982	OM329982
Myospalax aspalax	NC_026915	NC_026915	NC_026915	NC_026915	NC_026915
Myospalax psilurus	NC_026034	NC_026034	NC_026034	NC_026034	NC_026034
Cannomys badius			MH189134	KY753961	
Rhizomys pruinosus	NC_021478	NC_021478	NC_021478	NC_021478	NC_021478
Rhizomys sinensis	NC_026124	NC_026124	NC_026124	NC_026124	NC_026124
Rhizomys sumatrensis	NC_039104	NC_039104	NC_039104	NC_039104	NC_039104
Tachyoryctes macrocephalus	MW751806	MW751806	MW751806	MW751806	MW751806
Tachyoryctes splendens			MH189129	AF160602	
Nannospalax ehrenbergi	NC_005315	NC_005315	NC_005315	NC_005315	NC_005315
Nannospalax leucodon	HQ652111	HQ652155		MH300079	
Nannospalax xanthodon	HQ652139	HQ652152		MH300127	
Spalax antiquus				KF021263	
Spalax arenarius	HQ652149	HQ652193			
Spalax graecus	HQ652148	HQ652191		JX455995	
Spalax microphthalmus	HQ652127	HQ652172			
Spalax zemni	HQ652150	HQ652194			

Table S13. Species and GenBank accession numbers used for phylogeny reconstruction of *Niviventer* (Mammalia, Rodentia, Muridae).

**Species**	***IRBP***	***Cytb***	***COI***	**D-loop**
Niviventer_andersoni	KF769343	KF740115	KF739984	KF739913
Niviventer_brahma		EF053011		
Niviventer_bukit		FJ665459		
Niviventer_confucianus	KF769392	KF740299	KF739935	KF739851
Niviventer_coninga	KY068458	KY068787		
Niviventer_cremoriventer	KC953417	KJ607284		
Niviventer_culturatus		EF053027		
Niviventer_eha	KF769332	KF740082	KF739990	KF739886
Niviventer_excelsior	KY068520	KY068849		
Niviventer_fengi	MN814697	MN814522	MN814604	
Niviventer_fulvescens	KF769369	KF740226	KF739981	KF739912
Niviventer_gladiusmaculus	MF642636	MF642357	MF642481	MF642558
Niviventer_hinpoon		KC010093	KC010232	---
Niviventer_huang	KF769380	KF740284	KF739972	KF739907
Niviventer_lepturus	MN273004			
Niviventer_lotipes	KF769358	KF740155	KF739947	KF739881
Niviventer_mekongis	MN814732	MN814574	MN814631	MN814684
Niviventer_niviventer	KF769373	KF740277	KF739956	KF739868
Niviventer_pianmaensis	MF642684	MF642436	MF642528	MF642606
Niviventer_rapit		DQ191483		
Niviventer_sacer	KY068475	KY068804		
Niviventer tenaster		GU457014		

Table S14. Species and GenBank accession numbers used for phylogeny reconstruction of Talpidae (Mammalia, Soricomorpha, Talpidae).

**Species**	***ADORA3***	***ADRB2***	***APOB***	***APP***	***ATP7A***	***BCHE***	***BDNF***	***BMI1***	***BRCA1***	***BRCA2***
Condylura cristata	KX754611	KX754938	KX755142	KX754760	KX754797	KX754827	KX754642	KX754673	KX754516	KX755195
Alpiscaptulus medogensis			MT349297						MT349299	
Parascalops breweri	KX754619	KX754964	KX755167	KX754771	KX754805	KX754833	KX754650	KX754680	KX754529	KX755219
Scalopus aquaticus	KX754621	KX754966	KX755169	KX754773	KX754807		KX754652	KX754682	KX754531	KX755221
Scapanulus oweni	KX754636	KX754982	KX755184	KX754788	KX754822	KX754847	KX754667	KX754697	KX754545	KX755236
Scapanus anthonyi										
Scapanus latimanus	KX754622	KX754967	KX755170	KX754774	KX754808	KX754835	KX754653	KX754683	KX754532	KX755222
Scapanus occultus										
Scapanus orarius	KX754625	KX754970	KX755173	KX754777	KX754811		KX754656	KX754686		
Scapanus townsendii	KX754627	KX754972	KX755175	KX754779	KX754813	KX754839	KX754658	KX754688	KX754536	KX755226
Desmana moschata	KX754613	KX754940	KX755143	KX754762	KX754799	KX754828	KX754644	KX754675	KX754518	KX755196
Galemys pyrenaicus	KX754615	KX754948	KX755151	KX754766	KX754801	KX754829		KX754676	KX754522	
Neurotrichus gibbsii	KX754618	KX754957	KX755160	KX754770	KX754804	KX754832	KX754649	KX754679	KX754528	KX755212
Scaptonyx fusicauda	KX754630	KX754976	KX755179	KX754783	KX754816	KX754842	KX754661	KX754691	KX754539	KX755230
Euroscaptor klossi										
Euroscaptor kuznetsovi									KX164219	KX164237
Euroscaptor longirostris	HG737951	KX754945	KX755148	HG738052	HG738071	HG738091	HG737972	HG737993	HG737899	KX755201
Euroscaptor malayanus		KX754942	KX755145	KX754764					KX754520	KX755198
Euroscaptor orlovi										KX164238
Euroscaptor parvidens		KX754944	KX755147	KX754765					KX754521	KX755200
Euroscaptor subanura									KX164226	KX164246
Mogera hainana										
Mogera imaizumii	HG737954	KX754949	KX755152	HG738050	HG738067	HG738088	HG737969	HG737989	KX754523	KX755204
Mogera insularis		KX754950	KX755153						KX754524	KX755205
Mogera kanoana										
Mogera latouchei									KP717202	KP717145

Mogera robusta		MK168934				MK168959			MK168838	MK168861
Mogera tokudae										
Mogera wogura	HG737957	KX754952	KX755155	HG738057	HG738076	HG738096	HG737977	HG737998	HG737903	KX755207
Oreoscaptor mizura	HG737949	KX754943	KX755146	HG738051	HG738069	HG738089	KX754646	HG737991	HG737898	KX755199
Parascaptor leucurus	HG737961	KX754959	KX755162	HG738060	HG738080	HG738100	HG737981	HG738002	HG737906	KX755214
Scaptochirus moschatus	HG737966	KX754973	KX755176	KX754780	HG738085	HG738105	HG737986	HG738007	HG737911	KX755227
Talpa altaica	HG737967	KX754983	KX755185	HG738065	HG738086	HG738106	HG737987	HG738008	HG737912	KX755237
Talpa aquitania										
Talpa caeca									KP717168	KP717114
Talpa caucasica									KP717186	KP717124
Talpa davidiana									KP717193	KP717134
Talpa europaea		KX754984	KX755186							KX755238
Talpa levantis									KP717179	KP717121
Talpa martinorum										
Talpa occidentalis									KP717162	KP717108
Talpa ognevi										
Talpa romana									KP717164	KP717110
Talpa stankovici									KP717181	KP717123
Dymecodon pilirostris	KX754614	KX754941	KX755144	KX754763	KX754800		KX754645		KX754519	KX755197
Urotrichus talpoides	KX754640	KX754985	KX755193	KX754795	KX754826	KX754854	KX754671	KX754701	KX754552	KX755245
Uropsilus aequodonenia										MH209906
Uropsilus andersoni										MH209871
Uropsilus atronates										MH209884
Uropsilus dabieshanensis					MT710700					
Uropsilus gracilis										MH209853
Uropsilus investigator										MH209830
Uropsilus nivatus	KX754637		KX755187	KX754789	KX754823	KX754848	KX754668	KX754698	KX754546	MH209807
Uropsilus soricipes	KX754639		KX755191	KX754793	KX754825	KX754852	KX754670	KX754700	KX754550	KX755243

Table S14. continued

Species	*CREM*	*DMP1*	*ENAM*	*GHR*	*PLCB4*	*RAG1*	*RAG2*	*TTN*	*vWF*	12S rRNA	*Cytb*
Condylura_cristata	KX754703	KX754887	KX755091	KX755038	KX754733	KX754553	KX754581	KX754856	KX754986	KX754488	KX754467
Alpiscaptulus_medogensis							MT349301			MT349878	MT349295
Parascalops_breweri	KX754712	KX754911	KX755116	KX755064	KX754740	KX754560	KX754589	KX754863	KX755010	KX754496	AB076808
Scalopus_aquaticus	KX754714	KX754913	KX755118	KX755066	KX754742	KX754562	KX754591	KX754865	KX755012	KX754498	KX754471
Scapanulus_oweni	KX754728	KX754927	KX755132	KX755081	KX754755	KX754576	KX754605	KX754879	KX755027	KX754512	KX754483
Scapanus_anthonyi											MZ217151
Scapanus_latimanus	KX754715	KX754914	KX755119	KX755067	KX754743	KX754563	KX754592	KX754866	KX755013	KX754499	AB076813
Scapanus_occultus											MZ217142
Scapanus_orarius	KX754718		KX755122	KX755070	KX754746	KX754566			KX755016	KX754501	AB076816
Scapanus_townsendii	KX754720	KX754918		KX755072		KX754568	KX754596	KX754870	KX755018	KX754503	AB076820
Desmana_moschata	KX754705	KX754888	KX755092	KX755040	KX754734	KX754554	KX754583	KX754858	KX754987	KX754490	AB076836
Galemys_pyrenaicus	KX754708	KX754895	KX755100	KX755048	KX754736	KX754556	KX754585		KX754995	KX754492	
Neurotrichus_gibbsii	KX754711	KX754904	KX755109	KX755057	KX754739	KX754559	KX754588	KX754862		KX754495	AB076827
Scaptonyx_fusicauda	KX754723	KX754922	KX755127	KX755076	KX754750	KX754571	KX754599	KX754873	KX755022	KX754506	KX754477
Euroscaptor_klossi						AB823179				AB823142	AB823107
Euroscaptor_kuznetsovi						KX164277					KX164259
Euroscaptor_longirostris	HG738013	KX754892	KX755097	KX755045	HG738034	HG737914	HG737932	HG738112	KX754992	HG737884	HG737870

Euroscaptor_malayanus			KX755094	KX755042		AB185155			KX754989	AB185153	AB185151
Euroscaptor_orlovi											
Euroscaptor_parvidens	KX754707	KX754891	KX755096	KX755044		AB823193			KX754991	AB823156	AB823121
Euroscaptor_subanura						KX164285					KX164266
Mogera_hainana						MW504277				MW675335	MW504276
Mogera_imaizumii	HG738010	KX754896	KX755101	KX755049	HG738030		HG737928	HG738108	KX754996		
Mogera_insularis		KX754897	KX755102	KX755050		AB823158			KX754997	AB181640	AB181616
Mogera_kanoana						AB823159				AB181645	AB181621
Mogera_latouchei						KP717316					KP717377
Mogera_robusta		MK169082	MK168983	MK169008		MK168815		MK169032	MK169057		MK169179
Mogera_tokudae						AB106243				AB106235	AB037607
Mogera_wogura	HG738018	KX754899	KX755104	KX755052	HG738039	HG737918	HG737937	HG738117	KX754999	HG737888	HG737874
Oreoscaptor_mizura	HG738012	KX754890	KX755095	KX755043	HG738032		HG737930	HG738110	KX754990		AB076828
Parascaptor_leucurus	HG738022	KX754906	KX755111	KX755059	HG738043	HG737922	HG737940	HG738121	KX755005	HG737892	HG737878
Scaptochirus_moschatus	HG738027	KX754919	KX755124	KX755073	HG738048	HG737927	HG737945	HG738126	KX755019	HG737897	HG737883
Talpa_altaica	HG738028	KX754928	KX755133	KX755082		AB176542	HG737946	HG738127	KX755028		
Talpa_aquitania										NC_053257	NC_053257
Talpa_caeca						KP717280					KP717333
Talpa_caucasica						KP717299					KP717351

Talpa_davidiana						KP717307					KP717367
Talpa_europaea		KX754929	KX755134	KX755083		AB106246			KX755029		AB076829
Talpa_levantis						KP717292					KP717340
Talpa_martinorum											MH093593
Talpa_occidentalis						KP717273					KP717327
Talpa_ognevi											KP717360
Talpa_romana						KP717275					KP717330
Talpa_stankovici						KP717293					KP717348
Dymecodon_pilirostris	KX754706	KX754889	KX755093	KX755041	KX754735	KX754555	KX754584	KX754859	KX754988	KX754491	AB076830
Urotrichus_talpoides	KX754732	KX754936	KX755141	KX755090	KX754759		KX754609	KX754886	KX755036	AB106239	AB076833
Uropsilus_aequodonenia				MH210079	MH210194	MH210316	MH210425	MH210604			MH209767
Uropsilus_andersoni				MH210042	MH210173	MH210288	MH210413	MH210566			MH209738
Uropsilus_atronates				MH210056	MH210183	MH210298	MH210423	MH210580			MH209748
Uropsilus_dabieshanensis					MW321511	MT710698	MT710699				MT710697
Uropsilus_gracilis				MH210021	MH210163	MH210273	MH210398	MH210545			MH209723
Uropsilus_investigator				MH209980	MH210133	MH210245	MH210368	MH210500			MH209689
Uropsilus_nivatus	KX754729	KX754930	KX755135	MH209954	MH210113	MH210229	MH210351	MH210474	KX755030	KX754513	MH209670
Uropsilus_soricipes	KX754731	KX754934	KX755139	KX755088	KX754758	KX754579	KX754608	KX754884	KX755034	KX754515	KX754486

Table S15. Species and GenBank accession numbers used for phylogeny reconstruction of Arvicolinae (Mammalia, Rodentia, Cricetidae).

**Species**	***ATP 6***	***ATP 8***	***Cytb***	***ND1***	***ND2***	***ND3***
Arvicola amphibius	MN122834	MN122834	MN122834	MN122834	MN122834	MN122834
Arvicola persicus			MN165744			
Arvicola sapidus			JX457749			
Alticola argentatus	ON165386	ON165386	ON165386		ON165386	ON165386
Alticola barakshin			DQ845194			
Alticola lemminus	MT381922	MT381922	MT381922	MT381922	MT381922	MT381922
Alticola macrotis	MT381923	MT381923	MT381923	MT381923	MT381923	MT381923
Alticola olchonensis	MT381924	MT381924	MT381924	MT381924	MT381924	MT381924
Alticola semicanus			KJ556709			
Alticola stoliczkanus	ON165289	ON165289	ON165289	ON165289	ON165289	ON165289
Alticola strelzovi	MT381925	MT381925	MT381925	MT381925	MT381925	MT381925
Alticola tuvinicus	MT381926	MT381926	MT381926	MT381926	MT381926	MT381926
Caryomys eva	ON165322	ON165322	ON165322	ON165322	ON165322	ON165322
Caryomys inez	ON165282	ON165282	ON165282	ON165282	ON165282	ON165282
Clethrionomys californicus			KJ556723			
Clethrionomys centralis	MT381940	MT381940	MT381940	MT381940	MT381940	MT381940
Clethrionomys gapperi	OM963005	OM963005	OM963005	OM963005	OM963005	OM963005
Clethrionomys glareolus	KM892841	KM892841	KM892841	KM892841	KM892841	KM892841
Clethrionomys rutilus	MK482363	MK482363	MK482363	MK482363	MK482363	MK482363
Craseomys regulus	NC_016427	NC_016427	NC_016427	NC_016427	NC_016427	NC_016427
Craseomys rex						
Craseomys rufocanus	ON165320	ON165320	ON165320	ON165320	ON165320	ON165320
Eothenomys chinensis	FJ483847	FJ483847	FJ483847	FJ483847	FJ483847	FJ483847
Eothenomys custos	ON165280	ON165280	ON165280	ON165280	ON165280	ON165280
Eothenomys hintoni	ON165281	ON165281	ON165281	ON165281	ON165281	ON165281
Eothenomys olitor	ON165374	ON165374	ON165374	ON165374	ON165374	ON165374
Eothenomys proditor	ON165284	ON165284	ON165284	ON165284	ON165284	ON165284
Eothenomys tarquinius	ON165301	ON165301	ON165301	ON165301	ON165301	ON165301
Eothenomys wardi	ON165324	ON165324	ON165324	ON165324	ON165324	ON165324

Eothenomys colurnus	ON165370	ON165370	ON165370	ON165370	ON165370	ON165370
Eothenomys eleusis	ON165310	ON165310	ON165310	ON165310	ON165310	ON165310
Eothenomys melanogaster	NC_027418	NC_027418	NC_027418	NC_027418	NC_027418	NC_027418
Eothenomys shimianensis	ON165296	ON165296	ON165296	ON165296	ON165296	ON165296
Eothenomys luojishanensis	ON165327	ON165327	ON165327	ON165327	ON165327	ON165327
Eothenomys jinyangensis	ON165288	ON165288	ON165288	ON165288	ON165288	ON165288
Eothenomys meiguensis	ON165331	ON165331	ON165331	ON165331	ON165331	ON165331
Eothenomys miletus	NC_030330	NC_030330	NC_030330	NC_030330	NC_030330	NC_030330
Eothenomys cachinus	NC_081577	NC_081577	NC_081577	NC_081577	NC_081577	NC_081577
Eothenomys fidelis			KY997446			
Dicrostonyx groenlandicus	KX712239	KX712239	KX712239	KX712239	KX712239	KX712239
Dicrostonyx hudsonius	OL752780	OL752780	OL752780	OL752780	OL752780	OL752780
Dicrostonyx richardsoni						
Dicrostonyx torquatus	KX066190	KX066190	KX066190	KX066190	KX066190	KX066190
Bramus fuscocapillus	MT483991	MT483991	MT483991	MT483991	MT483991	MT483991
Bramus lutescens	MT483992	MT483992	MT483992	MT483992	MT483992	MT483992
Ellobius talpinus	NC_054160	NC_054160	NC_054160	NC_054160	NC_054160	NC_054160
Ellobius tancrei	ON165304	ON165304	ON165304	ON165304	ON165304	ON165304
Eolagurus luteus	MT492448	MT492448	MT492448	MT492448	MT492448	MT492448
Eolagurus przewalskii			MK750944			
Lagurus lagurus	MT492449	MT492449	MT492449	MT492449	MT492449	MT492449
Lemmus lemmus			MK895062			
Lemmus trimucronatus	MT381930	MT381930	MT381930	MT381930	MT381930	MT381930
Myopus schisticolor	MT381931	MT381931	MT381931		MT381931	MT381931
Synaptomys borealis			KY754161			
Synaptomys cooperi	MT492450	MT492450	MT492450	MT492450	MT492450	MT492450
Alexandromys evoronensis			MF099588			

Alexandromys fortis	ON165311	ON165311	ON165311	ON165311	ON165311	ON165311
Alexandromys kikuchii			MF099529			
Alexandromys limnophilus	ON165283	ON165283	ON165283			ON165283
Alexandromys maximowiczii	ON165383	ON165383	ON165383	ON165383	ON165383	ON165383
Alexandromys middendorffii			MF099537			
Alexandromys mongolicus			MK750920			
Alexandromys montebelli			MF099543			
Alexandromys mujanensis			MF099593			
Alexandromys oeconomus			MW598284			
Alexandromys sachalinensis			MF099547			
Alexandromys shantaricus			MT990637			
Chionomys gud	MT381928	MT381928	MT381928	MT381928	MT381928	MT381928
Chionomys roberti	OK323269	OK323269	OK323269	OK323269	OK323269	OK323269
Chionomys stekolnikovi			OL698789			
Hyperacrius fertilis	MT433094	MT433094	MT433094	MT433094	MT433094	MT433094
Lasiopodomys brandtii	MT614214	MT614214	MT614214	MT614214	MT614214	MT614214
Lasiopodomys mandarinus	MT614219	MT614219	MT614219	MT614219	MT614219	MT614219
Microtus abbreviatus			GU809089			
Microtus agrestis	MH152570	MH152570	MH152570	MH152570	MH152570	MH152570
Microtus anatolicus			FJ767740			
Microtus arvalis	MG948434	MG948434	MG948434	MG948434	MG948434	MG948434
Microtus brachycercus			KT896523			
Microtus bucharensis			AM392369			
Microtus cabrerae	MT381938	MT381938	MT381938	MT381938	MT381938	MT381938
Microtus californicus	MT381939	MT381939	MT381939	MT381939	MT381939	MT381939

Microtus canicaudus			AF163892			
Microtus chrotorrhinus	MN058078	MN058078	MN058078	MN058078	MN058078	MN058078
Microtus daghestanicus	MT381933	MT381933	MT381933	MT381933	MT381933	MT381933
Microtus dogramacii			AY513793			
Microtus drummondii			ON989673			
Microtus duodecimcostatus			JX457797			
Microtus felteni			FJ641180			
Microtus guatemalensis			AF410262			
Microtus guentheri	MT381935	MT381935	MT381935	MT381935	MT381935	MT381935
Microtus hartingi			ON324051			
Microtus ilaeus	MZ362410	MZ362410	MZ362410	MZ362410	MZ362410	MZ362410
Microtus irani			KC953618			
Microtus kermanensis			MG721550			
Microtus lavernedii			OQ885464			
Microtus liechtensteini			LT222303			
Microtus longicaudus	MT381942	MT381942	MT381942	MT381942	MT381942	MT381942
Microtus lusitanicus			JX457762			
Microtus majori			MZ198208			
Microtus mexicanus			AF163897			
Microtus mogollonensis			OK135031			
Microtus montanus	MW387163	MW387163	MW387163	MW387163	MW387163	MW387163
Microtus multiplex			LT222306			
Microtus mustersi			MK631994			
Microtus mystacinus			KX581061			
Microtus nebrodensis			KT896526			
Microtus oaxacensis			AF410260			
Microtus obscurus			MZ198209			
Microtus ochrogaster	KT166982	KT166982	KT166982	KT166982	KT166982	KT166982
Microtus oregoni			GU954308			
Microtus paradoxus			KC953624			

Microtus pennsylvanicus			KC473489			
Microtus pinetorum			AF163904			
Microtus pyrenaicus			LT222302			
Microtus quasiater			AF410259			
Microtus richardsoni	OM501078	OM501078	OM501078	OM501078	OM501078	OM501078
Microtus rossiaemeridionalis	DQ015676	DQ015676	DQ015676	DQ015676	DQ015676	DQ015676
Microtus savii			EU158779			
Microtus schelkovnikovi			LT222309			
Microtus schidlovskii			KJ739801			
Microtus socialis	MT381932	MT381932	MT381932	MT381932	MT381932	MT381932
Microtus subterraneus	MN326850	MN326850	MN326850	MN326850	MN326850	MN326850
Microtus tatricus			LT222298			
Microtus thomasi	MN058079	MN058079	MN058079	MN058079	MN058079	MN058079
Microtus townsendii			AF163906			
Microtus transcaspicus			KX581070			
Microtus umbrosus			AF410261			
Microtus xanthognathus			AF163907			
Mictomicrotus liangshanensis	FJ463038	FJ463038	FJ463038	FJ463038	FJ463038	FJ463038
Neodon clarkei	ON165352	ON165352	ON165352	ON165352	ON165352	ON165352
Neodon forresti	ON165367	ON165367	ON165367	ON165367	ON165367	ON165367
Neodon fuscus	ON165391	ON165391	ON165391	ON165391	ON165391	ON165391
Neodon irene	ON165323	ON165323	ON165323	ON165323	ON165323	ON165323
Neodon leucurus	ON165297	ON165297	ON165297	ON165297	ON165297	ON165297
Neodon linzhiensis	ON165279	ON165279	ON165279	ON165279	ON165279	ON165279
Neodon medogensis	ON165346	ON165346	ON165346	ON165346	ON165346	ON165346
Neodon nyalamensis	ON165379	ON165379	ON165379	ON165379	ON165379	ON165379
Neodon sikimensis	ON165360	ON165360	ON165360	ON165360	ON165360	ON165360
Proedromys bedfordi			KU577347			
Stenocranius gregalis			OP765480			

Stenocranius raddei			OP765406			
Volemys millicens	ON165377	ON165377	ON165377	ON165377	ON165377	ON165377
Volemys musseri	ON165362	ON165362	ON165362	ON165362	ON165362	ON165362
Neofiber alleni			AM910618			
Ondatra zibethicus	MT084805	MT084805	MT084805	MT084805	MT084805	MT084805
Arborimus albipes			AY338814			
Arborimus longicaudus			KY753947			
Arborimus pomo			AY338824			
Phenacomys intermedius	MT381941	MT381941	MT381941	MT381941		MT381941
Phenacomys ungava			OR350912			
Dinaromys bogdanovi	MT588182	MT588182	MT588182	MT588182	MT588182	MT588182
Prometheomys schaposchnikowi	MT118739	MT118739	MT118739	MT118739	MT118739	MT118739

Table S15. continued

**Species**	***ND4***	***ND5***	***ND6***	***COI***	***COII***	***COIII***
Arvicola amphibius	MN122834	MN122834	MN122834	MN122834	MN122834	MN122834
Arvicola persicus						
Arvicola sapidus						
Alticola argentatus	ON165386			ON165386	ON165386	ON165386
Alticola barakshin						
Alticola lemminus	MT381922	MT381922	MT381922	MT381922	MT381922	MT381922
Alticola macrotis	MT381923	MT381923	MT381923	MT381923	MT381923	MT381923
Alticola olchonensis	MT381924	MT381924	MT381924	MT381924	MT381924	MT381924
Alticola semicanus						
Alticola stoliczkanus	ON165289	ON165289	ON165289	ON165289	ON165289	ON165289
Alticola strelzovi	MT381925	MT381925	MT381925	MT381925	MT381925	MT381925
Alticola tuvinicus	MT381926	MT381926	MT381926	MT381926	MT381926	MT381926
Caryomys eva	ON165322	ON165322	ON165322	ON165322	ON165322	ON165322
Caryomys inez	ON165282	ON165282	ON165282	ON165282	ON165282	ON165282
Clethrionomys californicus						
Clethrionomys centralis	MT381940	MT381940	MT381940	MT381940	MT381940	MT381940
Clethrionomys gapperi	OM963005	OM963005	OM963005	OM963005	OM963005	OM963005

Clethrionomys glareolus	KM892841	KM892841	KM892841	KM892841	KM892841	KM892841
Clethrionomys rutilus	MK482363	MK482363	MK482363	MK482363	MK482363	MK482363
Craseomys regulus	NC_016427	NC_016427	NC_016427	NC_016427	NC_016427	NC_016427
Craseomys rex				JF693314		
Craseomys rufocanus	ON165320	ON165320	ON165320	ON165320	ON165320	ON165320
Eothenomys chinensis	FJ483847	FJ483847	FJ483847	FJ483847		FJ483847
Eothenomys custos	ON165280	ON165280	ON165280	ON165280	ON165280	ON165280
Eothenomys hintoni	ON165281	ON165281	ON165281	ON165281	ON165281	ON165281
Eothenomys olitor	ON165374	ON165374	ON165374	ON165374	ON165374	ON165374
Eothenomys proditor	ON165284	ON165284	ON165284	ON165284	ON165284	ON165284
Eothenomys tarquinius	ON165301	ON165301	ON165301	ON165301	ON165301	ON165301
Eothenomys wardi	ON165324	ON165324	ON165324	ON165324	ON165324	ON165324
Eothenomys colurnus	ON165370	ON165370	ON165370	ON165370	ON165370	ON165370
Eothenomys eleusis	ON165310	ON165310	ON165310	ON165310	ON165310	ON165310
Eothenomys melanogaster	NC_027418	NC_027418	NC_027418	NC_027418	NC_027418	NC_027418
Eothenomys shimianensis	ON165296	ON165296	ON165296	ON165296	ON165296	ON165296
Eothenomys luojishanensis	ON165327	ON165327	ON165327	ON165327	ON165327	ON165327
Eothenomys jinyangensis	ON165288	ON165288	ON165288	ON165288	ON165288	ON165288
Eothenomys meiguensis	ON165331	ON165331	ON165331	ON165331	ON165331	ON165331
Eothenomys miletus	NC_030330	NC_030330	NC_030330	NC_030330	NC_030330	NC_030330
Eothenomys cachinus	NC_081577	NC_081577	NC_081577	NC_081577	NC_081577	NC_081577
Eothenomys fidelis				KY997268		
Dicrostonyx groenlandicus	KX712239	KX712239	KX712239	KX712239	KX712239	KX712239
Dicrostonyx hudsonius	OL752780	OL752780	OL752780	OL752780	OL752780	OL752780
Dicrostonyx richardsoni				JF456589		
Dicrostonyx torquatus	KX066190	KX066190	KX066190	KX066190	KX066190	KX066190
Bramus fuscocapillus	MT483991	MT483991	MT483991	MT483991	MT483991	MT483991
Bramus lutescens	MT483992	MT483992	MT483992	MT483992	MT483992	MT483992
Ellobius talpinus	NC_054160	NC_054160	NC_054160	NC_054160	NC_054160	NC_054160
Ellobius tancrei	ON165304	ON165304	ON165304	ON165304	ON165304	ON165304
Eolagurus luteus	MT492448	MT492448	MT492448	MT492448	MT492448	MT492448
Eolagurus przewalskii						
Lagurus lagurus	MT492449	MT492449	MT492449	MT492449	MT492449	MT492449

Lemmus lemmus				MK895073		
Lemmus trimucronatus	MT381930	MT381930	MT381930	MT381930	MT381930	MT381930
Myopus schisticolor	MT381931	MT381931	MT381931	MT381931	MT381931	MT381931
Synaptomys borealis						
Synaptomys cooperi	MT492450	MT492450	MT492450	MT492450	MT492450	MT492450
Alexandromys evoronensis						
Alexandromys fortis	ON165311	ON165311	ON165311	ON165311	ON165311	ON165311
Alexandromys kikuchii						
Alexandromys limnophilus	ON165283	ON165283	ON165283	ON165283	ON165283	ON165283
Alexandromys maximowiczii	ON165383	ON165383	ON165383	ON165383	ON165383	ON165383
Alexandromys middendorffii						
Alexandromys mongolicus						
Alexandromys montebelli						
Alexandromys mujanensis						
Alexandromys oeconomus						
Alexandromys sachalinensis						
Alexandromys shantaricus						
Chionomys gud	MT381928	MT381928	MT381928	MT381928	MT381928	MT381928
Chionomys roberti	OK323269	OK323269	OK323269	OK323269	OK323269	OK323269
Chionomys stekolnikovi						
Hyperacrius fertilis	MT433094	MT433094	MT433094	MT433094	MT433094	MT433094
Lasiopodomys brandtii	MT614214	MT614214	MT614214	MT614214	MT614214	MT614214
Lasiopodomys mandarinus	MT614219	MT614219	MT614219	MT614219	MT614219	MT614219
Microtus abbreviatus						
Microtus agrestis	MH152570	MH152570	MH152570	MH152570	MH152570	MH152570
Microtus anatolicus						
Microtus arvalis	MG948434	MG948434	MG948434	MG948434	MG948434	MG948434
Microtus brachycercus						
Microtus bucharensis						
Microtus cabrerae	MT381938	MT381938	MT381938	MT381938	MT381938	MT381938
Microtus californicus	MT381939	MT381939	MT381939	MT381939	MT381939	MT381939

Microtus canicaudus						
Microtus chrotorrhinus	MN058078	MN058078	MN058078	MN058078	MN058078	MN058078
Microtus daghestanicus	MT381933	MT381933	MT381933	MT381933		MT381933
Microtus dogramacii						
Microtus drummondii						
Microtus duodecimcostatus						
Microtus felteni						
Microtus guatemalensis						
Microtus guentheri	MT381935	MT381935		MT381935	MT381935	MT381935
Microtus hartingi						
Microtus ilaeus	MZ362410	MZ362410	MZ362410	MZ362410	MZ362410	MZ362410
Microtus irani						
Microtus kermanensis						
Microtus lavernedii				OQ706755		
Microtus liechtensteini						
Microtus longicaudus	MT381942	MT381942	MT381942	MT381942	MT381942	MT381942
Microtus lusitanicus						
Microtus majori						
Microtus mexicanus						
Microtus mogollonensis						
Microtus montanus	MW387163	MW387163	MW387163	MW387163	MW387163	MW387163
Microtus multiplex						
Microtus mustersi						
Microtus mystacinus						
Microtus nebrodensis						
Microtus oaxacensis						
Microtus obscurus						
Microtus ochrogaster	KT166982	KT166982	KT166982	KT166982	KT166982	KT166982
Microtus oregoni						
Microtus paradoxus						
Microtus pennsylvanicus						
Microtus pinetorum						
Microtus pyrenaicus						

Microtus quasiater						
Microtus richardsoni	OM501078	OM501078	OM501078	OM501078	OM501078	OM501078
Microtus rossiaemeridionalis	DQ015676	DQ015676	DQ015676	DQ015676	DQ015676	DQ015676
Microtus savii						
Microtus schelkovnikovi						
Microtus schidlovskii						
Microtus socialis	MT381932	MT381932	MT381932	MT381932	MT381932	MT381932
Microtus subterraneus	MN326850	MN326850	MN326850	MN326850	MN326850	MN326850
Microtus tatricus						
Microtus thomasi	MN058079	MN058079	MN058079	MN058079	MN058079	MN058079
Microtus townsendii						
Microtus transcaspicus						
Microtus umbrosus						
Microtus xanthognathus						
Mictomicrotus liangshanensis	FJ463038	FJ463038	FJ463038	FJ463038	FJ463038	FJ463038
Neodon clarkei	ON165352	ON165352	ON165352	ON165352	ON165352	ON165352
Neodon forresti	ON165367	ON165367	ON165367	ON165367	ON165367	ON165367
Neodon fuscus	ON165391	ON165391	ON165391	ON165391	ON165391	ON165391
Neodon irene	ON165323	ON165323	ON165323	ON165323	ON165323	ON165323
Neodon leucurus	ON165297	ON165297	ON165297	ON165297	ON165297	ON165297
Neodon linzhiensis	ON165279	ON165279	ON165279	ON165279	ON165279	ON165279
Neodon medogensis	ON165346	ON165346	ON165346	ON165346	ON165346	ON165346
Neodon nyalamensis	ON165379	ON165379	ON165379	ON165379	ON165379	ON165379
Neodon sikimensis	ON165360	ON165360	ON165360	ON165360	ON165360	ON165360
Proedromys bedfordi				KU577348		
Stenocranius gregalis						
Stenocranius raddei						
Volemys millicens	ON165377	ON165377	ON165377	ON165377	ON165377	ON165377
Volemys musseri	ON165362	ON165362	ON165362	ON165362	ON165362	ON165362
Neofiber alleni						
Ondatra zibethicus	MT084805	MT084805	MT084805	MT084805	MT084805	MT084805
Arborimus albipes						

Arborimus longicaudus						
Arborimus pomo						
Phenacomys intermedius	MT381941	MT381941	MT381941	MT381941	MT381941	MT381941
Phenacomys ungava						
Dinaromys bogdanovi	MT588182	MT588182	MT588182	MT588182	MT588182	MT588182
Prometheomys schaposchnikowi	MT118739	MT118739	MT118739	MT118739	MT118739	MT118739

Table S16. Inferred times with 95% HPD of *in situ* diversification events of QTP and its subregions.

Number	Group	Median (Ma)	Lower (Ma)	Upper (Ma)	Region
1	Invertebrates	24.38	15.78	31.76	HDM
2	Invertebrates	14.76	7.47	22.55	HDM
3	Invertebrates	12.2	6.34	18.55	QTP
4	Invertebrates	8.21	3.14	13.73	QTP
5	Invertebrates	11.61	5.15	18.23	QTP
6	Invertebrates	25.86	20.23	32.79	HDM
7	Invertebrates	10.15	6.56	14.45	HDM
8	Invertebrates	16.88	11.38	23.24	HDM
9	Invertebrates	18.96	14.32	24.48	HDM
10	Invertebrates	15.73	10.89	21.63	HDM
11	Invertebrates	23.73	18.29	29.95	HDM
12	Invertebrates	16.13	12.08	20.86	HDM
13	Invertebrates	5.02	2.94	7.5	HDM
14	Invertebrates	12.01	8.84	15.67	HDM
15	Invertebrates	9.52	6.61	12.65	HDM
16	Invertebrates	6.18	3.83	8.88	HDM
17	Invertebrates	12.29	7.7	17.88	HDM
18	Invertebrates	27.34	20.86	34.45	HDM
19	Invertebrates	21.6	15.47	28.47	HDM
20	Invertebrates	6.39	3.79	9.71	HDM
21	Invertebrates	21.36	14.44	29.31	QTP
22	Invertebrates	3	1.46	5.18	HDM
23	Invertebrates	23.9	18.13	30.25	HDM
24	Invertebrates	18.8	13.76	24.24	HDM
25	Invertebrates	5.37	3.3	7.98	QTP
26	Invertebrates	6.19	4.14	8.67	HDM
27	Invertebrates	26.45	20.59	32.44	HDM
28	Invertebrates	21.42	15.47	27.68	HDM
29	Invertebrates	17.37	12.26	22.85	QTP
30	Invertebrates	22.97	17.66	28.63	HDM
31	Invertebrates	18.31	13.04	24.24	HDM
32	Invertebrates	24.05	18.56	30.03	HDM
33	Invertebrates	40.35	31.32	50.56	HDM
34	Invertebrates	33.17	24.12	43.83	HDM
35	Invertebrates	56.6	47.99	65.24	HDM
36	Invertebrates	38.99	28.51	49.52	HDM
37	Invertebrates	53.63	45.29	62.26	QTP
38	Invertebrates	9.67	6.08	13.91	HIM
39	Invertebrates	24.2	17.68	31.14	HIM
40	Invertebrates	25.2	19.4	31.76	HIM

41	Invertebrates	15.24	10.32	20.91	HIM
42	Invertebrates	3.69	2.08	5.63	HIM
43	Invertebrates	46.2	38.84	53.74	HIM
44	Invertebrates	33.84	26.74	41.36	QTP
45	Invertebrates	15.03	10.04	20.45	TP
46	Invertebrates	23.56	17.52	30.13	QTP
47	Invertebrates	80	60.03	101.55	QTP
48	Invertebrates	21.99	15.83	29.21	QTP
49	Invertebrates	10.83	5.91	15.96	QTP
50	Invertebrates	14.08	10.4	18.11	QTP
51	Invertebrates	9.53	6.26	13.31	HDM
52	Invertebrates	12.06	8.39	16.54	HDM
53	Invertebrates	10.15	6.53	15.7	QTP
54	Invertebrates	2.98	1.27	5.23	HDM
55	Invertebrates	3.04	1.55	5.09	HDM
56	Invertebrates	8.54	5.71	11.78	HDM
57	Invertebrates	15.04	11.62	18.35	HDM
58	Invertebrates	3.69	1.99	6.04	QTP
59	Invertebrates	11.92	8.49	15.41	HDM
60	Invertebrates	17.1	13.62	20.44	HDM
61	Invertebrates	15.82	12.46	19.21	QTP
62	Invertebrates	6.17	4.27	8.48	HDM
63	Invertebrates	13.28	9.99	16.51	HDM
64	Invertebrates	4.82	2.28	8.16	HDM
65	Invertebrates	19.19	16.08	22.54	QTP
66	Invertebrates	6.33	3.71	9.5	HIM
67	Invertebrates	12.51	9.01	15.94	HIM
68	Invertebrates	9.27	5.98	13.03	HIM
69	Invertebrates	17.26	13.69	21.15	HIM
70	Invertebrates	9.89	6.92	13.21	HIM
71	Invertebrates	7.87	5.18	11.16	HIM
72	Invertebrates	5.74	3.34	9.04	QTP
73	Invertebrates	22.02	18.78	25.48	HDM
74	Invertebrates	16.93	13.57	20.47	HDM
75	Invertebrates	3.32	1.72	5.42	HDM
76	Invertebrates	9.86	5.96	13.93	HDM
77	Invertebrates	18.86	15.54	22.34	HDM
78	Invertebrates	15.41	11.19	19.46	HDM
79	Invertebrates	10.11	6.73	13.88	HDM
80	Invertebrates	5.81	3.48	9.38	HDM
81	Invertebrates	21.56	18.51	24.8	HDM
82	Invertebrates	27.52	23.93	30.92	HDM
83	Invertebrates	7.75	3.85	11.82	HDM

84	Invertebrates	15.87	12.1	20.25	HDM
85	Invertebrates	20.06	16.62	23.37	HDM
86	Invertebrates	14.5	10.58	18.27	HDM
87	Invertebrates	12.57	8.39	16.61	HDM
88	Invertebrates	17.69	14.88	21.06	HDM
89	Invertebrates	13.61	10.14	16.8	HDM
90	Invertebrates	8.02	4.81	11.77	HDM
91	Invertebrates	15.88	12.93	18.91	HDM
92	Invertebrates	9.75	7.61	12.18	HDM
93	Invertebrates	7.68	4.48	10.87	HDM
94	Invertebrates	5.66	2.61	8.81	HDM
95	Invertebrates	11.12	8.8	13.83	QTP
96	Invertebrates	13.63	10.89	16.45	HDM
97	Invertebrates	9.84	6.73	13.33	HDM
98	Invertebrates	7.68	4.67	10.97	HDM
99	Invertebrates	23.11	19.62	26.61	HDM
100	Invertebrates	24.38	20.41	28.32	HIM
101	Invertebrates	9.11	3.5	14.31	HIM
102	Invertebrates	19.01	14.95	23.11	QTP
103	Invertebrates	22.77	18.75	26.8	HIM
104	Invertebrates	18.72	15.7	21.78	HDM
105	Invertebrates	3.28	1.63	5.37	HDM
106	Invertebrates	11.47	7.45	15.35	HDM
107	Invertebrates	17.48	14.66	20.58	HDM
108	Invertebrates	2.42	1.39	3.73	QTP
109	Invertebrates	4.22	2.32	6.14	HDM
110	Invertebrates	3.33	1.68	5	HDM
111	Invertebrates	13.19	10.02	16.9	HDM
112	Invertebrates	11.2	8.33	14.2	HDM
113	Invertebrates	6.76	4.5	9.26	HDM
114	Invertebrates	5.18	3.19	7.32	HDM
115	Invertebrates	3.82	1.93	5.89	HDM
116	Invertebrates	17.71	14.56	21.52	HDM
117	Invertebrates	3.71	1.72	6.15	TP
118	Invertebrates	9.35	5.53	13.19	HDM
119	Invertebrates	13.05	10.27	15.83	HDM
120	Invertebrates	7.32	4.25	10.32	HDM
121	Invertebrates	11.68	9.21	14.39	HDM
122	Invertebrates	3.47	1.85	5.38	HDM
123	Invertebrates	6.51	4.36	9.01	HDM
124	Invertebrates	10.95	8.63	13.62	HDM
125	Invertebrates	6.97	4.72	9.51	HDM
126	Invertebrates	3.71	2.36	5.49	HDM

127	Invertebrates	2.1	1.06	3.45	HDM
128	Invertebrates	23.77	20.5	27.27	HDM
129	Amphibians	18.45	15.41	24.76	HIM
130	Amphibians	12.03	9.37	15.48	HDM
131	Amphibians	4.03	2.61	5.09	HDM
132	Amphibians	1.16	0.85	1.78	HDM
133	Amphibians	4.75	3.54	6.29	QTP
134	Amphibians	5.4	4.19	6.84	HIM
135	Amphibians	4.15	3.06	5.54	HIM
136	Amphibians	1.76	0.46	3.91	QTP
137	Amphibians	8.04	5.93	10.87	QTP
138	Amphibians	10.01	7.65	15.15	HIM
139	Amphibians	13.76	10.73	18.88	HIM
140	Amphibians	8.2	6.26	12.4	HDM
141	Amphibians	9.48	7.09	11.48	HIM
142	Amphibians	10.56	8.3	14.91	HIM
143	Amphibians	15.25	11.62	20.4	HIM
144	Amphibians	10.16	8.06	12.76	QTP
145	Amphibians	5.56	3.9	7.66	HIM
146	Amphibians	6.77	5.23	9.28	HIM
147	Amphibians	10.54	8.13	17.55	HIM
148	Amphibians	3.11	1.52	4.96	HIM
149	Amphibians	1.53	0.81	2.72	HIM
150	Amphibians	9.99	7.31	13.2	HIM
151	Amphibians	3.6	2.44	6.5	HIM
152	Amphibians	3.95	2.77	5.26	HIM
153	Amphibians	7.86	6.35	12.64	HIM
154	Amphibians	1.78	1.01	2.63	HDM
155	Amphibians	2.78	1.88	3.64	HDM
156	Amphibians	5.14	3.92	6.96	HDM
157	Amphibians	1.83	1.02	2.97	HDM
158	Amphibians	20.21	16.28	25.93	HDM
159	Amphibians	10.56	8.45	14.61	HDM
160	Amphibians	6.2	3.77	9.12	HDM
161	Amphibians	4.46	2.96	8.76	HDM
162	Amphibians	0	0	0.91	HDM
163	Amphibians	3.72	2.26	8.76	HDM
164	Amphibians	25.71	17.94	32.28	QTP
165	Amphibians	22.77	18.27	28.06	QTP
166	Amphibians	15.68	10.91	19.42	QTP
167	Amphibians	13.53	10.87	16.92	QTP
168	Amphibians	12.67	9.75	15.74	QTP
169	Amphibians	1.54	0	3.06	HIM

170	Amphibians	12.81	10.24	15.71	QTP
171	Amphibians	12.3	9.6	15.41	QTP
172	Amphibians	11.38	8.48	14.57	HIM
173	Amphibians	10.38	8.02	15.82	HIM
174	Amphibians	6.01	4.29	7.5	HDM
175	Amphibians	7.2	5.74	9.22	HDM
176	Amphibians	3.96	2.82	5.3	HDM
177	Amphibians	5.22	4.02	6.96	HDM
178	Amphibians	0.5	0.16	1.03	QTP
179	Amphibians	4.49	3.07	6.58	HIM
180	Amphibians	9.14	7.04	12.83	HIM
181	Amphibians	10.86	8.65	15.93	HIM
182	Amphibians	0.68	0.44	0.98	HDM
183	Amphibians	7.6	5.71	9.34	QTP
184	Amphibians	1.92	1.06	3.3	QTP
185	Amphibians	4.72	3.19	6.96	HDM
186	Amphibians	9.71	7.38	12.06	HDM
187	Amphibians	10.48	8.32	12.76	HDM
188	Amphibians	12.7	10.14	15.02	HDM
189	Amphibians	14.31	11.37	16.54	HDM
190	Amphibians	20.68	17.14	24.1	HDM
191	Amphibians	16.95	12.77	20.69	HDM
192	Amphibians	4.38	3.43	5.66	HDM
193	Reptiles	10.86	6.02	13.95	HIM
194	Reptiles	13.97	9.38	18.43	HIM
195	Reptiles	11.33	7.42	16.47	HDM
196	Reptiles	7.68	4.86	11.45	HDM
197	Reptiles	9.94	6.5	14.52	HDM
198	Reptiles	7.56	4.77	11.21	HDM
199	Reptiles	12.01	7.7	17.74	HDM
200	Reptiles	3.87	2.28	5.91	HDM
201	Reptiles	15.8	9.79	24.05	HDM
202	Reptiles	17.3	11.59	21.08	HDM
203	Reptiles	16.5	10.76	24.42	HDM
204	Reptiles	8.71	5.66	12.7	HDM
205	Reptiles	4.99	3.11	7.54	HDM
206	Reptiles	4.22	2.47	6.54	HDM
207	Reptiles	7.45	4.82	10.67	HDM
208	Reptiles	3.42	2.18	5.16	HDM
209	Reptiles	3.51	1.85	4.84	HDM
210	Reptiles	4.23	2.74	6.2	HDM
211	Reptiles	6.61	4.34	9.66	HDM
212	Reptiles	6	3.93	8.76	HDM

213	Reptiles	2.38	1.41	3.67	HDM
214	Reptiles	1.84	1.01	2.89	HDM
215	Reptiles	4.06	2.63	6.06	HDM
216	Reptiles	2.9	1.77	4.44	HDM
217	Reptiles	3.37	2.13	4.99	HDM
218	Reptiles	3.44	2.06	5.16	HDM
219	Reptiles	0.82	0.38	1.43	HDM
220	Reptiles	5.79	3.66	8.46	HDM
221	Reptiles	18.65	12.22	25.27	HIM
222	Reptiles	5.72	3.02	8.79	HDM
223	Reptiles	3.2	1.65	5.81	HDM
224	Reptiles	3.52	1.92	5.53	HDM
225	Reptiles	7.36	4.56	10.48	HDM
226	Reptiles	6.29	3.71	9.28	HDM
227	Reptiles	9.2	5.93	12.93	HDM
228	Reptiles	7.42	4.33	10.99	HDM
229	Reptiles	29.93	20.67	40.95	HIM
230	Reptiles	17.45	11.57	23.33	QTP
231	Reptiles	9.09	5.98	12.8	HIM
232	Reptiles	8.84	5.28	13.89	QTP
233	Reptiles	7.98	5.16	11.48	QTP
234	Reptiles	2.68	1.21	4.16	TP
235	Reptiles	4.42	3.23	6.05	TP
236	Reptiles	5.79	3.66	7.96	TP
237	Reptiles	19.84	13.04	27.32	HDM
238	Reptiles	4.4	1.71	8.12	HDM
239	Reptiles	15.19	9.51	21.71	HDM
240	Reptiles	12.73	7.04	19.19	HDM
241	Reptiles	6.94	2.97	11.92	QTP
242	Reptiles	5.3	2.04	8.89	HIM
243	Reptiles	1.98	0.4	3.95	HDM
244	Reptiles	1.84	0.94	2.75	QTP
245	Reptiles	27.4	23.54	31.44	HIM
246	Reptiles	18.41	14.25	22.76	HIM
247	Reptiles	15.66	11.92	19.43	HIM
248	Reptiles	20.9	17.03	24.78	HIM
249	Reptiles	15.92	10.37	22.09	HIM
250	Reptiles	8.25	4.38	12.56	HIM
251	Reptiles	14.02	9.58	18.5	HIM
252	Reptiles	16.18	11.74	20.31	HIM
253	Birds	8.31	5.58	11.34	HIM
254	Birds	7.55	4.8	10.02	HIM
255	Birds	1.59	0.7	2.57	HIM

256	Birds	5.18	3.14	7.58	HIM
257	Birds	10.28	6.22	15.95	HDM
258	Birds	0.71	0.27	1.32	HIM
259	Birds	11.09	8.46	13.81	HIM
260	Birds	5.96	3.77	8.05	HIM
261	Birds	8.93	6.72	10.79	HIM
262	Birds	6.63	3.97	10.08	QTP
263	Birds	8.39	5.11	11.73	HIM
264	Birds	10.95	7.39	14.53	HIM
265	Birds	1.66	0.59	2.94	HDM
266	Birds	7.52	4.49	11.43	HDM
267	Birds	10.93	7.08	14.54	HIM
268	Birds	12.84	8.75	17.9	HIM
269	Birds	13.48	9.51	18.96	HIM
270	Birds	9.1	5.84	12.23	HIM
271	Birds	8.41	5.87	11.26	HIM
272	Birds	5.85	3.79	8.2	HIM
273	Birds	1.3	0.68	2.07	QTP
274	Birds	5.48	3.66	7.55	HIM
275	Birds	11.22	7.23	14.82	HIM
276	Birds	3.15	1.55	4.98	QTP
277	Birds	4.45	2.44	6.85	QTP
278	Birds	8.65	5.56	12.17	QTP
279	Birds	6.41	4.37	8.87	HIM
280	Birds	3.33	1.72	5.75	QTP
281	Birds	7.29	3.93	10.45	QTP
282	Birds	9.38	6.33	12.72	QTP
283	Birds	10.95	8.02	15.31	HIM
284	Birds	10.95	11.76	21.2	QTP
285	Birds	3.38	1.64	5.19	HIM
286	Birds	7.09	4.25	9.87	QTP
287	Birds	8.15	4.64	12.18	HDM
288	Birds	9.56	6.71	12.47	QTP
289	Birds	2.22	1.36	3.19	QTP
290	Birds	13.7	10.22	17.69	HDM
291	Birds	10.61	7.71	13.82	HIM
292	Birds	11.64	8.6	15.14	QTP
293	Birds	7.44	7.1	12.68	HDM
294	Birds	4.19	2.25	6.29	QTP
295	Birds	7.77	5.4	9.84	HDM
296	Birds	7.33	5.52	9.87	TP
297	Birds	2.82	1.59	4.33	TP
298	Birds	1.6	0	3.62	HIM

299	Birds	2.58	0.01	6.39	HIM
300	Birds	4.93	2.78	7.73	HIM
301	Birds	1.81	0.79	3.5	QTP
302	Birds	10.9	6.74	16.93	QTP
303	Birds	15.34	9.48	23.04	HDM
304	Birds	3.53	1.68	5.65	QTP
305	Birds	8.58	4.91	12.61	QTP
306	Mammals	5.51	3.52	7.79	QTP
307	Mammals	11.38	8.5	14.25	HDM
308	Mammals	14.78	11.84	17.71	HDM
309	Mammals	13.79	10.81	17.04	QTP
310	Mammals	15.64	12.87	18.7	HDM
311	Mammals	5.98	3.66	8.64	HDM
312	Mammals	0.67	0.14	1.36	QTP
313	Mammals	4.13	2.71	5.49	QTP
314	Mammals	5.27	3.96	6.54	HIM
315	Mammals	2.7	1.6	3.87	HIM
316	Mammals	0.69	0.22	1.19	HDM
317	Mammals	1.69	0.86	2.57	QTP
318	Mammals	3.74	2.75	4.86	HDM
319	Mammals	6.62	5.21	8.27	HDM
320	Mammals	3.24	2.2	4.38	HDM
321	Mammals	7.18	5.56	8.92	HDM
322	Mammals	10.17	7.43	13.25	HDM
323	Mammals	8.09	5.28	11.04	QTP
324	Mammals	0.38	0.16	0.68	QTP
325	Mammals	13.64	10.18	17.63	HDM
326	Mammals	4.93	3	7.18	HDM
327	Mammals	7.7	5.19	10.7	HDM
328	Mammals	8.42	5.86	11.34	HDM
329	Mammals	12.88	9.41	16.89	HDM
330	Mammals	5.95	4.15	8.11	HDM
331	Mammals	5.51	3.76	7.48	HDM
332	Mammals	2.9	1.77	4.22	QTP
333	Mammals	3.73	2.48	5.16	HIM
334	Mammals	4.19	2.85	5.67	QTP
335	Mammals	2.5	1.45	3.75	QTP
336	Mammals	3.08	2.12	4.13	HDM
337	Mammals	7.98	6.59	9.37	QTP
338	Mammals	2.93	2.16	3.76	QTP
339	Mammals	2.69	1.92	3.52	HDM
340	Mammals	1.58	0.42	3.08	HDM
341	Mammals	7.49	3.94	11.45	HDM

342	Mammals	4.23	1.52	7.15	HDM
343	Mammals	9.82	5.07	14.99	HIM
344	Mammals	2.15	1.59	2.85	HDM
345	Mammals	0.93	0.72	1.15	HDM
346	Mammals	0.82	0.62	1.04	HDM
347	Mammals	0.74	0.55	0.96	HDM
348	Mammals	2.15	1.56	2.74	HDM
349	Mammals	5.43	4.59	6.32	HDM
350	Mammals	6.47	5.51	7.46	HDM
351	Mammals	7.37	6.31	8.48	HDM
352	Mammals	4.84	4.05	5.65	HDM
353	Mammals	3.96	3.24	4.75	HDM
354	Mammals	2.57	2.05	3.15	HDM
355	Mammals	0.44	0.25	0.67	HDM
356	Mammals	9.55	8.33	10.74	HDM
357	Mammals	5.82	4.44	7.32	HDM
358	Mammals	7.43	6.16	8.68	HDM
359	Mammals	8.36	7.34	9.4	HDM
360	Mammals	6.39	5.44	7.38	QTP
361	Mammals	6.39	5.44	7.38	QTP
362	Mammals	4.59	3.7	5.52	QTP
363	Mammals	1.71	1.22	2.32	QTP
364	Mammals	5.8	4.85	6.75	HIM
365	Mammals	5.14	4.18	6.07	QTP
366	Mammals	6.19	5.25	7.17	HIM
367	Mammals	5.87	4.88	6.84	QTP

Table S17. Inferred times with 95% HPD and direction of dispersal events of QTP and its subregions.

Number	Group	Median (Ma)	Lower (Ma)	Upper (Ma)	Direction
1	Invertebrates	8.21	3.14	13.73	HDM→TP
2	Invertebrates	21.36	14.44	29.31	HDM→TP
3	Invertebrates	5.37	3.3	7.98	HDM→TP
4	Invertebrates	17.37	12.26	22.85	HDM→TP
5	Invertebrates	53.63	45.29	62.26	HDM→HIM
6	Invertebrates	33.84	26.74	41.36	HIM→HDM
7	Invertebrates	23.56	17.52	30.13	HDM→TP
8	Invertebrates	10.83	5.91	15.96	HDM→TP
9	Invertebrates	10.15	6.53	15.7	HDM→TP
10	Invertebrates	6.78	2.24	11.96	HDM→RW
11	Invertebrates	8.36	3.68	13.29	HDM→CNA
12	Invertebrates	14.93	9.78	20.52	HDM→CNA
13	Invertebrates	25.48	19.05	30.79	SC→HDM
14	Invertebrates	2.31	1.02	3.9	SC→HDM
15	Invertebrates	3.69	1.99	6.04	HDM→TP
16	Invertebrates	15.82	12.46	19.21	HDM→HIM
17	Invertebrates	19.19	16.08	22.54	HDM→HIM
18	Invertebrates	14.78	11.21	18.62	HIM→CNA
19	Invertebrates	5.74	3.34	9.04	HIM→HDM
20	Invertebrates	13.64	9.7	17.48	HDM→SEA
21	Invertebrates	14.09	10.75	17.52	HDM→SEA
22	Invertebrates	17.89	14.33	21.54	HDM→SC
23	Invertebrates	26.32	22.78	30.25	HDM→SC
24	Invertebrates	25.06	21.47	28.64	HDM→SEA
25	Invertebrates	12.96	8.91	17.85	HDM→SC
26	Invertebrates	6.96	5.55	8.64	HDM→SEA
27	Invertebrates	11.12	8.8	13.83	HDM→HIM
28	Invertebrates	22.19	18.82	25.96	HDM→SEA
29	Invertebrates	22.94	18.88	26.75	SC→HDM
30	Invertebrates	27.11	23.86	30.91	SC→HIM
31	Invertebrates	18.92	12.32	24.06	HIM→SEA
32	Invertebrates	15.61	11.8	19.54	HIM→SC
33	Invertebrates	19.01	14.95	23.11	HIM→HDM
34	Invertebrates	3.31	1.7	5.62	SC→TP
35	Invertebrates	14.42	11.95	17.23	SC→HDM
36	Invertebrates	17.99	12.22	23.1	HDM→SEA
37	Invertebrates	22.48	19.39	26.22	SC→HDM
38	Invertebrates	19.75	16.7	22.96	CNA→HDM
39	Invertebrates	11.62	8.67	14.51	HDM→EA
40	Invertebrates	5.74	3.64	8.43	SC→HDM
41	Invertebrates	2.42	1.39	3.73	HDM→TP
42	Invertebrates	13.17	10.26	16.32	HDM→SC

43	Invertebrates	11.5	8.87	14.45	HDM→SC
44	Invertebrates	9.13	6.8	11.53	HDM→SEA
45	Invertebrates	16.8	13.79	20.5	HDM→RW
46	Invertebrates	12.2	8.39	16.61	HDM→SC
47	Invertebrates	15.28	12.24	18.72	EA→TP
48	Invertebrates	16.59	13.73	20.15	HDM→EA
49	Invertebrates	29.02	25.54	32.75	SC→HDM
50	Invertebrates	29.8	26.15	33.4	SC→HDM
51	Invertebrates	22.66	19.09	26.13	HDM→CNA
52	Amphibians	4.8	3.6	6.17	SEA→HDM
53	Amphibians	5.05	3.99	6.24	EA→HDM
54	Amphibians	9.14	7.26	12.11	SA→HIM
55	Amphibians	15.47	11.69	19.49	SEA→HIM
56	Amphibians	13.55	10.78	17.59	SEA→HIM
57	Amphibians	5.32	3.02	7.67	SEA→HIM
58	Amphibians	27.45	19.51	33.16	SEA→HIM
59	Amphibians	14.98	10.1	20.36	SEA→HDM
60	Amphibians	9.08	5.53	12.05	SC→HDM
61	Amphibians	16.06	12.77	21.27	SC→HIM
62	Amphibians	19.9	8.73	28.16	SEA→HIM
63	Amphibians	11.55	9.04	15.3	SC→HDM
64	Amphibians	6.24	4.67	8.53	SEA→HIM
65	Amphibians	0.37	0.21	0.57	SC→HDM
66	Amphibians	13.86	11.61	18.66	SEA→QTP
67	Amphibians	20.87	18.12	26.51	SEA→HIM
68	Amphibians	17.22	14.16	21.52	SEA→HIM
69	Amphibians	2.51	1.45	3.35	SC→HDM
70	Amphibians	9.47	5.04	15.12	SEA→HDM
71	Amphibians	6.52	2.6	10.8	SEA→HIM
72	Amphibians	9.39	7.23	11.97	HDM→EA
73	Amphibians	6.26	5.47	10.08	HIM→SEA
74	Amphibians	10.01	7.65	16.15	HIM→SEA
75	Amphibians	3.43	2.4	4.61	HDM→EA
76	Amphibians	11.25	7.29	16.5	SEA→HDM
77	Amphibians	7	3.96	10.23	SC→HDM
78	Amphibians	18.12	15.29	22.7	SC→HDM
79	Amphibians	10.94	8.62	13.11	SEA→HIM
80	Reptiles	48.08	36.27	55.72	HIM→CNA
81	Reptiles	16.78	10.27	25.88	SA→HIM
82	Reptiles	5.75	2.59	8.49	SEA→HIM
83	Reptiles	1.48	0.39	2.72	HIM→EA
84	Reptiles	7.09	3.7	10.17	HDM→EA
85	Reptiles	27.87	20.3	35.15	HDM→SC
86	Reptiles	29.69	22.96	38.34	SEA→HDM

87	Reptiles	17.45	11.57	23.33	HIM→TP
88	Reptiles	22.3	15.42	28.71	CNA→HIM
89	Reptiles	36.7	27.86	44.12	CNA→HIM
90	Reptiles	6.98	4.14	10.72	HIM→SCSEA
91	Reptiles	13.5	8.45	19.73	SEA→HIM
92	Reptiles	12.21	7.77	16.81	CNA→HIM
93	Reptiles	7.98	5.16	11.48	HDM→HIM
94	Reptiles	7.29	4.71	10.15	CNA→TP
95	Reptiles	17.04	11.65	23.13	SC→HDM
96	Reptiles	13.05	7.36	19.19	SEA→QTP
97	Reptiles	20.64	14.15	27.68	EA→HDM
98	Reptiles	21.39	16.85	34.45	SC→QTP
99	Reptiles	6.13	3.07	8.71	SEA→HIM
100	Reptiles	6.73	3.48	11	SEA→HIM
101	Reptiles	13.61	8.6	19.67	SEA→HIM
102	Reptiles	7.32	3.7	11.29	SA→HIM
103	Reptiles	3.65	0.89	6.83	SC→HDM
104	Reptiles	3.51	1.24	6.3	SC→HDM
105	Reptiles	6.08	2.9	9.65	SEA→HDM
106	Reptiles	4.93	1.3	8.83	SC→HIM
107	Reptiles	3.83	1.33	6.72	SC→HIM
108	Reptiles	1.84	0.94	2.75	HIM→HDM
109	Reptiles	3.08	2.12	4.08	SA→HIM
110	Reptiles	13.74	10.58	17.05	SA→QTP
111	Reptiles	30.02	25.62	34.27	SA→HIM
112	Reptiles	0.65	0.22	1.11	SEA→HDM
113	Reptiles	25.2	21.58	28.95	HIM→SEA
114	Reptiles	17.9	14.32	21.61	HIM→SEA
115	Birds	5.66	3.27	7.84	HIM→SCSEA
116	Birds	9.3	6.39	12.3	HIM→TPCNAEA
117	Birds	0.18	0.02	0.42	HIM→SC
118	Birds	1.92	0.82	3.51	RW→TP
119	Birds	12.26	7.55	18.38	RW→HDM
120	Birds	15.57	11.92	20.58	RW→HIM
121	Birds	1.47	0.8	2.24	CNA→HIM
122	Birds	6.63	3.97	10.08	HDM→HIM
123	Birds	5.86	3.59	9.29	HIM→SEA
124	Birds	0.07	0	0.3	HDM→EA
125	Birds	3.83	2.21	5.85	SEA→HIM
126	Birds	14.46	9.76	19.26	CNA→HIM
127	Birds	11.52	7.79	15.7	HIM→RW
128	Birds	1.34	0.55	2.4	SEA→HIM
129	Birds	12.78	8.62	17.41	HIM→SEA
130	Birds	1.3	0.68	2.07	HIM→HDM

131	Birds	11.83	8.32	16.45	SEA→HIM
132	Birds	4.14	2.56	5.89	HIM→SEA
133	Birds	1.03	0.52	1.67	CNA→QTP
134	Birds	11.1	8.68	13.63	SEA→QTP
135	Birds	8.76	5.89	11.87	HIM→SC
136	Birds	11.44	7.65	15.95	HIM→SEA
137	Birds	20.67	15.75	25.97	RW→TP
138	Birds	10.95	11.76	21.2	TP→HIM
139	Birds	13.45	9.83	17.5	CNA→HDM
140	Birds	9.56	6.71	12.47	HDM→TP
141	Birds	2.22	1.36	3.19	TP→HIM
142	Birds	11.64	8.6	15.14	HDM→HIM
143	Birds	1.49	0.69	2.56	TP→CNAEA
144	Birds	14.53	10.46	18.46	RW→HDM
145	Birds	7.44	5.08	9.99	HDM→RW
146	Birds	2.8	1.75	4.05	RW→HIM
147	Birds	16.98	10.52	21.91	RW→HIM
148	Birds	2.53	1.11	4.52	SEA→HIM
149	Birds	11.54	7.48	15.92	RW→HIM
150	Birds	7.8	4.97	11.15	RW→HIM
151	Birds	3.44	2.03	5.4	SEA→HIM
152	Birds	2.3	0.93	4.26	SC→HIM
153	Birds	4.65	2.56	6.87	CNA→HIM
154	Birds	6.55	3.65	9.75	RW→HIM
155	Birds	13.23	8.15	18.42	SEA→HIM
156	Mammals	6.69	5.45	8.2	SEARW→HDM
157	Mammals	1.18	0.43	2.02	CNA→QTP
158	Mammals	23.54	19.48	27.82	RW→QTP
159	Mammals	4.89	3.49	6.49	RW→HIM
160	Mammals	4.13	2.71	5.49	HIM→HDM
161	Mammals	5.84	4.38	7.27	RW→HIM
162	Mammals	0.6	0.12	1.21	SEA→HIM
163	Mammals	9.27	7.33	11.52	SC→HDM
164	Mammals	5.22	2.82	8.39	HDM→SEA
165	Mammals	6.5	4.75	8.68	CNA→HIM
166	Mammals	1.69	0.86	2.57	HDM→QTP
167	Mammals	2	1.24	2.85	HDM→SA
168	Mammals	2.11	1.51	2.78	CNA→QTP
169	Mammals	10.41	7.72	12.77	CNA→TP
170	Mammals	3.51	2.25	4.82	CNA→TP
171	Mammals	7.88	6.4	9.33	SEA→QTP
172	Mammals	8.89	6.51	11.74	HDM→CNA
173	Mammals	7.94	5.66	10.64	HDM→CNA
174	Mammals	7.68	5.29	10.46	CNA→TP

175	Mammals	5.48	2.96	9.09	CNA→QTP
176	Mammals	5.25	3.26	7.44	HDM→CNAEA
177	Mammals	2.9	1.77	4.22	HIM→HDM
178	Mammals	4.19	2.85	5.67	HDM→HIM
179	Mammals	0.79	0.5	1.13	HDM→CNA
180	Mammals	7.37	5.98	8.77	HDM→SEA
181	Mammals	3.18	2.52	3.88	SEA→HIM
182	Mammals	7.53	6.16	8.87	HDM→SC
183	Mammals	14	8.91	19.5	RW→HIM
184	Mammals	1.45	1.06	1.9	HDM→SC
185	Mammals	3.7	3.05	4.42	CNA→QTP
186	Mammals	12.06	10.5	13.79	CNA→HIM
187	Mammals	10.3	9	11.7	CNA→HDM
188	Mammals	8.71	7.6	9.85	HDM→CNARW
189	Mammals	8.1	7.11	9.11	HDM→EA
190	Mammals	6.39	5.44	7.38	HDM→HIM
191	Mammals	5.87	4.88	6.84	HIM→TP

Table S18. MID and MDE data of the QTP and its three subregions inferred from biogeographical events.

	QTP			Plateau Platform			Hengduan Mountains			Himalaya		
age (Ma)	MID	MDE immigration	MDE emigration	MID	MDE_immigration	MDE_emigration	MID	MDE immigration	MDE emigration	MID	MDE immigration	MDE emigration
2	81.4	23.6	7.2	3.8	4.8	1.4	48.2	8.8	7.4	18	14.6	5.6
3	105.6	27.8	9.2	4.4	6.2	1.4	63.6	10.2	10.4	24	18	6.6
4	121.6	30	11.6	4.4	7.2	1.2	74	10.8	13.6	29.8	19.8	7.4
5	132.2	31	14.8	4	7.8	1	81.8	11	16.8	34.8	20.2	8.2
6	137.8	31.8	18.2	3.2	7.8	1	87.2	10.6	19.6	39	20.8	9
7	138.8	31.8	21	2.4	7.8	1	87.8	10.8	22.2	42.6	20.2	9.4
8	135.8	32.8	22.4	1.8	7.6	1	87	10.6	23.4	43.8	20.6	9.2
9	133.8	34.4	23	1.2	7	1	86.2	10.8	23.2	45	21.8	9.4
10	130.8	35.2	21.8	1	7	1	84	10.8	22.2	45	22.6	9.2
11	124.8	34.2	20.4	1	6.8	1	80.2	10.6	20.8	43	22	8.8
12	118.4	33.6	19.6	1	6.6	1	77.6	10.6	19.6	40.2	21.8	8.8
13	110.6	33	18.8	1	6.6	0.8	74.2	10.8	18.2	36.4	21.2	9.2
14	100.2	32	18	1	6.4	0.6	70.4	10.6	17	31.4	20.2	9
15	90.4	31	17.2	1	6	0.4	65.6	10.6	16	27.6	19.2	8.6
16	83	30.2	15.4	1	6	0.2	61	10.6	14.4	24.6	18.4	8.2
17	75.8	29.4	13.4	1	5.8	0	56.6	10.2	13.2	21.6	18	7.2
18	70	26.8	11.8	1	5.4	0	53.2	9.6	12.4	19.8	16.2	6
19	65.2	24	10.4	0.8	5.4	0	49.4	9	11.6	18.6	14.4	5.4
20	59.8	21.4	9.4	0.6	5.2	0	46.2	8.8	11	16.6	12.2	4.8
21	53.8	19	9	0.4	4.8	0	42.8	8.4	10.4	14.8	10	4.4
22	48.8	16.6	8.4	0.2	4.4	0	39.2	7.6	9.4	13.6	7.8	3.8
23	41.8	15.8	7.6	0	4	0	33.8	7	8.4	11.8	7	3.2
24	36.4	15.2	6.8	0	3.4	0	29.4	7	7.6	10.4	6.2	2.6
25	32.2	14.6	6	0	2.8	0	25.8	6.4	6.6	9.2	6.2	2.2
26	28.4	13.8	5.2	0	2.4	0	22.4	6	6	8.2	6.2	1.8
27	24.6	12.8	4.2	0	2.2	0	19	6	5.4	7	5.8	1.6
28	22	11.4	3.4	0	1.8	0	16.6	5.8	4.6	6.4	5.2	1.6
29	19.2	9.8	2.6	0	1.4	0	14	5	3.6	5.8	4.6	1.4
30	16	8.6	2	0	1	0	11.6	4.6	2.8	4.8	4	1.2
31	12.6	7.6	1.4	0	0.6	0	9.2	4.2	2	3.8	3.4	1
32	10	6.6	1.2	0	0.2	0	7.4	3.6	1.4	3.2	3	1

33	7.8	5.2	1	0	0	0	5.8	3	1	2.6	2.4	1
34	6.2	4.2	1	0	0	0	4.8	2.6	1	2.2	2	1.2
35	5.4	3.2	1	0	0	0	4	2.2	1	2.4	1.6	1.4
36	5.4	2.4	1	0	0	0	3.6	2	1	2.8	1.2	1.6
37	5.4	1.8	1	0	0	0	3.2	1.8	1	3.2	1	1.8
38	5.6	1.6	1	0	0	0	3	1.6	1	3.6	1	2
39	5.6	1.4	1	0	0	0	3	1.4	1	3.6	1	2
40	5.4	1.2	1	0	0	0	3	1	1	3.4	1	1.8
41	5	1	1	0	0	0	3	0.6	1	3	1	1.6
42	4.4	1	1	0	0	0	2.8	0.4	1	2.6	1	1.4
43	4	0.8	1	0	0	0	2.8	0.2	1	2.2	1	1.2
44	3.8	0.6	1	0	0	0	2.8	0	1	2	1	1
45	4	0.4	1	0	0	0	3	0	1	2	1	1
46	4.2	0.2	1	0	0	0	3.2	0	1	2	1	1
47	4.6	0	1	0	0	0	3.6	0	1	2	1	1
48	4.6	0	1	0	0	0	3.6	0	1	2	1	1
49	4.4	0	1	0	0	0	3.4	0	1	2	1	1
50	4	0	1	0	0	0	3	0	1	2	1	1
51	3.6	0	1	0	0	0	2.6	0	1	2	1	1
52	3	0	1	0	0	0	2.2	0	0.8	1.8	1	1
53	2.6	0	1	0	0	0	2	0	0.6	1.6	1	1
54	2.4	0	0.8	0	0	0	2	0	0.4	1.2	1	0.8
55	2.2	0	0.6	0	0	0	2	0	0.2	0.8	1	0.6
56	2	0	0.4	0	0	0	2	0	0	0.4	1	0.4
57	2	0	0.2	0	0	0	2	0	0	0.2	1	0.2
58	2.2	0	0	0	0	0	2	0	0	0	1	0
59	2.4	0	0	0	0	0	2	0	0	0	1	0
60	2.6	0	0	0	0	0	2	0	0	0	1	0
61	2.6	0	0	0	0	0	1.8	0	0	0	0.8	0
62	2.6	0	0	0	0	0	1.6	0	0	0	0.6	0
63	2.4	0	0	0	0	0	1.4	0	0	0	0.4	0
64	2	0	0	0	0	0	1	0	0	0	0.2	0
65	1.6	0	0	0	0	0	0.6	0	0	0	0	0

Table S19. A brief summary of Cenozoic Vegetation and Climatic Evolution in the Tibetan Plateau and Adjacent Regions based on previous studies.

	Early to Middle Eocene	Early Oligocene	Miocene
Plateau Platform	Hot, dry desert (12).	Hot, dry desert persists (12).	Cold, dry desert (12, 13).
Himalaya	Lowland tropical forest with palms (14).	Uplifted alpine zone with temperate forests (14).	High-altitude alpine climate with coniferous forests (14, 15).
Hengduan Mountains	Low-elevation hot desert (16).	Mixed subtropical and alpine flora (17).	Alpine forests dominant, subtropical elements in south (18, 19).
East Asia	Widespread boreotropical flora (12).	Warm temperate climate (20, 21).	Temperate climate with deciduous forests (22).
South China	Subtropical, hot and dry except south (23).	Subtropical humid climate (12).	Subtropical humid climate persists (12).
Southeast Asia	Tropical humid rainforest (12, 24).	Tropical humid climate stable (12, 24).	Tropical humid climate continues (12, 24).

Table S20. Residuals from the multiple predictor analysis on the effect of dispersal events, for each of the clades and regions included in the model_Scheme1.

Direction	Taxon	QTP	SC	SEA	EA	CNA	SA	RW
Emigration	Invertebrates	1.87	0.97	-0.62	-0.62	-0.8	-0.91	-0.37
Emigration	Amphibians	0.16	0.57	2.02	-0.65	-1.17	-1.12	-0.67
Emigration	Reptiles	0.6	0.21	1.49	-0.74	0.1	0.37	-0.77
Emigration	Birds	1	-1.66	1.09	-0.8	0.3	-1.31	1.44
Emigration	Mammals	1.11	-1.49	0.4	-0.63	1.17	-1.11	0.73
Immigration	Invertebrates	1.19	0.41	1.06	-0.27	0.26	-1.22	0.36
Immigration	Amphibians	2.03	-1.33	-0.1	-0.07	-0.67	-0.88	0.06
Immigration	Reptiles	2.08	-1.25	-0.16	-0.08	-0.67	-0.87	0.06
Immigration	Birds	2.07	-0.53	0.62	-0.76	-0.68	-0.85	0.79
Immigration	Mammals	1.77	-0.62	-0.15	-0.84	0.33	-0.95	-0.01

Table S21. Residuals from the multiple predictor analysis on the effect of dispersal events, for each of the clades and regions included in the model_Scheme2.

Direction	Taxon	TP	HIM	HDM	SC	SEA	EA	CNA	SA	RW
Emigration	Invertebrates	-0.93	0.32	2.22	1.48	-1.13	-1.09	-0.57	-0.89	-0.65
Emigration	Amphibians	-0.93	-0.76	0.07	1.16	1.61	-1.06	-0.44	-0.81	-0.54
Emigration	Reptiles	-1.05	0.52	-0.3	0.92	0.96	-1.19	0.83	0.69	-0.66
Emigration	Birds	0.02	1.19	0.67	-0.91	0.69	-1.23	0.99	-0.97	1.61
Emigration	Mammals	-1.01	0.07	1.59	-0.85	-0.09	-1.17	1.75	-0.91	0.74
Immigration	Invertebrates	1.26	-0.64	0.55	0.84	0.52	-0.69	0.47	-1.2	0.1
Immigration	Amphibians	-0.32	0.6	0.33	-0.79	-0.5	-0.4	0.05	-0.55	0.22
Immigration	Reptiles	-0.32	0.79	0.34	-0.57	-0.66	-0.41	0.08	-0.52	0.21
Immigration	Birds	0.47	1.28	0.31	0.15	0.21	-1.11	0	-0.51	0.97
Immigration	Mammals	0.91	0.43	-0.06	-0.01	-0.63	-1.27	0.93	-0.71	0.04

Table S22. Descriptive statistics of the multiple predictor model on the effect of dispersal events.

Scheme	Direction	Taxa	Predictor	Coefficient mean	Coefficient 2.5%	Coefficient 97.5%	Importance
1	Emigrations	Amphibians + Birds + Invertebrates + Mammals + Reptiles	Area	0.516	-0.349	1.356	
1	Emigrations	Amphibians + Birds + Invertebrates + Mammals + Reptiles	Branch length	0.134	-0.294	0.567	
1	Emigrations	Amphibians + Birds + Invertebrates + Mammals + Reptiles	Geographic Isolation	-0.731	-1.591	0.164	
1	Immigrations	Amphibians + Birds + Invertebrates + Mammals + Reptiles	Area	0.056	-0.765	0.879	
1	Immigrations	Amphibians + Birds + Invertebrates + Mammals + Reptiles	Branch length	-0.101	-0.511	0.311	
1	Immigrations	Amphibians + Birds + Invertebrates + Mammals + Reptiles	Geographic Isolation	-0.411	-1.255	0.426	
2	Emigrations	Amphibians + Birds + Invertebrates + Mammals + Reptiles	Area	-0.596	-1.377	0.162	
2	Emigrations	Amphibians + Birds + Invertebrates + Mammals + Reptiles	Branch length	-0.041	-0.448	0.342	
2	Emigrations	Amphibians + Birds + Invertebrates + Mammals + Reptiles	Geographic Isolation	0.382	-0.397	1.163	
2	Immigrations	Amphibians + Birds + Invertebrates + Mammals + Reptiles	Area	-1.091	-1.573	-0.613	*
2	Immigrations	Amphibians + Birds + Invertebrates + Mammals + Reptiles	Branch length	-0.273	-0.55	-0.013	*
2	Immigrations	Amphibians + Birds + Invertebrates + Mammals + Reptiles	Geographic Isolation	0.647	0.176	1.127	*

Table S23. Pearsons correlation of predictor variables for the multivariate regression model on number of shifts per region_Scheme1.

Variable	Area	Geographic isolation	Perimeter	Total branch length
Area	1	0.91	0.88	0.21
Geographic isolation	0.91	1	0.94	0.26
Perimeter	0.88	0.94	1	0.13
Total branch length	0.21	0.26	0.13	1

Table S24. Pearson's correlation of predictor variables for the multivariate regression model on number of shifts per region_Scheme2.

Variable	Area	Geographic isolation	Perimeter	Total branch length
Area	1	0.91	0.94	0.37
Geographic isolation	0.91	1	0.94	0.39
Perimeter	0.94	0.94	1	0.34
Total branch length	0.37	0.39	0.34	1

Dataset S1 (separate file). Ancestral range reconstructions of five mega-phylogenies spanning amphibians, reptiles, birds, and mammals.

Dataset S2 (separate file). Ancestral range reconstructions of 15 additional terrestrial fauna clades of the QTP.

SI References

1. M. Li, *et al*., Biogeographical Origin and Speciation of the *Anthocoris nemorum* Group. *J. Insect Sci*. **12**, 1–16 (2012).
2. Z. Zhao, S. Li, Extinction vs. Rapid Radiation: The Juxtaposed Evolutionary Histories of Coelotine Spiders Support the Eocene–Oligocene Orogenesis of the Tibetan Plateau. *Syst. Biol.* **66**, 988–1006 (2017).
3. Y. Che, *et al*., Uplift-driven diversification revealed by the historical biogeography of the cockroach *Cryptocercus* Scudder (Blattodea: Cryptocercidae) in eastern Asia. *Syst. Entomol.* **45**, 60–72 (2020).
4. X. Li, X. Bai, G. J. Kergoat, Z. Pan, G. Ren, Phylogenetics, historical biogeography and molecular species delimitation of *Gnaptorina* Reitter (Coleoptera: Tenebrionidae: Blaptini). *Syst. Entomol.* **46**, 239–251 (2021).
5. J. F. R. Tonini, K. H. Beard, R. B. Ferreira, W. Jetz, R. A. Pyron, Fully-sampled phylogenies of squamates reveal evolutionary patterns in threat status. *Biol. Conserv.* **204**, 23–31 (2016).
6. V. Deepak, *et al*., Multilocus phylogeny, natural history traits and classification of natricine snakes (Serpentes: Natricinae). *Zool. J. Linn. Soc.* **195**, 279–298 (2022).
7. N. A. Poyarkov, *et al*., An integrative taxonomic revision of slug-eating snakes (Squamata: Pareidae: Pareineae) reveals unprecedented diversity in Indochina. *PeerJ* **10**, e12713 (2022).
8. L. R. V. Alencar, *et al*., Diversification in vipers: Phylogenetic relationships, time of divergence and shifts in speciation rates. *Mol. Phylogenet. Evol.* **105**, 50–62 (2016).
9. X. Wang, *et al*., Out of Tibet: Genomic Perspectives on the Evolutionary History of Extant Pikas. *Mol. Biol. Evol.* **37**, 1577–1592 (2020).
10. Y. He, et al., Evolutionary history of Spalacidae inferred from fossil occurrences and molecular phylogeny. *Mammal Rev.* **50**, 11–24 (2020).
11. N. S. Upham, J. A. Esselstyn, W. Jetz, Inferring the mammal tree: Species-level sets of phylogenies for questions in ecology, evolution, and conservation. *PLoS Biol.* **17**, e3000494 (2019).

12. H. Meng, *et al*., Evolution of East Asian subtropical evergreen broad-leaved forests: When and how? *J. Syst. Evol.* **63**, 1045–1060 (2025).
13. R. A. Spicer *et al.*, The progressive co-evolutionary development of the Pan-Tibetan Highlands, the Asian monsoon system and Asian biodiversity. *Geol. Soc. Spec. Publ.* **549**, SP549-2023–180 (2025).
14. L. Ding, *et al.*, Quantifying the rise of the Himalaya orogen and implications for the South Asian monsoon. *Geology* **45**, 215–218 (2017).
15. M. A. Khan, *et al*., Miocene to Pleistocene floras and climate of the Eastern Himalayan Siwaliks, and new palaeoelevation estimates for the Namling–Oiyug Basin, Tibet. *Glob. Planet. Change* **113**, 1–10 (2014).
16. S. He, *et al.*, A distinctive Eocene Asian monsoon and modern biodiversity resulted from the rise of eastern Tibet. *Sci. Bull.* **67**, 2245–2258 (2022).
17. L. Ding, *et al.*, Timing and mechanisms of Tibetan Plateau uplift. *Nat. Rev. Earth Environ.* **3**, 652–667 (2022).
18. B.-N. Sun, *et al.*, Reconstructing Neogene vegetation and climates to infer tectonic uplift in western Yunnan, China. *Palaeogeogr. Palaeoclimatol. Palaeoecol.* **304**, 328–336 (2011).
19. J. Dong, Z. Li, J. Gao, Q. Wang, B. Sun, A New Fossil Species of *Nothotsuga* from the Mula Basin, Litang County, Sichuan Province and Its Paleoclimate and Paleoecology Significance. *Biology* **12**, 46 (2022).
20. X. Li, *Fossil Floras of China Through the Geological Ages* (Guangdong Science & Technology Press, 1995), pp. 1–695.
21. J. Jin, W. Liao, B. Wang, S. Peng, Global change in Cenzoic and evolution of flora in China. *Guihaia* **23**, 217–225 (2003).
22. G. Liu, Late Tertiary palynological records in northern China. *Acta Palaeontol. Sin.* **27**, 75–85 (1988).
23. Y. Xie, F. Wu, Y. Miao, L. Yang, X. Fang, Reappraisal of Eocene climate patterns in East Asia: A synthetic review. *Earth-Sci. Rev.* **271**, 105281 (2025).
24. M. B. Bush, *Tropical Rainforest Responses to Climatic Change, 2nd ed* (Springer Berlin / Heidelberg, 2011).
