# Supplementary material for: Sources and sinks: Geoclimatic dynamics shaped the assembly of endemic fauna in the world’s Third Pole
Source: Proc Natl Acad Sci U S A. 2026 Oct 26;123(44):e2602323123. doi: 10.1073/pnas.2602323123 (PMC13732247; doi:10.1073/pnas.2602323123)

Caudata BioGeoBEARS DEC
ancstates: global optim, 3 areas max. d=1e−04; e=0; j=0; LnL=−234.93

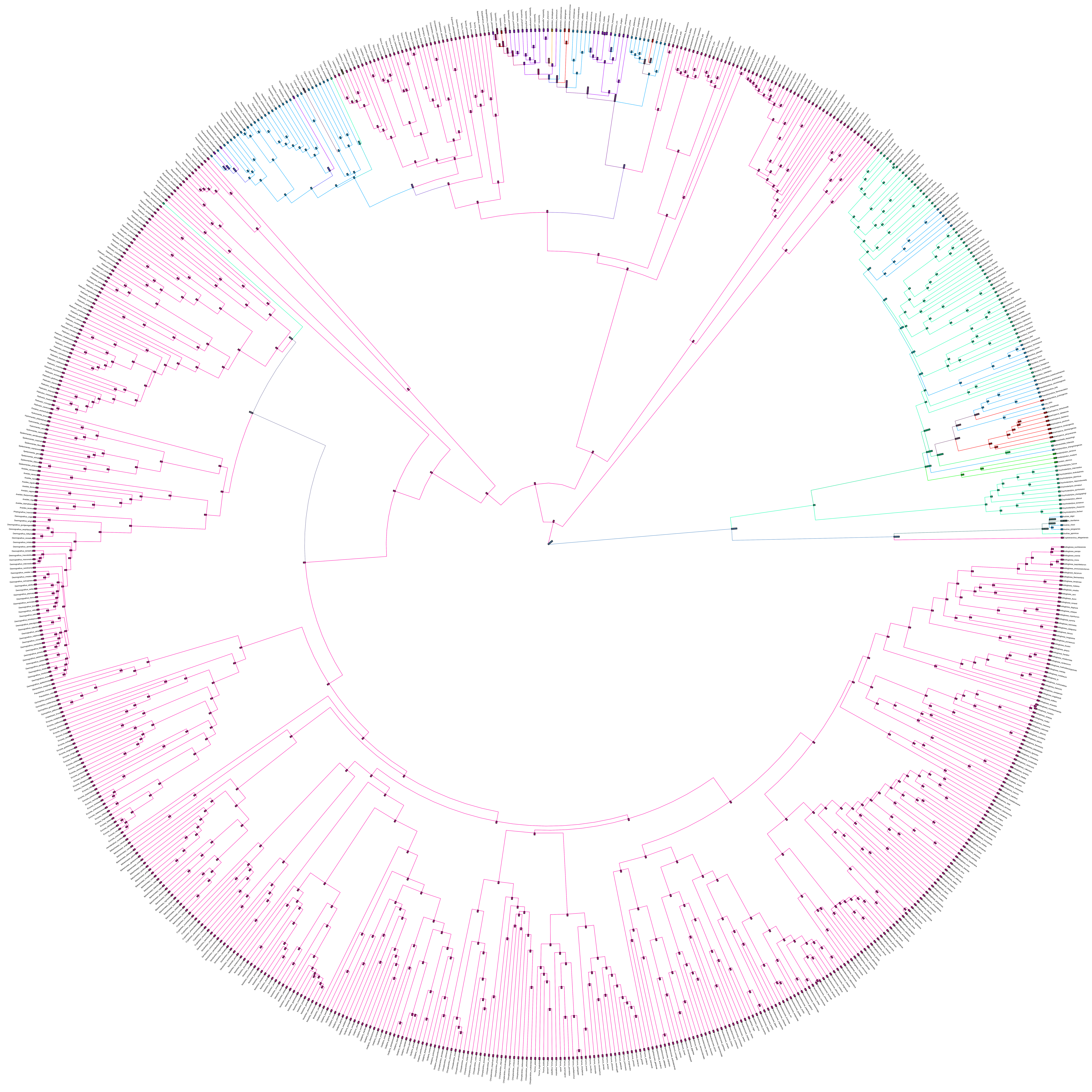

Caudata BioGeoBEARS DEC+J

ancstates: global optim, 3 areas max. d=1e−04; e=0; j=0.0012; LnL=−216.67

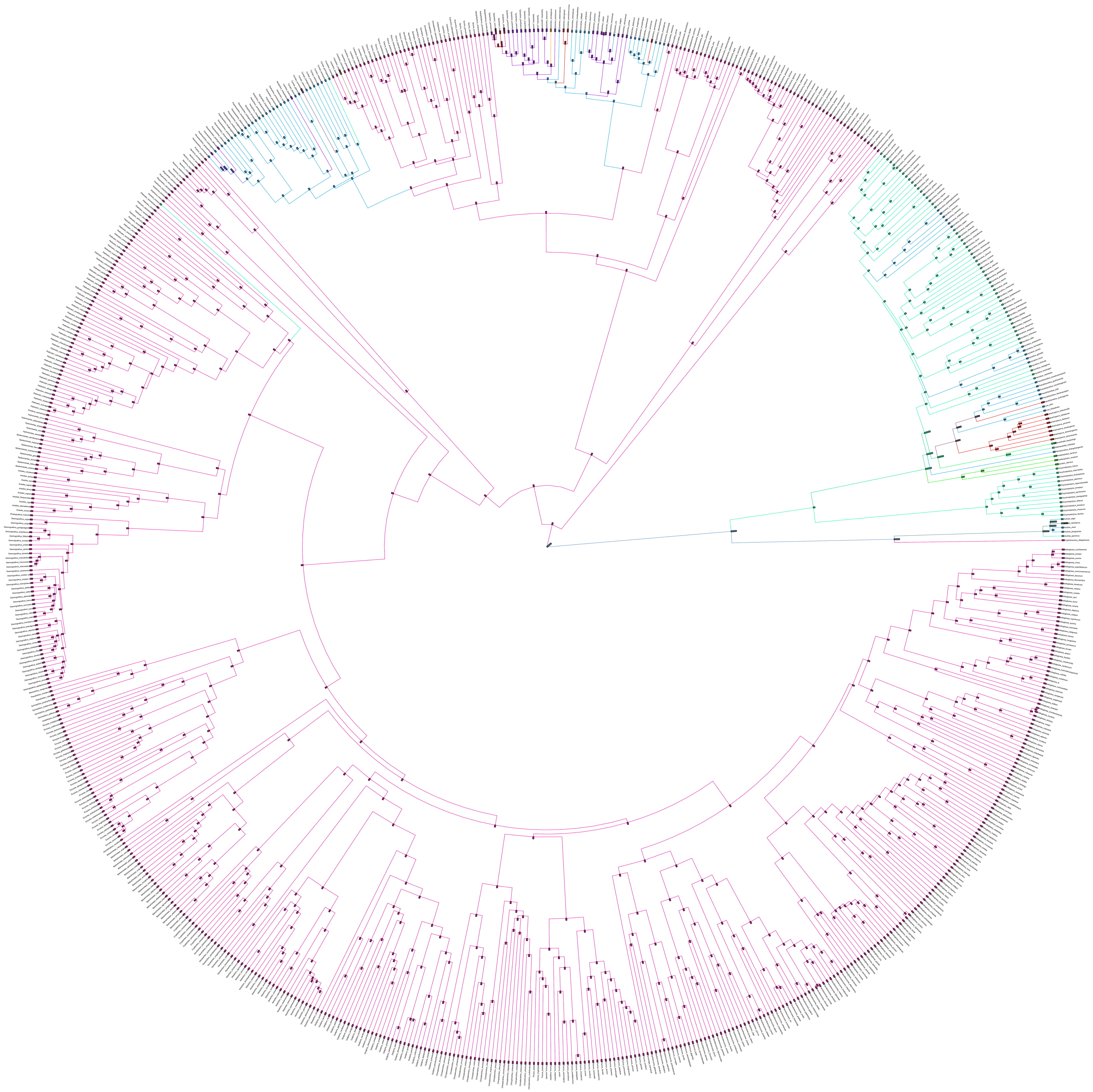

Anura BioGeoBEARS DEC
ancstates: global optim, 6 areas max. d=6e−04; e=0; j=0; LnL=−2707.51

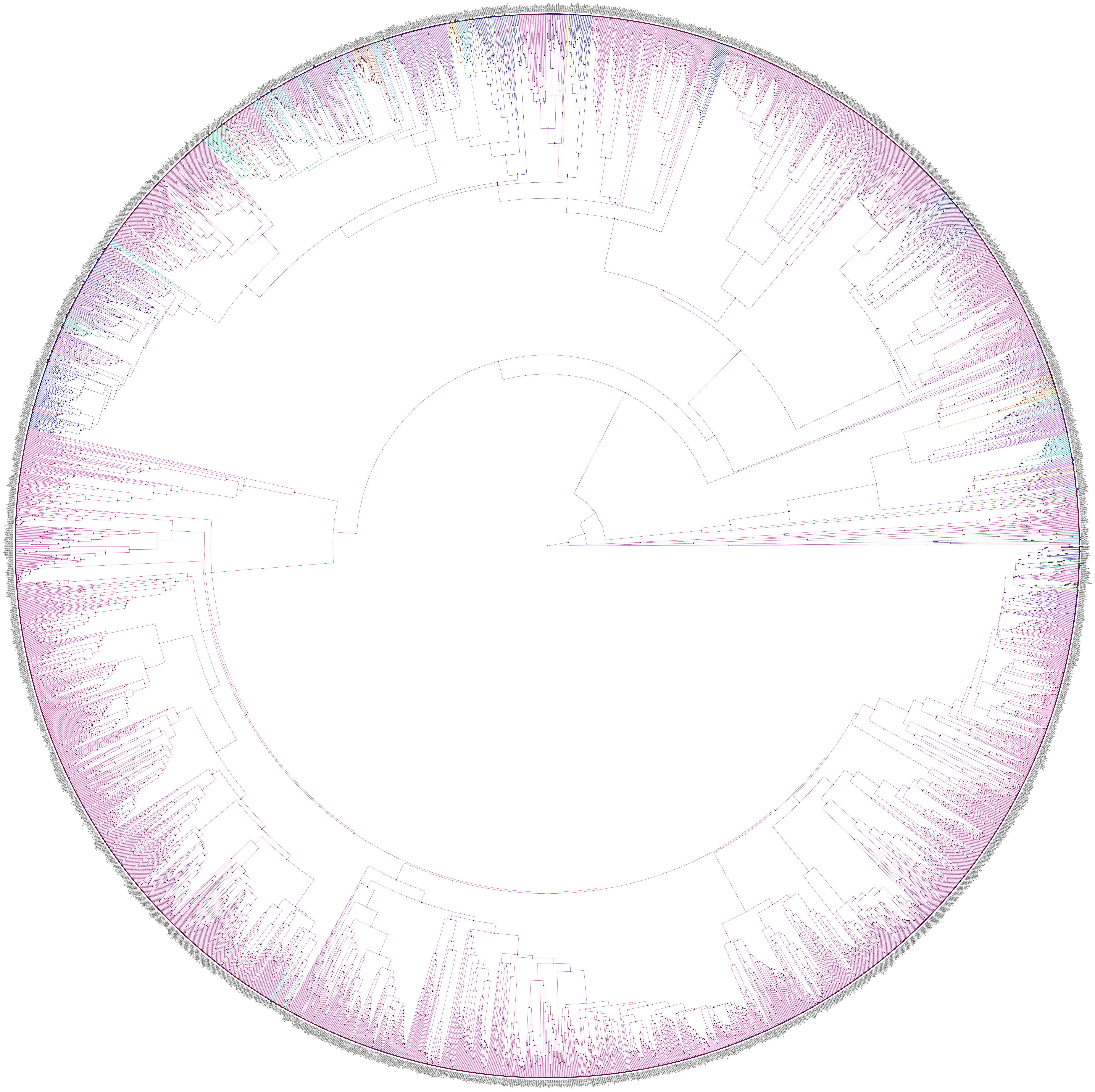

Anura BioGeoBEARS DEC+J
ancstates: global optim, 6 areas max. d=5e−04; e=0; j=9e−04; LnL=−2660.49

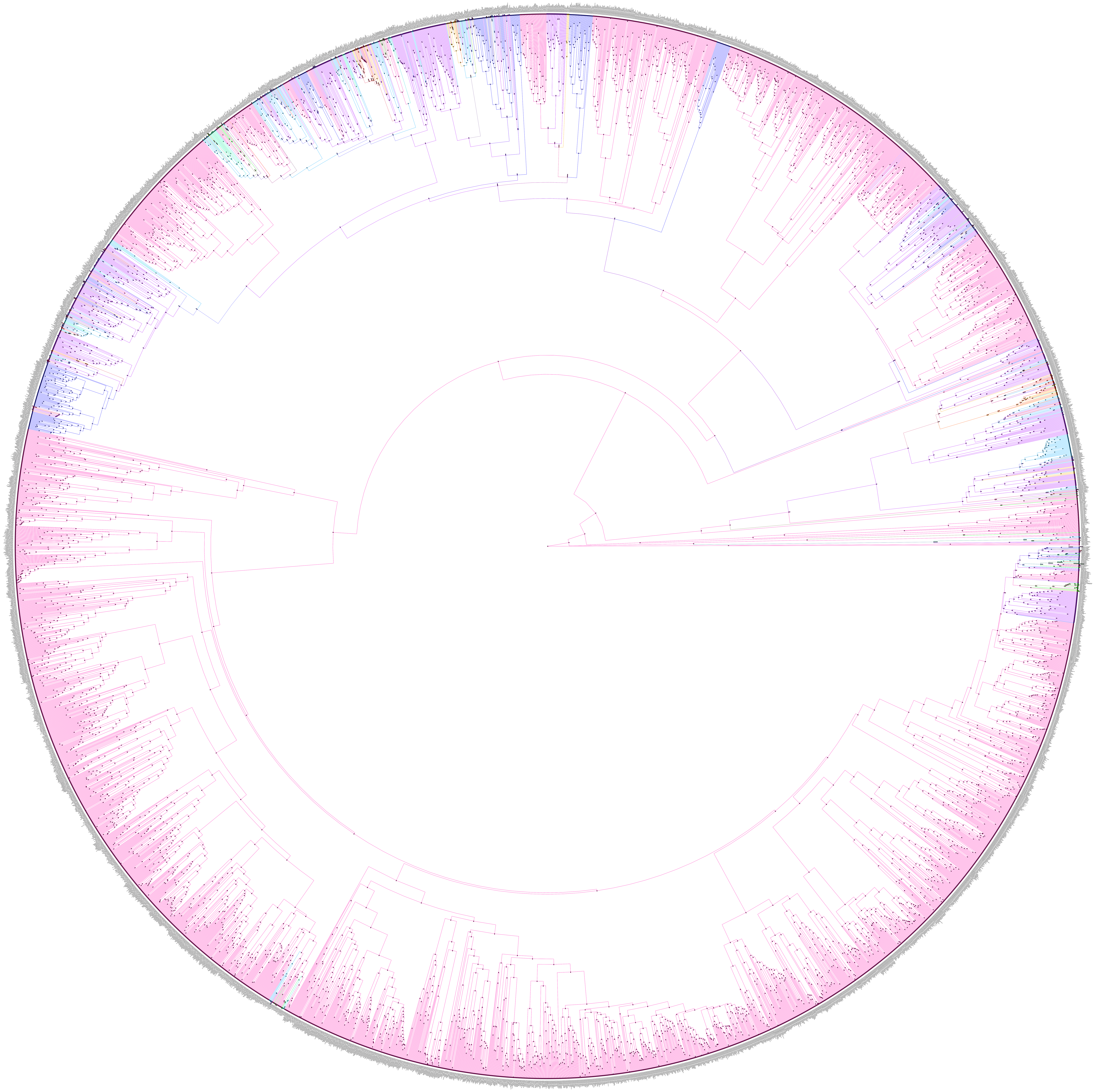

Reptiles BioGeoBEARS DEC
ancstates: global optim, 6 areas max. d=6e-04; e=0; j=0; LnL=-3326.99

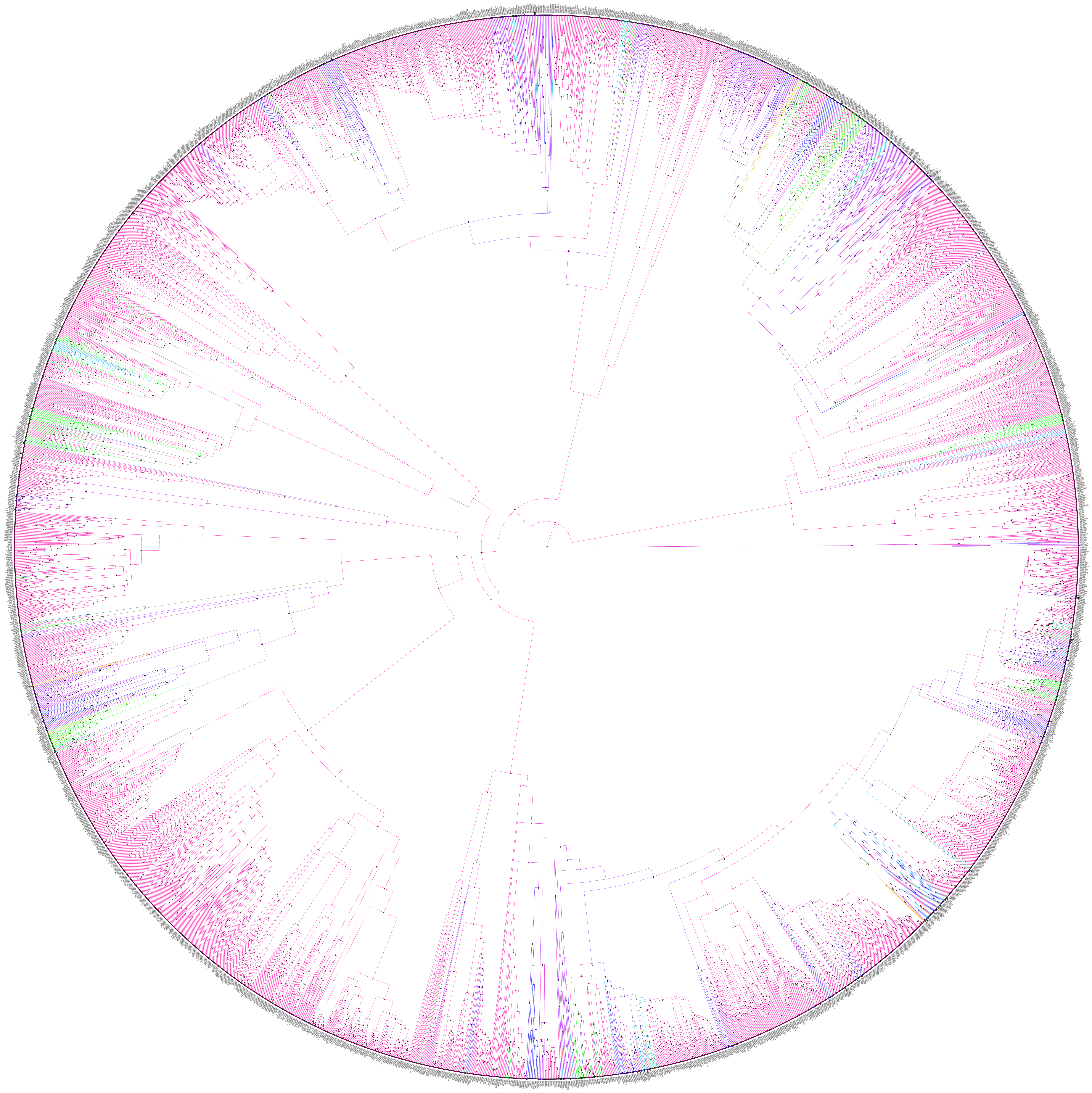

Reptiles BioGeoBEARS DEC+J
ancstates: global optim, 6 areas max. d=4e−04; e=0; j=0.0018; LnL=−3234.22

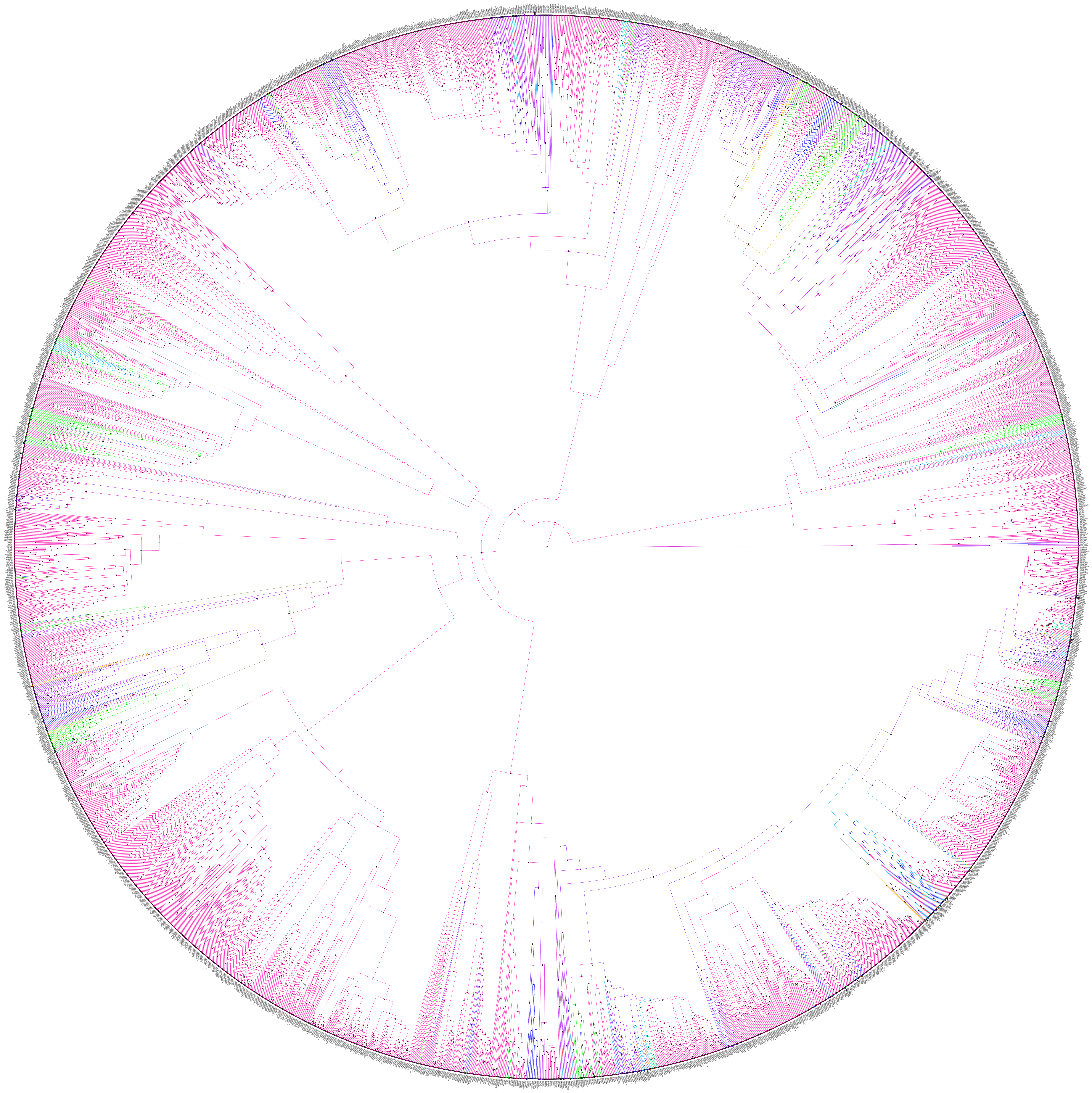

Aves BioGeoBEARS DEC
ancstates: global optim, 9 areas max. d=3e−04; e=0; j=0; LnL=−11165.72

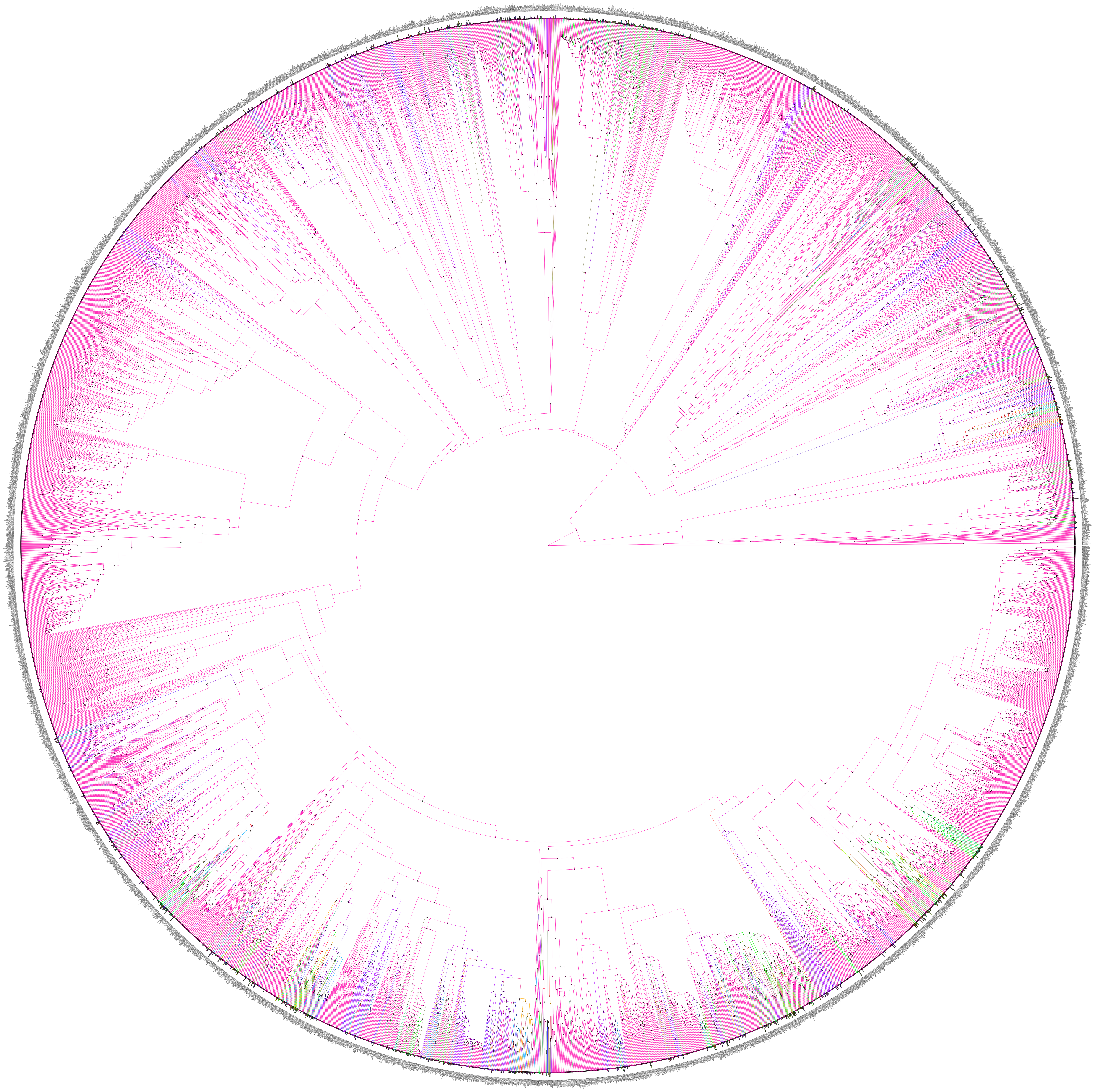

Aves BioGeoBEARS DEC+J
ancstates: global optim, 9 areas max. d=3e−04; e=0; j=0.0022; LnL=−11073.65

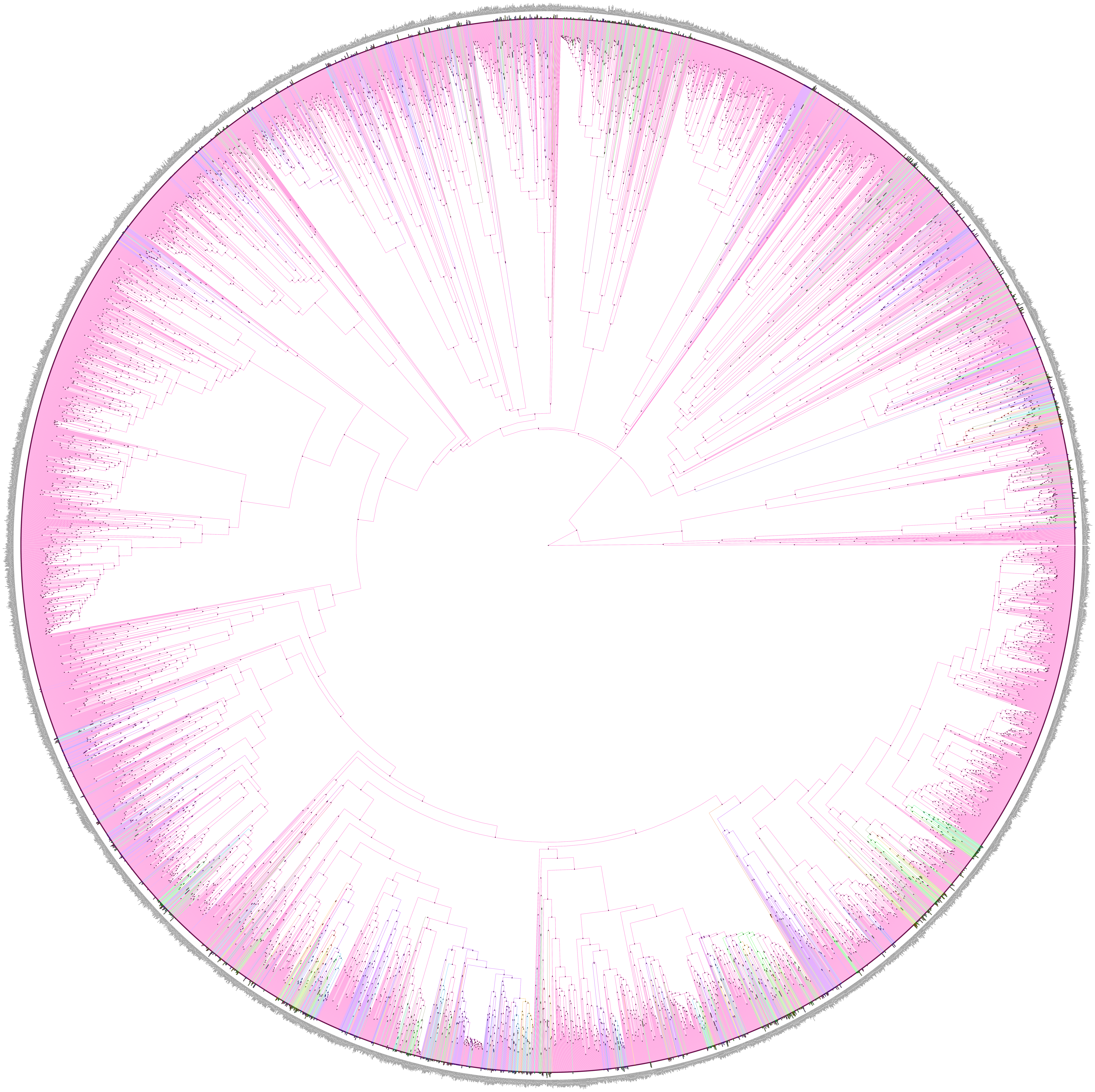

Mammals BioGeoBEARS DEC
ancstates: global optim, 8 areas max. d=0.0036; e=0; j=0; LnL=−5797.37

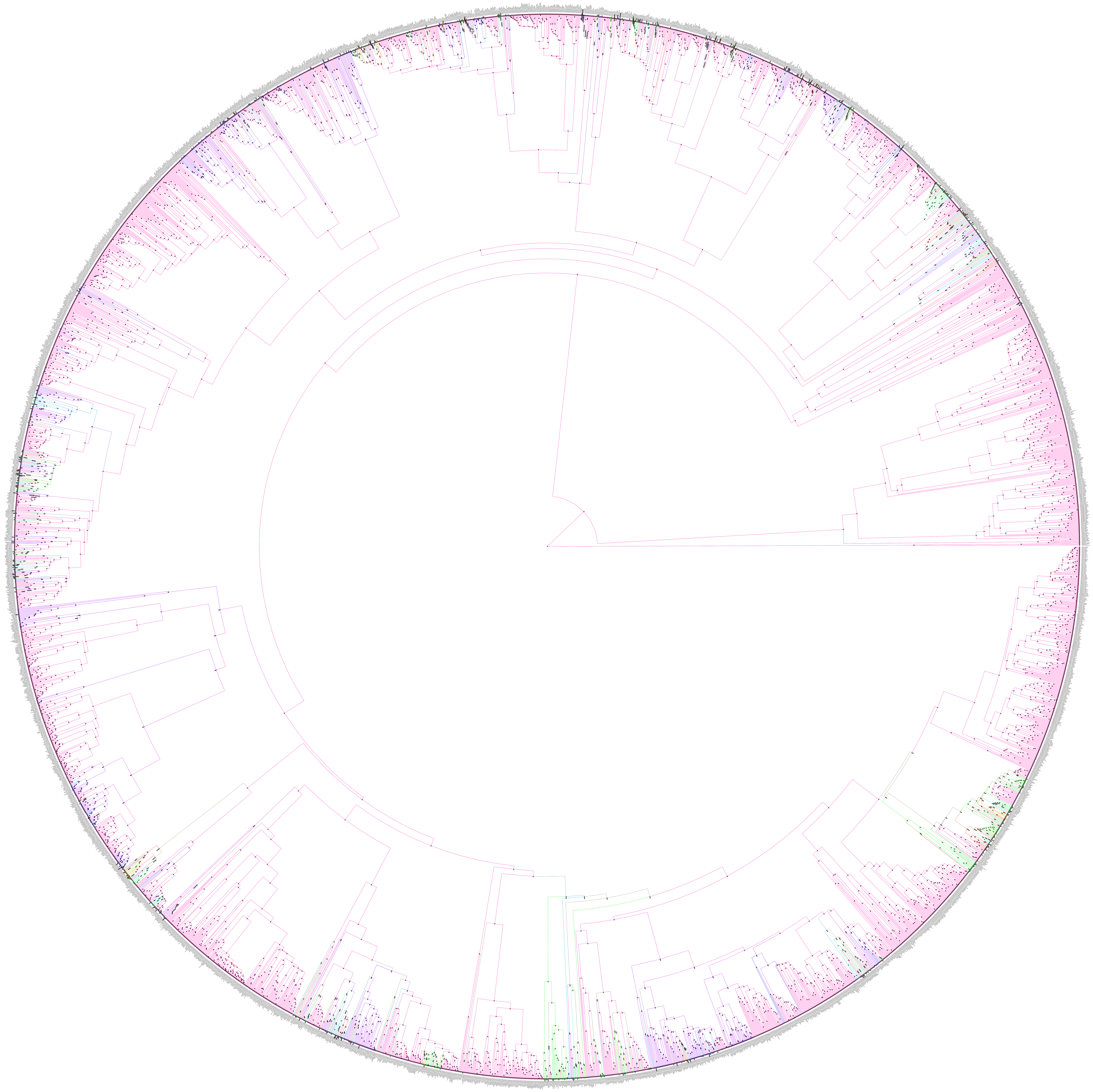

Mammals BioGeoBEARS DEC+J
ancstates: global optim, 8 areas max. d=0.0033; e=0; j=0.0023; LnL=−5733.95

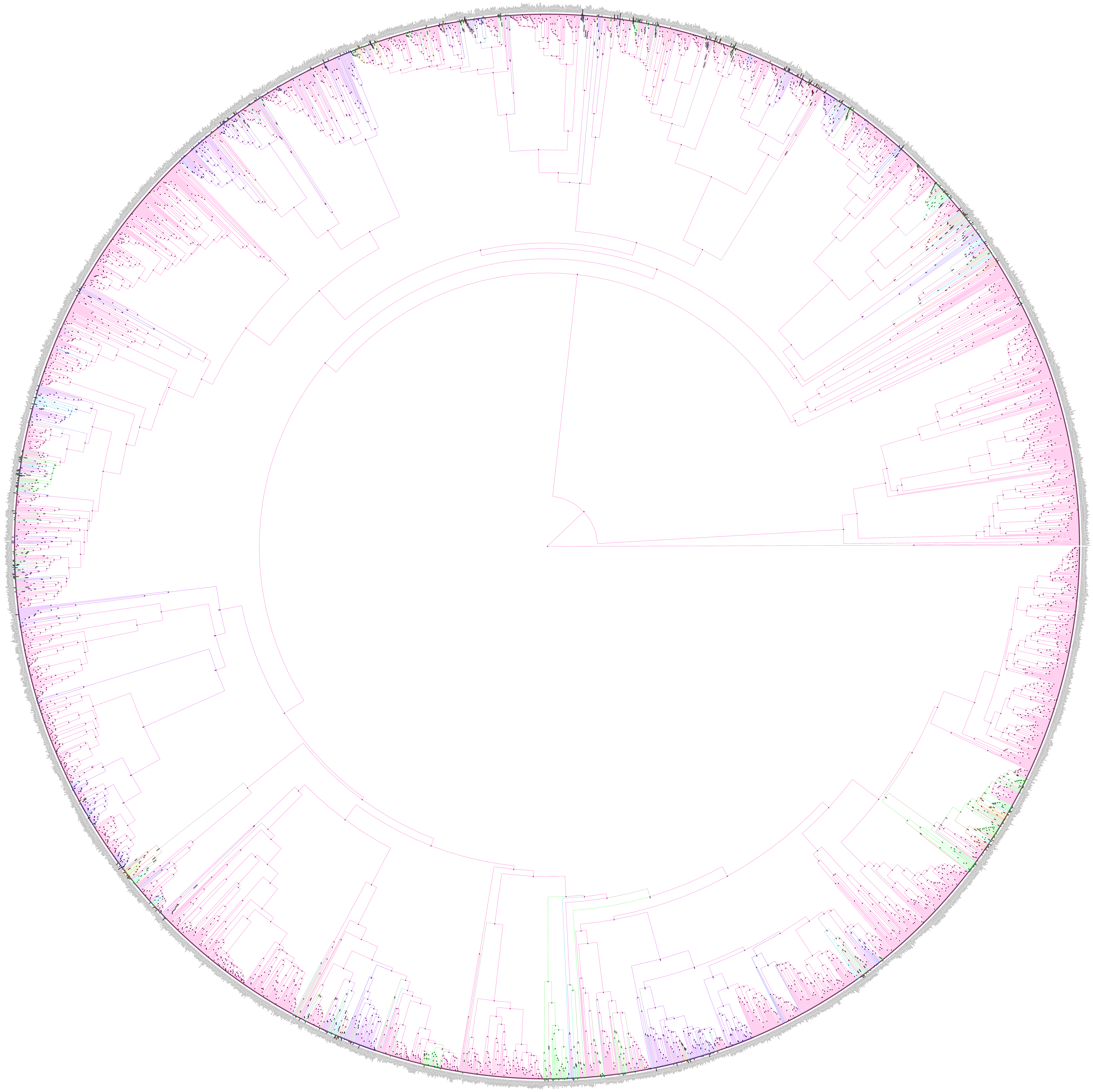

Supplement: Supplementary file 2 [file pnas.2602323123.sd01.pdf]
