# Supplementary material for: Sources and sinks: Geoclimatic dynamics shaped the assembly of endemic fauna in the world’s Third Pole
Source: Proc Natl Acad Sci U S A. 2026 Oct 26;123(44):e2602323123. doi: 10.1073/pnas.2602323123 (PMC13732247; doi:10.1073/pnas.2602323123)

**Anthocoris BioGeoBEARS DEC**
**ancstates: global optim, 3 areas max. d=0.0054; e=0.0013; j=0; LnL=−37.72**

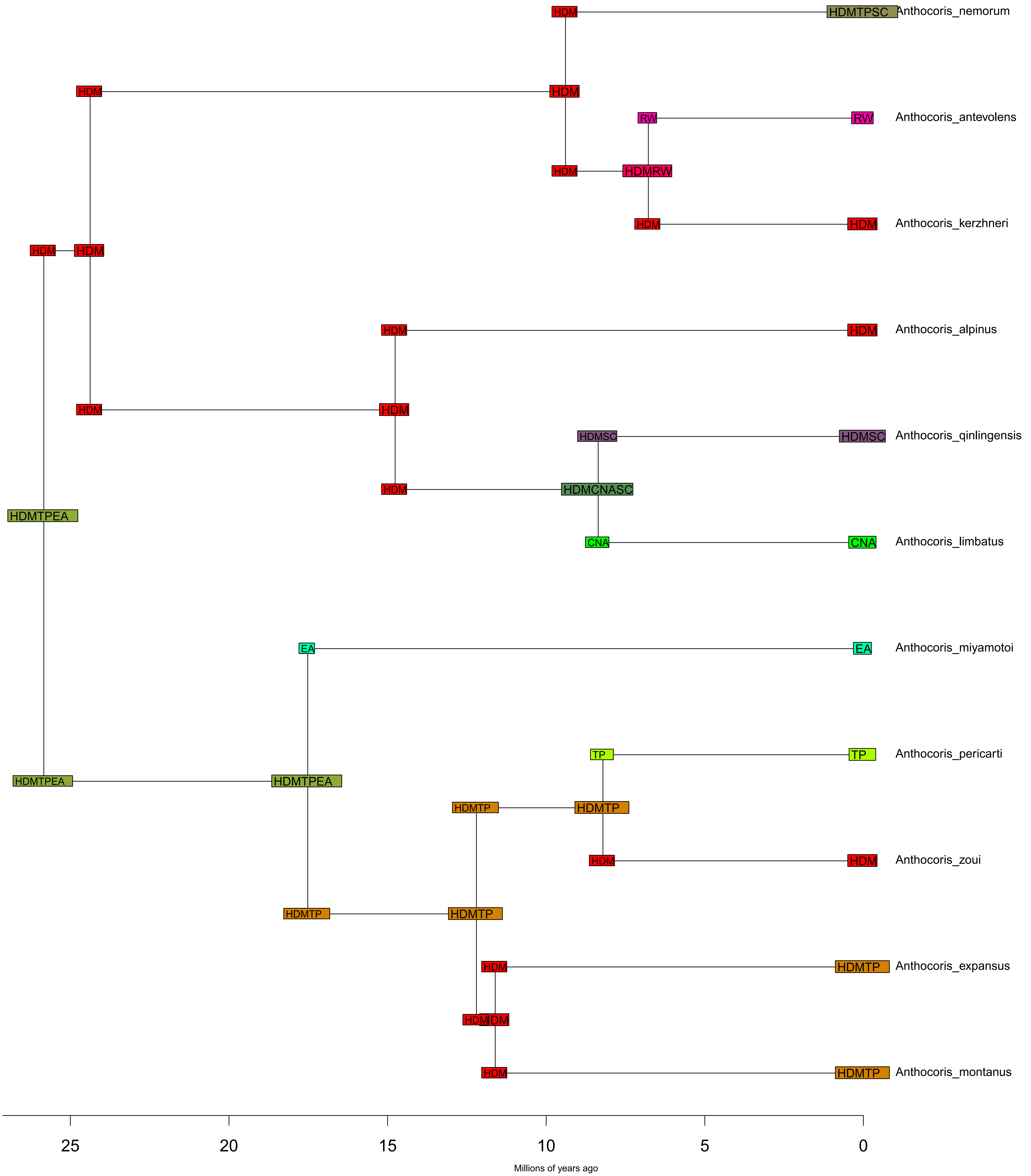

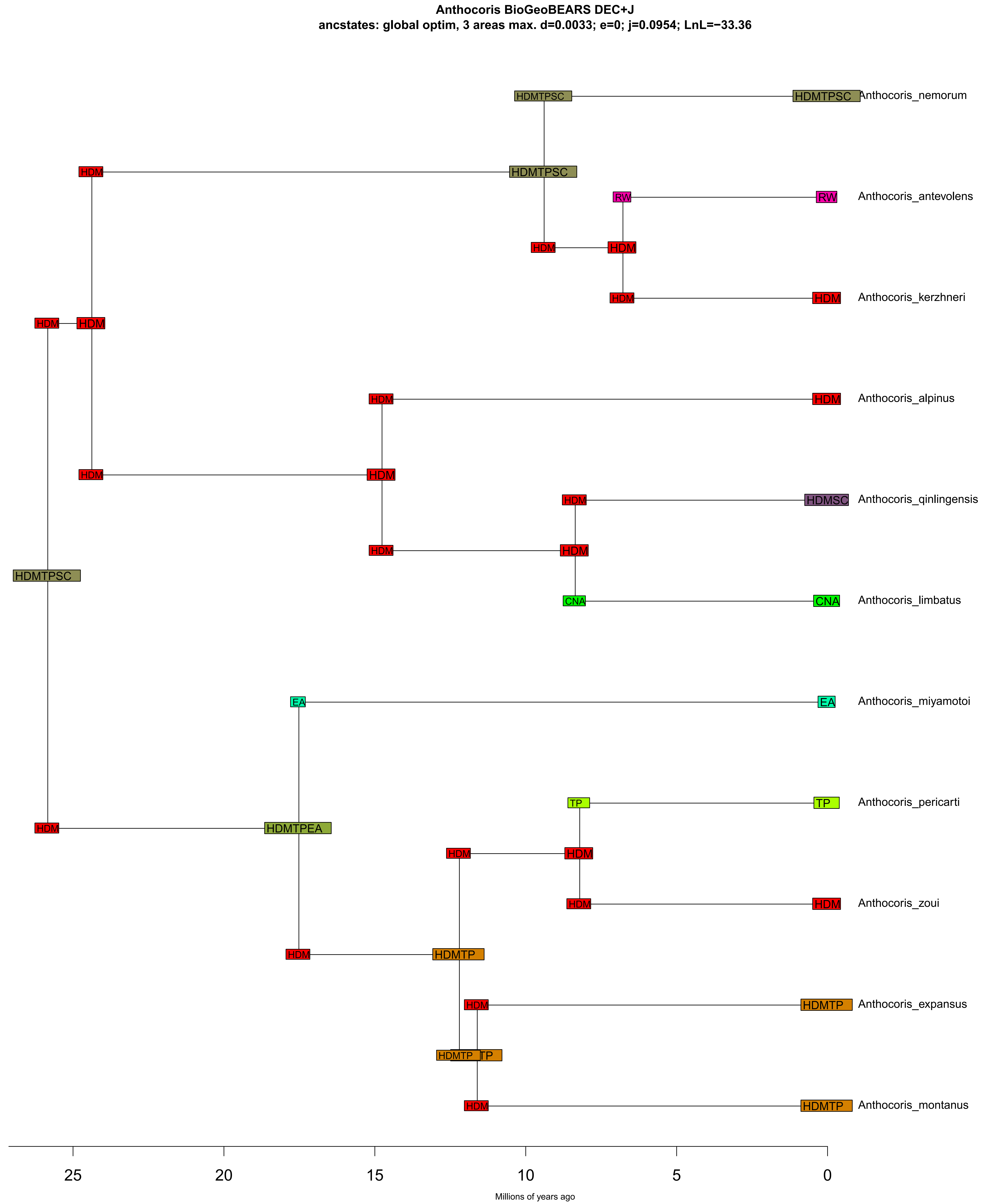
Anthocoris BioGeoBEARS DEC+J
ancstates: global optim, 3 areas max. d=0.0033; e=0; j=0.0954; LnL=−33.36
Anthocoris_nemorum
Anthocoris_antevolens
Anthocoris_kerzhneri
Anthocoris_alpinus
Anthocoris_qinlingensis
Anthocoris_limbatus
Anthocoris_miyamotoi
Anthocoris_pericarti
Anthocoris_zoui
Anthocoris_expansus
Anthocoris_montanus
25
20
15
10
5
0
Millions of years ago

**Gnaptorina BioGeoBEARS DEC**
**ancstates: global optim, 2 areas max. d=0.0017; e=0.0035; j=0; LnL=-395.07**

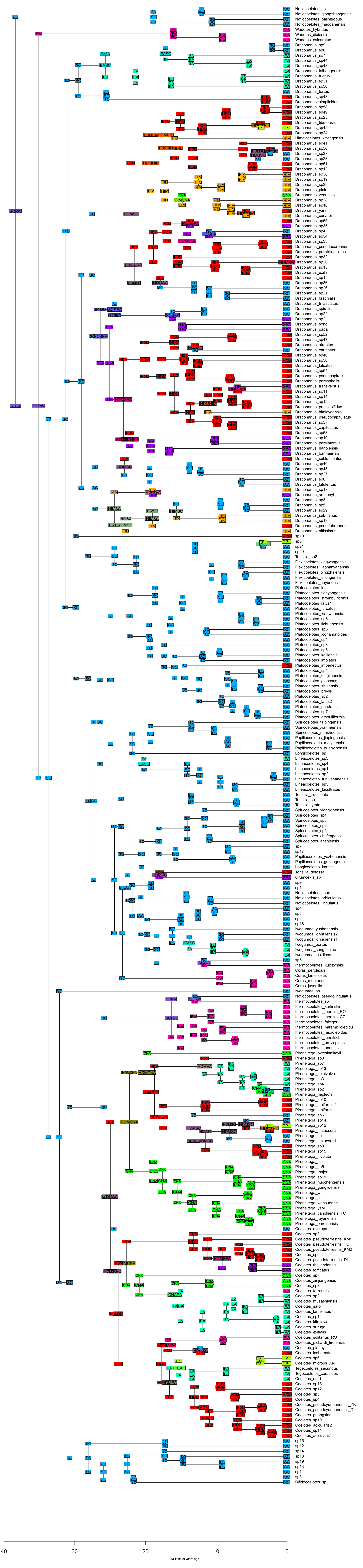

**Gnaptorina BioGeoBEARS DEC+J**
**ancstates: global optim, 2 areas max. d=1e−04; e=0; j=0.009; LnL=−303.65**

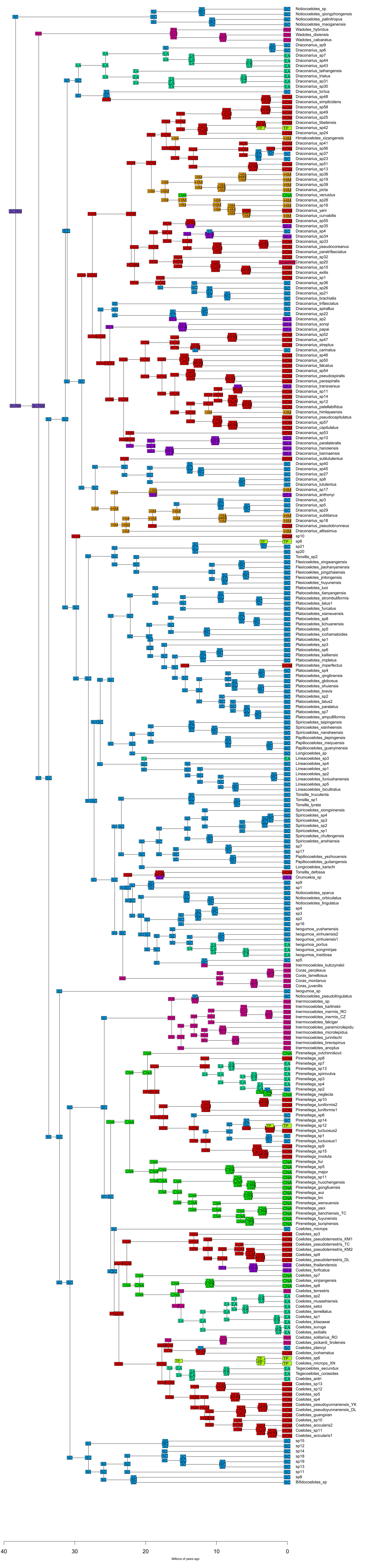

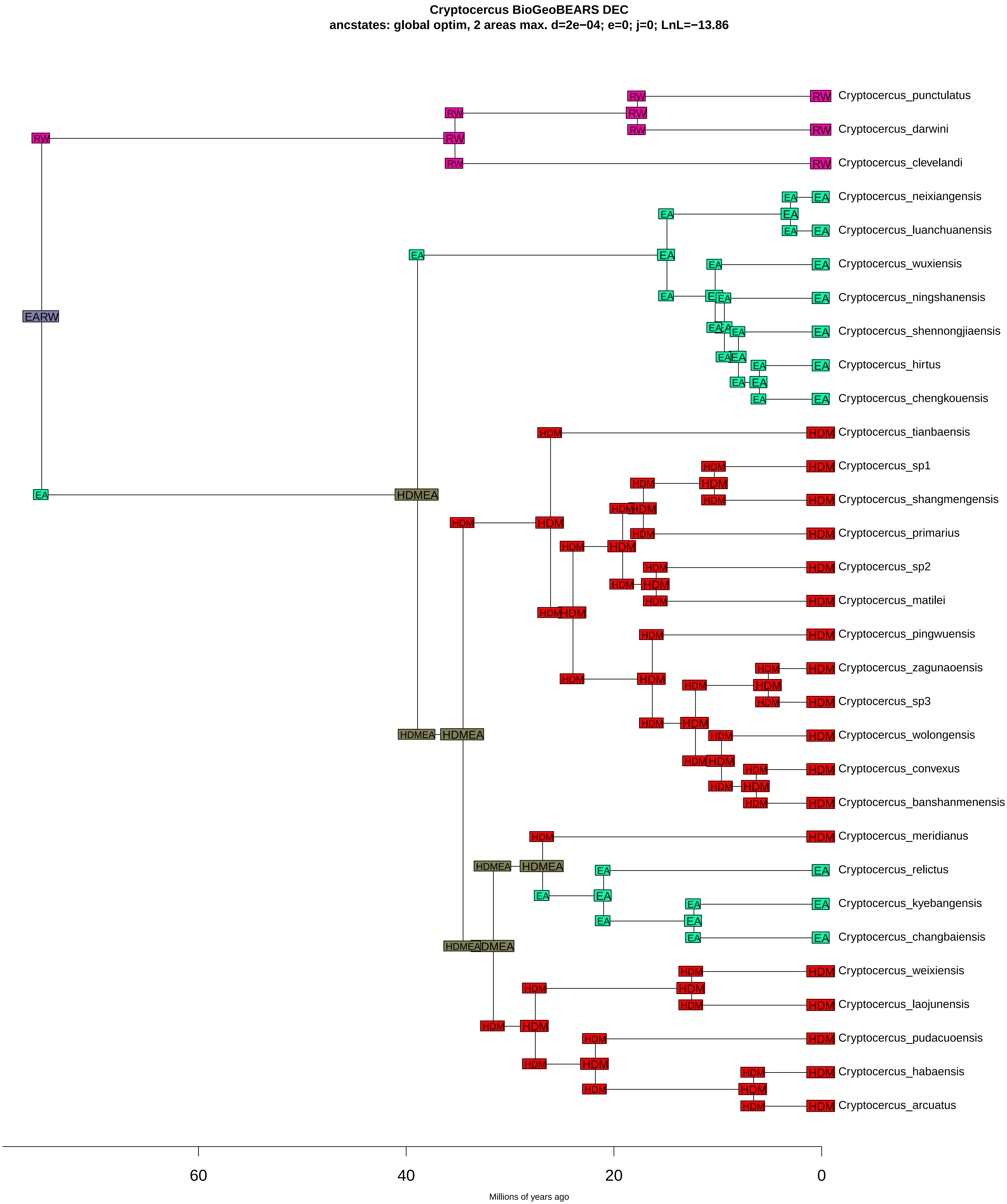
Cryptocercus BioGeoBEARS DEC
ancstates: global optim, 2 areas max. d=2e−04; e=0; j=0; LnL=−13.86
Cryptocercus_punctulatus
Cryptocercus_darwini
Cryptocercus_clevelandi
Cryptocercus_neixiangensis
Cryptocercus_luanchuanensis
Cryptocercus_wuxiensis
Cryptocercus_ningshanensis
Cryptocercus_shennongjiaensis
Cryptocercus_hirtus
Cryptocercus_chengkouensis
Cryptocercus_tianbaensis
Cryptocercus_sp1
Cryptocercus_shangmengensis
Cryptocercus_primarius
Cryptocercus_sp2
Cryptocercus_matilei
Cryptocercus_pingwuensis
Cryptocercus_zagunaoensis
Cryptocercus_sp3
Cryptocercus_wolongensis
Cryptocercus_convexus
Cryptocercus_banshanmenensis
Cryptocercus_meridianus
Cryptocercus_relictus
Cryptocercus_kyebangensis
Cryptocercus_changbaiensis
Cryptocercus_weixiensis
Cryptocercus_laojunensis
Cryptocercus_pudacuoensis
Cryptocercus_habaensis
Cryptocercus_arcuatus
RW
EA
HDM
HDMEA
EARW
60
40
20
0
Millions of years ago

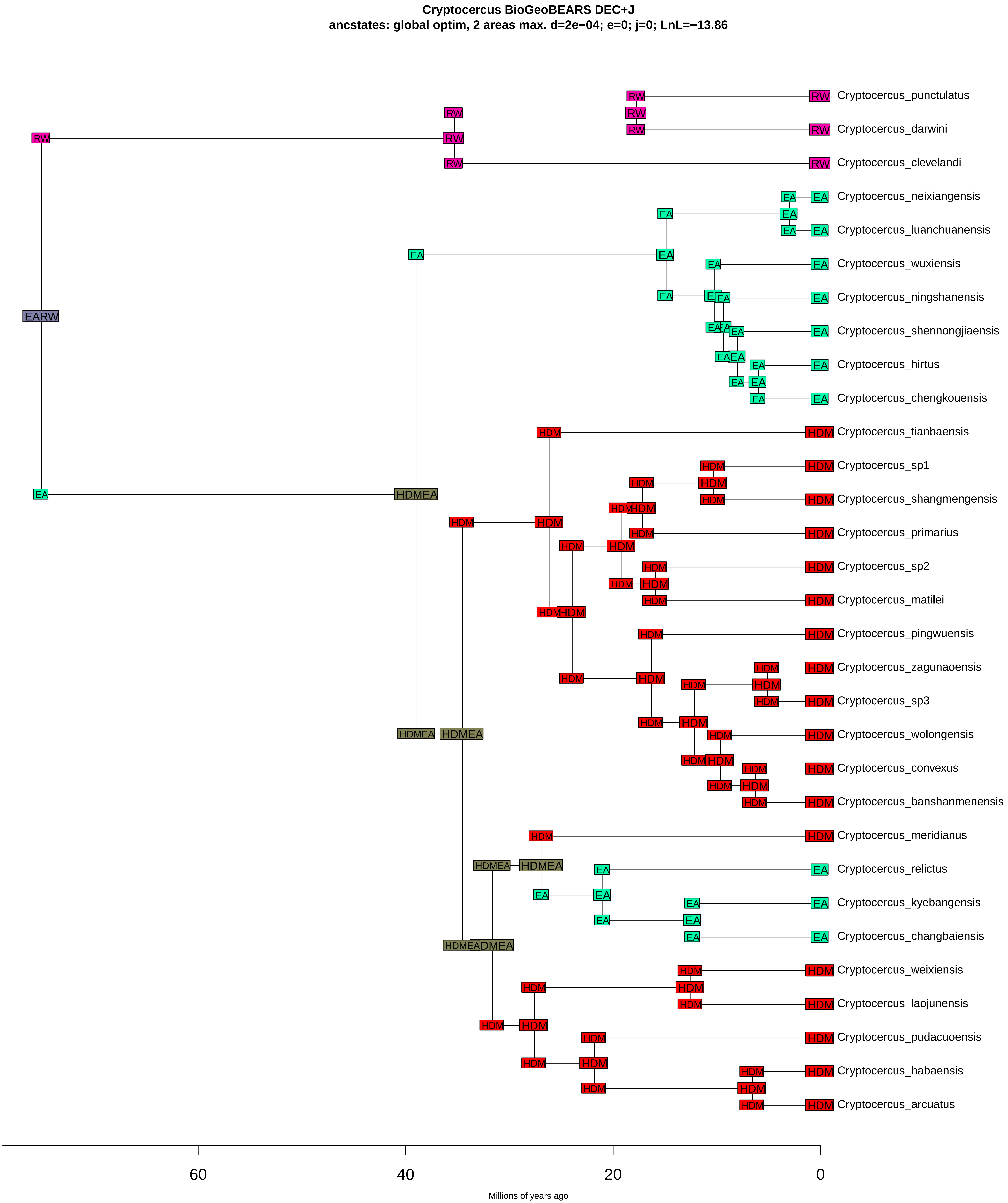
Cryptocercus BioGeoBEARS DEC+J
ancstates: global optim, 2 areas max. d=2e−04; e=0; j=0; LnL=−13.86
Cryptocercus_punctulatus
Cryptocercus_darwini
Cryptocercus_clevelandi
Cryptocercus_neixiangensis
Cryptocercus_luanchuanensis
Cryptocercus_wuxiensis
Cryptocercus_ningshanensis
Cryptocercus_shennongjiaensis
Cryptocercus_hirtus
Cryptocercus_chengkouensis
Cryptocercus_tianbaensis
Cryptocercus_sp1
Cryptocercus_shangmengensis
Cryptocercus_primarius
Cryptocercus_sp2
Cryptocercus_matilei
Cryptocercus_pingwuensis
Cryptocercus_zagunaoensis
Cryptocercus_sp3
Cryptocercus_wolongensis
Cryptocercus_convexus
Cryptocercus_banshanmenensis
Cryptocercus_meridianus
Cryptocercus_relictus
Cryptocercus_kyebangensis
Cryptocercus_changbaiensis
Cryptocercus_weixiensis
Cryptocercus_laojunensis
Cryptocercus_pudacuoensis
Cryptocercus_habaensis
Cryptocercus_arcuatus
60
40
20
0
Millions of years ago

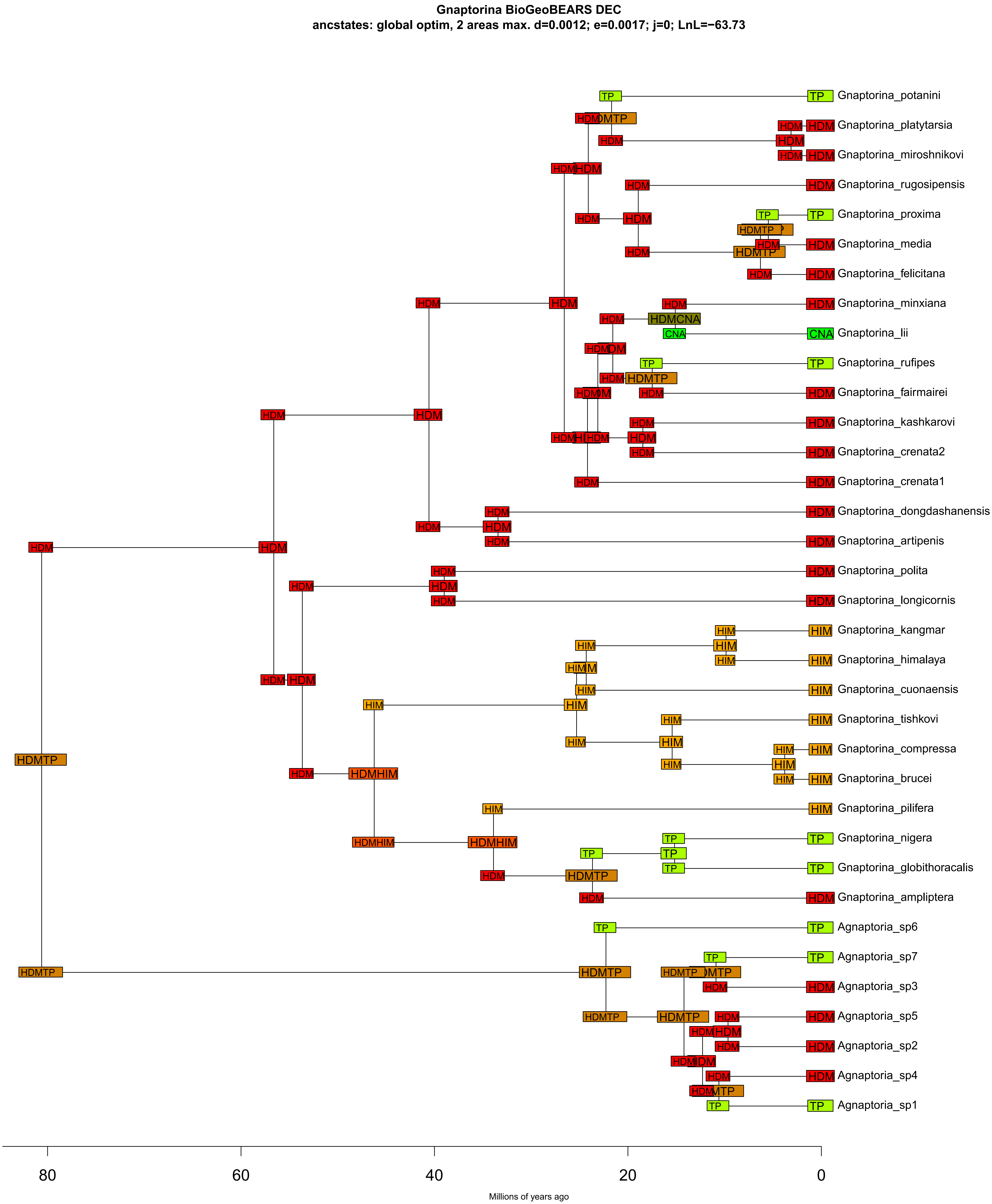

Gnaptorina BioGeoBEARS DEC
ancstates: global optim, 2 areas max. d=0.0012; e=0.0017; j=0; LnL=−63.73
Gnaptorina_potanini
Gnaptorina_platytarsia
Gnaptorina_miroshnikovi
Gnaptorina_rugosipensis
Gnaptorina_proxima
Gnaptorina_media
Gnaptorina_felicitana
Gnaptorina_minxiana
Gnaptorina_lii
Gnaptorina_rufipes
Gnaptorina_fairmairei
Gnaptorina_kashkarovi
Gnaptorina_crenata2
Gnaptorina_crenata1
Gnaptorina_dongdashanensis
Gnaptorina_artipenis
Gnaptorina_polita
Gnaptorina_longicornis
Gnaptorina_kangmar
Gnaptorina_himalaya
Gnaptorina_cuonaensis
Gnaptorina_tishkovi
Gnaptorina_compressa
Gnaptorina_brucei
Gnaptorina_pilifera
Gnaptorina_nigera
Gnaptorina_globithoracalis
Gnaptorina_ampliptera
Agnaptoria_sp6
Agnaptoria_sp7
Agnaptoria_sp3
Agnaptoria_sp5
Agnaptoria_sp2
Agnaptoria_sp4
Agnaptoria_sp1
80
60
40
20
0
Millions of years ago

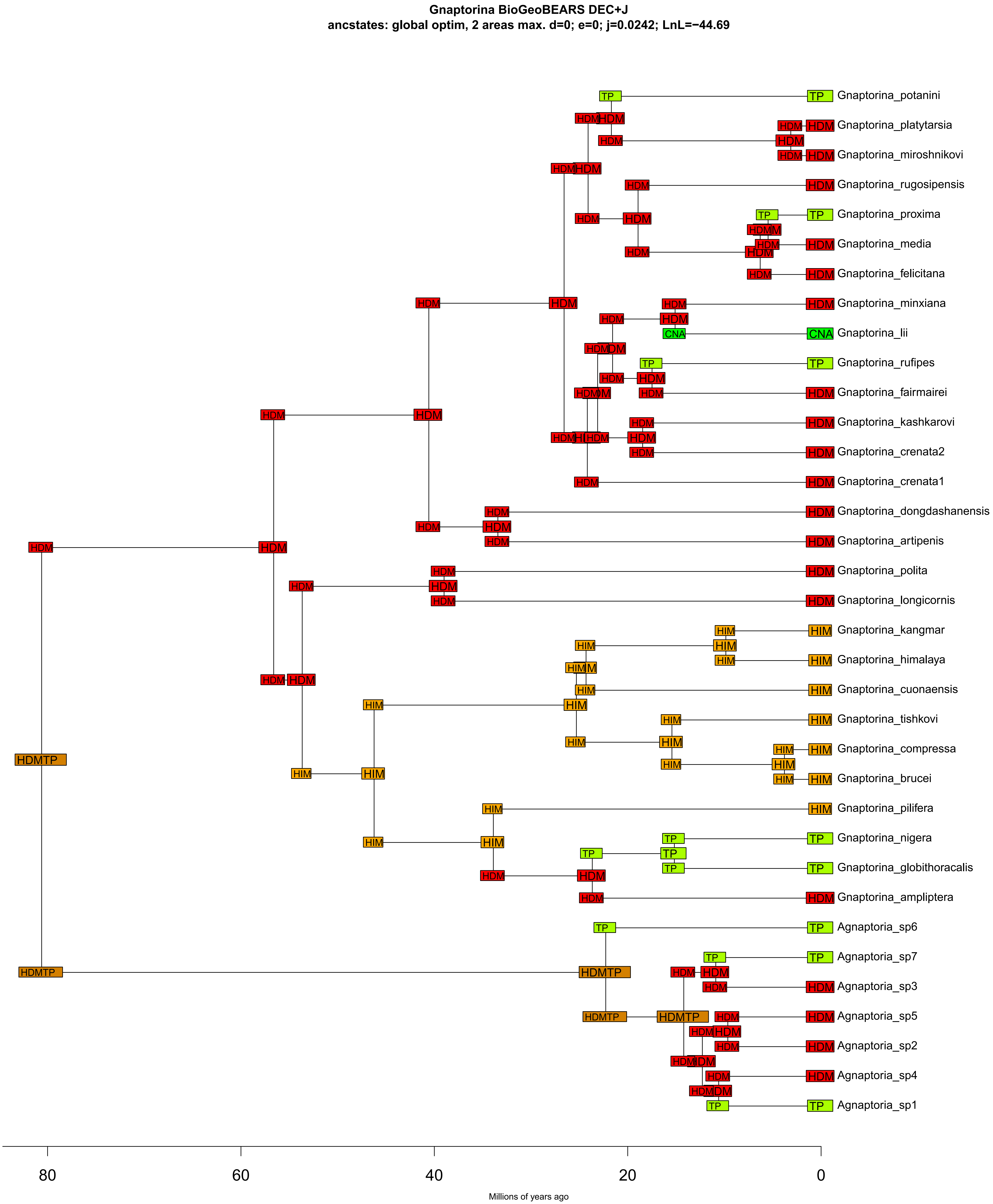
Gnaptorina BioGeoBEARS DEC+J
ancstates: global optim, 2 areas max. d=0; e=0; j=0.0242; LnL=−44.69
TP Gnaptorina_potanini
HDM Gnaptorina_platytarsia
HDM Gnaptorina_miroshnikovi
HDM Gnaptorina_rugosipensis
TP Gnaptorina_proxima
HDM Gnaptorina_media
HDM Gnaptorina_felicitana
HDM Gnaptorina_minxiana
CNA Gnaptorina_lii
TP Gnaptorina_rufipes
HDM Gnaptorina_fairmairei
HDM Gnaptorina_kashkarovi
HDM Gnaptorina_crenata2
HDM Gnaptorina_crenata1
HDM Gnaptorina_dongdashanensis
HDM Gnaptorina_artipenis
HDM Gnaptorina_polita
HDM Gnaptorina_longicornis
HIM Gnaptorina_kangmar
HIM Gnaptorina_himalaya
HIM Gnaptorina_cuonaensis
HIM Gnaptorina_tishkovi
HIM Gnaptorina_compressa
HIM Gnaptorina_brucei
HIM Gnaptorina_pilifera
TP Gnaptorina_nigera
TP Gnaptorina_globithoracalis
HDM Gnaptorina_ampliptera
TP Agnaptoria_sp6
TP Agnaptoria_sp7
HDM Agnaptoria_sp3
HDM Agnaptoria_sp5
HDM Agnaptoria_sp2
HDM Agnaptoria_sp4
TP Agnaptoria_sp1
HDMTP
80
60
40
20
0
Millions of years ago

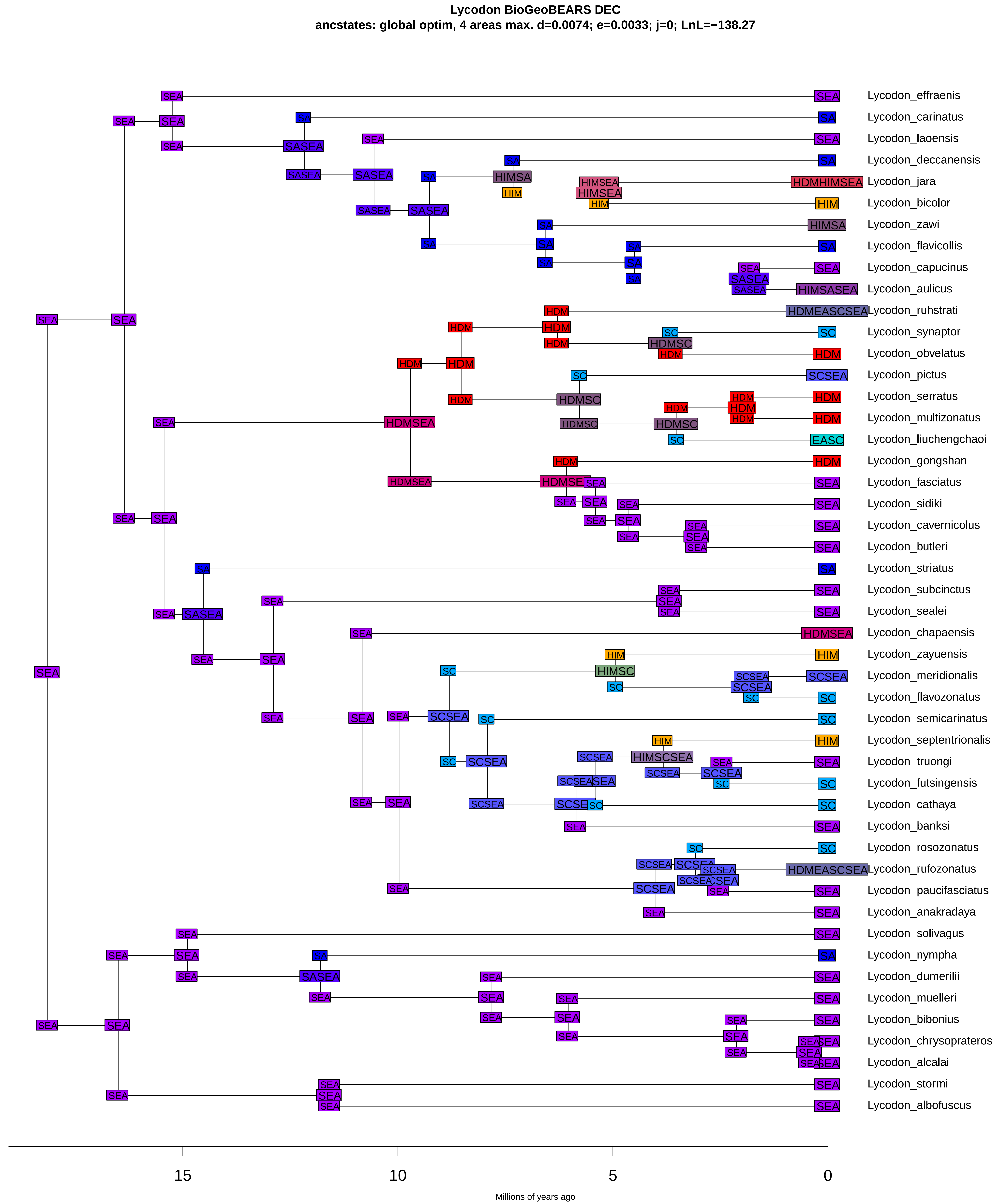
Lycodon BioGeoBEARS DEC
ancstates: global optim, 4 areas max. d=0.0074; e=0.0033; j=0; LnL=−138.27
Lycodon_effraenis
Lycodon_carinatus
Lycodon_laoensis
Lycodon_deccanensis
Lycodon_jara
Lycodon_bicolor
Lycodon_zawi
Lycodon_flavicollis
Lycodon_capucinus
Lycodon_aulicus
Lycodon_ruhstrati
Lycodon_synaptor
Lycodon_obvelatus
Lycodon_pictus
Lycodon_serratus
Lycodon_multizonatus
Lycodon_liuchengchaoi
Lycodon_gongshan
Lycodon_fasciatus
Lycodon_sidiki
Lycodon_cavernicolus
Lycodon_butleri
Lycodon_striatus
Lycodon_subcinctus
Lycodon_sealei
Lycodon_chapaensis
Lycodon_zayuensis
Lycodon_meridionalis
Lycodon_flavozonatus
Lycodon_semicarinatus
Lycodon_septentrionalis
Lycodon_truongi
Lycodon_futsingensis
Lycodon_cathaya
Lycodon_banksi
Lycodon_rosozonatus
Lycodon_rufozonatus
Lycodon_paucifasciatus
Lycodon_anakradaya
Lycodon_solivagus
Lycodon_nympha
Lycodon_dumerilii
Lycodon_muelleri
Lycodon_bibonius
Lycodon_chrysoprateros
Lycodon_alcalai
Lycodon_stormi
Lycodon_albofuscus
15
10
5
0
Millions of years ago

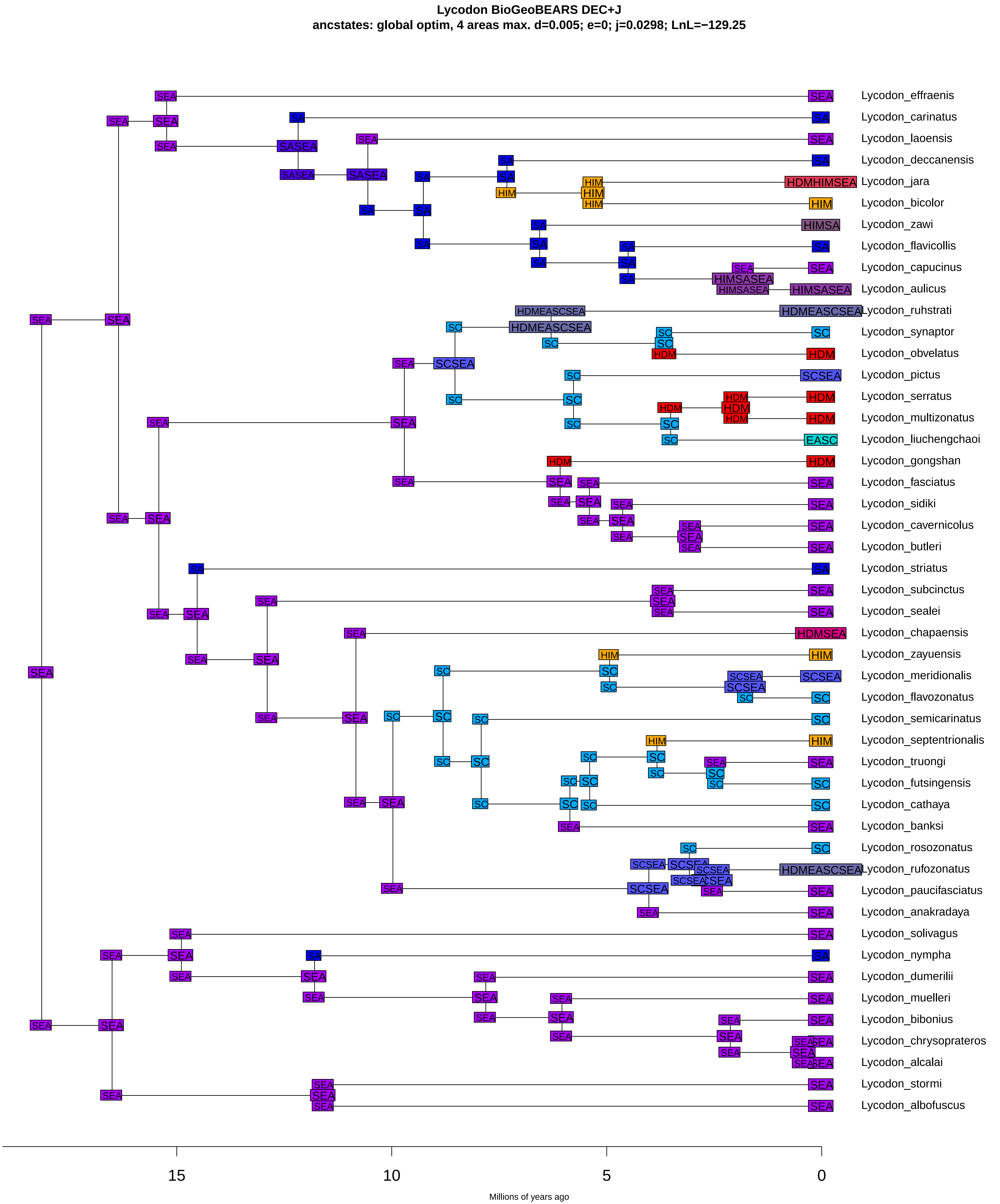
Lycodon BioGeoBEARS DEC+J
ancstates: global optim, 4 areas max. d=0.005; e=0; j=0.0298; LnL=−129.25
Lycodon_effraenis
Lycodon_carinatus
Lycodon_laoensis
Lycodon_deccanensis
Lycodon_jara
Lycodon_bicolor
Lycodon_zawi
Lycodon_flavicollis
Lycodon_capucinus
Lycodon_aulicus
Lycodon_ruhstrati
Lycodon_synaptor
Lycodon_obvelatus
Lycodon_pictus
Lycodon_serratus
Lycodon_multizonatus
Lycodon_liuchengchaoi
Lycodon_gongshan
Lycodon_fasciatus
Lycodon_sidiki
Lycodon_cavernicolus
Lycodon_butleri
Lycodon_striatus
Lycodon_subcinctus
Lycodon_sealei
Lycodon_chapaensis
Lycodon_zayuensis
Lycodon_meridionalis
Lycodon_flavozonatus
Lycodon_semicarinatus
Lycodon_septentrionalis
Lycodon_truongi
Lycodon_futsingensis
Lycodon_cathaya
Lycodon_banksi
Lycodon_rosozonatus
Lycodon_rufozonatus
Lycodon_paucifasciatus
Lycodon_anakradaya
Lycodon_solivagus
Lycodon_nympha
Lycodon_dumerilii
Lycodon_muelleri
Lycodon_bibonius
Lycodon_chrysoprateros
Lycodon_alcalai
Lycodon_stormi
Lycodon_albofuscus
15
10
5
0
Millions of years ago

Natricidae BioGeoBEARS DEC
ancstates: global optim, 4 areas max. d=0.0019; e=0; j=0; LnL=−321.60

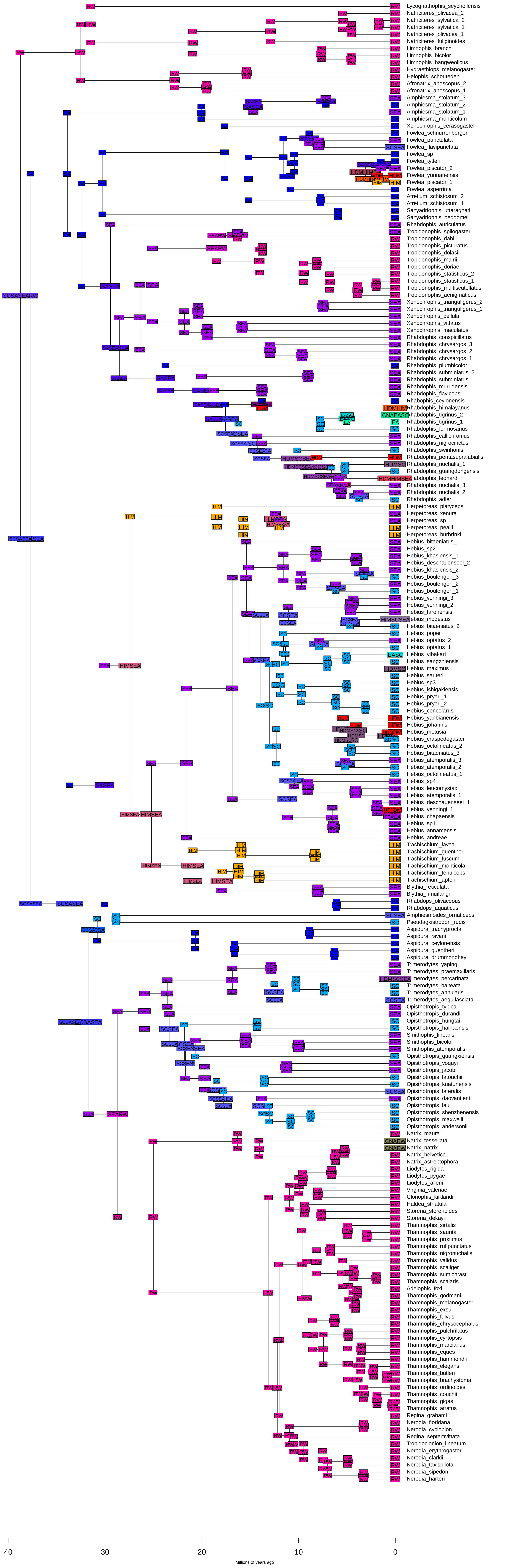

Natricidae BioGeoBEARS DEC+J
ancstates: global optim, 4 areas max. d=0.0011; e=0; j=0.0108; LnL=−299.04

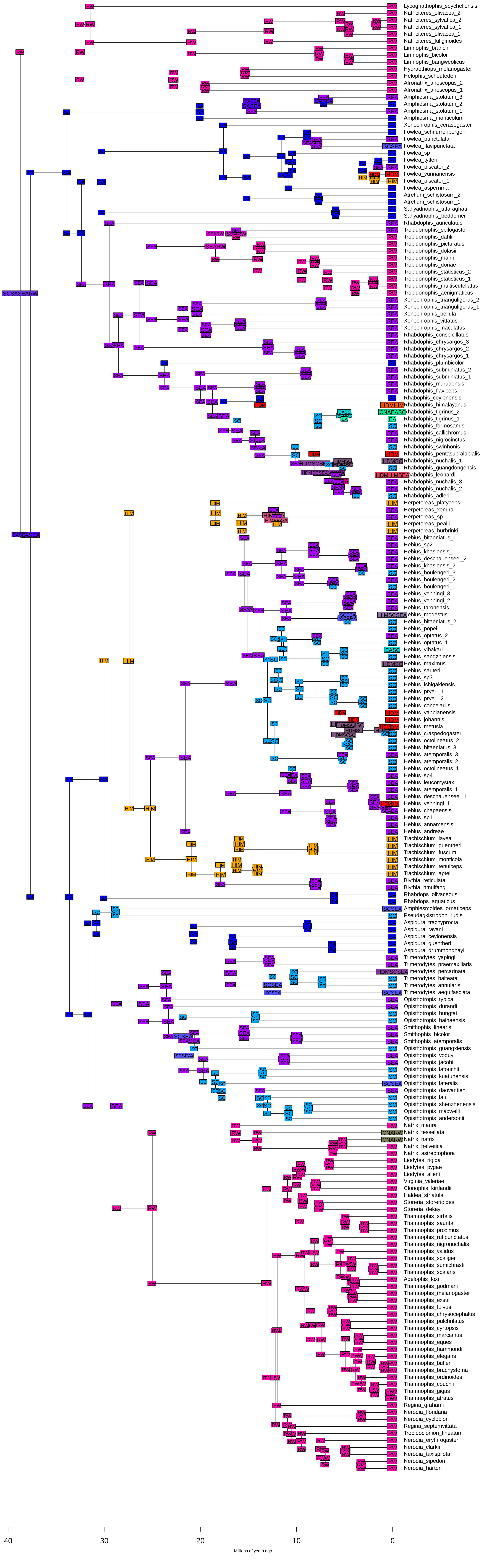

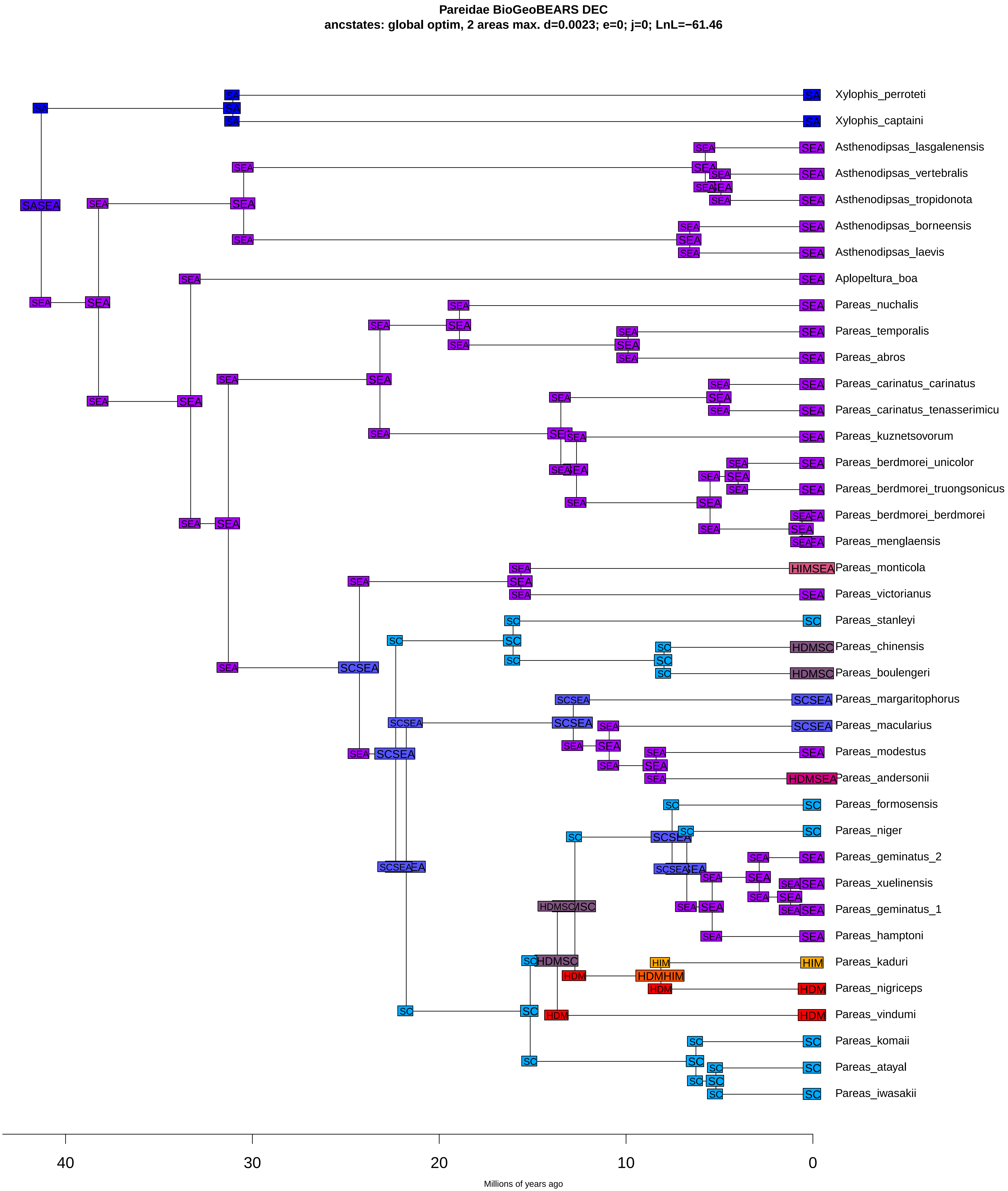
Pareidae BioGeoBEARS DEC
ancstates: global optim, 2 areas max. d=0.0023; e=0; j=0; LnL=−61.46
SA Xylophis_perroteti
SA Xylophis_captaini
SEA Asthenodipsas_lasgalenensis
SEA Asthenodipsas_vertebralis
SEA Asthenodipsas_tropidonota
SEA Asthenodipsas_borneensis
SEA Asthenodipsas_laevis
SEA Aplopeltura_boa
SEA Pareas_nuchalis
SEA Pareas_temporalis
SEA Pareas_abros
SEA Pareas_carinatus_carinatus
SEA Pareas_carinatus_tenasserimicu
SEA Pareas_kuznetsovorum
SEA Pareas_berdmorei_unicolor
SEA Pareas_berdmorei_truongsonicus
SEA Pareas_berdmorei_berdmorei
SEA Pareas_menglaensis
HIMSEA Pareas_monticola
SEA Pareas_victorianus
SC Pareas_stanleyi
HDMSC Pareas_chinensis
HDMSC Pareas_boulengeri
SCSEA Pareas_margaritophorus
SCSEA Pareas_macularius
SEA Pareas_modestus
HDMSEA Pareas_andersonii
SC Pareas_formosensis
SC Pareas_niger
SEA Pareas_geminatus_2
SEA Pareas_xuelinensis
SEA Pareas_geminatus_1
SEA Pareas_hamptoni
HIM Pareas_kaduri
HDM Pareas_nigriceps
HDM Pareas_vindumi
SC Pareas_komaii
SC Pareas_atayal
SC Pareas_iwasakii
40
30
20
10
0
Millions of years ago

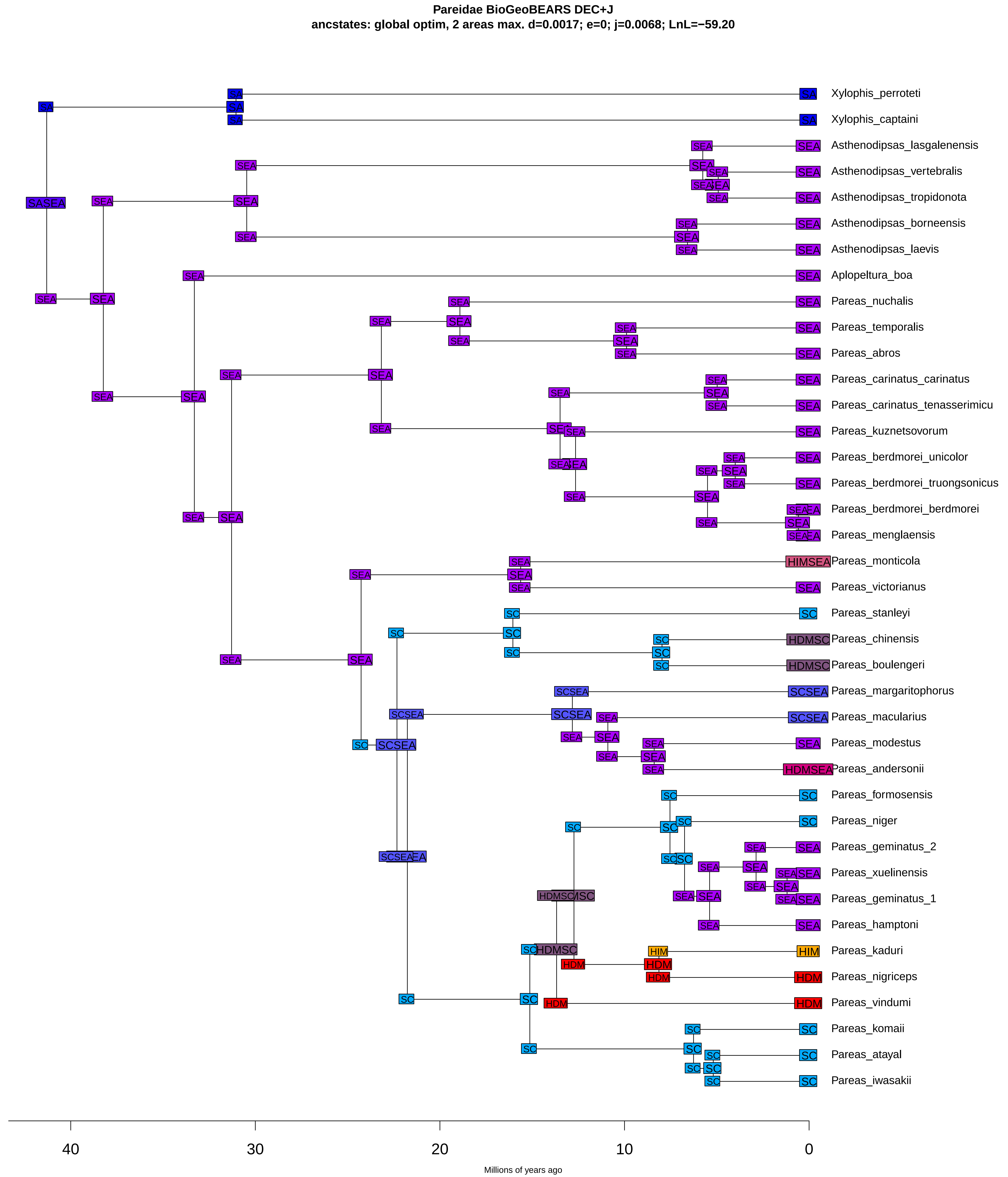
Pareidae BioGeoBEARS DEC+J
ancstates: global optim, 2 areas max. d=0.0017; e=0; j=0.0068; LnL=−59.20
Xylophis_perroteti
Xylophis_captaini
Asthenodipsas_lasgalenensis
Asthenodipsas_vertebralis
Asthenodipsas_tropidonota
Asthenodipsas_borneensis
Asthenodipsas_laevis
Aplopeltura_boa
Pareas_nuchalis
Pareas_temporalis
Pareas_abros
Pareas_carinatus_carinatus
Pareas_carinatus_tenasserimicu
Pareas_kuznetsovorum
Pareas_berdmorei_unicolor
Pareas_berdmorei_truongsonicus
Pareas_berdmorei_berdmorei
Pareas_menglaensis
Pareas_monticola
Pareas_victorianus
Pareas_stanleyi
Pareas_chinensis
Pareas_boulengeri
Pareas_margaritophorus
Pareas_macularius
Pareas_modestus
Pareas_andersonii
Pareas_formosensis
Pareas_niger
Pareas_geminatus_2
Pareas_xuelinensis
Pareas_geminatus_1
Pareas_hamptoni
Pareas_kaduri
Pareas_nigriceps
Pareas_vindumi
Pareas_komaii
Pareas_atayal
Pareas_iwasakii
40
30
20
10
0
Millions of years ago

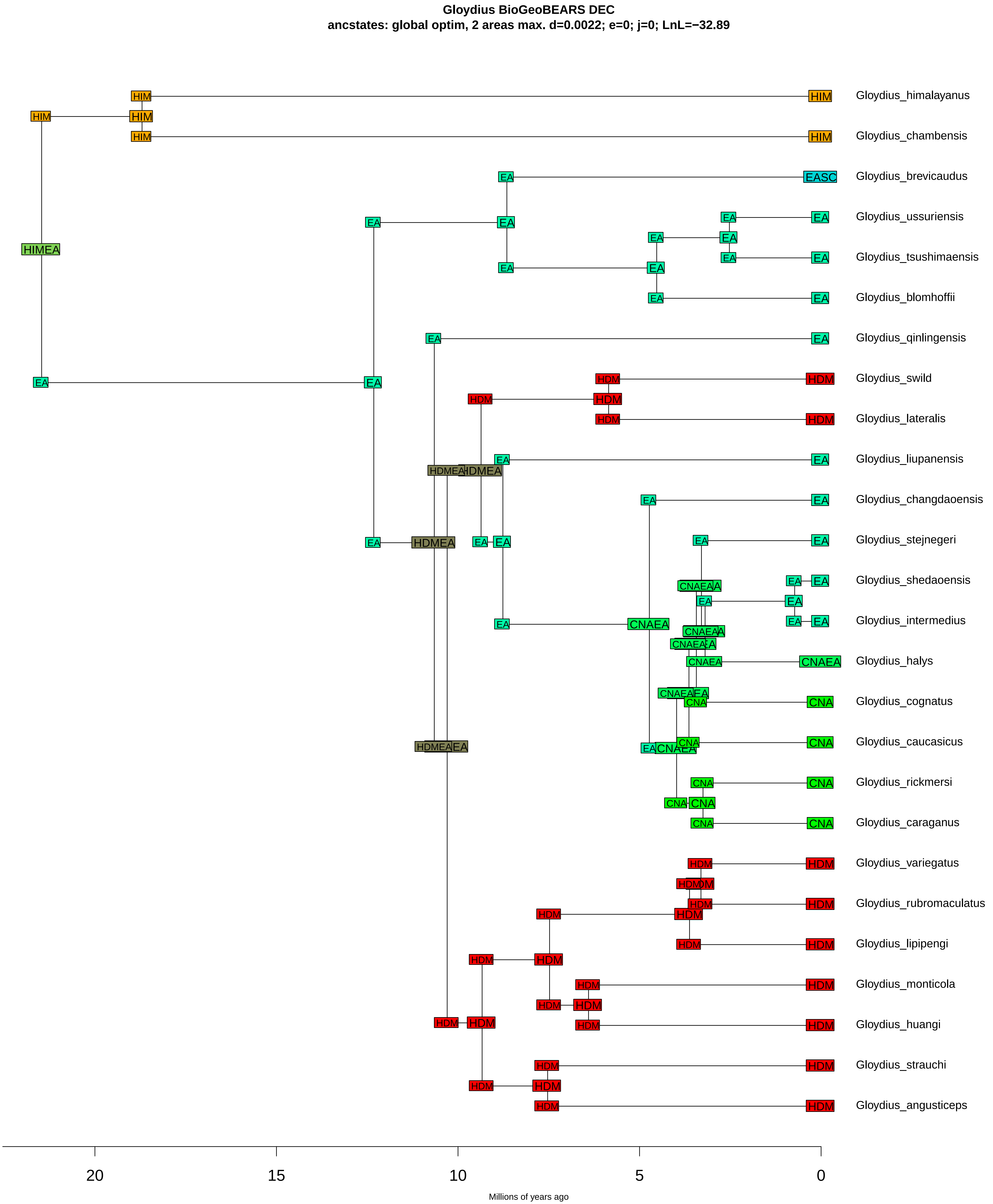
Gloydius BioGeoBEARS DEC
ancstates: global optim, 2 areas max. d=0.0022; e=0; j=0; LnL=−32.89
Gloydius_himalayanus
Gloydius_chambensis
Gloydius_brevicaudus
Gloydius_ussuriensis
Gloydius_tsushimaensis
Gloydius_blomhoffii
Gloydius_qinlingensis
Gloydius_swild
Gloydius_lateralis
Gloydius_liupanensis
Gloydius_changdaoensis
Gloydius_stejnegeri
Gloydius_shedaoensis
Gloydius_intermedius
Gloydius_halys
Gloydius_cognatus
Gloydius_caucasicus
Gloydius_rickmersi
Gloydius_caraganus
Gloydius_variegatus
Gloydius_rubromaculatus
Gloydius_lipipengi
Gloydius_monticola
Gloydius_huangi
Gloydius_strauchi
Gloydius_angusticeps
20
15
10
5
0
Millions of years ago

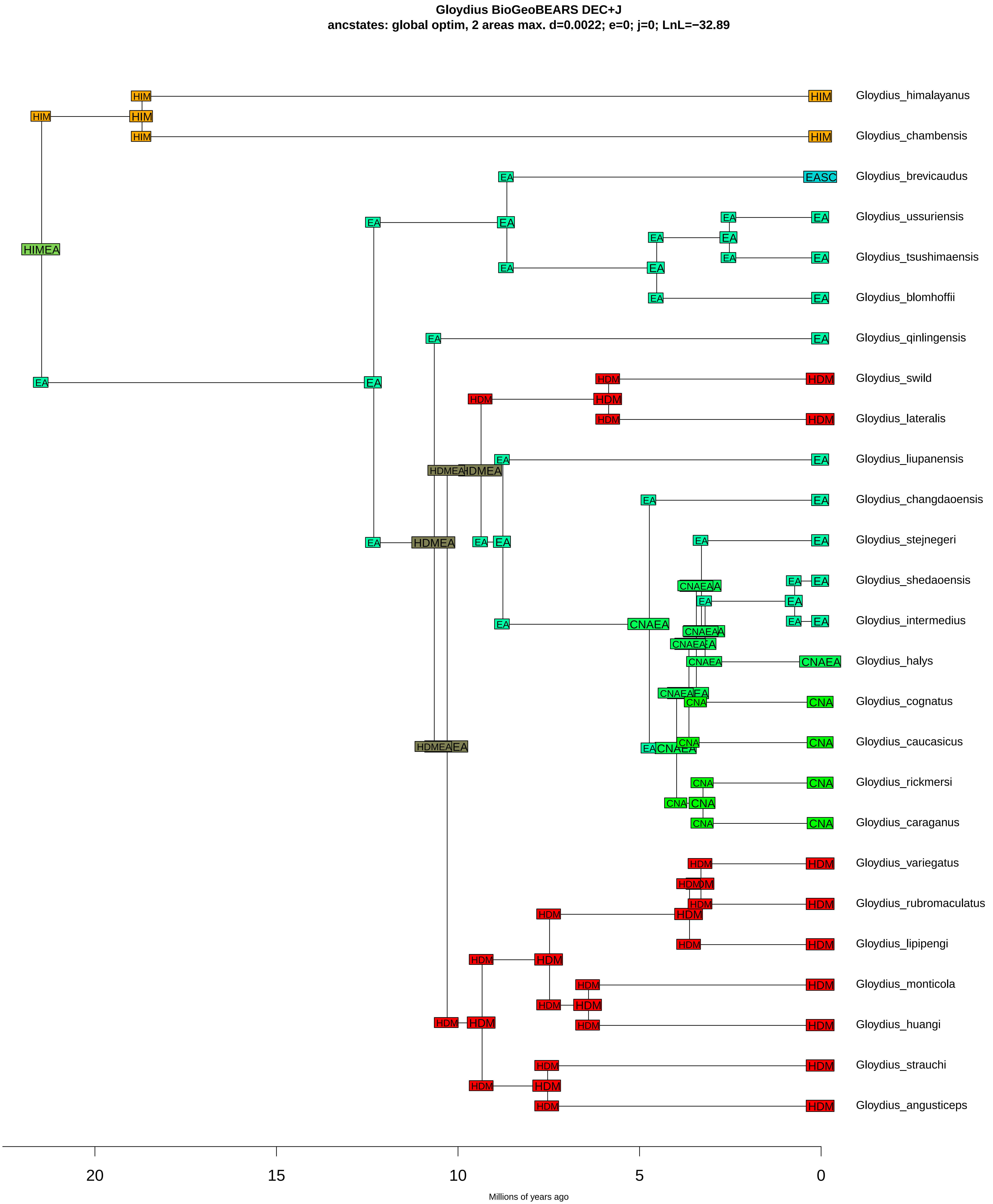
Gloydius BioGeoBEARS DEC+J
ancstates: global optim, 2 areas max. d=0.0022; e=0; j=0; LnL=−32.89
Gloydius_himalayanus
Gloydius_chambensis
Gloydius_brevicaudus
Gloydius_ussuriensis
Gloydius_tsushimaensis
Gloydius_blomhoffii
Gloydius_qinlingensis
Gloydius_swild
Gloydius_lateralis
Gloydius_liupanensis
Gloydius_changdaoensis
Gloydius_stejnegeri
Gloydius_shedaoensis
Gloydius_intermedius
Gloydius_halys
Gloydius_cognatus
Gloydius_caucasicus
Gloydius_rickmersi
Gloydius_caraganus
Gloydius_variegatus
Gloydius_rubromaculatus
Gloydius_lipipengi
Gloydius_monticola
Gloydius_huangi
Gloydius_strauchi
Gloydius_angusticeps
HIM
HIMEA
EA
EASC
HDM
HDMEA
CNAEA
CNA
20
15
10
5
0
Millions of years ago

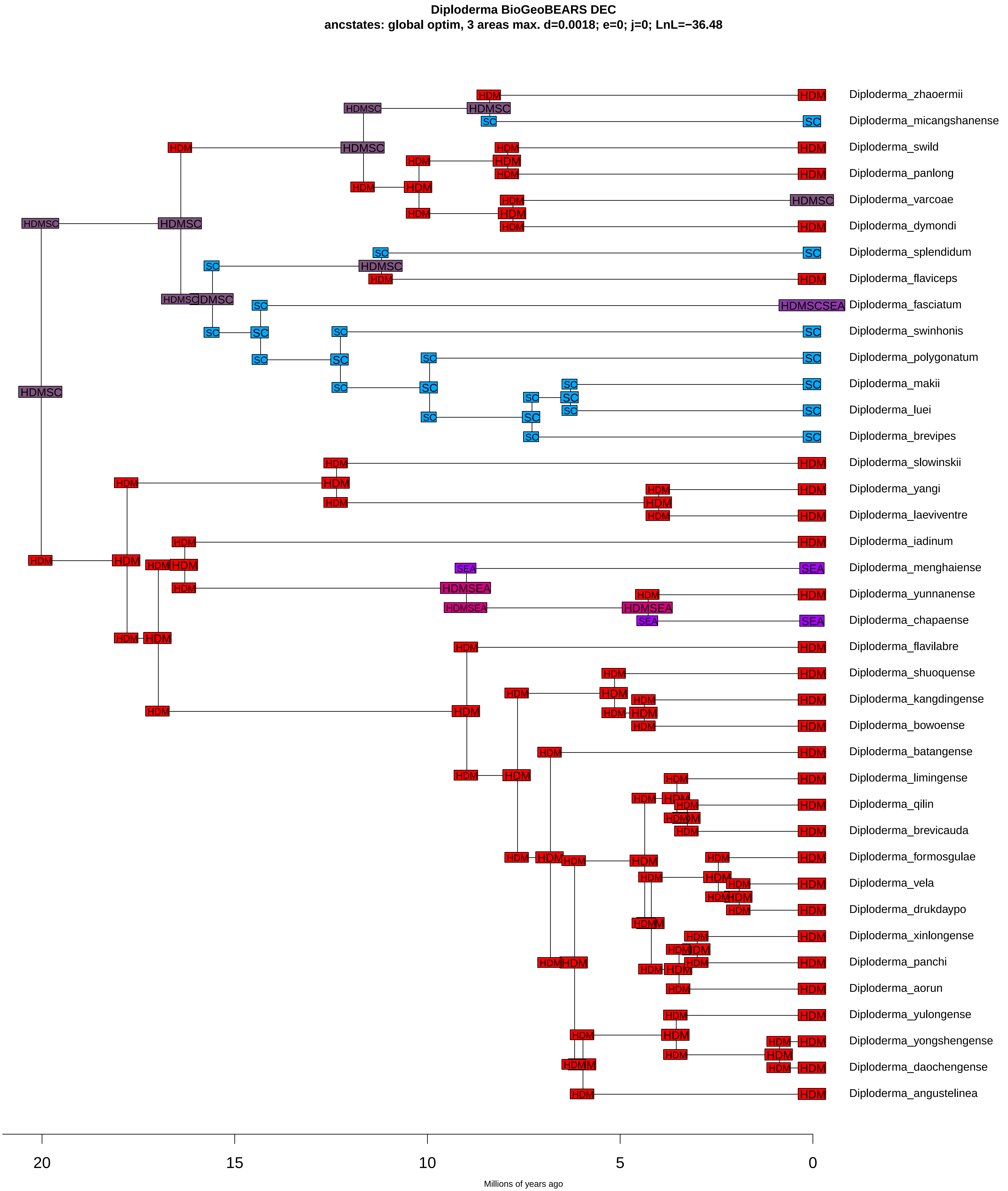
Diploderma BioGeoBEARS DEC
ancstates: global optim, 3 areas max. d=0.0018; e=0; j=0; LnL=−36.48
Diploderma_zhaoermii
Diploderma_micangshanense
Diploderma_swild
Diploderma_panlong
Diploderma_varcoae
Diploderma_dymondi
Diploderma_splendidum
Diploderma_flaviceps
Diploderma_fasciatum
Diploderma_swinhonis
Diploderma_polygonatum
Diploderma_makii
Diploderma_luei
Diploderma_brevipes
Diploderma_slowinskii
Diploderma_yangi
Diploderma_laeviventre
Diploderma_iadinum
Diploderma_menghaiense
Diploderma_yunnanense
Diploderma_chapaense
Diploderma_flavilabre
Diploderma_shuoquense
Diploderma_kangdingense
Diploderma_bowoense
Diploderma_batangense
Diploderma_limingense
Diploderma_qilin
Diploderma_brevicauda
Diploderma_formosgulae
Diploderma_vela
Diploderma_drukdaypo
Diploderma_xinlongense
Diploderma_panchi
Diploderma_aorun
Diploderma_yulongense
Diploderma_yongshengense
Diploderma_daochengense
Diploderma_angustelinea
20
15
10
5
0
Millions of years ago

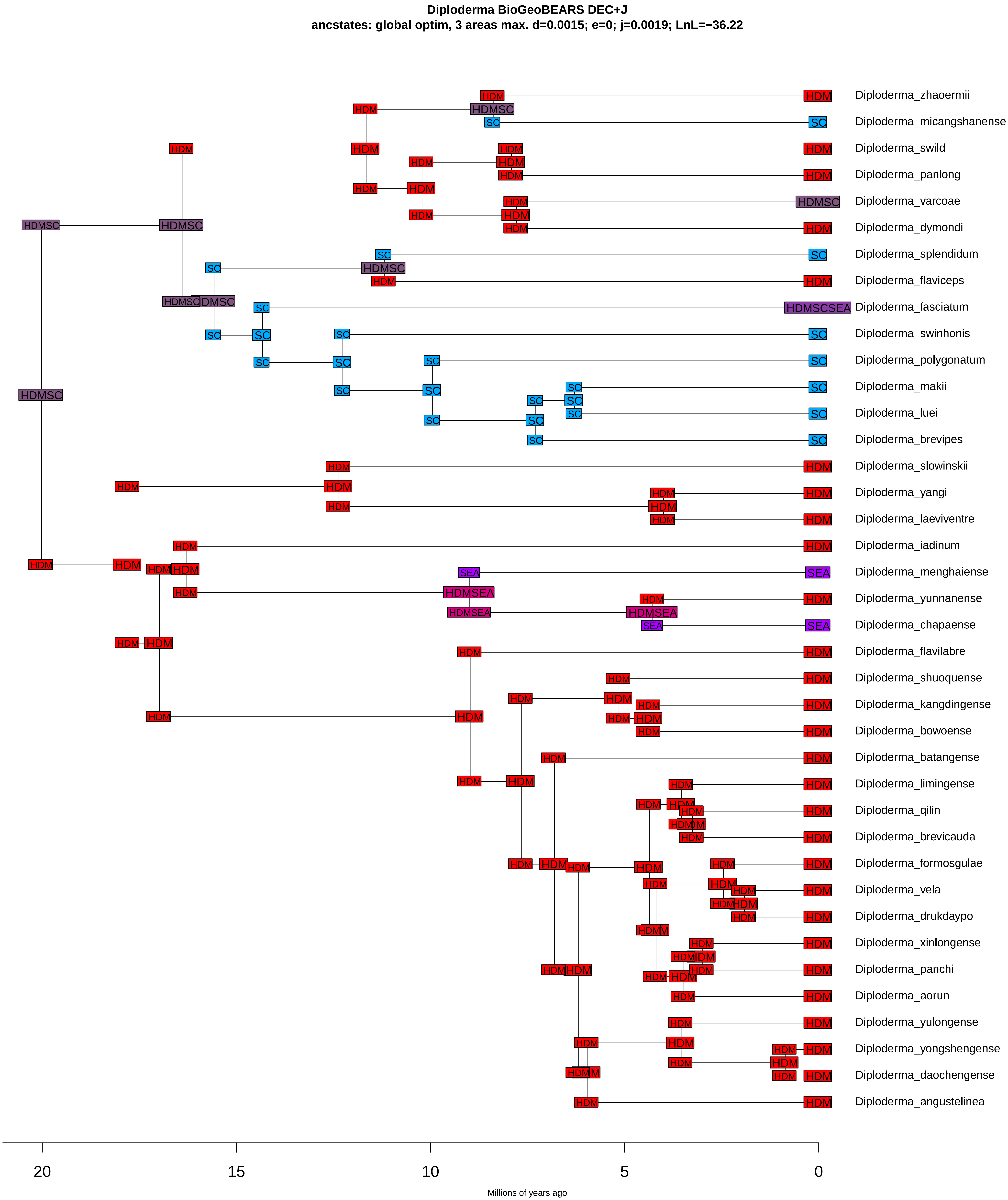

Diploderma BioGeoBEARS DEC+J
ancstates: global optim, 3 areas max. d=0.0015; e=0; j=0.0019; LnL=−36.22
Diploderma_zhaoermii
Diploderma_micangshanense
Diploderma_swild
Diploderma_panlong
Diploderma_varcoae
Diploderma_dymondi
Diploderma_splendidum
Diploderma_flaviceps
Diploderma_fasciatum
Diploderma_swinhonis
Diploderma_polygonatum
Diploderma_makii
Diploderma_luei
Diploderma_brevipes
Diploderma_slowinskii
Diploderma_yangi
Diploderma_laeviventre
Diploderma_iadinum
Diploderma_menghaiense
Diploderma_yunnanense
Diploderma_chapaense
Diploderma_flavilabre
Diploderma_shuoquense
Diploderma_kangdingense
Diploderma_bowoense
Diploderma_batangense
Diploderma_limingense
Diploderma_qilin
Diploderma_brevicauda
Diploderma_formosgulae
Diploderma_vela
Diploderma_drukdaypo
Diploderma_xinlongense
Diploderma_panchi
Diploderma_aorun
Diploderma_yulongense
Diploderma_yongshengense
Diploderma_daochengense
Diploderma_angustelinea
20
15
10
5
0
Millions of years ago

**Scincella BioGeoBEARS DEC**
**ancstates: global optim, 3 areas max. d=0.002; e=1e−04; j=0; LnL=−49.52**

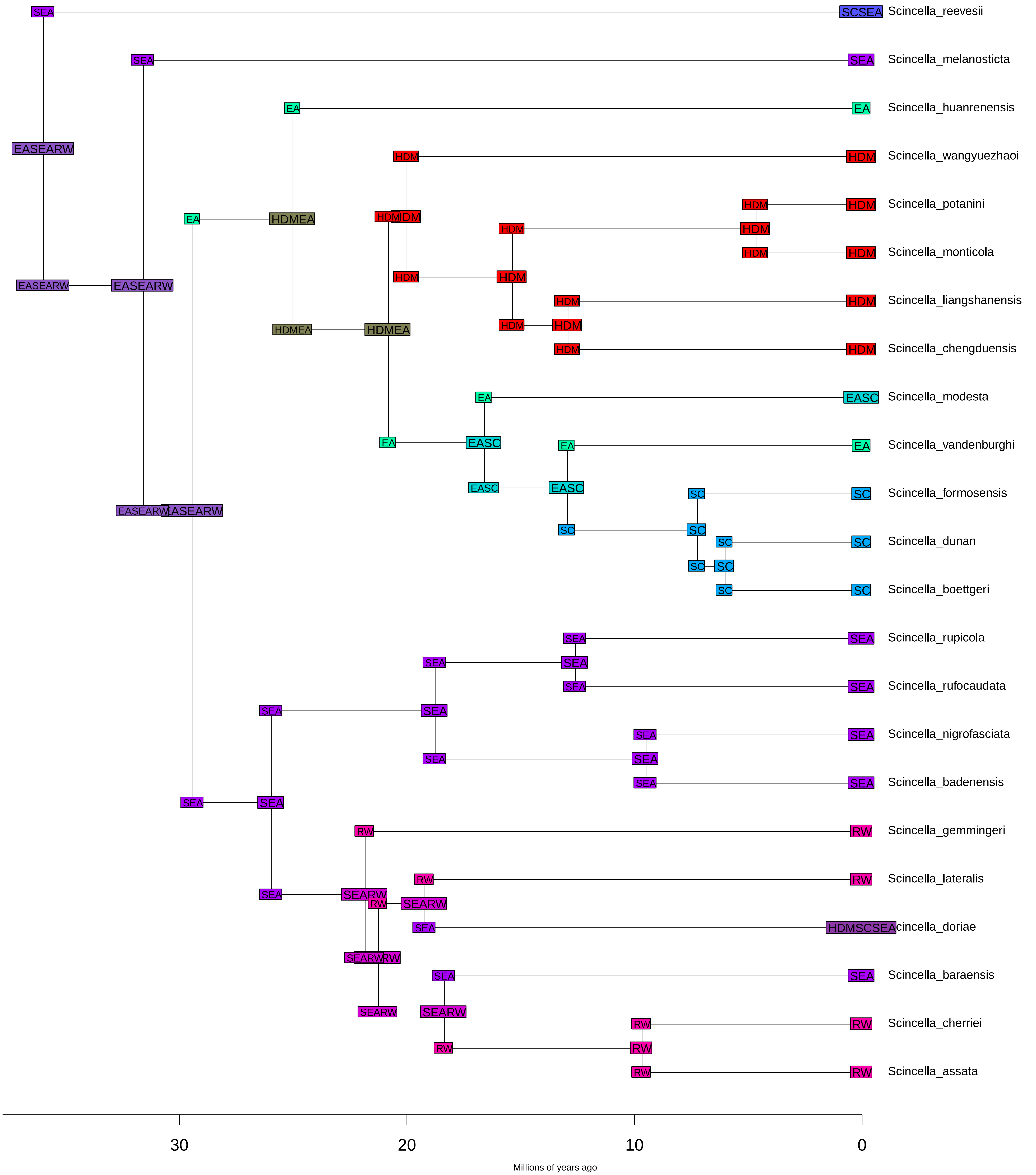

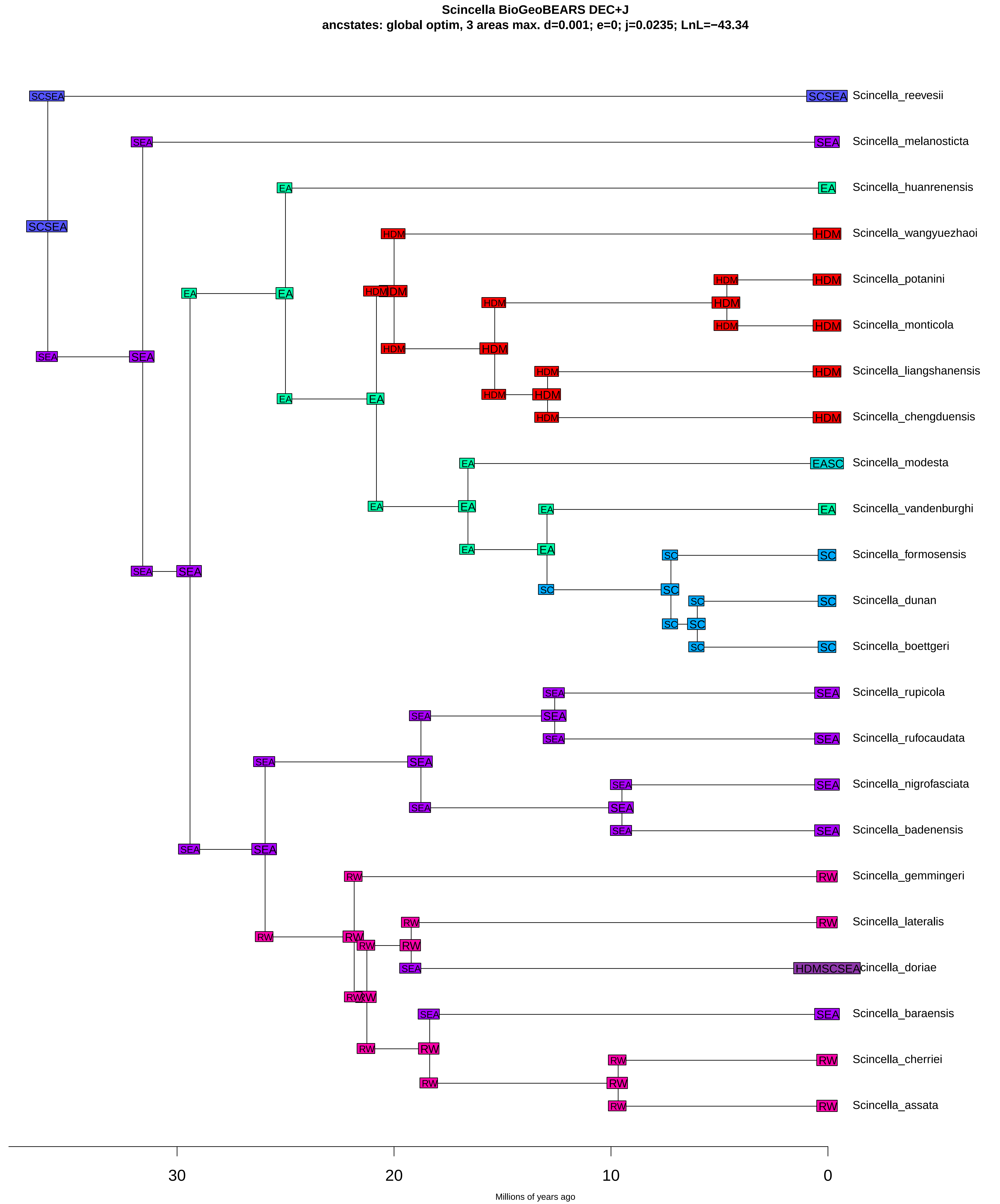

Scincella BioGeoBEARS DEC+J
ancstates: global optim, 3 areas max. d=0.001; e=0; j=0.0235; LnL=−43.34
Scincella_reevesii
Scincella_melanosticta
Scincella_huanrenensis
Scincella_wangyuezhaoi
Scincella_potanini
Scincella_monticola
Scincella_liangshanensis
Scincella_chengduensis
Scincella_modesta
Scincella_vandenburghi
Scincella_formosensis
Scincella_dunan
Scincella_boettgeri
Scincella_rupicola
Scincella_rufocaudata
Scincella_nigrofasciata
Scincella_badenensis
Scincella_gemmingeri
Scincella_lateralis
Scincella_doriae
Scincella_baraensis
Scincella_cherriei
Scincella_assata
SCSEA
SEA
EA
HDM
EASC
SC
RW
HDMSCSEA
30
20
10
0
Millions of years ago

**Ochotona BioGeoBEARS DEC**
**ancstates: global optim, 3 areas max. d=0.0092; e=7e−04; j=0; LnL=−102.91**

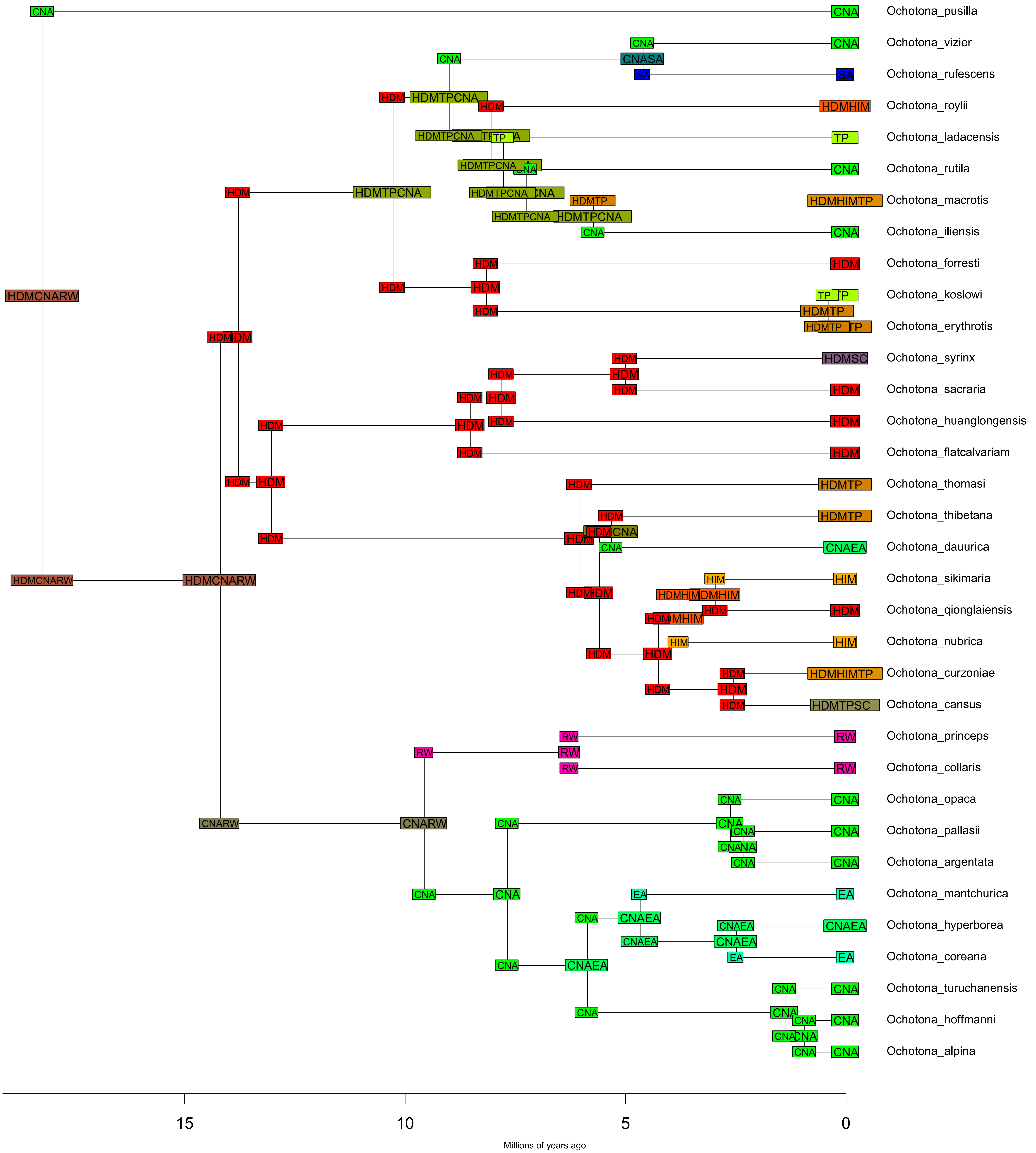

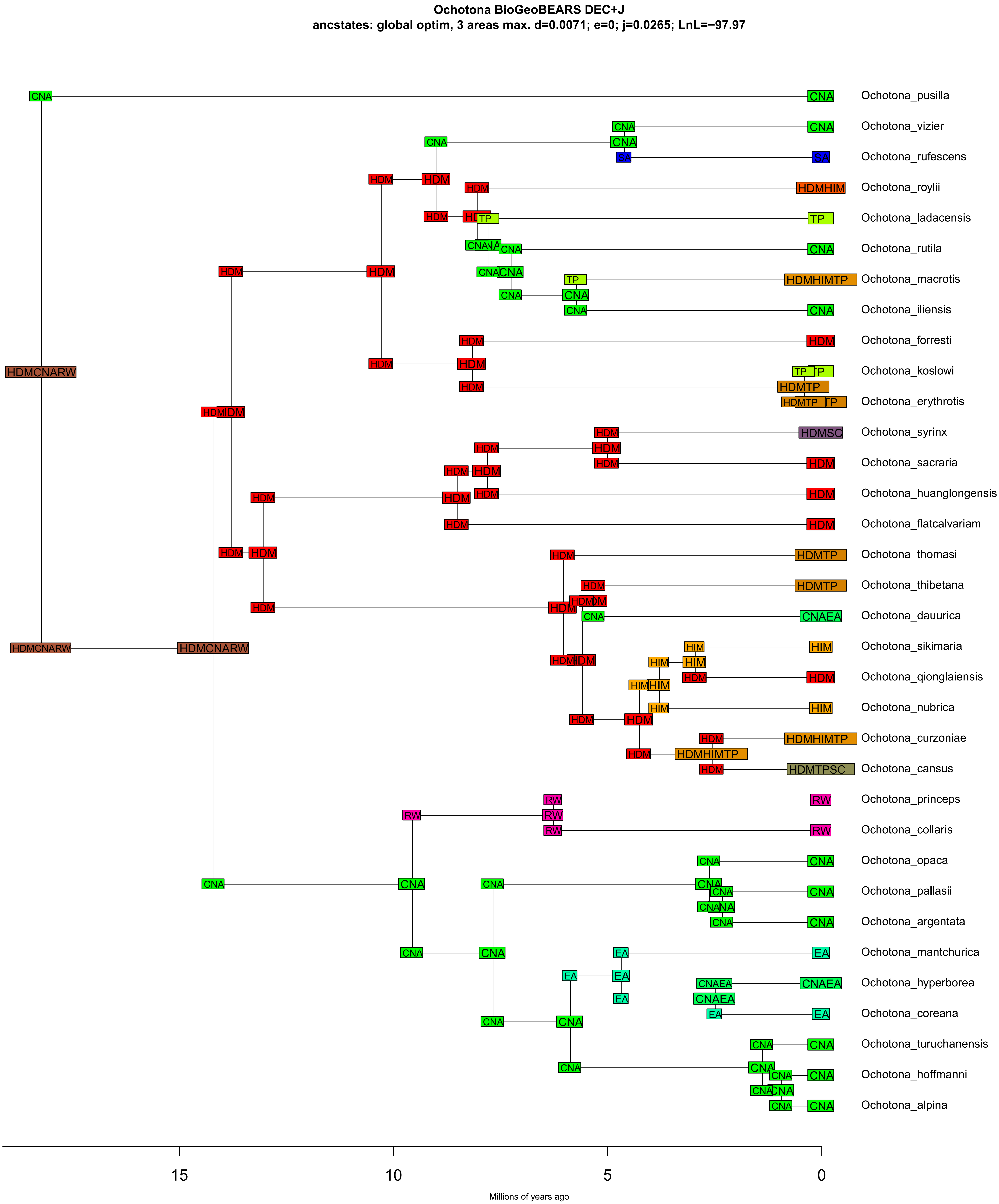
Ochotona BioGeoBEARS DEC+J
ancstates: global optim, 3 areas max. d=0.0071; e=0; j=0.0265; LnL=−97.97
Ochotona_pusilla
Ochotona_vizier
Ochotona_rufescens
Ochotona_roylii
Ochotona_ladacensis
Ochotona_rutila
Ochotona_macrotis
Ochotona_iliensis
Ochotona_forresti
Ochotona_koslowi
Ochotona_erythrotis
Ochotona_syrinx
Ochotona_sacraria
Ochotona_huanglongensis
Ochotona_flatcalvariam
Ochotona_thomasi
Ochotona_thibetana
Ochotona_dauurica
Ochotona_sikimaria
Ochotona_qionglaiensis
Ochotona_nubrica
Ochotona_curzoniae
Ochotona_cansus
Ochotona_princeps
Ochotona_collaris
Ochotona_opaca
Ochotona_pallasii
Ochotona_argentata
Ochotona_mantchurica
Ochotona_hyperborea
Ochotona_coreana
Ochotona_turuchanensis
Ochotona_hoffmanni
Ochotona_alpina
15
10
5
0
Millions of years ago

**Spalacidae BioGeoBEARS DEC**
**ancstates: global optim, 3 areas max. d=0.0074; e=0; j=0; LnL=−56.11**

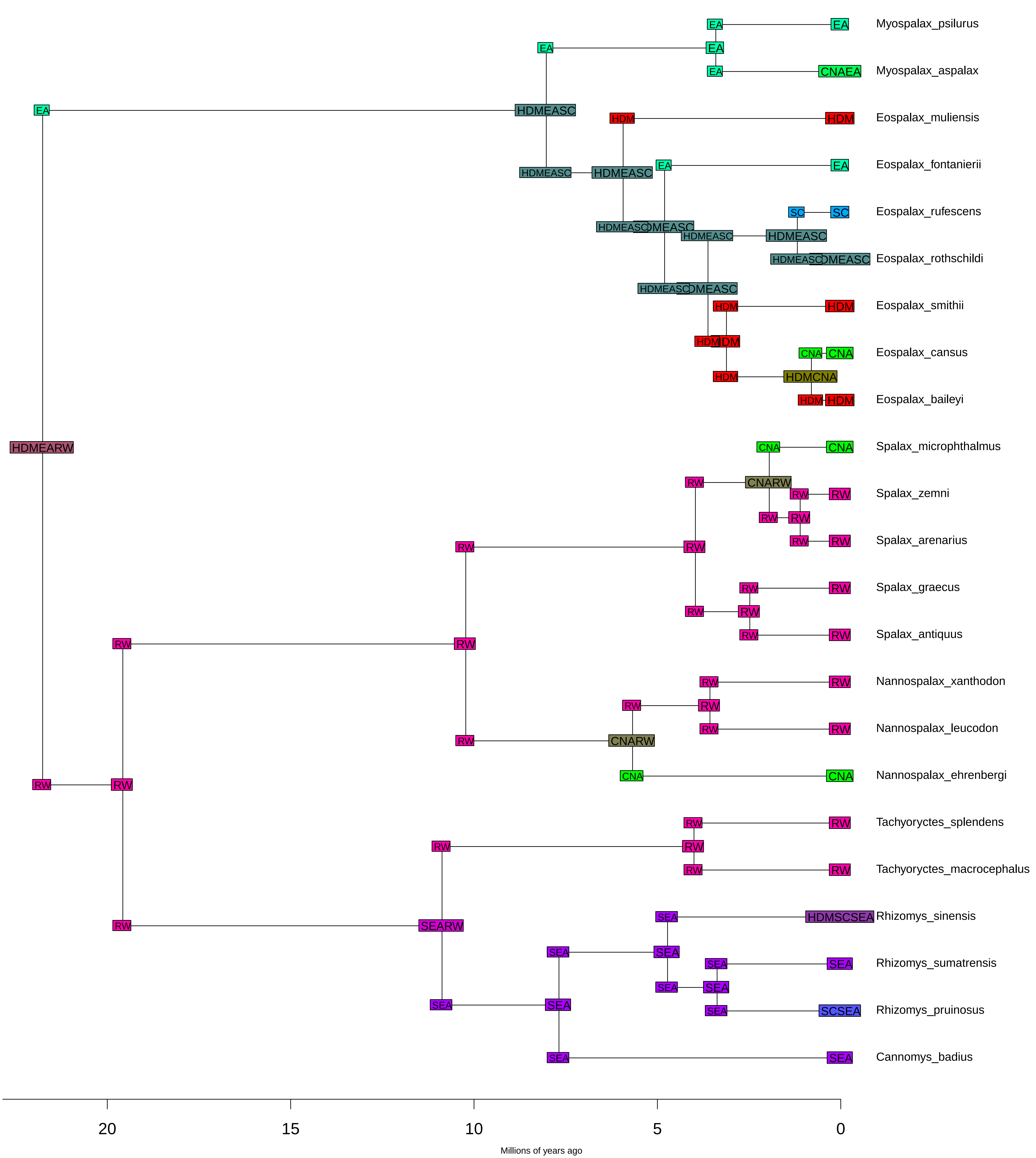

**Spalacidae BioGeoBEARS DEC+J**
**ancstates: global optim, 3 areas max. d=0.0052; e=0; j=0.0182; LnL=−53.25**

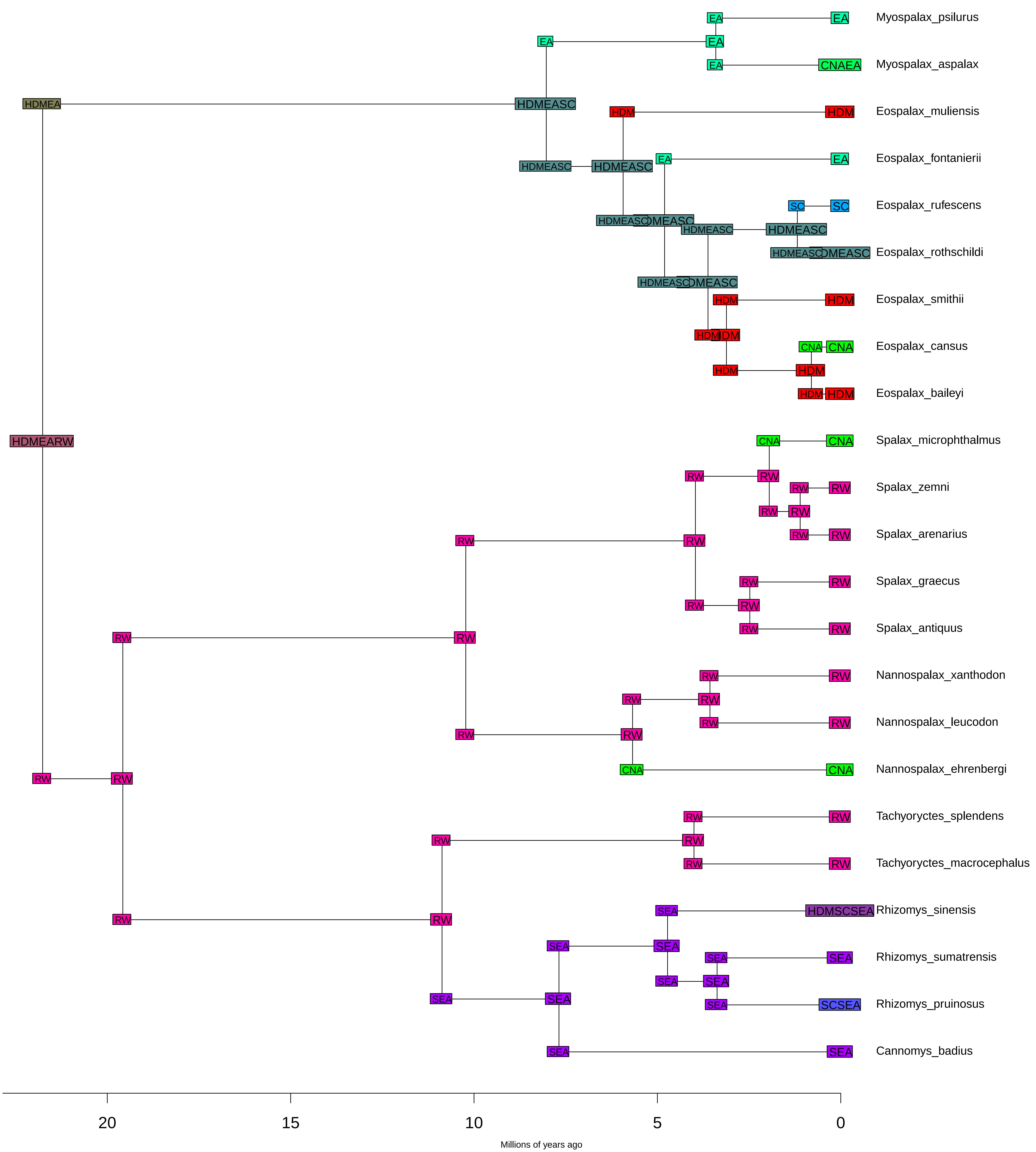

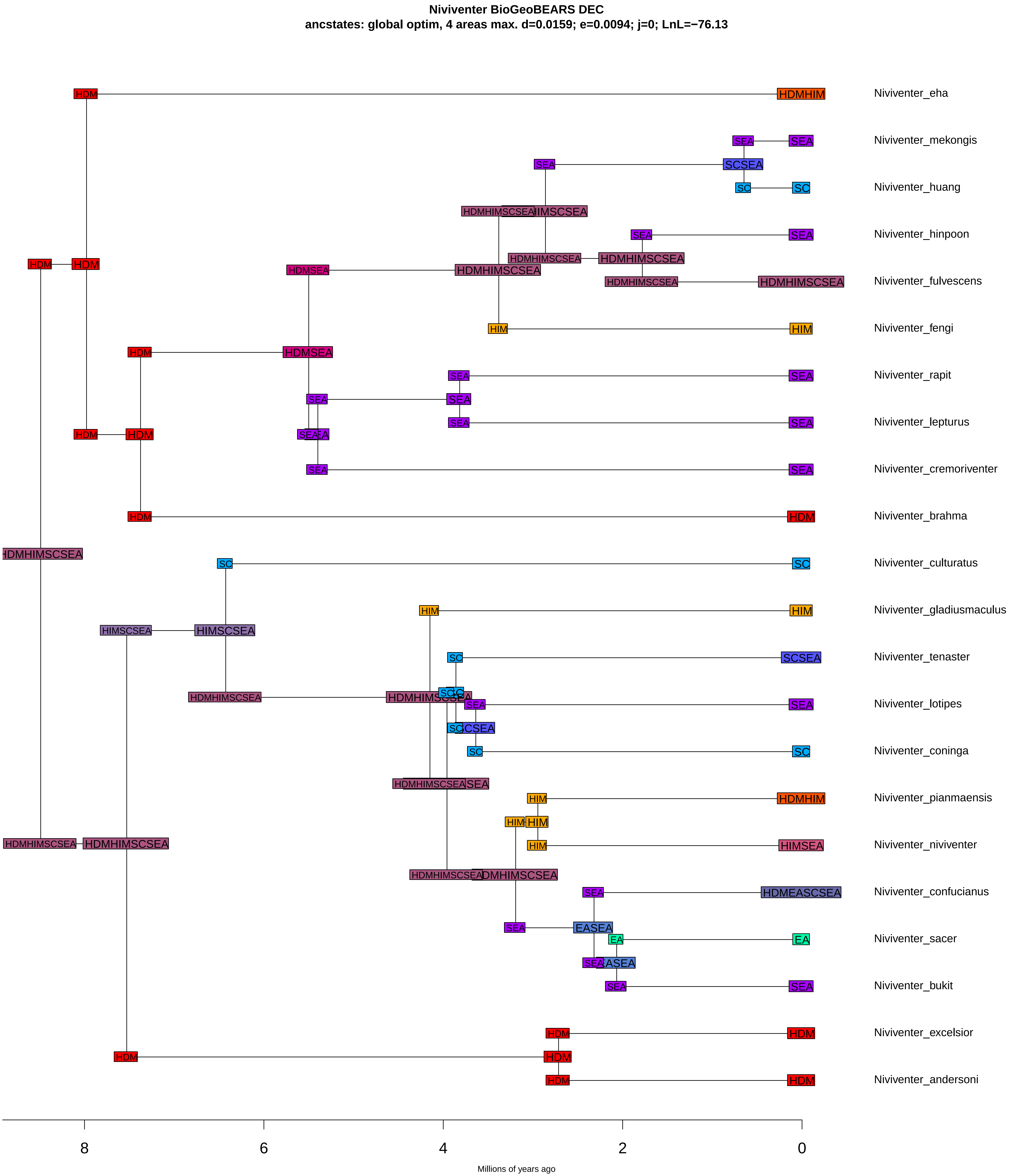

Niviventer BioGeoBEARS DEC
ancstates: global optim, 4 areas max. d=0.0159; e=0.0094; j=0; LnL=−76.13
Niviventer_eha
Niviventer_mekongis
Niviventer_huang
Niviventer_hinpoon
Niviventer_fulvescens
Niviventer_fengi
Niviventer_rapit
Niviventer_lepturus
Niviventer_cremoriventer
Niviventer_brahma
Niviventer_culturatus
Niviventer_gladiusmaculus
Niviventer_tenaster
Niviventer_lotipes
Niviventer_coninga
Niviventer_pianmaensis
Niviventer_niviventer
Niviventer_confucianus
Niviventer_sacer
Niviventer_bukit
Niviventer_excelsior
Niviventer_andersoni
8
6
4
2
0
Millions of years ago

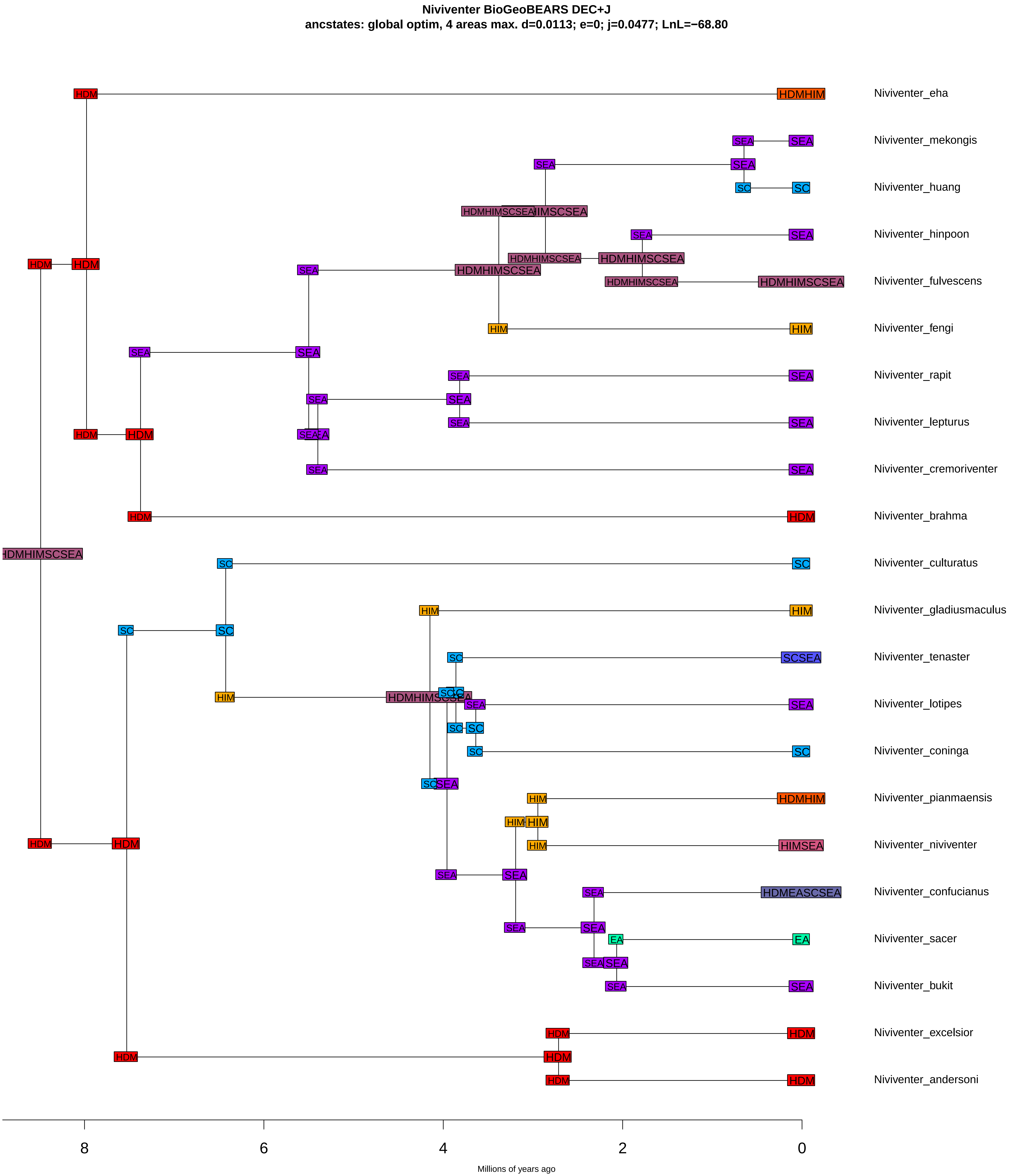
Niviventer BioGeoBEARS DEC+J
ancstates: global optim, 4 areas max. d=0.0113; e=0; j=0.0477; LnL=−68.80
Niviventer_eha
Niviventer_mekongis
Niviventer_huang
Niviventer_hinpoon
Niviventer_fulvescens
Niviventer_fengi
Niviventer_rapit
Niviventer_lepturus
Niviventer_cremoriventer
Niviventer_brahma
Niviventer_culturatus
Niviventer_gladiusmaculus
Niviventer_tenaster
Niviventer_lotipes
Niviventer_coninga
Niviventer_pianmaensis
Niviventer_niviventer
Niviventer_confucianus
Niviventer_sacer
Niviventer_bukit
Niviventer_excelsior
Niviventer_andersoni
HDMHIM
SEA
SC
HDMHIMSCSEA
HIM
HDM
SCSEA
HIMSEA
HDMEASCSEA
EA
8
6
4
2
0
Millions of years ago

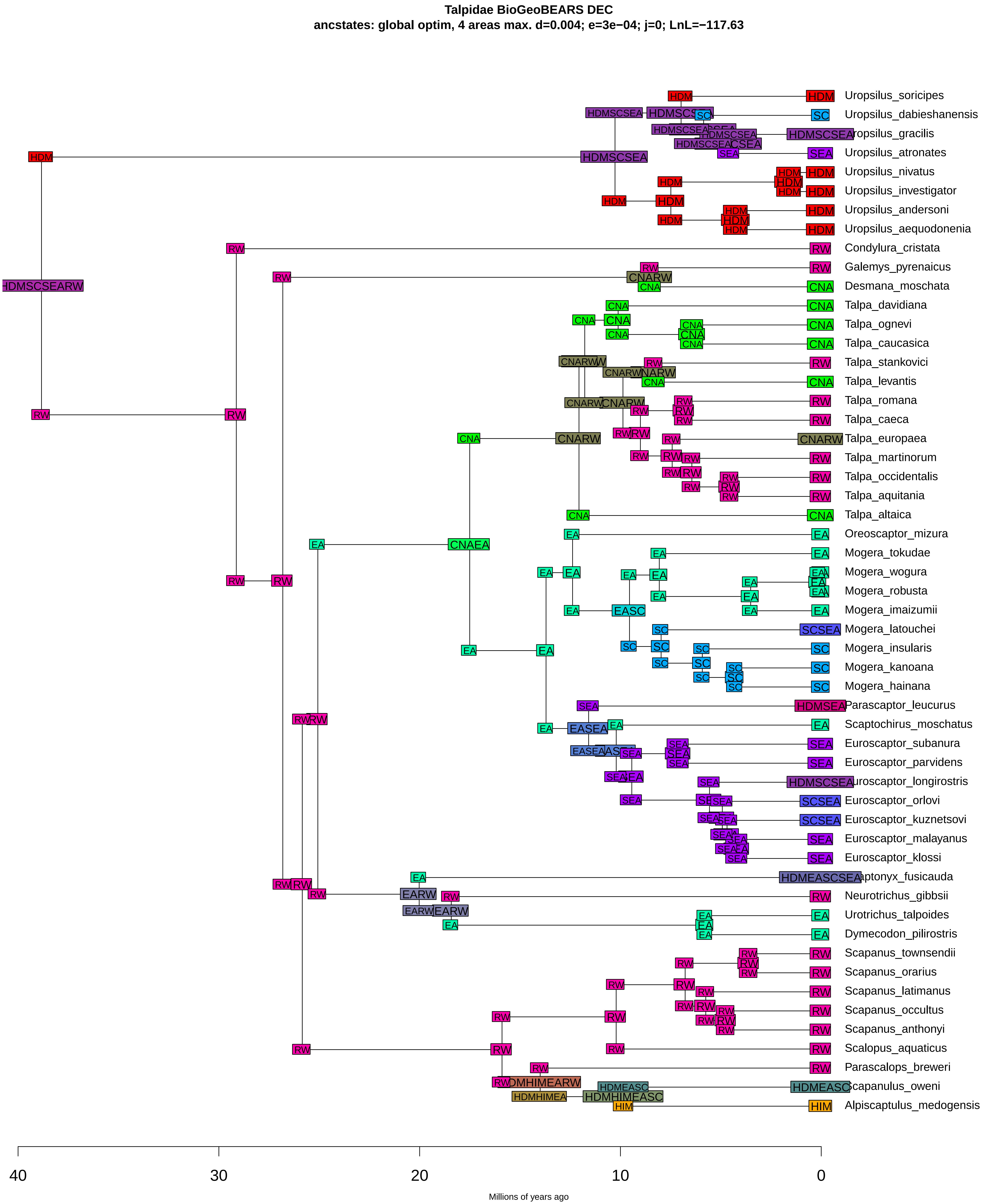
Talpidae BioGeoBEARS DEC
ancstates: global optim, 4 areas max. d=0.004; e=3e−04; j=0; LnL=−117.63
Uropsilus_soricipes
Uropsilus_dabieshanensis
Uropsilus_gracilis
Uropsilus_atronates
Uropsilus_nivatus
Uropsilus_investigator
Uropsilus_andersoni
Uropsilus_aequodonenia
Condylura_cristata
Galemys_pyrenaicus
Desmana_moschata
Talpa_davidiana
Talpa_ognevi
Talpa_caucasica
Talpa_stankovici
Talpa_levantis
Talpa_romana
Talpa_caeca
Talpa_europaea
Talpa_martinorum
Talpa_occidentalis
Talpa_aquitania
Talpa_altaica
Oreoscaptor_mizura
Mogera_tokudae
Mogera_wogura
Mogera_robusta
Mogera_imaizumii
Mogera_latouchei
Mogera_insularis
Mogera_kanoana
Mogera_hainana
Parascaptor_leucurus
Scaptochirus_moschatus
Euroscaptor_subanura
Euroscaptor_parvidens
Euroscaptor_longirostris
Euroscaptor_orlovi
Euroscaptor_kuznetsovi
Euroscaptor_malayanus
Euroscaptor_klossi
Scaptonyx_fusicauda
Neurotrichus_gibbsii
Urotrichus_talpoides
Dymecodon_pilirostris
Scapanus_townsendii
Scapanus_orarius
Scapanus_latimanus
Scapanus_occultus
Scapanus_anthonyi
Scalopus_aquaticus
Parascalops_breweri
Scapanulus_oweni
Alpiscaptulus_medogensis
40
30
20
10
0
Millions of years ago

**Talpidae BioGeoBEARS DEC+J**
**ancstates: global optim, 4 areas max. d=0.0031; e=0; j=0.0122; LnL=−115.06**

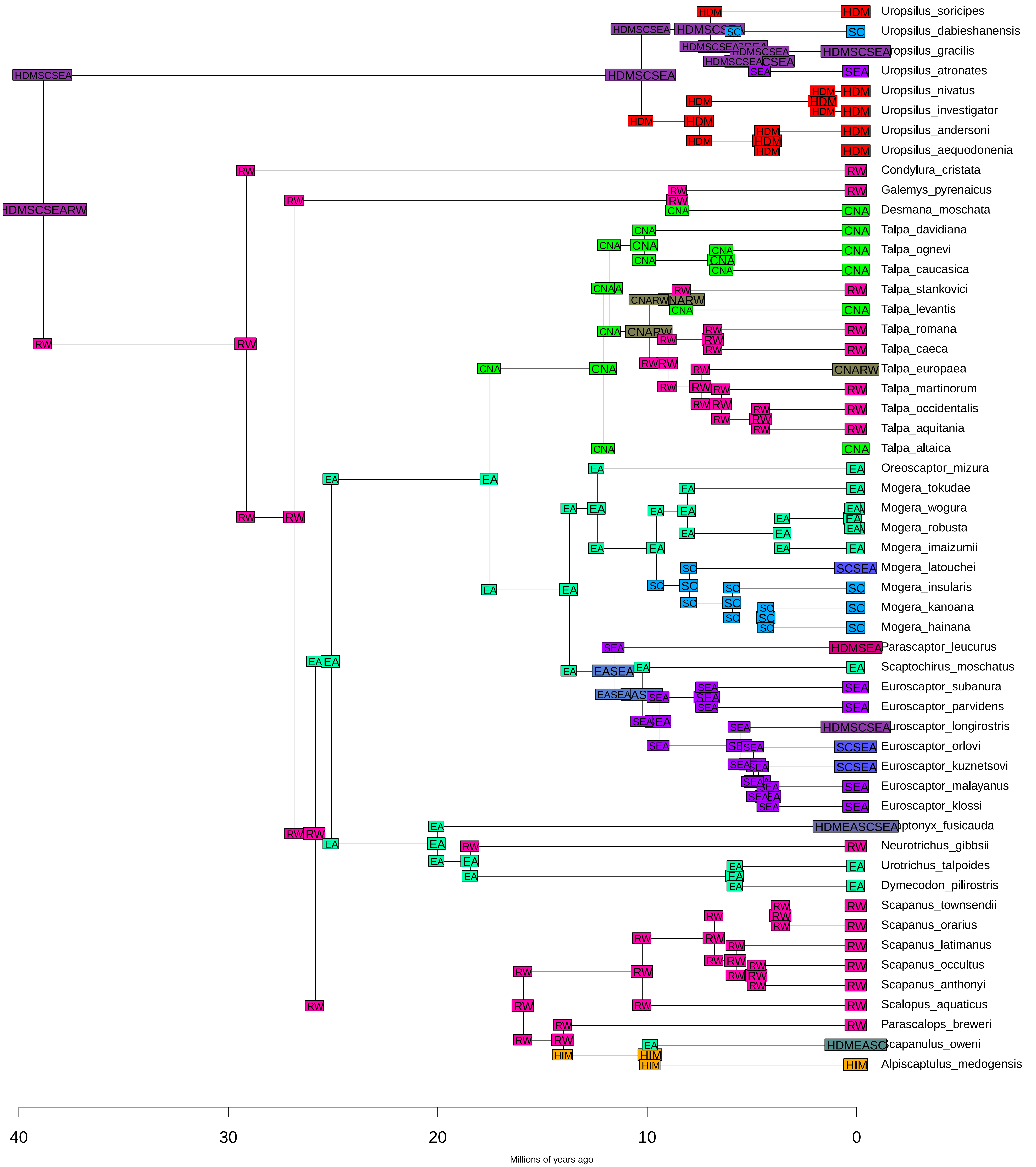

Arvicolinae BioGeoBEARS DEC
ancstates: global optim, 5 areas max. d=0.0093; e=0; j=0; LnL=-324.64

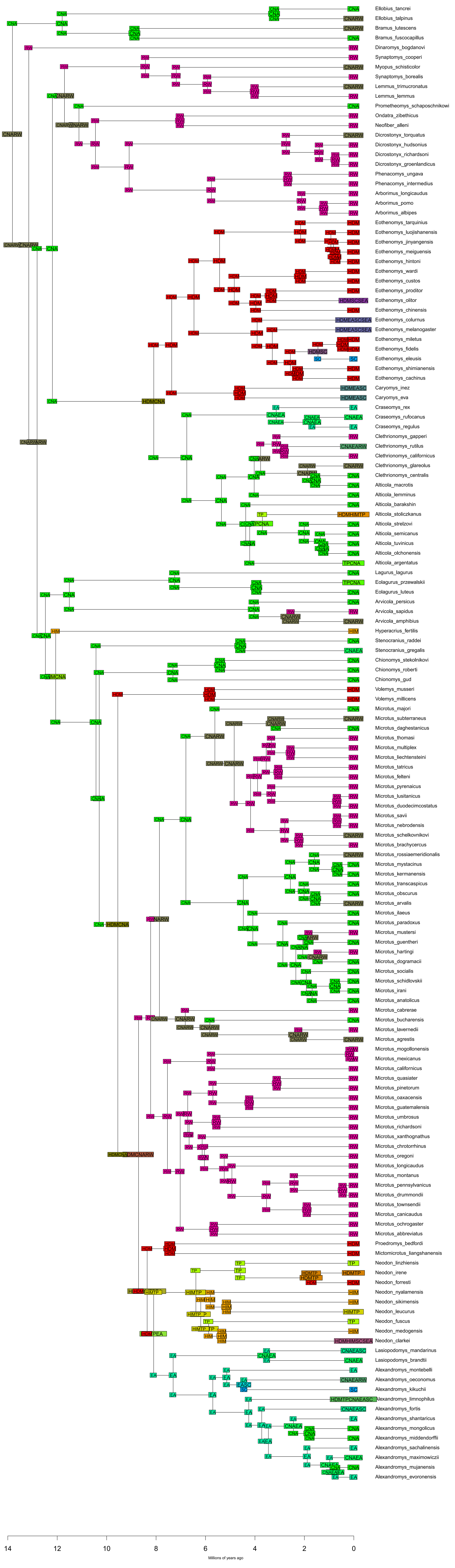

**Arvicolinae BioGeoBEARS DEC+J**
**ancstates: global optim, 5 areas max. d=0.0081; e=0; j=0.0066; LnL=-316.09**

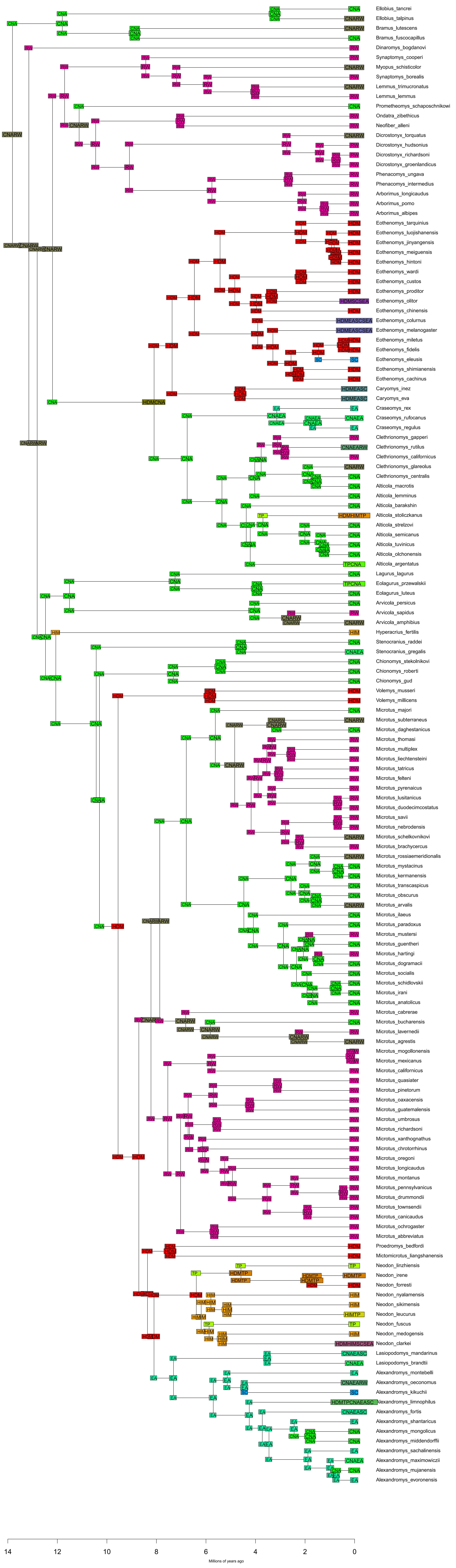

Supplement: Supplementary file 3 [file pnas.2602323123.sd02.pdf]
